# Self‐Complementary Dimers Based on Zwitterionic Halogen Bond Donors

**DOI:** 10.1002/chem.71076

**Published:** 2026-04-30

**Authors:** Dana Kutzinski, Raffaella Papagna, Elric Engelage, Lianne H. E. Wieske, Mate Erdelyi, Stefan M. Huber

**Affiliations:** ^1^ Fakultät für Chemie und Biochemie Ruhr‐Universität Bochum Bochum Germany; ^2^ Department of Chemistry for Life Sciences Uppsala University Uppsala Sweden

**Keywords:** halogen bonding, hypervalent iodine, molecular recognition, self‐assembly, zwitterions

## Abstract

Even though halogen bonding is routinely used in crystal engineering and beyond, motifs offering multipoint interactions are still rare. This applies in particular to self‐complementary systems which incorporate both halogen bond donating and accepting moieties. In this study, we present the first isolated homodimers based on a zwitterionic halogen bond donor, in which an iodolium core acts as Lewis acidic unit and a sulfonate group acts as Lewis base. Initial experiments with a carboxylate variant featured too strong coordination, leading to poor solubility. The optimized sulfonate systems, in contrast, resulted in strongly bound dimers with halogen bond distances of up to 2.73 Å. We anticipate this motif to form the basis for the further development of halogen‐bonding‐based linkers.

## Graphical abstract

In this study we present the first isolated zwitterionic homodimers based on halogen bonding (XB). After optimization of the structures by changing the Lewis base (LB) and the backbone, their dimerization was observed in gas phase, as well as, in solid state. With bond distances of up to 2.73 Å, they form the strongest XB based dimers to date.

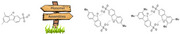

## 1 Introduction

**FIGURE 1:**
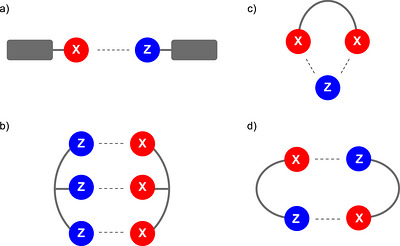
Different halogen bonding binding motifs: 1:1 binding (a), 2:1 binding (b), 3:3 multipoint binding (c), and self‐complementary binding (d). X = halogen atom and Z = Lewis basic atom.

Although halogen bonding (XB) is known since the 19^th^ century [1], it was defined by IUPAC only as late as 2013 [2, 3, 4] and is described as the non‐covalent interaction between an electrophilic halogenated compound (XB donor) and a Lewis base (XB acceptor). Since the 1990s, XB has found numerous applications, predominantly in crystal engineering [5, 6, 7, 8]. However, it has by now also very successfully been applied in solution, such as in molecular and anion recognition [9, 10, 11, 12], anion transport [13, 14], as well as in organocatalysis [15, 16, 17]. In contrast to the related hydrogen bonding, XB is more directional (with binding angles close to linearity), which places high demands on a rational design of the corresponding XB donor for any aspired application. The most frequently reported XB adducts feature a 1:1 interaction between donor and acceptor, which e.g. leads to the formation of infinite chains (Figure 1a) [5, 6, 7, 8], followed by 2:1 (Figure 1b), and 3:1 adducts that are usually applied as receptors [9, 10, 11, 12, 18, 19] and catalysts [15, 16, 17]. In contrast, multipoint XB interactions (Figure 1c) are very rare. In 2014, our group published the first example of a well‐defined three‐point XB interaction between an orthoamide and a tridentate XB‐donor [20].

Even more complex XB‐based multipoint interactions were designed by the groups of Aakeröy [21] and Diederich [22] in order to form molecular capsules. However, two different building blocks with multiple XB donor sites on one and multiple acceptor sites on the other part needed to be used. A topologically more elegant approach would involve the use of self‐complementary building blocks. In more general terms, self‐complementary XB‐based linkers (Figure 1d) would allow the rapid assembly of elaborate motifs, but they are difficult to design and synthesize (see below).

In fact, to date only very few self‐complementary XB motifs are known. One of these was reported by Stoddart et al. who observed intramolecular two‐point XB interactions between dihalopyromellitic diimides [23]. Moreover, the group of Philp elegantly employed neutral iodotriazoles with accompanying phenolate [24] or phosphine oxide counterparts [25], Bowling et al. used motifs based on the combination of pyridines with iodopolyfluoroarenes [26] or iodoarenes [27], and McGuirk et al. used 2‐iodooxazole for multidimensional XB based networks [28]. In these cases of self‐complementarity, the XB donor moiety was a neutral scaffold. It is well established by now, however, that cationic variants are typically stronger XB‐based Lewis acids, e.g. in organocatalysis [17, 29]. Iodine(III)‐derived XB donors in particular have been found to form very strong XB [30, 31, 32]. Consequently, self‐complementary dimers based on iodine(III) systems arguably constitute a solid basis for potent XB linkers, especially if the Lewis basic counterpart would be included in anionic fashion in an overall zwitterion. Such zwitterionic species have been reported and were structurally characterized [33, 34, 35], but in all cases they were based on acyclic iodonium compounds (which are often reactive and used as reagents [36]). None formed isolated self‐complementary dimers in the sense of Figure 1d. In parallel to our work [37], a closely related study by Postnikov, Resnati, Kukushkin, and coworkers described porous halogen‐bonded organic frameworks (XOFs) [35] that were based on zwitterionic 4‐(aryliodonio)‐benzenesulfonates. These acyclic compounds formed dimer‐like motifs, but within an overall polymeric structure.

**FIGURE 2:**
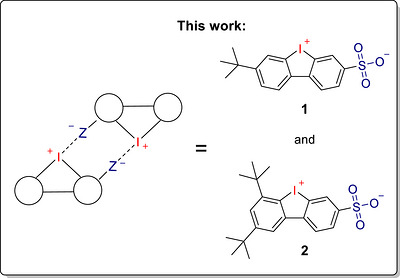
Self‐complementary assembly of zwitterionic iodolium‐based compounds.

In this study, we now report the first self‐assembled dimeric XB complexes based on zwitterionic iodolium derivatives **1** and **2** (Figure 2). Such cyclic compounds are typically more stable [38] and more Lewis acidic [39, 40] than acyclic systems, and thus seem particularly suitable for the task at hand.

## 2 Results and Discussion

Achieving XB‐based self‐complementary dimer assembly is much more challenging than it is, for example, for their hydrogen bonding analogues due to the following geometric constraints: (1) the bond angle ∡_R‐X_ **_···_**_LB_ (here with X = I) has to be close to linearity, (2) the interacting distance between the XB donor and acceptor should be less than the sum of their van‐der‐Waals radii [41, 42, 43], and (3) the intermolecular distance between both iodine atoms should be ≧ 4 Å (as the van‐der‐Waals radius of iodine is 1.98 Å [41]). A further goal was to develop compact self‐complementary units, in which donor and acceptor functionality are united in a reasonably close proximity without the use of spacers. Next to the advantages mentioned above, iodolium derivatives also feature a rigid core structure, which should allow a reliable prediction of dimer geometries. In principle, they possess two electrophilic axes on iodine [44], but only one of those (per iodine) was to be used for dimer formation.

**FIGURE 3:**
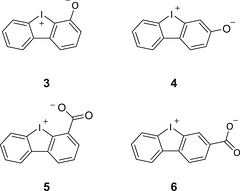
Zwitterionic iodolium derivatives as first candidate structures for self‐complementary dimer formation.

In search of appropriate structures, orientating DFT calculations (M06‐2X [45]/def2‐TZVP(D) [46, 47, 48]; see Supporting Information) were performed on compounds **3**–**6**, in which phenolate and carboxylate Lewis bases were placed in positions *ortho* or *meta* to iodine (Figure 3).

Table 1 summarizes the geometric parameters and Gibbs free energies obtained for the formation of the dimers of **3–6** in the gas phase and in solution (entries 1–4). According to the gas‐phase calculations, all dimer formations are exergonic.

**TABLE 1:** Geometric parameters of the iodolium dimers **1–6** and **14** obtained from gas‐ and solution‐phase calculations. ∡_C‐I_…_LB_ angles are given in °, d_I_…_LB_ distances in Å, and Gibbs free binding energies (including Grimme's D3 dispersion correction [49, 50]) in kcal·mol−^1^.

#	Cpd.	∡_C—I_…_LB_ ^a^	d_I_…_LB_ ^a^	ΔG^a^	ΔG_MeCN_ ^b^	ΔG_DCM_ ^b^
1	**3**	152	2.7	−8.3	24.7	1.6
2	**4**	150	2.9	−8.2	4.4	3.1
3	**5**	143	2.9	−4.5	7.0	5.1
4	**6**	164	2.4	−60.9	−13.1	−17.2
5	**14**	166	2.5	−48.9	−8.8	−10.7
6	**1**	168	2.5	−50.6	−7.3	−11.5
7	**2**	168	2.5	−48.3	−8.2	−11.3

^a^ Results obtained from the gas‐phase calculated with M06‐2X / def2‐TZVP.

^b^ Binding energies obtained from solution calculated with SMD18 [51] using parameters either for acetonitrile (MeCN) or dichloromethane (DCM).

**FIGURE 4:**
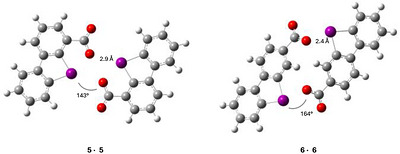
DTF‐calculated (M06‐2X TZVP(D)) self‐complementary dimers of **5** and **6** (with the Lewis base either in *ortho* or *meta* position) and corresponding C─I···O bond angles.

While the position of the Lewis basic part does not have a strong influence on the overall association strength for compounds **3** (‐8.2 kcal mol^−1^) and **4** (‐8.2 kcal mol^−1^), a big difference is observed for compounds **5** and **6** (−4.5 vs. ‐60.9 kcal mol^−1^). As depicted in Figure 4, a plausible rational for these marked energy differences between *ortho* and *meta* substitution in **5** and **6** is based on steric hinderance, which is in line with earlier studies by our group [30]. This effect also results in more linear C─I**···**O bond angles and shorter distances for the *meta* compound, with ∡143° and 2.9 Å for **5** and ∡164° and 2.4 Å for **6**, respectively.

With these results in hand, the formation of the self‐assembled dimers **3‐6** was also investigated computationally in solution, using a polar (MeCN) and a less polar (DCM) solvent. Now, only dimer **6** (the *meta*‐substituted variant of the carboxylate) is still thermodynamically favored in both solvents (Table 1, entry 4). As expected, stronger complex formation is predicted in the less polar solvent DCM. Overall, *meta*‐substituted carboxylate **6** outperforms phenolates **3** and **4** and even carboxylate **5** by a very noticeable margin of at least 17 kcal mol^−1^ or more in both solvents and is thus identified as most suitable zwitterionic iodolium compound for the aspired dimer formation.

**SCHEME 1:**
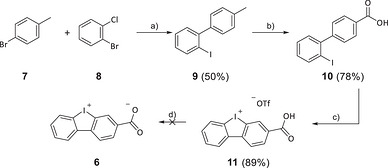
Synthesis of **6**: (a) Mg, I_2_, THF, 60°C, 2 h, (b) KMnO_4_, *t*BuOH/water, 4 h, reflux, (c), *m*CPBA, HOTf, DCM, r.t., 16 h, and (d) NaH, THF, 3 h, r.t.

Based on these promising results, compound **6** was synthesized via an arene coupling between *p*‐bromotoluene (**7**) and 1‐bromo‐2‐chlorobenzene (**8**) via a modified procedure from the Buchwald group [52]. Next, the methyl group was oxidized to the carboxylic acid **10** [23] and the iodolium was formed after oxidation [53], yielding compound **11** (Scheme 1).

**FIGURE 5:**
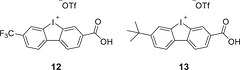
Synthesized iodolium compounds **12** and **13**.

For the final deprotonation, compound **11** was treated with sodium hydride to obtain the target zwitterionic iodolium compound **6**. Unfortunately, the obtained solid was not soluble either in organic or aqueous solvents and thus, NMR analysis could not be performed to confirm the formation of **6**. An IR measurement hinted at the presence of a carboxylate group and thus at successful deprotonation, but for any structural characterization, better solubility was clearly necessary. Accordingly, different substituents, such as *tert*‐butyl (in **12**) or CF_3_ (in **13**), were introduced at the iodolium backbone (Figure 5), which unfortunately lead to no noticeable improvement of solubility. Their synthesis is explained in the Supporting information.

At this point we speculated that monomers **6**, **12**, and **13** were associating too strongly with further molecules, due to the high Lewis basicity of the carboxylate. Thus, introducing a less Lewis basic group might be the key to a more soluble zwitterionic iodolium compound. Therefore, the carboxylate was changed to a sulfonate group, since organic sulfonic acids are stronger acids (*p*K_a_ of benzenesulfonic acid = −2.8) [54] than their carboxylic acid analogues (*p*K_a_ of benzoic acid = 4.2) [55]. Another possible explanation of the bad solubility could be the formation of oligomers. To prevent this, we planned to introduce a methyl group in *ortho* position to the iodine (zwitterion 14), as we had seen in other studies that methyl groups are able to block one axis of the XB donor [30].

Again, DFT calculations both in the gas phase and in solution were performed to check whether the formation of a self‐assembled dimer is feasible (Table 1, entry 7). The obtained geometric parameters of dimer **14** gave promising results, with a C─I**^…^** O bond angle close to linearity (166°) and a bond distance of 2.5 Å, which is still markedly shorter than the sum of their van‐der‐Waals radii (3.5 Å) [41]. The gas‐phase Gibbs free binding energy of ‐ 48.9 kcal·mol^−1^ is reduced to −8.8 kcal·mol^−1^ in MeCN and – 10.7 kcal·mol^−1^ in DCM, which indicates still very favorable dimer formation, but overall a less strong association than for carboxylate **6** – as aspired.

**SCHEME 2:**
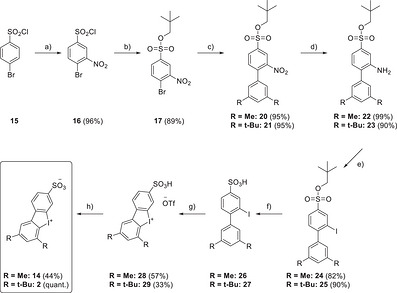
Synthesis of **14** and **2** over 9 steps starting from 4‐bromobenzenesulfony chloride (**15**): (a) H_2_SO_4_, HNO_3_, r.t., 18 h, (b) neopentyl alcohol, pyridine, CHCl_3_, 0°C to r.t., 17 h, (c) R = Me: 3,5‐dimethylphenylboronic acid (**18**) or R = *t*‐But: 2‐(3,5‐di‐*tert*‐butylphenyl)‐ ‐4,4,5,5‐tetramethyl‐1,3,2‐dioxaborolane (**19**), K_2_CO_3_, (PPh_3_)_2_PdCl_2_, 1,4‐dioxane/water, 70°C, 120 h, (d) H_2_, Pd/C, EtOH, r.t., 140 h, (e) *p*TsOH, NaNO_2_, KI, MeCN, 0°C to r.t., 20 h, (f) TFA, 70°C, 17 h, (g) *m*CPBA, HOTf, DCM, r.t., 16 h, and (h) pyridine, 16 h, r.t.

To confirm these results experimentally, we synthesized compound **14** starting from 4‐bromobenzenesulfonyl chloride (**15**). After nitration and protection of the sulfonyl chloride as a sulfonic acid ester, the aryl was coupled via Suzuki coupling to biaryl **20** (R = Me). The nitro group was reduced, and the resulting amine was converted to iodine by a Sandmeyer type reaction yielding compound **24** in 82% yield. After saponification of the ester to benzsulfonic acid **26**, the iodolium **28** was obtained by oxidation. After deprotonation with pyridine, the desired zwitterion **14** was isolated in 44% yield (Scheme 2).

**FIGURE 6:**
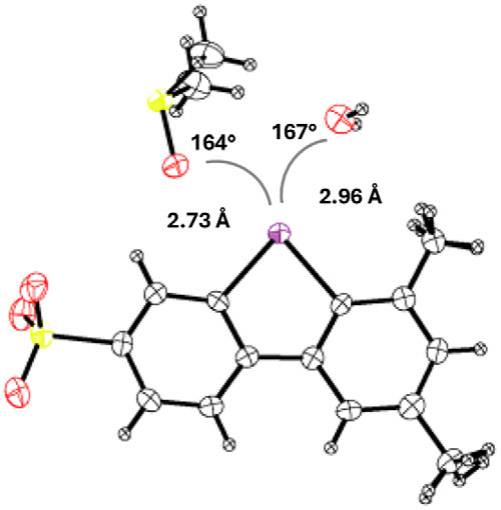
X‐ray structural analysis of the zwitterionic monomer **14**. Carbon atoms are shown in grey, iodine in purple, hydrogen in white, oxygen in red, and sulfur in yellow. Halogen bonds are highlighted in red including their bond angles and binding distances. Thermal ellipsoids at 50% probability level.

The use of a sulfonate group as LB and the introduction of the methyl groups in *ortho* position indeed led to a slight improvement of solubility of **14** compared to the solubility of **6**, so that a single crystal of **14** could be grown by the slow evaporation of the solvent DMSO [56]. However, in the solid state only a monomer was found (Figure 6). The crystal structure also revealed that in this case, the methyl group did not lead to sufficient blockage of the axis of the XB donor, as solvent molecules (DMSO and water) are still coordinating via both axes.

**FIGURE 7:**
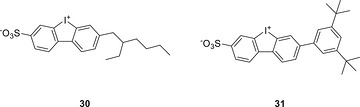
Synthesized iodolium compounds **30** and **31**.

In parallel, we had also undertaken further attempts to improve the solubility of the sulfonate‐based zwitterions via the modification of the backbone (yet without blocking groups in *ortho* position). Derivatives with branched alkyl chains (**30**) or bulky phenyl groups (**31**) (Figure 7) to break up the planarity of the compounds showed promising results in DFT calculations (see Supporting Information).

However, the experimental investigations did not show a significant improvement of solubility compared to zwitterion **14** and also no crystalline material could be obtained (for synthesis see Supporting Information). Nevertheless, for both zwitterions **30** and **31** we could confirm the dimerization in the gas phase by mass spectroscopy. The masses of the protonated dimers (*m/z* = 941.082 [2M+H]+ for **30** and 1094.0 [2M+H]+ for **31**) were detected in the mass spectrum. Additionally, we observed even bigger clusters like trimers or tetramers in the gas phase (Figures S101 and S104). For zwitterion **31**, the obtained isotope pattern was comparable to a simulated one (Figure S104) which further corroborated the presence of the different clusters.

We then further modified the zwitterions by implementing *tert*‐butyl groups in *ortho* (derivative **1**, Figure 1) or *meta* position (derivative **2**) to the iodine. Accompanying DFT calculations yielded results similar to the ones of zwitterion **14** (Table 1, entries 6 and 7); the C─I**^…^** O bond angles of 166 ° (compound **1**) and 168 ° (compound **2**) are close to linearity and also the bond distances of 2.5 Å are significantly shorter than the sum of their van‐der‐Waals radii [41]. The Gibbs free binding energies in gas phase are ‐49.2 kcal/mol (zwitterion **1**) and ‐48.3 kcal/mol (zwitterion **2**). As expected, these values are lower in MeCN (‐7.6 kcal/mol for **1** and ‐8.2 kcal/mol for **2**) and DCM (‐13.2 kcal/mol for **1** and ‐11.3 kcal/mol for **2**). These combined results were again promising for the self‐complementary dimerization of both zwitterions **1** and **2**.

**SCHEME 3:**
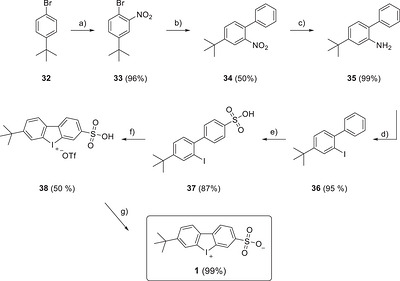
Synthesis of **1**: (a) HNO_3_, H_2_SO_4_, r.t., 18 h, (b) PhB(OH)_2_, Pd_2_(dba)_3_, SPhos, K_3_PO_4_, 1,4‐dioxane/water, 110°C, 15 h, (c) Pd/C, H_2_, EtOH, r.t., 72 h, (d) pTsOH, NaNO_2_, KI, 0°C to r.t., 15 h, (e) SO_3_ClH, chloroform, 3 h, (f), *m*CPBA, HOTf, DCM, r.t., 16 h, and (g) pyridine, r.t., 16 h.

To investigate these compounds experimentally, we synthesized zwitterion **1**, starting from 1‐bromo‐4‐(*tert*‐butyl)benzene (**32**) (Scheme 3). After nitration and coupling with phenylboronic acid, the nitro compound **34** was reduced to amine **35**. Finally, after Sandmeyer reaction, sulfonation, oxidation, and deprotonation of the resulting sulfonic acid **38**, the targeted zwitterionic iodolium compound **1** was obtained in high yields.

Zwitterionic iodolium **2** required a more elaborate synthetic pathway similar to that of zwitterion **14** illustrated in Scheme 2.

**FIGURE 8:**
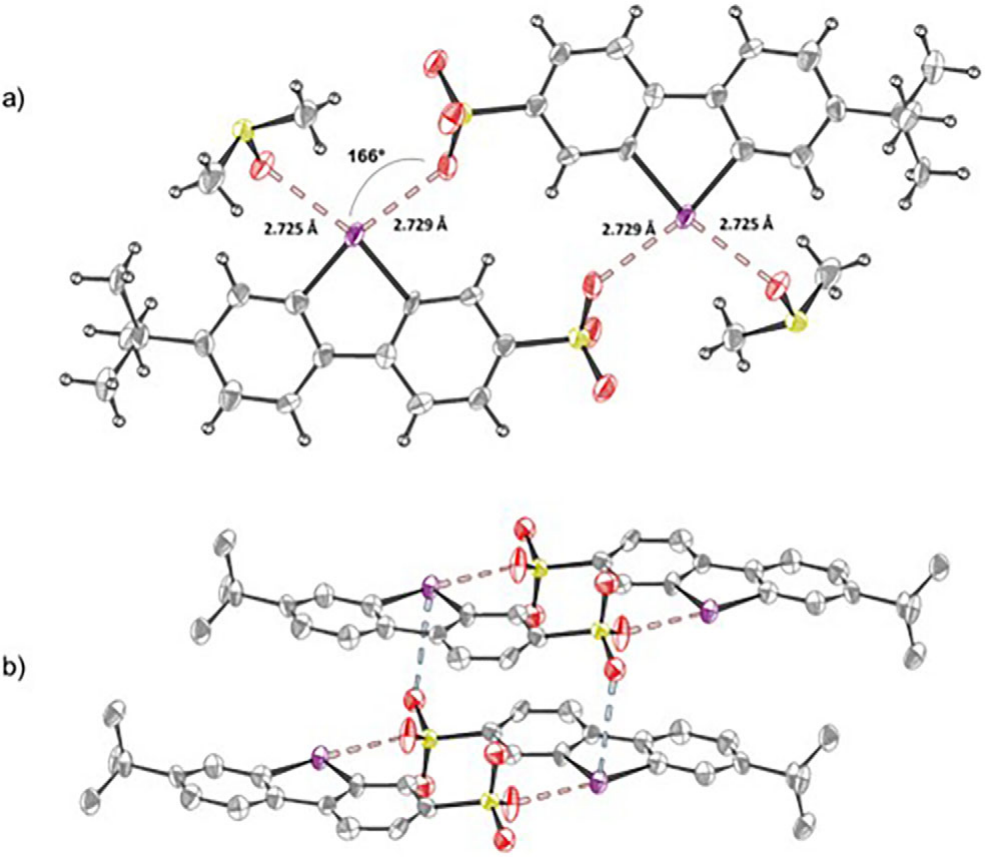
(a) X‐ray structural analysis of the self‐assembled dimer of compound **1**. (b) Cutout of the unit cell of dimer **1**. Hydrogens in (b) are omitted for clarity. Carbon atoms are shown in grey, iodine in purple, hydrogen in white, oxygen in red, and sulfur in yellow. Halogen bonds are highlighted in red including their bond angles and binding distances, and electrostatic interactions are indicated in blue. Thermal ellipsoids at 50% probability level.

**FIGURE 9:**
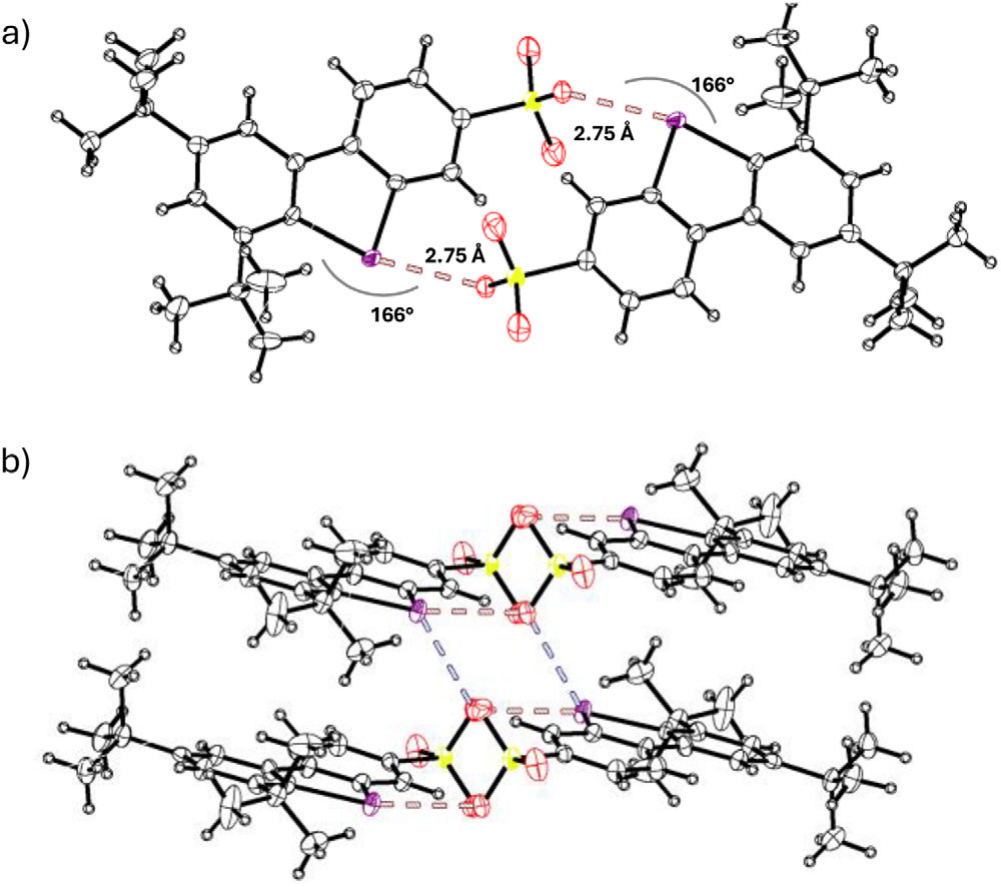
(a) X‐ray structural analysis of the self‐assembled dimer of compound **2**. (b) Cutout of the unit cell of dimer **2**. Carbon atoms are shown in grey, iodine in purple, hydrogen in white, oxygen in red, and sulfur in yellow. Halogen bonds are highlighted in red including their bond angles and binding distances, and electrostatic interactions are indicated in blue. Thermal ellipsoids at 50% probability level.

Both zwitterionic iodolium compounds **1** and **2** still feature limited solubility but are at least soluble in DMSO. Consequently, single crystals could again be grown by slow evaporation of this solvent. Indeed, the targeted self‐complementary dimer was formed for **1** and **2**, and it contains very pronounced halogen bonds between iodine and one oxygen of the sulfonate. The bond angles are close to linearity ∡_C‐I···O_ = 166° and the bond distances are *d*_I‐O_ = 2.73 Å (22% shorter than the sum of their van‐der‐Waals radii (Figure 8a) for **1** and 2.75 Å (21% shorter than the sum of their vdW radii (Figure 9a)) for **2** [41]. To the best of our knowledge, these are the strongest XB in a homodimeric assembly to date [57].

The obtained geometric parameters are in good agreement with both our gas‐phase calculations (Table 1, entry 6 and 7) and with the criteria listed above for obtaining halogen bond‐based self‐complementary dimers.

The second electrophilic axis of each iodolium center in zwitterion **1** features a further strong halogen bond to a DMSO molecule (*d*_I‐O_ = 2.725 Å and ∡_C‐I_ **_···_**_O_ = 167°). For zwitterion **2**, the tert‐butyl groups are blocking the second axis of the iodine(III), so that there is no XB to a solvent molecule. In addition, a further packing analysis shows that two self‐assembled dimers are stacked and that the flat planes are interconnected via electrostatic interactions between the cationic iodine and an oxygen of the sulfonate moiety (Figures 8b and 9b, blue dots). This interaction, however, is markedly weaker than the dimer‐forming XB, as is evident from the corresponding I‐O distance of 3.39 Å for **1** and 3.31 Å for **2** (which is equivalent to 96% respectively 95% of the sum of the vdW radii).

For the zwitterionic iodolium **2**, it was also possible to obtain a single crystal by the slow evaporation of methanol. Again, a self‐complementary dimer, which is based on strong halogen bonding (with ∡_C‐I···O_ = 166° and *d*_I‐O_ = 2.75 Å), was observed (see Figure S105).

After obtaining the aspired zwitterionic iodolium compounds and demonstrating their dimer formation in the solid‐state, DOSY [58] measurements were performed to evaluate whether this dimer also forms in solution. Since the zwitterions were sufficiently soluble in DMSO only, we recorded DOSY experiments on a solution of **1** or **2** in DMSO‐*d_6_* in various dilutions (see Supporting Information). An identical diffusion coefficient, *D* = 1.8·10^−10^ m^2^·s^−1^ (for **1**) and 1.67·10^−10^ m^2^·s^−1^ (for **2**), was observed at all concentrations. This indicates a hydrodynamic radius [59] of *r*_H_ = 6 Å (for **1**) and *r*_H_ = 8 Å (for **2**), which corresponds to those of the respective monomers. This may be explained by one DMSO molecule coordinating each electrophilic axis of the iodine centers in solution (somewhat similar to the solid‐state structure of **14**, Figure 6); apparently, this solvation is strong enough to prevent dimer formation. Dimerization would have been expected to result in a decreased diffusion coefficient at higher concentrations, which has not been observed.

## 3 Conclusion

This study introduced zwitterionic self‐complementary halogen bond donors based on iodolium carboxylates and sulfonates, and demonstrated their self‐assembly into isolated homodimers in the solid state. DFT calculations had indicated that Lewis basic groups in *meta* position to the iodine in iodolium compounds should allow strong self‐association via inter‐molecular halogen bonding. The originally chosen carboxylate group as Lewis basic unit was found to lead to insoluble zwitterions, likely due to overly strong coordination. Consequently, the carboxylate was replaced by a sulfonate group. These sulfonate‐based systems were soluble in DMSO (only), could thus be structurally characterized, and clean dimeric adducts with short iodine‐oxygen contacts were found in two cases. To the best of our knowledge, the presented zwitterions form the strongest XB based homodimers in the solid state to date. Additional changes in the backbone of the iodolium compounds such as the introduction of bulky groups or branched alkyl chains did not lead to a further improvement of solubility. In solution, no dimer formation was observed via NMR spectroscopy, which could possibly be explained by the strong coordination of the solvent to the two electrophilic axes of the XB donor.

The self‐complementary motifs presented herein represent a very promising core structure for the further development of XB‐based linkers. These would allow the construction of supramolecular aggregates from multiple copies of the same building block. To realize our ultimate goal, such self‐assembly in solution, further studies on structural modifications are necessary (and ongoing).

## Conflicts of Interest

The authors declare no conflicts of interest.

## Supporting information




**Supporting file**: chem71076‐sup‐0001‐SuppMat.pdf.

## Data Availability

Data available from the authors upon reasonable request.
