# Supplementary material for: Self‐Complementary Dimers Based on Zwitterionic Halogen Bond Donors
Source: Chemistry. 2026 Apr 30;32(27):e71076. doi: 10.1002/chem.71076 (PMC13380381; doi:10.1002/chem.71076)
Supplement: Supplementary file 1 — Supporting file: chem71076‐sup‐0001‐SuppMat.pdf. [file CHEM-32-e71076-s001.pdf]

Self-complementary Dimers Based on Zwitterionic Halogen Bond Donors

Dana Kutzinski,‡[a] Raffaella Papagna,‡[a] Elric Engelage,[a] Lianne H. E. Wieske,[b] Máté Erdélyi,[b] and Stefan M. Huber*[a]

[a] D. Kutzinski, Dr. R. Papagna, Dr. E. Engelage, Prof. Dr. S. M. Huber
Fakultät für Chemie und Biochemie, Ruhr-Universität Bochum
Universitätsstraße 150, 44801 Bochum (Germany)
E-Mail: stefan.m.huber@rub.de

[b] Dr. L. H. E. Wieske, Prof. Dr. M. Erdélyi
Department of Chemistry – BMC
Husargatan 3, 752 37 Uppsala (Sweden)

‡These authors contributed equally to this work.

Table of Contents

1. Experimental Procedures
2. X-Ray Data
3. DOSY experiments
4. Computational Details
5. References

1. Experimental Procedures

1.1 Chemicals and General Information

All the used chemicals were commercially obtained from *Sigma Aldrich/Merck, Acros, Alfa Aesar, Carbolution Chemicals, Carl Roth, Chempur, Fluorochem, Fluka, BLDPharm, and TCI* and used, unless otherwise noted, without further purifications. For all reactions that were performed under inert conditions, oven dried Schlenk flasks were used and flooded with argon before use. Dry solvents as dichloromethane (DCM), tetrahydrofuran (THF), and diethyl ether (DEE) were obtained from a *MBraun MP* SPS-800 and stored over 4 Å molecular sieve. Methanol, acetonitrile (MeCN), and chloroform were bought in analytical grade quality and also dried over 4 Å molecular sieves. For diisopropylamine, triethylamine, and pyridine, fresh bottles were opened, molecular sieves were added, and the bottles closed with *AcroSeals* (black), obtained from old n-BuLi bottles. Furthermore, all reagents were either added under argon counter-flow or via a septum using plastic *NORMJECT* syringes and *BRAUN Luer* cannulas. Thin layer chromatography (TLC) was performed using either *Merck* plates (silica gel 60, F254) or aluminum oxide plates from *Macherey-Nagel* (ALUGRAM ALOX N/UV254) and the detection of the substance was obtained by fluorescence detection under UV light (wavelength λ = 254 nm). Accordingly, column chromatography (CC) was performed using silica gel (grain size 0.04-0.063 mm, *Macherey-Nagel* Si60) or aluminum oxide (grain size: 0.063-0.200 mm, *Merck*, aluminum oxide 90 active neutral).

1.2 Solvents

Common solvents as DCM, DEE, THF, ethyl acetate (EtOAc), and pentane were bought in technical grade quality and distilled before using. Deuterated solvents as $CDCl_3$, Acetonitrile-d_3, CD_2Cl_2, Methanol-d_4, DMSO-d_6, and D_2O were obtained either from *Deutero, Eurisotop,* or *Sigma Aldrich* and dried over molecular sieves.

1.3 Analytical Methods

NMR-Spectra were recorded on a *Bruke*r DPX-200, DPX-250, DPX-400, AVIII-300 or AVIII-400 spectrometer at 298 K and the spectra analyzed using *MestreNova.* The chemical shifts were recorded in δ (ppm), the coupling constants in Hertz (Hz), and the corresponding multiplicity are labeled with s (singlet), d (duplet), t (triplet), q (quartet), p (pentet), m (multiplet), and combination of those. The signal assignment of all protons and carbons of all new compounds was done using COSY, HSQC, and HMBC spectra. **Infrared-Spectra** were recorded on a *Shimadzu IR Affinity* – 1S spectrometer equipped with a *Specac-Quest* ATR module and the wavenumbers reported in cm^{-1}. The intensity peaks are labeled as followed: very strong (vs), strong (s), medium (m), weak (w), very weak (vw), and broad (b). **Elemental analysis** (C,H,N,S) was carried out on a vario Micro cube from *Elementar Analysentechnik*. **Gas chromatography–mass spectrometry (GC-MS)-**Spectra were measured on an *Agilent* 7820GC/5977B-EI-MDS, both equipped with a 30 m HP5MS column using Helium as carrier gas. **Mass-Spectra** were recorded either on a *Bruker Daltonics Esquire6000* instrument (ESI) or on a *VG Instruments Autospec / EBEE-Geometry* (EI) or *Xevo G2-XS QTof* for high resoltion mass (HR). **Karl-Fischer-Titrations (KF):** To determine the water content of solvents, a Karl Fischer titration device using a *SI Analytics/Xylem* TitroLine 7500 KF trace with a *Honeywell* (former *Fluka*) Hydranal-Coulomat-AD solution was used. **X-Ray**: Single crystals were analyzed on a *Rigaku* XtaLAB mini, equipped with a 600 W Mo micro-fine focus glass sealed tube, graphite monochromator (Mo Kα) and CCD detector. Crystals were mounted in Hampton CrypLoops using Parabar/Paratone or GE/Bayer silicone grease. The crystals were cooled using an *Oxford Cryososystems* Desktop Cooler, which cools pre-dried air to 170 K. Data were recorded and reduced using the CrysalisPro[1] Software. Structures were solved using WinGX[2] in combination with ShelXT and refined with shelXle[3,4] and ShelXL. Graphics were obtained from Diamond 4.[5]

1.4 Synthesis

Synthesis of known compounds

Methyl 4-chloro-3-nitrobenzoate,[6] (3,5-dimethylphenyl)boronic acid,[7] (4-bromophenyl)-trimethylsilane[8] and 1-bromo-(4-tert-butyl)-nitrobenzene[9] were synthesized as described in literature.

Synthesis of 2-Iodo-4'-methyl-1,1'-biphenyl (9)

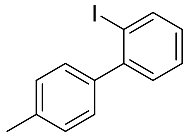

Chemical Formula: $C_{13}H_{11}I$
Molecular Weight: 294.14 $^{g}/_{mol}$

2-Iodo-4'-methyl-1,1'-biphenyl was synthesized according to a modified procedure from Buchwald et. al.[10] A 500 ml three-neck flask was charged with 250 ml dry THF, 9.1 g (374.4 mmol, 2.3 eq) of magnesium and small amounts of iodine. Once the magnesium is activated, 22.15 ml (30.8 g, 180 mmol, 1.1 eq) of 1-bromo-4-methylbenzene (**7**) are added dropwise under argon counterflow and the mixture is stirred at 60 °C for 2 h. Once the mixture is cooled down to room temperature, 18.8 ml (30.6 g, 160 mmol, 1 eq) of 1-bromo-2-chlorobenzene (**8**) are added dropwise and the mixture is stirred for additional 2 h at 60 °C. Afterwards, the dark reaction mixture is cooled down to 0 – 5 °C and quenched with 40.6 g (160 mmol, 1 eq) iodine and stirred overnight at r.t. Next, an aqueous solution of sodium thiosulphate is added to the mixture until the characteristic iodine color disappears. The organic phase was separated, and the aqueous solution is extracted three times with diethyl ether, and the combined organic phases are washed with brine, dried over magnesium sulphate, and the solvent removed in vacuo The brownish residue was distilled under vacuum to afford 23 g (50%) of a yellowish oil. The obtained NMR data fit those reported in literature.[11]

^{1}H-NMR (300 MHz, DMSO-d_6): δ = 7.96 (dd, *J* = 7.9, 1.2 Hz, 1H, 7.45 (td, *J* = 7.4, 1.2 Hz, 1H), 7.30 (dd, *J* = 7.6, 1.7 Hz, 1H), 7.23 (m, 4H), 7.10 (ddd, *J* = 7.9, 7.3, 1.8 Hz, 1H), 2.36 (s, 3H) ppm.

^{13}C-NMR (75 MHz, DMSO-d_6): δ = 145.9, 140.8, 139.2, 136.8, 130.1, 129.0, 128.9, 128.6, 128.4, 98.8, 20.8 ppm.

EI-MSD: m/z (+) = calc. 294.99, found: 294.12

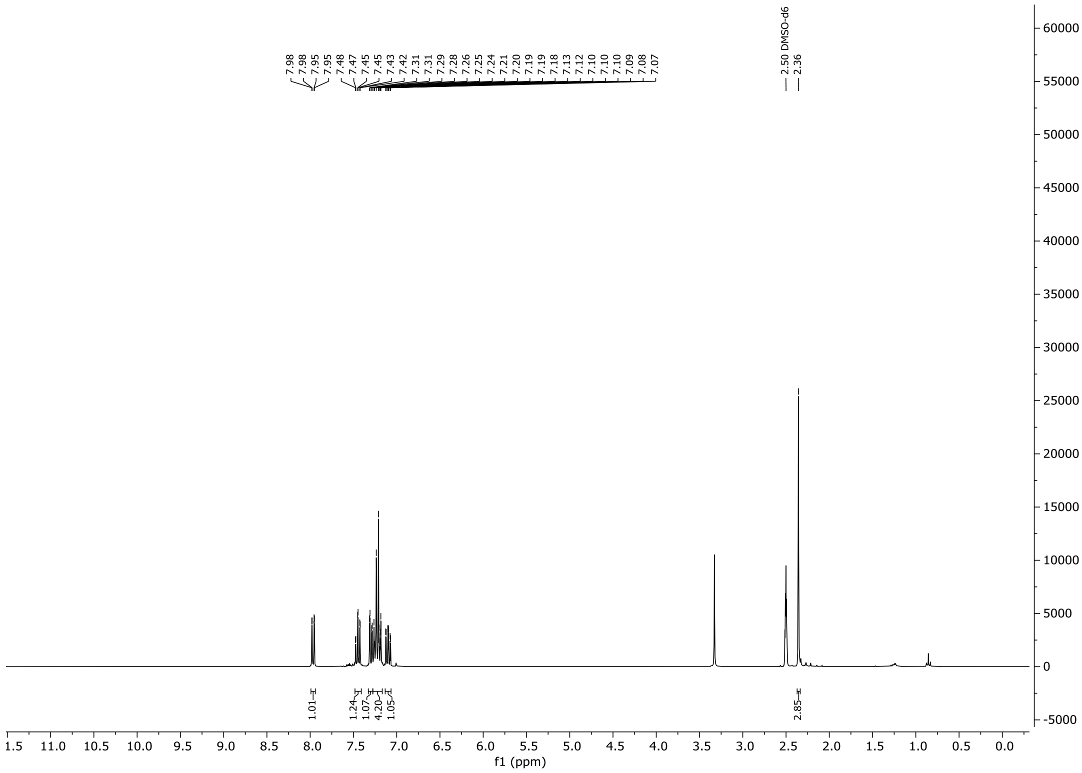

Figure S1: ^{1}H NMR spectrum (300 MHz) of compound **9** in DMSO-d$_6$.

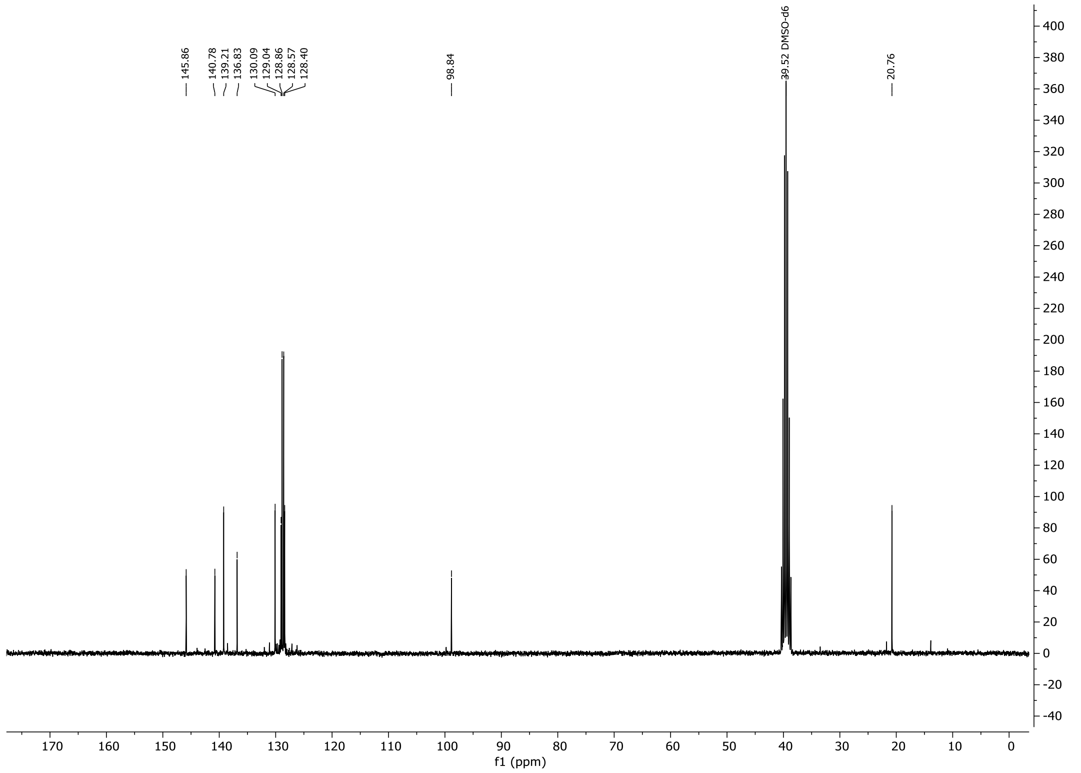

Figure S2: ^{13}C NMR spectrum (75 MHz) of compound **9** in DMSO-d$_6$.

Synthesis of 2'-Iodo-[1,1'-biphenyl]-4-carboxylic acid (10)

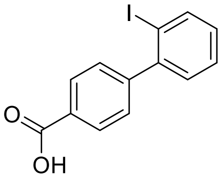

Chemical Formula: $C_{13}H_9IO_2$
Molecular Weight: 324.12 $^g/_{mol}$

2-Iodo-4'-methyl-1,1'-biphenyl was synthesized according to the procedure of Stoddart et al.[12] Compound **9** (6.2 g, 21 mmol, 1.0 eq) and $KMnO_4$ (17.24 g, 109 mmol, 5.2 eq) were dissolved in a *t*BuOH:water (1:1, v/v, 86 ml) mixture and refluxed until the purple color of the solution had disappeared and turned brown. After cooling to r.t, additional $KMnO_4$ (4.3 g, 27.3 mmol, 1.30 eq) were added and the mixture refluxed again for additional 4 h. Ethanol (10 ml) was added to the refluxing mixture to quench remaining $KMnO_4$ and the brown solids were removed by hot filtration. After the filtrate had cooled to r.t, it was acidified with aq. 2 M HCl solution and the solvent removed in vacuo. The remaining aq. solution was basified with aq. NaOH to dissolve the carboxylic acid and the remaining solid filtered. The filtrate was acidified again with HCl solution and the precipitate filtered and dried under high vacuum to obtain 5.3 g (78%) an off-white solid.

^{1}H-NMR (300 MHz, DMSO-*d*$_6$): δ = 13.05 (s, 1H), 8.02 (m 2H), 7.53 – 7.08 (m, 6H) ppm.

^{13}C-NMR (75 MHz, DMSO-*d*$_6$): δ = 167.0, 147.8, 145.0, 139.4, 130.0, 129.7, 129.4, 129.1, 128.5, 98.1 ppm.

ATR-IR [cm^{-1}]: 2965 (s), 2710 (s), 2536 (w), 1669 (s), 1606 (m), 1567 (w), 1457 (w), 1419 (w), 1315 (m), 1285 (s), 1183 (w), 1130 (w), 1102 (w), 1014 (w), 997 (m), 928 (m), 871 (m), 851 (m), 777 (m), 755 (vs), 720 (s9, 701 (s), 658 (s), 627 (w), 544 (s), 516 (s), 461 (w).

ESI-MS: m/z (+) = calc. 342.0 [M+NH_4]+, found 342.2 [M+NH_4]+

CHNS [%] calc.: C: 48.10, H: 2.80
found: C: 49.25, H: 2.83

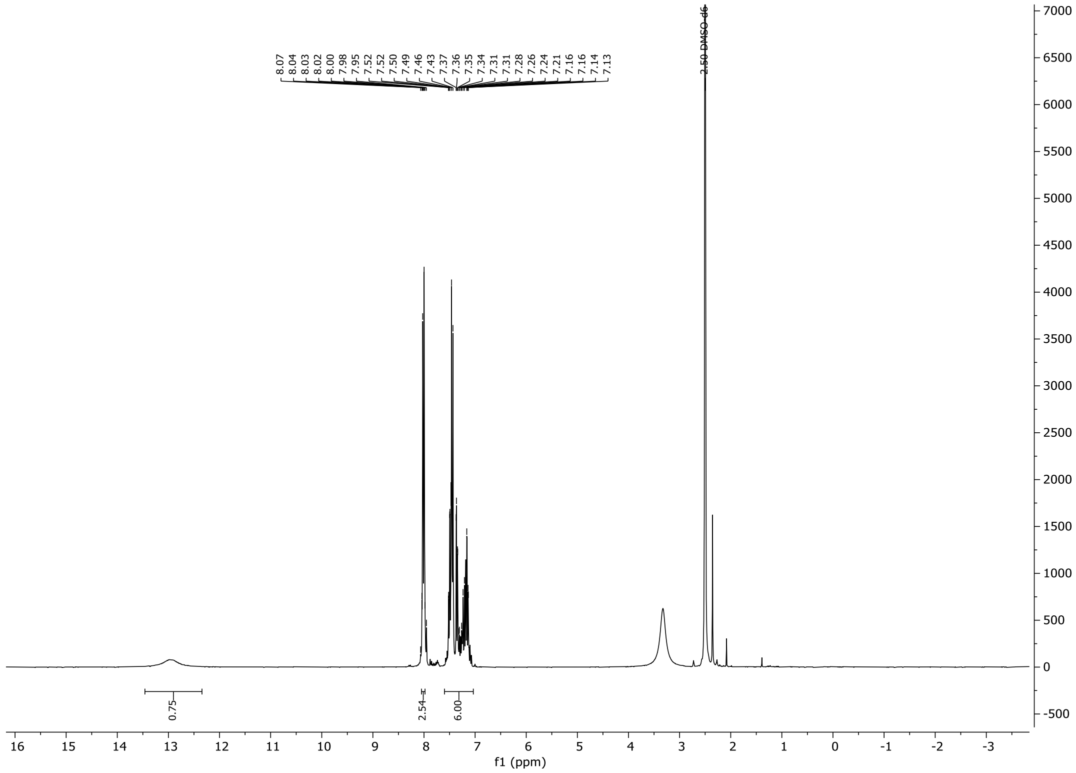

Figure S3: ^{1}H NMR spectrum (300 MHz) of compound **10** in DMSO-*d*$_6$.

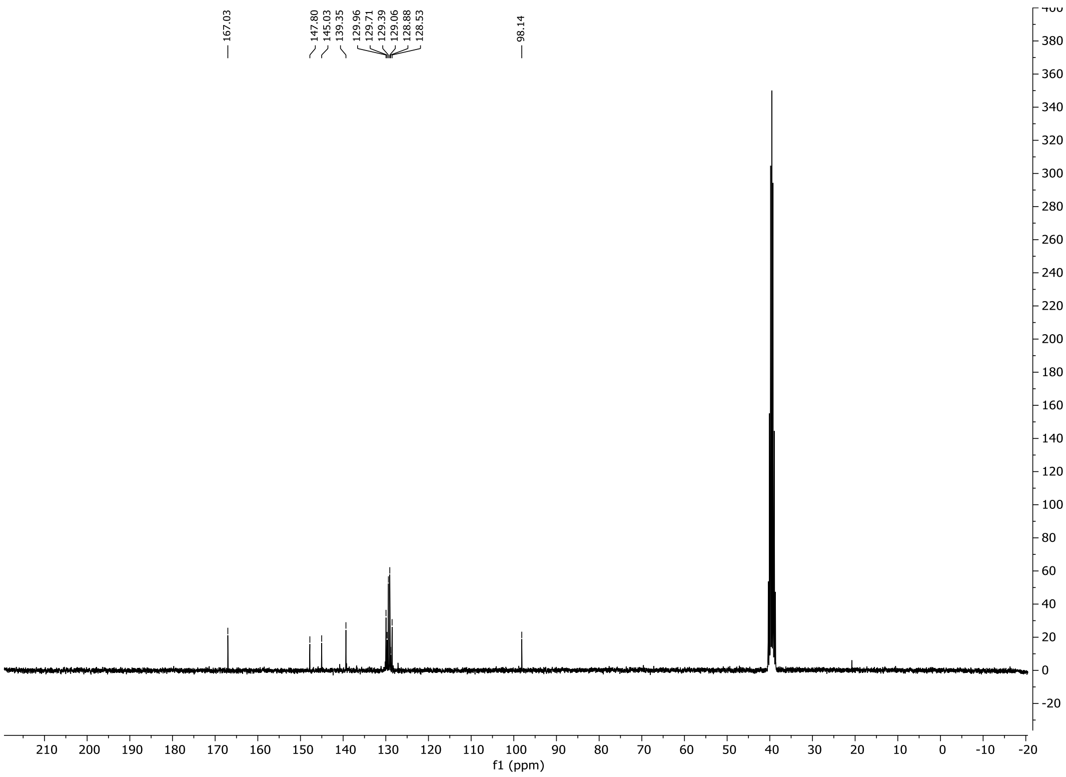

Figure S4: ^{13}C NMR spectrum (75 MHz) of compound **10** in DMSO-*d₆*.

Synthesis of 3-Carboxydibenzo[b,d]iodol-5-ium trifluoromethanesulfonate (11)

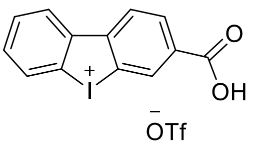

Chemical Formula: $C_{14}H_8F_3IO_5S$
Molecular Weight: 472,1727

Compound **11** was synthesized according to a modified procedure published by Olofsson et al.[13] 2.00 g (6.17 mmol) of biphenyl **10** were suspended in DCM (12.3 ml, 0.5 M) and cooled to 0 °C. Afterwards, 2.07 g (77% active oxidant, 9.26 mmol, 1.5 eq) of *meta*-chloroperoxybenzoic acid (*m*CPBA) were added and stirred for 30 min at this temperature. Triflic acid (HOTf) (1.09 ml, 12.34 mmol, 2.0 eq) was slowly added and the reaction mixture was warmed up to r.t and stirred overnight. The solvent was removed in vacuo and the resulting solid suspended in 20 ml diethyl ether and stirred for 15 min. The precipitate was filtered and the solid washed with small amounts of diethyl ether. Finally, 2.6 g (89%) of a white solid were obtained.

^{1}H-NMR (300 MHz, DMSO-d_6): δ = 8.77 (d, *J* = 1.5 Hz, 1H), 8.55 (t, *J* = 7.0 Hz, 2H), 8.31 (dd, *J* = 8.2, 1.6 Hz, 1H), 8.24 (dd, *J* = 8.2, 1.1 Hz, 1H), 7.89 (t, *J* = 7.0 Hz, 1H), 7.77 (td, *J* = 7.8, 7.2, 1.5 Hz, 1H) ppm.

^{13}C-NMR (75 MHz, DMSO-d_6): δ = 167.0, 147.8, 145.0, 139.4, 130.0, 129.7, 129.4, 129.1, 128.5, 98.1.

^{19}F-NMR (235 MHz, DMSO-d_6): δ = -77.73 ppm.

ATR-IR [cm^{-1}]: 3142 (vw), 1689 (vs), 1594 (w), 1556 (vw), 1397 (w), 1286 (s), 1273 (s), 1240 (s), 1216 (vs), 1166 (s), 1117 (m), 1023 (s), 986 (w), 905 (w), 821 (w), 799 (w), 757 (s), 726 (m), 652 (w), 631 (s), 574 (m), 514 (s), 412 m).

FD-MS: m/z (+) = calc.: 322.9564 [M]+, found: 322.9560 [M]+

CHNS [%] calc.: C: 35.61, H: 1.71, S: 6.79
found: C: 36.27, H: 1.75, S: 6.80

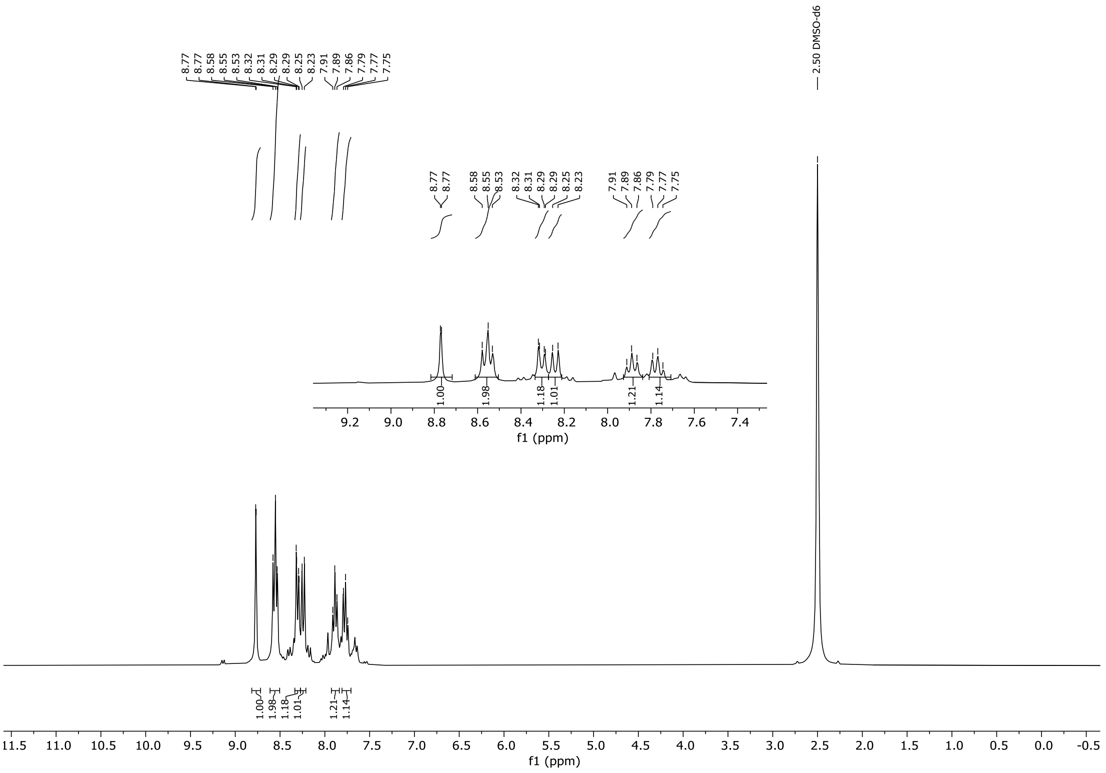

Figure S5: ^{1}H NMR spectrum (300 MHz) of compound **11** in DMSO-d_6.

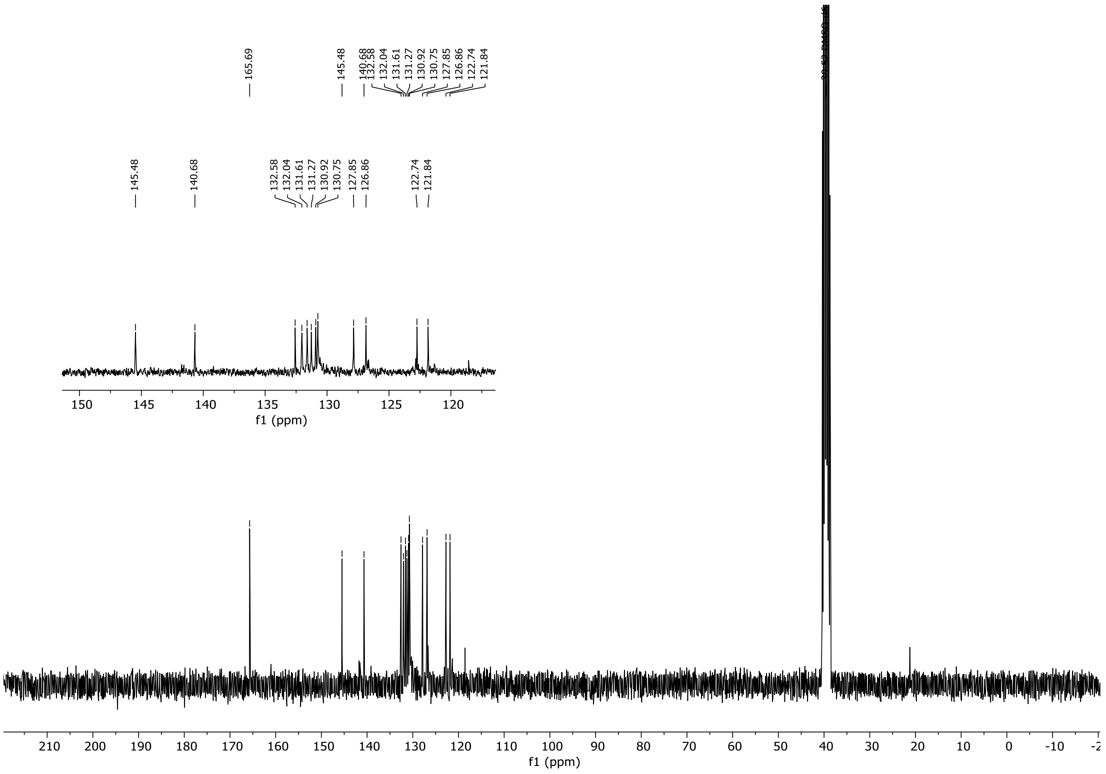

Figure S6: ^{13}C NMR spectrum (75 MHz) of compound **11** in DMSO-d_6.

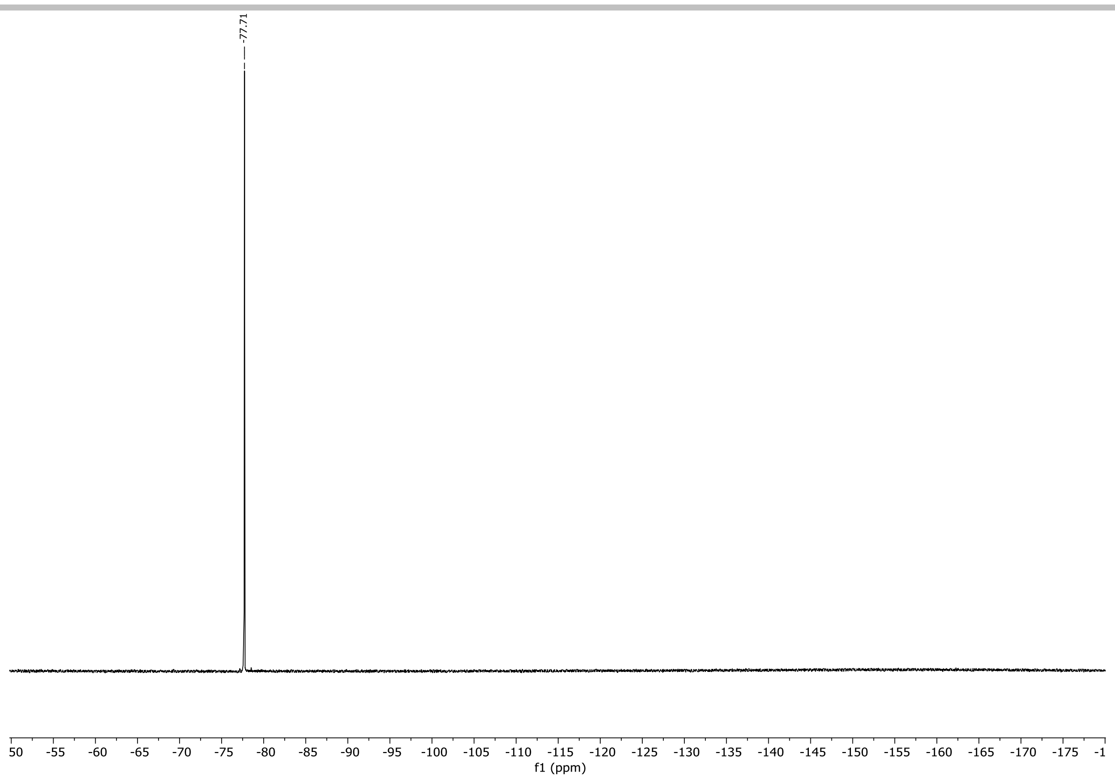

Figure S7: ^{19}F NMR spectrum (235 MHz) of compound **11** in DMSO-d_6.

Synthesis of Methyl 2-nitro-4'-(trifluoromethyl)-[1,1'-biphenyl]-4-carboxylate (S1)

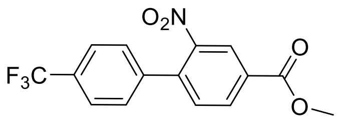

Chemical Formula: $C_{15}H_{10}F_3NO_4$
Molecular Weight: 325.24 $^{g}/_{mol}$

Methyl 4-chloro-3-nitrobenzoate (2.41 g, 11.18 mmol, 1 eq), 2.34 g (12.3 mmol, 1.1 eq) of (4-(trifluoromethyl)phenyl)boronic acid, 3.1 g (22.36 mmol, 2 eq) of potassium carbonate, and 2 mol% of tetrakis(triphenylphosphine)palladium(0) (258.36 mg, 0.233 mmol) were added under inert gas conditions to 25 ml of a degassed (via freeze-pump-thaw) water/toluene (2:1) solution and stirred at 110 °C overnight. After cooling to room temperature, the dark mixture was diluted with 150 ml of diethyl ether and filtered through a plug of celite. After washing the celite with diethyl ether, the organic phase was washed with water and brine and dried over $MgSO_4$. The solvent was subsequently removed under reduced pressure and the crude product purified via column chromatography (silica gel) using first pentane and then diethyl ether to achieve 2.5 g (69%) of an off-white solid.

^{1}H-NMR (300 MHz, $CDCl_3$): δ = 8.57 (d, J = 1.7 Hz, 1H), 8.30 (dd, J = 8.0, 1.7 Hz, 1H), 7.73 – 7.69 (m, 2H), 7.53 (d, J = 8.0 Hz, 1H), 7.46 – 7.43 (m, 2H), 4.00 (s, 3H) ppm.

^{13}C-NMR (75 MHz, $CDCl_3$): δ = 164.8, 149.1, 140.4, 139.2, 133.4, 132.3, 131.4, 130.9, 128.4, 126.0, 125.7, 122.2, 53.1 ppm.

^{19}F-NMR (235 MHz, $CDCl_3$): δ = -62.72 ppm.

ATR-IR [cm^{-1}]: 3089 (vw), 2955 (vw), 1714 (m), 1612 (w), 1564 (vw), 1528 (w), 1484 (vw), 1436 (w), 1330 (m), 1305 (m), 1287 (m), 1243 (vw), 1159 (w), 1104 (m), 1064 (m), 1005 (w), 972(vw), 936 (vw), 921 (vw), 899 (vw), 863 (w), 833 (w), 764 (w), 742 (w), 702 (w), 688 (vw), 614 (w).

FD-MS: m/z (+) = calc.: 325.0562 [M]+, found: 325.0591 [M]+

CHNS [%] calc.: C: 55.41, H: 3.25, N: 4.28
found: C: 55.39, H: 3.10, N: 4.31

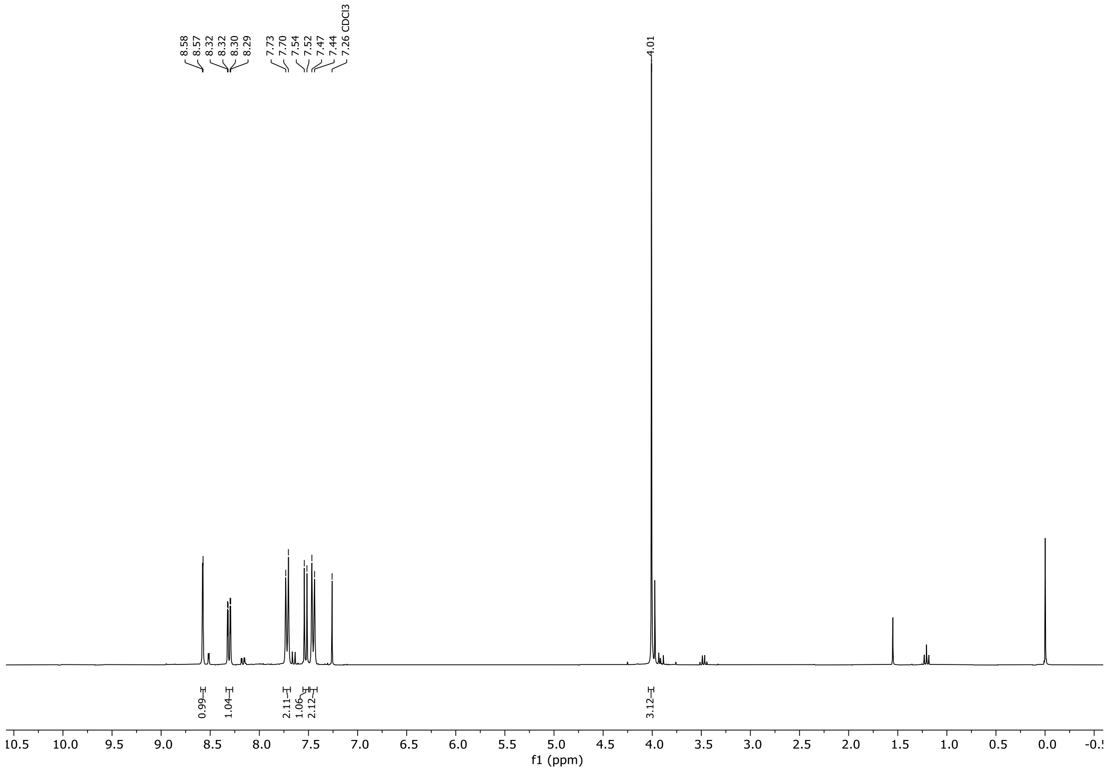

Figure S8: ^{1}H NMR spectrum (300 MHz) of compound **S1** in $CDCl_3$.

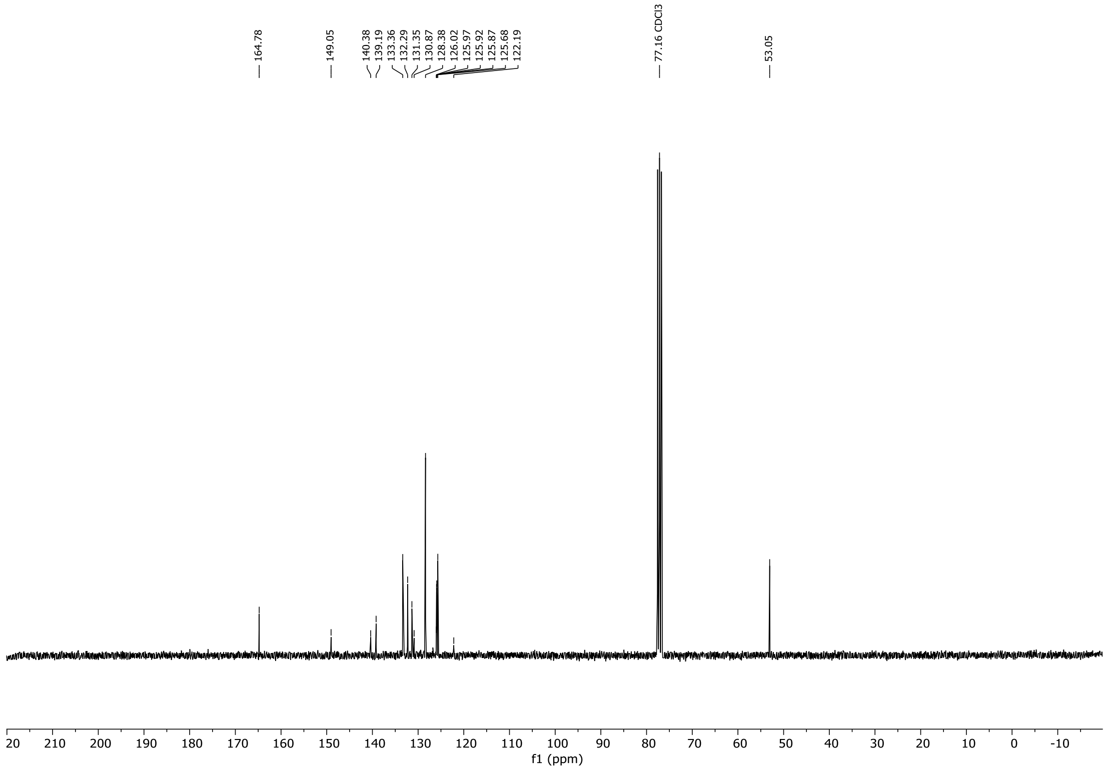

Figure S9: ^{13}C NMR spectrum (75 MHz) of compound **S1** in $CDCl_3$.

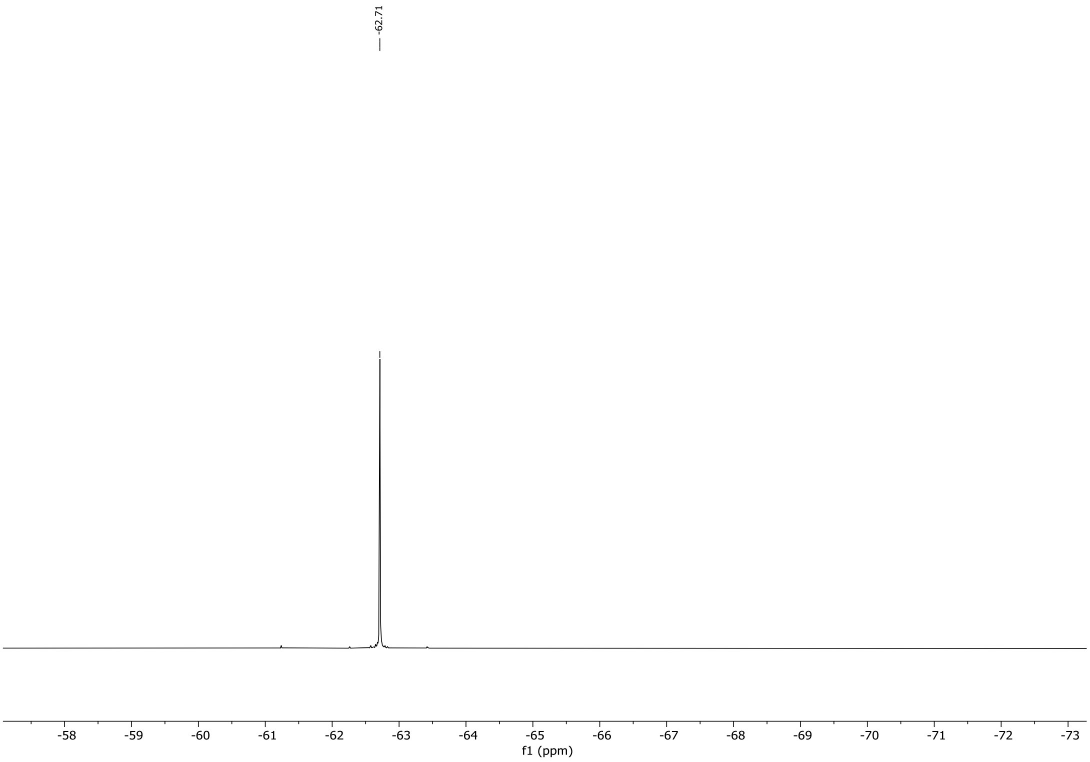

Figure S10: ^{19}F NMR spectrum (235 MHz) of compound **S1** in $CDCl_3$.

Synthesis of Methyl 2-amino-4'-(trifluoromethyl)-[1,1'-biphenyl]-4-carboxylate (S2)

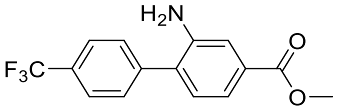

Chemical Formula: $C_{15}H_{12}F_3NO_2$
Molecular Weight: 295.26 $^g/_{mol}$

A 50 ml Schlenk flask was charged with 1.77 g (5.44 mmol, 1.00 eq) of compound **S1** and 2 mol% of 10% Pd/C in 20 ml of ethanol. The flask was evacuated 2 times to remove the oxygen and a balloon filled with hydrogen was inserted. Again, vacuum was applied to increase the hydrogen atmosphere, and the black mixture was stirred at r.t until full conversion (monitored by GC/MS, 1-2 d reaction time). After completion, the reaction mixture was filtered through a plug of celite and washed with DCM. The solvent was removed under reduced pressure and 1.5 g (94%) of an orange solid were obtained.

^{1}H-NMR (300 MHz, $CDCl_3$): δ = 7.76 – 7.69 (m, 2H), 7.62 – 7.57 (m, 2H), 7.52 – 7.44 (m, 2H), 7.16 (d, J = 7.9 Hz, 1H), 3.92 (s, 3H), 3.86 (bs, 2H) ppm.

^{13}C-NMR (75 MHz, $CDCl_3$): δ = 167.1, 143.7, 142.5, 131.0, 130.5, 130.6, 129.8, 129.4, 126.1, 122.4, 119.9, 116.8, 52.3 ppm.

^{19}F-NMR (235 MHz, $CDCl_3$): δ = -62.26 ppm.

ATR-IR [cm^{-1}]: 3483 (w), 3369 (m), 1704 (s), 1627 (m), 1612 (m), 1578 (m), 1559 (m), 1441 (s), 1429 (m), 1400 (m), 1319 (s), 1294 (s), 1262 (s), 1249 (s), 1156 (m), 1111 (vs), 1066 (s), 1018 (w), 989 (m), 845 (m), 825 (m), 798 (w), 768 (s), 745 (s), 715 (w), 614 (m), 551 (m), 472 (m).

EI-MS: m/z (+) = calc.: 295.08 [M]+, found: M+ 295.09 [M]+

CHNS [%] calc.: C: 61.02, H: 4.01, N: 6.01
found: C: 61.51, H: 3.85, N: 4.74

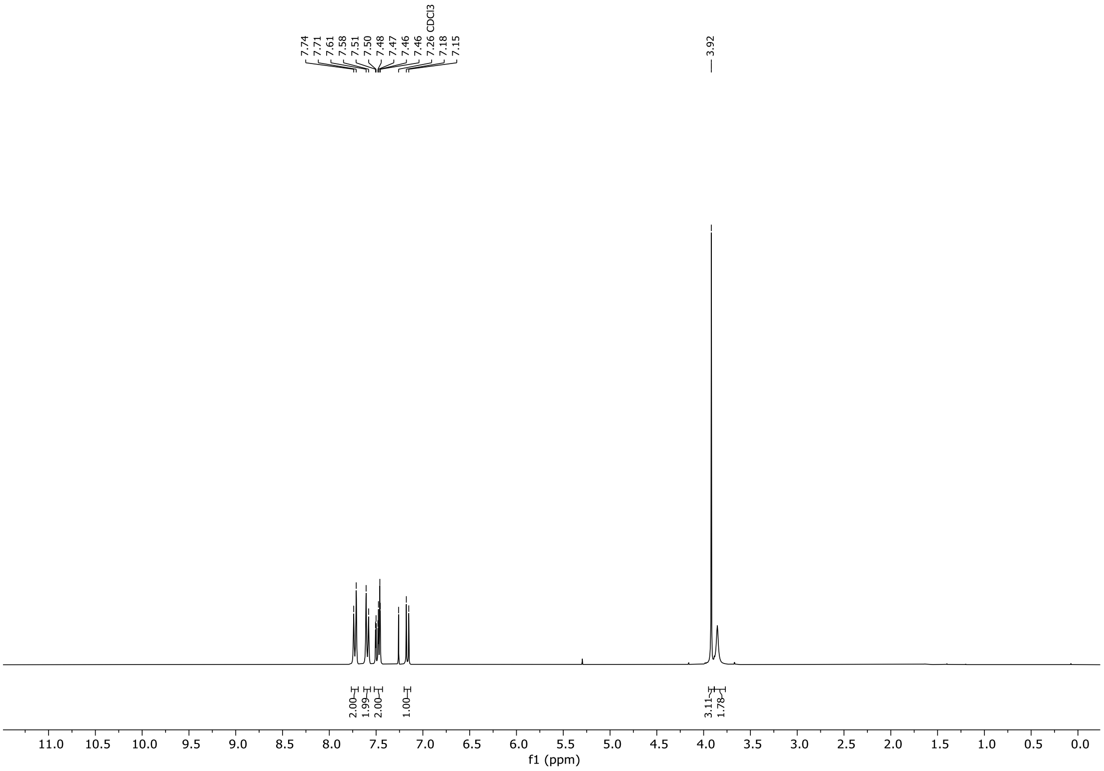

Figure S11: ^{1}H NMR spectrum (300 MHz) of compound **S2** in $CDCl_3$.

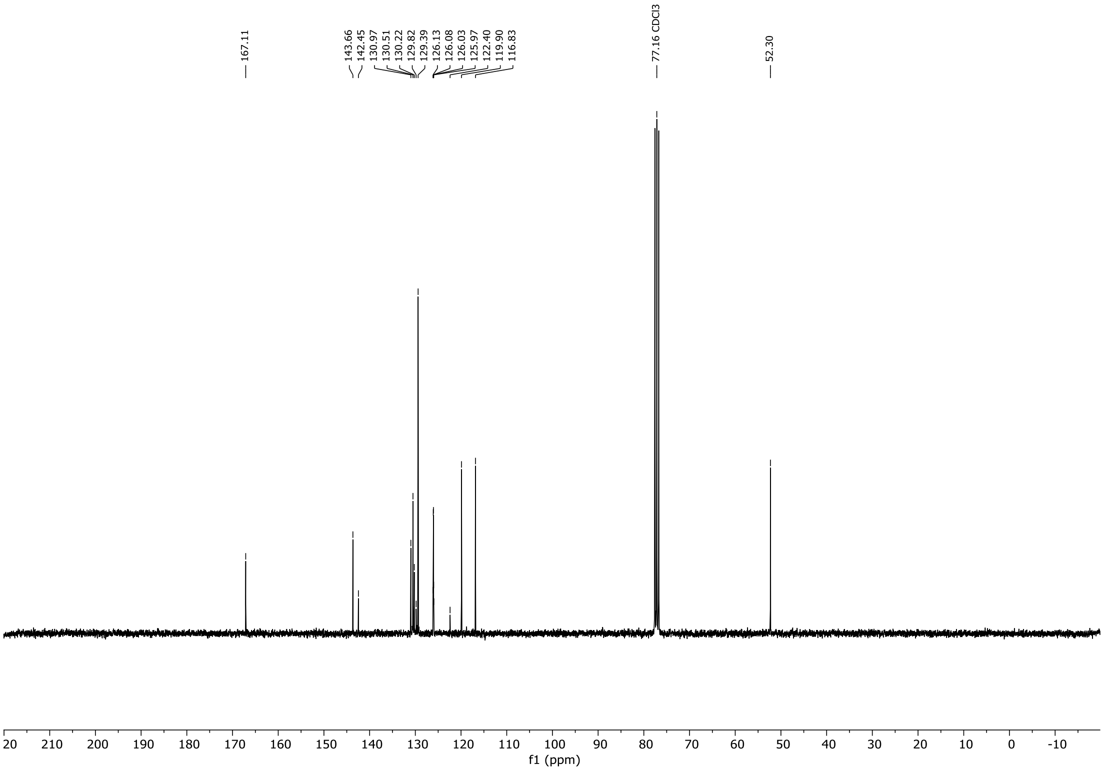

Figure S12: ^{13}C NMR spectrum (75 MHz) of compound **S2** in $CDCl_3$.

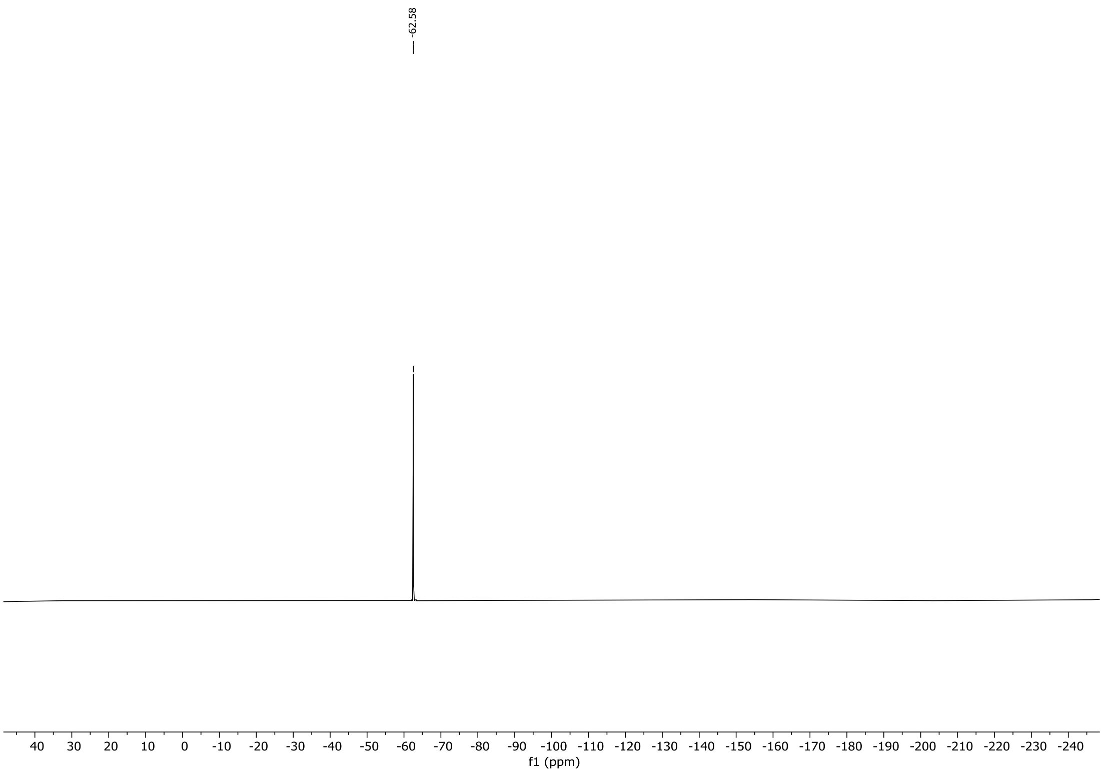

Figure S13: ^{19}F NMR spectrum (235 MHz) of compound **S2** in $CDCl_3$.

Synthesis of Methyl 2-iodo-4'-(trifluoromethyl)-[1,1'-biphenyl]-4-carboxylate (S3)

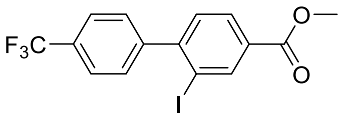

Chemical Formula: $C_{15}H_{10}F_3IO_2$
Molecular Weight: 406.14 $^{g}/_{mol}$

Compound **S2** (1.2 g, 4.1 mmol, 1 eq) was dissolved in 27 ml acetonitrile (0.15 M) and 2.32 g (12.2 mmol, 3 eq) of p-toluenesulfonic acid monohydrat were added at r.t forming a suspension. After stirring for 15 min, the reaction mixture was cooled to 0°C and 560 mg (8.13 mmol, 2 eq) of sodium nitrite, dissolved in small amounts of water, were slowly added. After complete addition, 1.69 g (10.16 mmol, 2.5 eq) of potassium iodide, dissolved in small amounts of water, were added dropwise to the obtained solution and stirred at r.t overnight. A saturated solution of $NaHCO_3$ (until pH = 9-10) and sodium sulfite were added and the aq. phase extracted three times with 50 ml diethyl ether. The combined org. phases were washed with brine and dried over Na_2SO_4. The solvent was removed in vacuo and the crude compound purified via column chromatography using silica gel and pentane:EtOAc (3:1) as eluent to obtain 1.25 g (76%) of an off-white solid.

^{1}H-NMR (300 MHz, $CDCl_3$): δ = 8.63 (d, J = 1.6 Hz, 1H), 8.07 (dd, J = 8.0, 1.7 Hz, 1H), 7.74 – 7.68 (m, 2H), 7.50 – 7.44 (m, 2H), 7.35 (d, J = 7.9 Hz, 1H), 3.96 (s, 3H) ppm.

^{13}C-NMR (75 MHz, $CDCl_3$): δ = 165.4, 149.6, 140.9, 131.2, 130.7, 130.2, 129.9, 129.6, 129.5, 125.3, 97.5, 52.7 ppm.

^{19}F-NMR (235 MHz, $CDCl_3$): δ = -62.59 ppm.

ATR-IR [cm^{-1}]: 3063 (vw), 2997 (vw), 2952 (w), 2845 (vw), 1709 (vs), 1615 (w), 1591 (w), 1547 (vw), 1433 (m), 1407 (vw), 1374 (m), 1332 (vs), 1284 (vs), 1240 (s), 1192 (w), 1159 (s), 1108 (vs), 1070 (s), 1049 (m), 1016 (m), 998 (m), 968 (w), 909 (vw), 861 (m), 837 (m), 766 (s), 745 (m), 715 (w), 679 (m), 652 (w), 491 (w).

EI-MS: m/z (+) = calc.: 406.0, found: 406.4 [M]+

CHNS [%] calc.: C: 44.36, H: 2.48
found: C: 42.34, H: 2.48

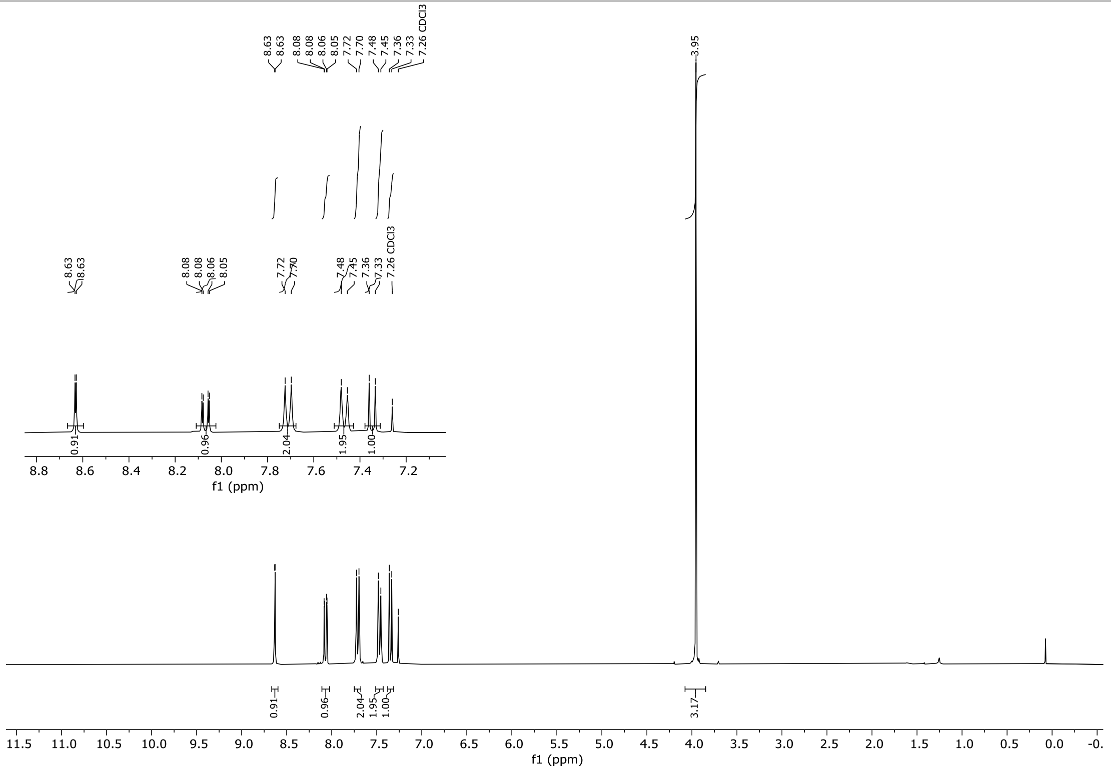

Figure S14: ^{1}H NMR spectrum (300 MHz) of compound **S3** in $CDCl_3$.

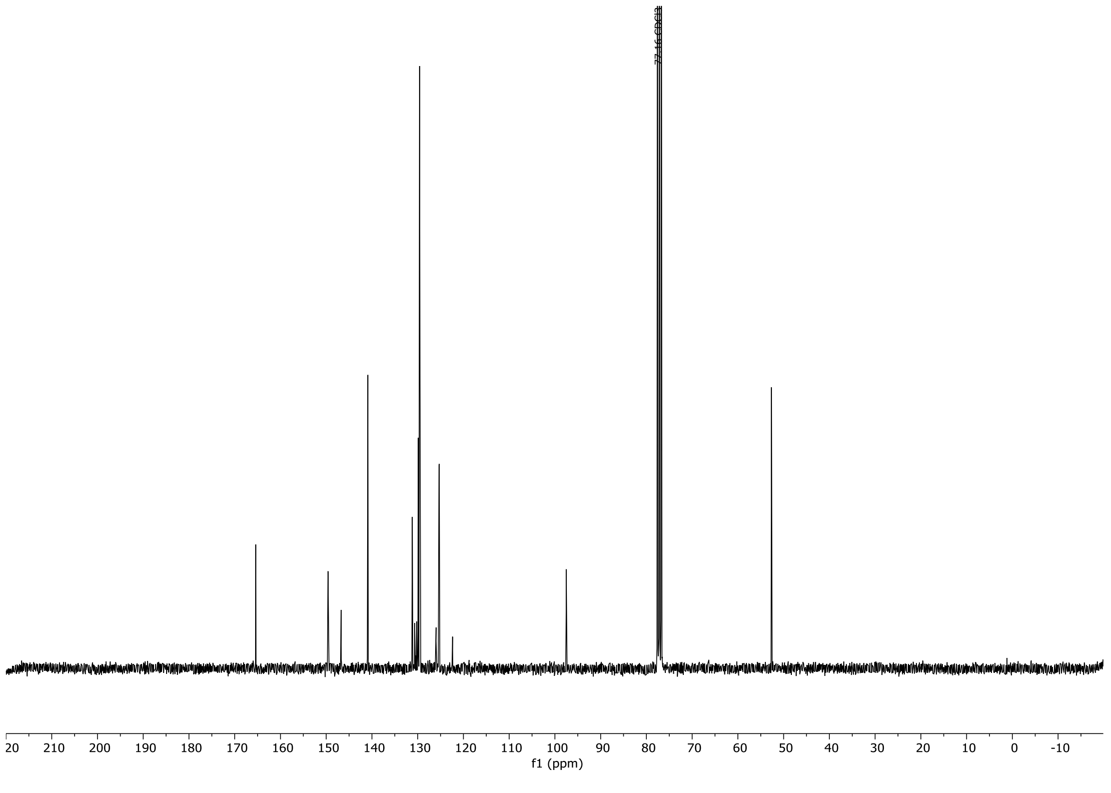

Figure S15: ^{13}C NMR spectrum (75 MHz) of compound **S3** in $CDCl_3$.

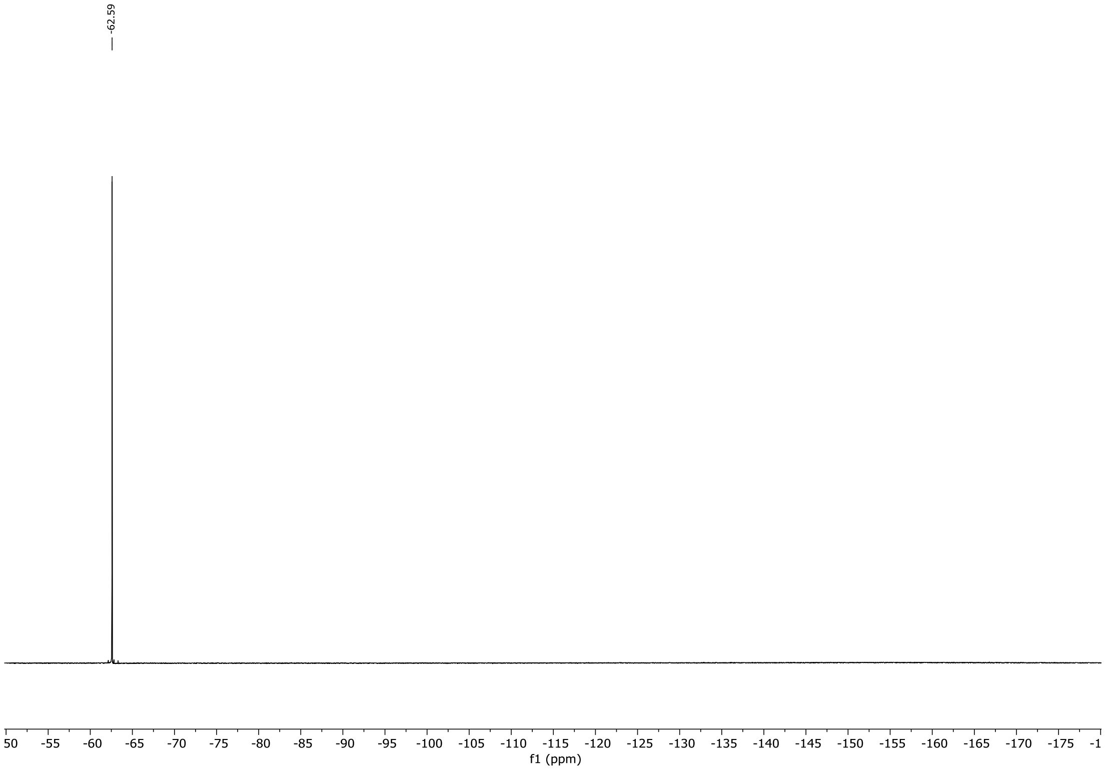

Figure S16: ^{19}F NMR spectrum (235 MHz) of compound **S3** in $CDCl_3$.

Synthesis of 2-Iodo-4'-(trifluoromethyl)-[1,1'-biphenyl]-4-carboxylic acid (S4)

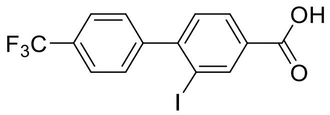

Chemical Formula: $C_{14}H_8F_3IO_2$
Molecular Weight: 392.12 $^{g}/_{mol}$

Compound **S3** (3.00 g, 7.4 mmol, 1.0 eq.) and sodium hydroxide (54.16 mg, 1.35mmol, 1.1 eq) were dissolved in 50 ml of ethanol and refluxed for 3 h. After removing the solvent, the residue was dissolved in 20 ml EtOAc and the org. phase washed three times with water and dried over Na_2SO_4. The solvent was removed under reduced pressure and 2.9 g (quant.) of a yellow solid were obtained.

^{1}H-NMR (300 MHz, $CDCl_3$): δ = 12.27 (s, 1H), 8.72 (d, J = 1.6 Hz, 1H), 8.16 (dd, J = 8.0, 1.7 Hz, 1H), 7.73 (d, J = 8.1 Hz, 2H), 7.49 (d, J = 8.0 Hz, 2H), 7.40 (d, J = 7.9 Hz, 1H) ppm.

^{13}C-NMR (75 MHz, $CDCl_3$): δ = 170.6, 150.6, 146.6, 141.5, 130.8, 130.4, 130.2, 130.1, 130.0, 129.6, 125.4, 97.7 ppm.

^{19}F-NMR (235 MHz, $CDCl_3$): δ = -62.61 ppm.

ATR-IR [cm^{-1}]: 3086-2811 (bs), 2645 (w), 2533 (w), 1688 (s), 1615 (w), 1589 (m), 1545 (m), 1420 (m), 1375 (w), 1323 (vs), 1301 (s), 1287 (s), 1246 (m), 1161 (m), 1120 (s), 1105 (vs), 1072 (s), 1050 (m), 1020 (m), 1002 (m), 918 (w), 856 (m), 833 (s), 767 (m), 745 (m), 675 (s), 604 (m), 567 (m), 523 (m).

ESI-MS: m/z (+) = calc. 456.0 [M+CH_3CN+Na]+, found 455.9 [M+CH_3CN+Na]+
m/z (-) = calc. 390.9 [M-H]-, found 391.0 [M-H]-

CHNS [%] calc.: C: 42.88, H: 2.06
found: C: 44.49, H: 2.11

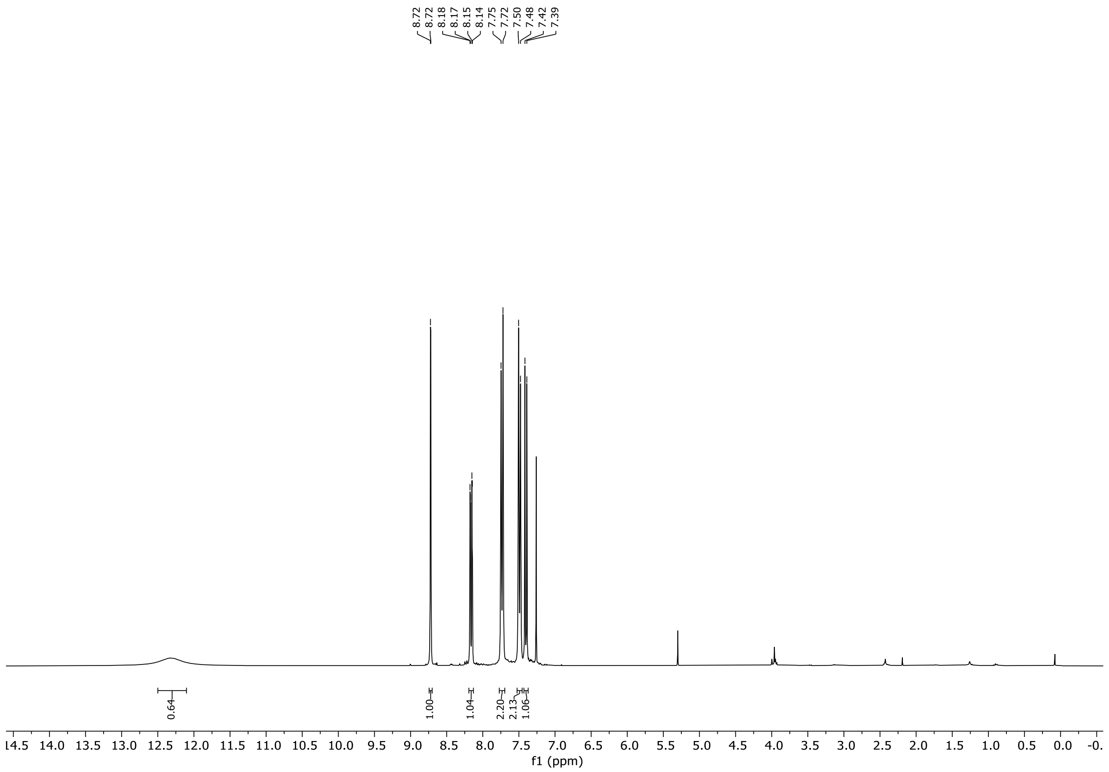

Figure S17: ^{1}H NMR spectrum (300 MHz) of compound **S4** in $CDCl_3$.

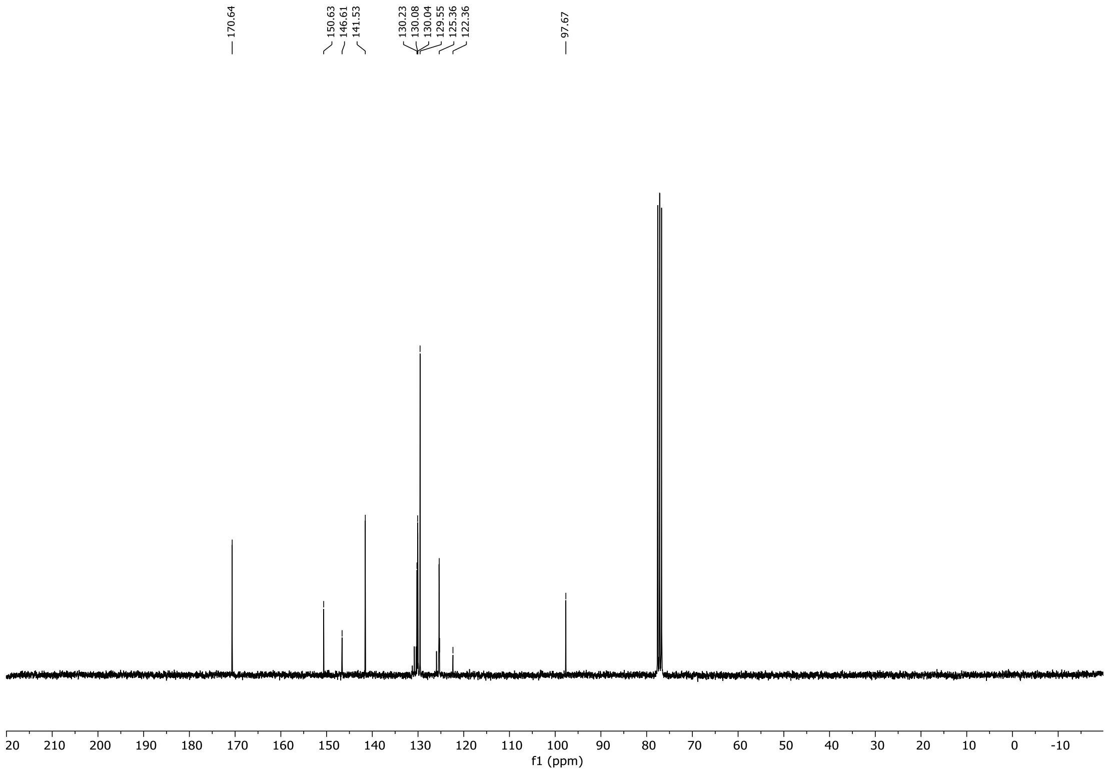

Figure S18: ^{13}C NMR spectrum (75 MHz) of compound **S4** in $CDCl_3$.

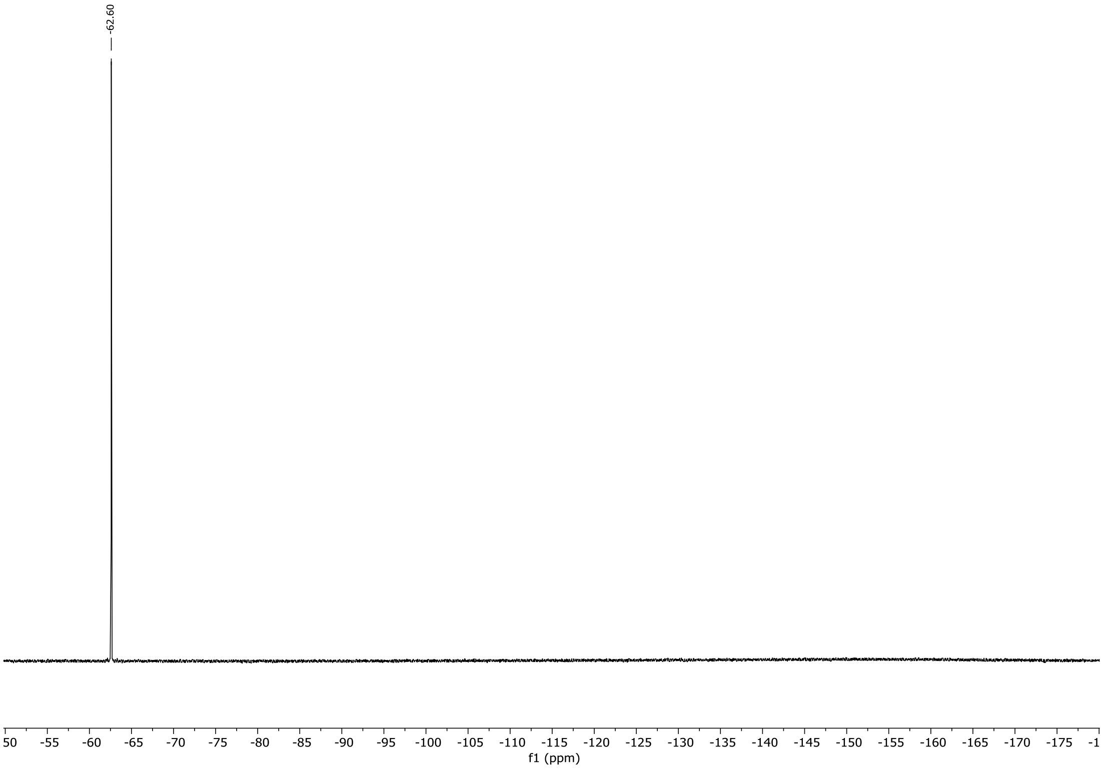

Figure S19: ^{19}F NMR spectrum (235 MHz) of compound **S4** in $CDCl_3$.

Synthesis of 3-Carboxy-7-(trifluoromethyl)dibenzo[b,d]iodol-5-ium-trifluoromethanesulfonate (12)

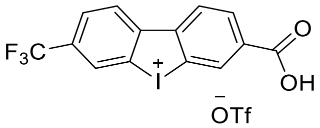

Chemical Formula: $C_{15}H_7F_6IO_5S$
Molecular Weight: 540,1709

Compound **S4** (200 mg, 0.510 mmol, 1.00 eq) was suspended in 1.00 ml DCM (0.5 M) and cooled to 0 °C. Afterwards, 171 mg (77% active oxidant, 0.765 mmol, 1.5 eq) of *meta*-chloroperoxybenzoic acid (*m*CPBA) were added and stirred for 30 min at this temperature. Triflic acid (HOTf) (135 µl, 1.53 mmol, 3 eq) was slowly added and the reaction mixture was warmed up to r.t and stirred overnight. The solvent was removed in vacuo and the resulting solid suspended in 10 ml diethyl ether and stirred for 15 min. The precipitate was filtered and the solid washed with small amounts of diethyl ether. Finally, 173 mg (63%) of a white solid were obtained.

^{1}H-NMR (300 MHz, DMSO-d_6): δ = 9.10 (s, 1H), 8.81 (d, J = 1.5 Hz, 1H), 8.76 (d, J = 8.3 Hz, 1H), 8.70 (d, J = 8.3 Hz, 1H), 8.56 (d, J = 1.7 Hz, 1H), 8.36 (dd, J = 8.2, 1.6 Hz, 1H), 8.27 (dd, J = 8.4, 1.7 Hz, 1H) ppm.[14]

^{19}F-NMR (235 MHz, DMSO-d_6): δ = -61.61, -77.71 ppm.

ATR-IR [cm^{-1}]: 3101-2882 (b), 1672 (m), 1421 (w), 1382 (w), 1317 (m), 1278 (m), 1261 (vs), 1242 (m), 1215 (vs), 1168 (s), 1134 (vs), 1076 (s), 1055 (w), 1024 (s), 912 (vw), 896 (w), 839 (m), 771 (w), 732 (w), 675 (m), 636 (m), 582 (w), 547 (w), 516 (m), 482 (w), 430 (vw).

FD-MS: m/z (+) = calc.: 390.9438 [M]+, found: 390.9440 [M]+

CHNS [%] calc.: C: 33.35, H: 1.31, S: 5.94
found: C: 32.96, H: 1.35, S: 7.37

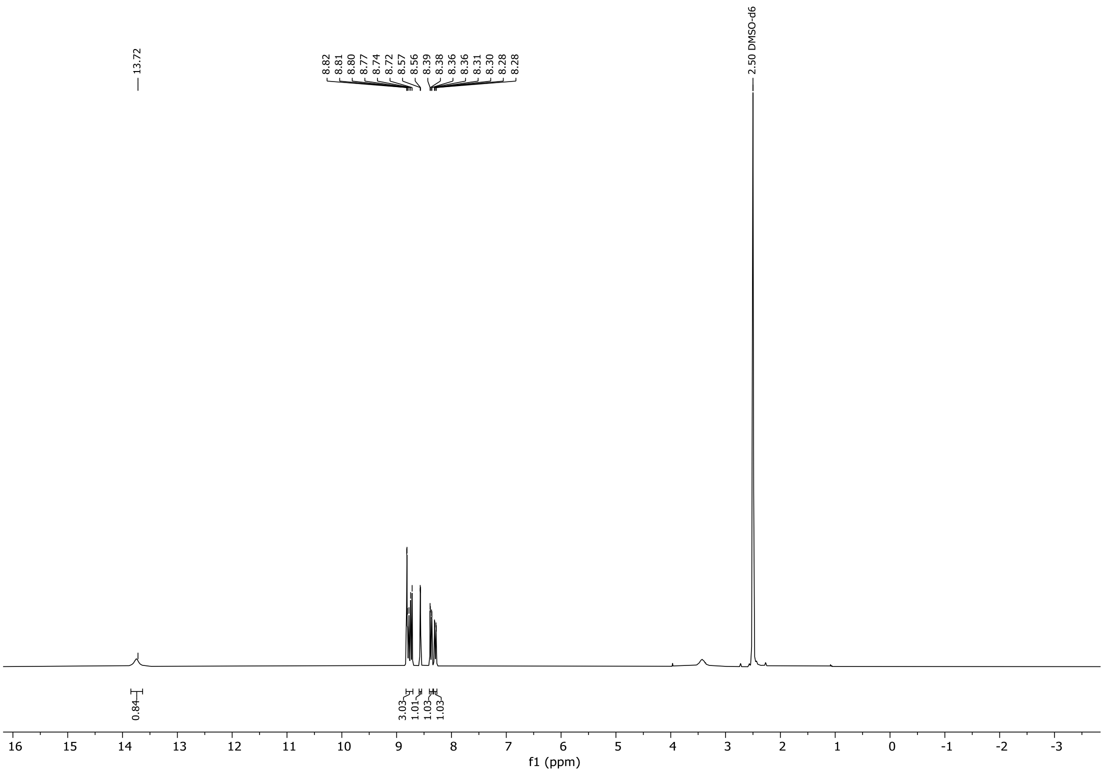

Figure S20: ^{1}H NMR spectrum (300 MHz) of compound **12** in DMSO-d_6.

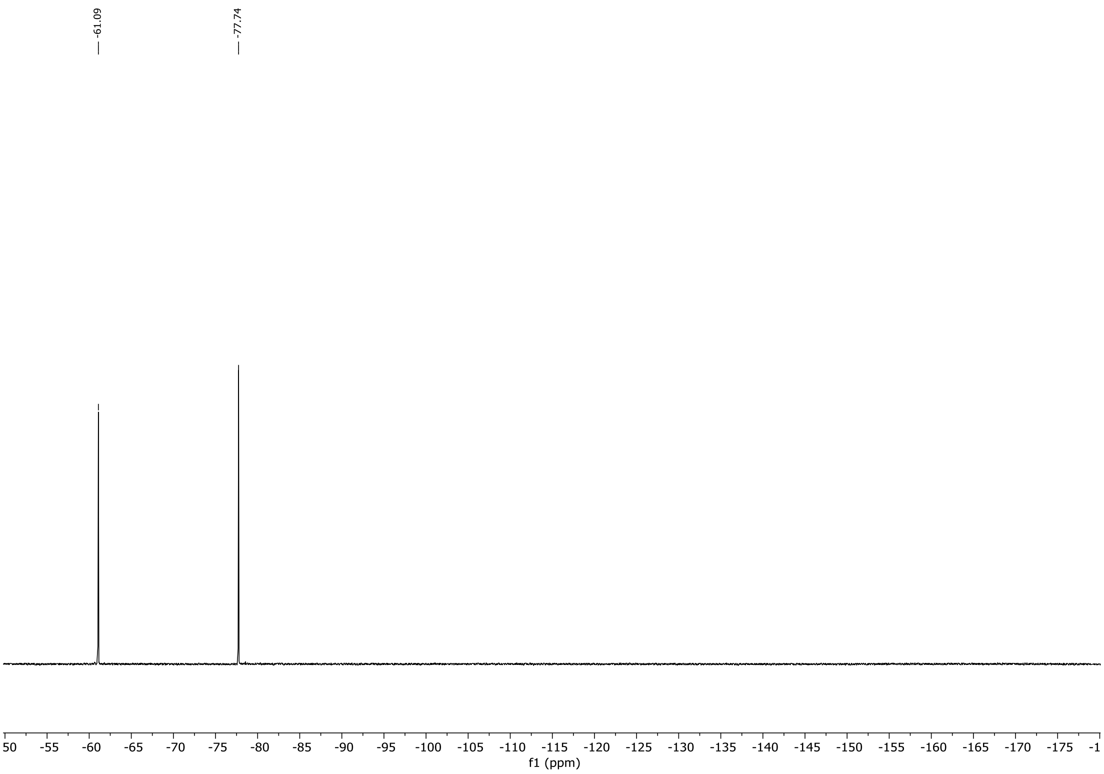

Figure S21: ^{19}F NMR spectrum (235 MHz) of compound **12** in DMSO-d_6.

Synthesis of Methyl 4'-(tert-butyl)-2-nitro-[1,1'-biphenyl]-4-carboxylate(S5)

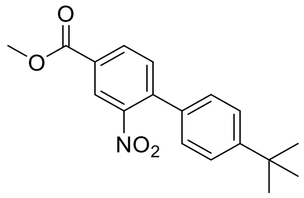

Chemical Formula: $C_{18}H_{19}NO_4$
Molecular Weight: 313,3530

Methyl 4-chloro-3-nitrobenzoate (328 mg, 1.52 mmol, 1 eq), 4-tert-butylphenylboronic acid (405 mg, 2.28 mmol, 1.5 eq), diisopropyl amine (308 mg, 427 µl, 3.04 mmol, 2 eq), and 12 mg (0.05 mmol, 0.035 eq) of palladium(II)acetate were suspended in 5 ml water (0.5 M) and refluxed for 16 h. After cooling down to r.t., the aq. phase was extracted with EtOAc and the org. phase first washed with water and brine and then dried over Na_2SO_4. After column chromatography (silica gel) using pentane/diethyl ether (2:1), 460 mg (97%) of an off-white solid were obtained.

^{1}H-NMR (300 MHz, $CDCl_3$): δ = 8.38 (d, J = 1.6 Hz, 1H), 8.20 – 8.14 (m, 1H), 7.47 (d, J = 8.0 Hz, 1H), 7.39 (d, J = 8.2 Hz, 2H), 7.20 (d, J = 8.5 Hz, 2H), 3.91 (s, 3H) ppm.

^{13}C-NMR (75 MHz, $CDCl_3$): δ = 165.1, 152.3, 140.4, 133.4, 132.8, 132.3, 130.2, 127.6, 126.0, 125.3, 52.9, 34.9, 31.4 ppm.

ATR-IR [cm-1]: 2956 (w), 1730 (s), 1720 (s), 1614 (w),1531 (s),1487 (vw), 1462 (vw), 1435 (m), 1392 (w), 1355 (m), 1282 (s), 1234 (s), 1192 (w), 1151 (m), 1128 (m), 1112 (m), 1087 (w), 1004 (w), 970 (w), 921 (w), 887 (w), 862 (w), 831 (m), 819 (m), 765 (s), 746 (s), 721 (m), 702 (w), 663 (w), 582 (m), 565 (w), 540 (w), 499 (w), 453 (w).

FD-MS: m/z (+) = calc.: 313.1314 [M]+, found: 313.1370 [M]+

CHNS [%] calc.: C: 69.0, H: 6.1, N: 4.47
found: C: 72.3, H: 6.47, N: 4.75

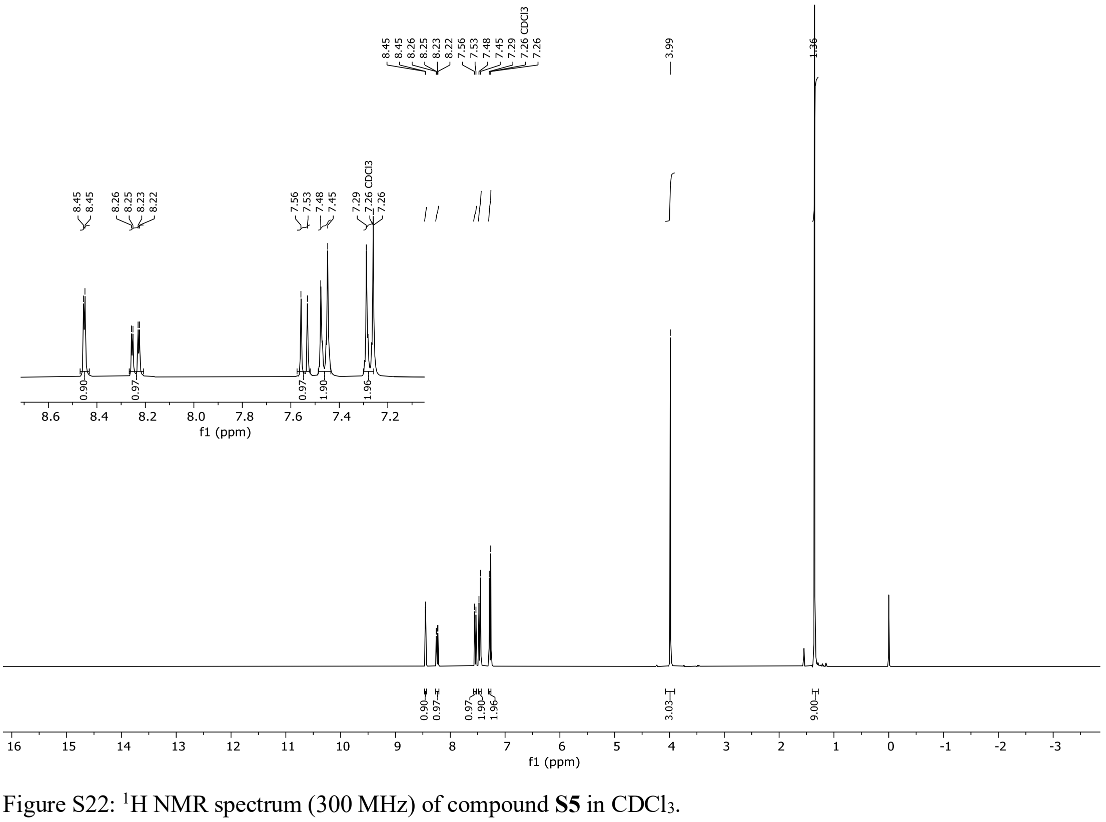

Figure S22: ^{1}H NMR spectrum (300 MHz) of compound **S5** in $CDCl_3$.

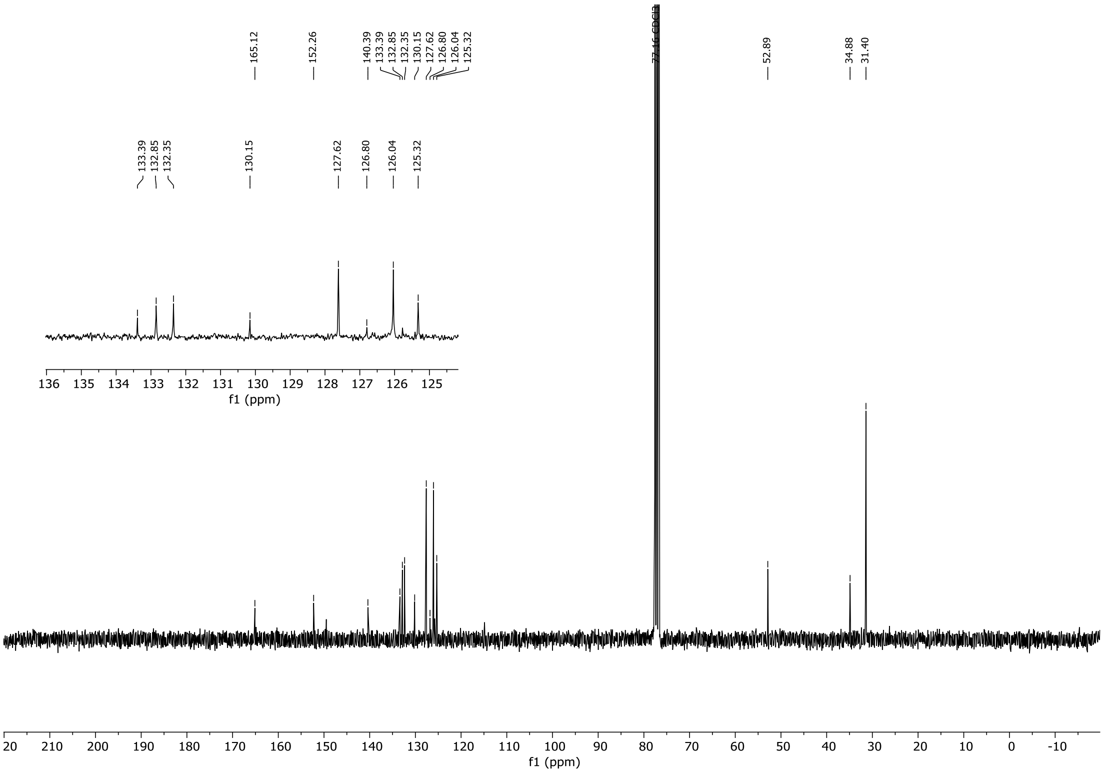

Figure S23: ^{13}C NMR spectrum (75 MHz) of compound **S5** in $CDCl_3$.

Synthesis of Methyl 2-amino-4'-(tert-butyl)-[1,1'-biphenyl]-4-carboxylate (S6)

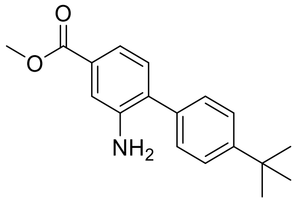

Chemical Formula: $C_{18}H_{21}NO_2$
Molecular Weight: 283,3710

A 50 ml Schlenk flask was charged with 580 mg (1.85 mmol, 1.00 eq) of compound **S5** and 2 mol% of 10% Pd/C in 4 ml of ethanol. The flask was first evacuated 2 times before a balloon filled with hydrogen was inserted. Again, vacuum was applied to increase the hydrogen atmosphere, and the black mixture was stirred at r.t until full conversion (monitored by GC/MS, 1-2 h reaction time). After completion, the reaction mixture was filtered through a plug of celite and washed with DCM. The solvent was removed under reduced pressure and 480 mg (92%) of a white solid were obtained.

^{1}H-NMR (300 MHz, $CDCl_3$): δ = 7.51 – 7.37 (m, 6H), 7.18 (d, J = 7.8 Hz, 1H), 3.90 (s, 3H), 1.37 (s, 1H) ppm.

^{13}C-NMR (75 MHz, $CDCl_3$): δ = 167.1, 150.8, 143.7, 135.5, 131.9, 130.5, 129.9, 128.4, 125.9, 119.7, 116.3, 52.1, 34.7, 31.4 ppm.

ATR-IR [cm^{-1}]: 3447 (vw), 3367 (vw), 2952 (vw), 1712 (s), 1608 (m), 1571 (w), 1519 (w), 1495 (w), 1440 (m), 1425 (w), 1397 (m), 1361 (vw), 1309 (s), 1254 (m), 1224 (s),1138 (w), 1108 (s), 1004 (w), 980 (w), 885 (w), 830 (m), 797 (w), 769 (s), 751 (s), 586 (m).

ESI-MS: m/z (+) = calc. 284.2 [M+H]+, found 284.1 [M+H]+

CHNS [%] calc.: C: 76.30, H: 7.47, N: 4.94
found: C: 77.89, H: 7.48, N: 7.06

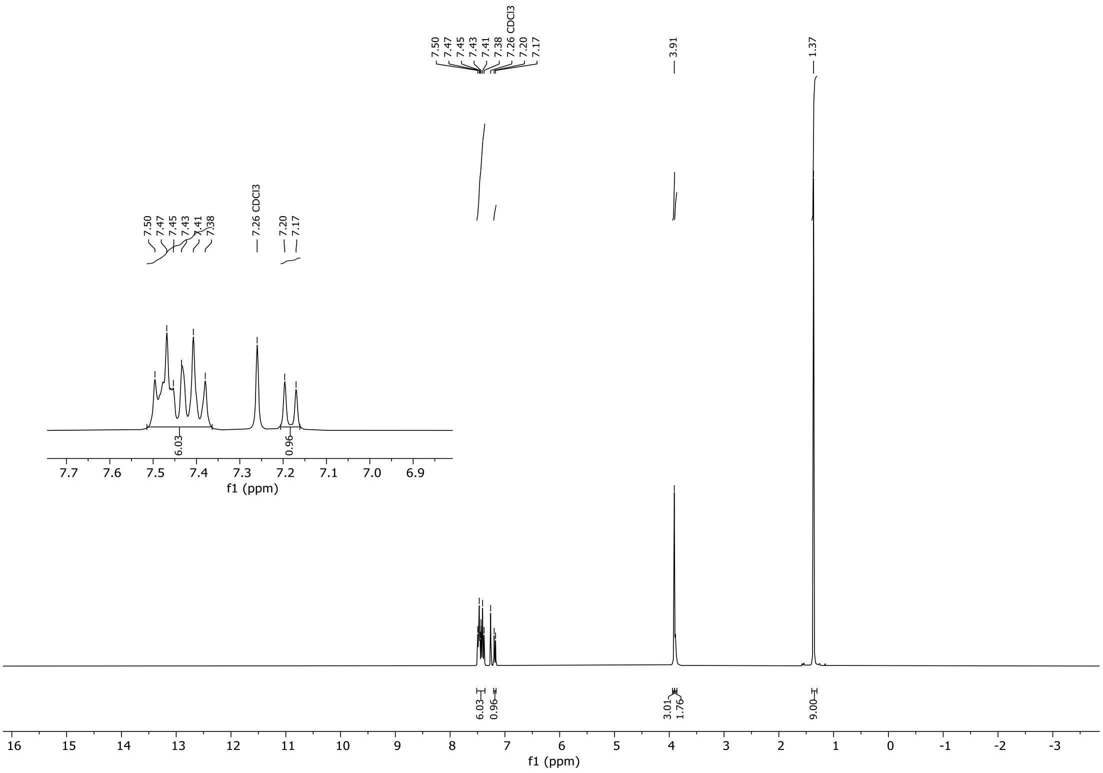

Figure S24: ^{1}H NMR spectrum (300 MHz) of compound **S6** in $CDCl_3$.

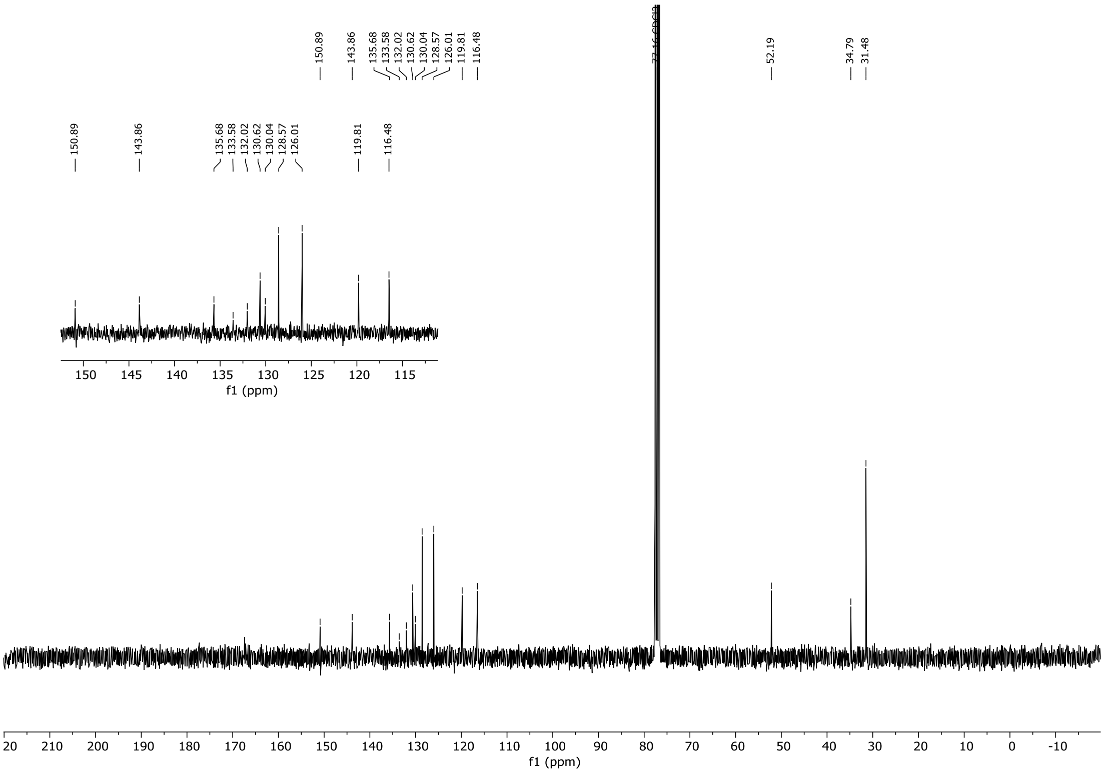

Figure S25: ^{13}C NMR spectrum (75 MHz) of compound **S6** in $CDCl_3$.

Synthesis of Methyl 4'-(tert-butyl)-2-iodo-[1,1'-biphenyl]-4-carboxylate (S7)

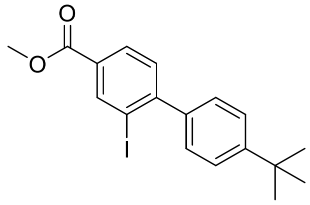

Chemical Formula: $C_{18}H_{19}IO_2$
Molecular Weight: 394,2525

Compound **S6** (480 mg, 1.69 mmol, 1 eq) was dissolved in acetonitrile (0.15 M) and 1 g (5.07 mmol, 3 eq) of p-toluenesulfonic acid monohydrate were added at room temperature, forming a white precipitation. The suspension was cooled to 0 °C and aqueous solution of sodium nitrite (233 mg, 3.38 mmol, 2 eq dissolved in small amounts of water) followed by aq. solution of potassium iodide (96 mg, 4.23 mmol, 2.5 eq) were added. The mixture was allowed to warm up to room temperature and stirred overnight. Afterwards, saturated aq. solution of $NaHCO_3$ (until pH = 9-10) and sodium sulfite were added and the aq. phase extracted with ethyl acetate (3x). The combined org. phases were dried over Na_2SO_4 and the solvent removed in vacuum. Finally, 640 mg (96%) of a yellow solid were obtained.

^{1}H-NMR (300 MHz, $CDCl_3$): δ = 8.61 (d, J = 1.6 Hz, 1H), 8.03 (dd, J = 7.9, 1.7 Hz, 1H), 7.46 (d, J = 8.1 Hz, 2H), 7.36 (d, J = 8.0 Hz, 1H), 7.30 (d, J = 8.2 Hz, 2H), 3.94 (s, 3H) ppm.

^{13}C-NMR (75 MHz, $CDCl_3$): δ = 165.5, 150.9, 140.7, 140.2, 130.2, 130.0, 129.2, 128.6, 125.0, 98.1, 52.4, 34.7, 31.4 ppm.

ATR-IR [cm^{-1}]: 2956 (b), 2903-2866 (wb), 1716 (vs), 1593 (w), 1462 (w), 1436 (m), 1396 (w), 1373 (w), 1365 (w), 1359 (w), 1274 (vs), 1240 (vs), 1195 (vs), 1109 (vs), 1055 (m), 1018 (w), 1001 (m), 968 (w), 898 (w), 852 (vw), 840 (m), 831 (s), 769 (s), 761 (vs), 746 (m), 717 (w), 707 (w), 653 (w), 584 (s), 547 (vw), 513 (vw), 441(m).

EI-MS: m/z = calc. 395.05 [M]+, found: 395.09 [M]+

CHNS [%] calc.: C: 54.84, H: 4.86
found: C:54.02, H: 4.51

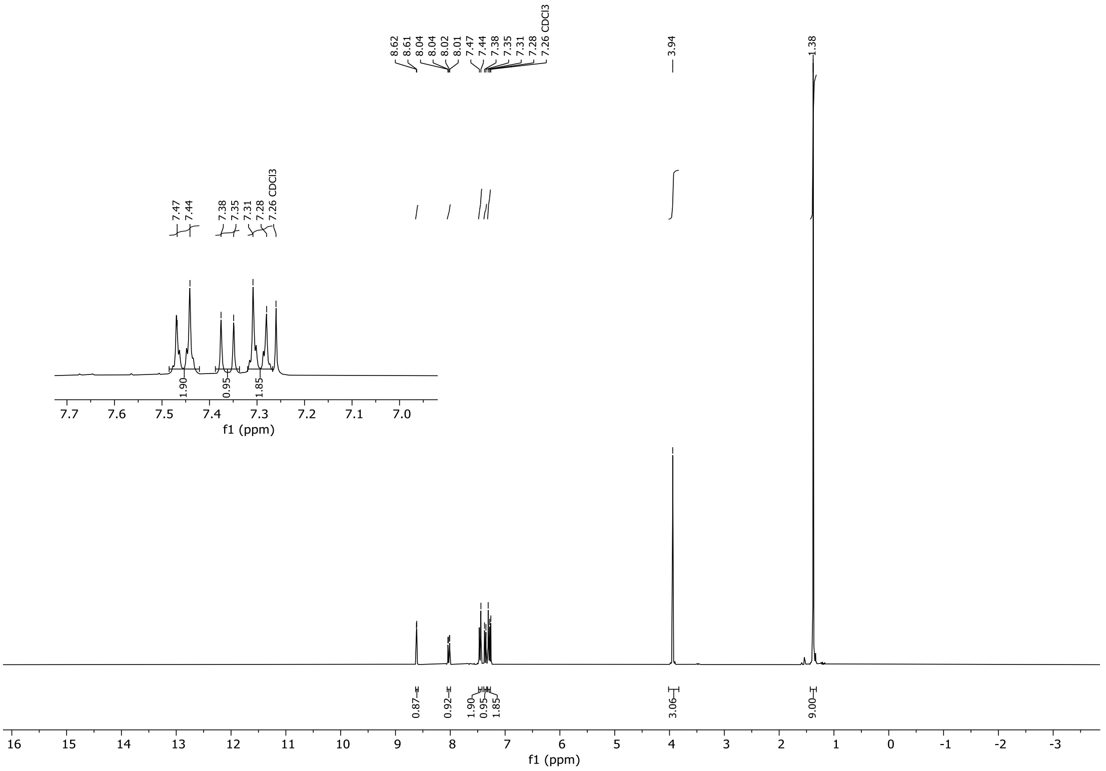

Figure S26: ^{1}H NMR spectrum (300 MHz) of compound **S7** in $CDCl_3$.

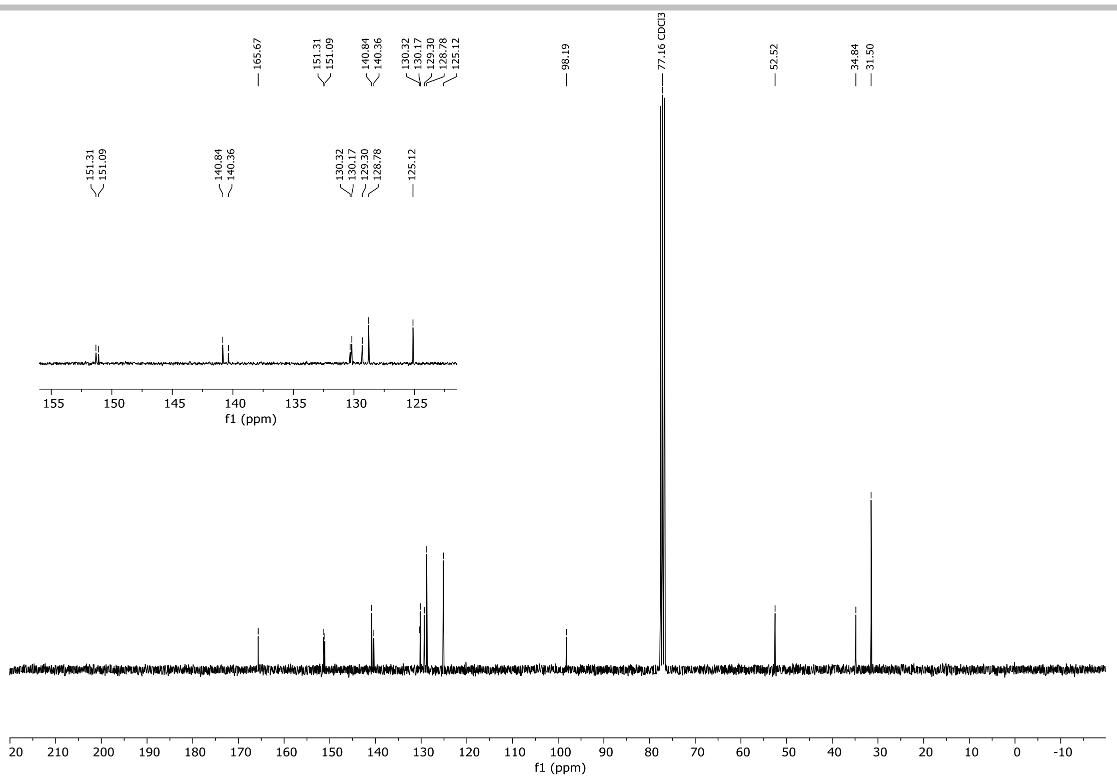

Figure S27: ^{13}C NMR spectrum (75 MHz) of compound **S7** in $CDCl_3$.

Synthesis of 4'-(tert-butyl)-2-iodo-[1,1'-biphenyl]-4-carboxylic acid (S8)

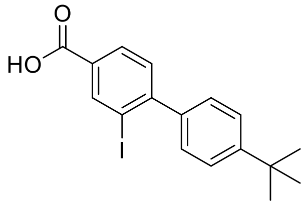

Chemical Formula: $C_{17}H_{17}IO_2$
Molecular Weight: 380,2255

Compound **S7** (260 mg, 0.659 mmol) was dissolved in 10 ml of ethanol, sodium hydroxide (29 mg, 724.9 mmol, 1.10 eq) added, and the mixture refluxed for 3 h. After removing the solvent, the residue was dissolved in 10 ml EtOAc and the org. phase washed three times with water and dried over Na_2SO_4. The solvent was removed under reduced pressure and 230 mg (92%) of an off-white crystalline solid were obtained.

^{1}H-NMR (300 MHz, $CDCl_3$): δ = 11.72 (s, 1H), 8.69 (d, J = 1.7 Hz, 1H), 8.11 (dd, J = 7.9, 1.7 Hz, 1H), 7.52 – 7.38 (m, 3H), 7.35 – 7.29 (m, 2H), 1.38 (s, 9H).

^{13}C-NMR (75 MHz, $CDCl_3$): δ = 170.2, 152.0, 151.5, 141.5, 140.3, 130.3, 129.9, 129.3, 128.8, 125.2, 98.3, 34.9, 31.5 ppm.

ATR-IR [cm^{-1}]: 1681 (s), 1591 (w), 1543 (w), 1460 (vw), 1413 (m), 1375 (w), 1361 (w), 1282 (s), 1240 (m), 1197 (w), 1122 (w), 1110 (w), 1055 (w), 1024 (w), 1002 (w), 970 (w), 952 (w), 912 (w), 848 (w), 831 (s), 771 (m), 752 (m), 744 (m), 694 (m), 655 (w), 582 (m), 549 (m), 540 (m), 472 (w), 441 (w).

ESI-MS: m/z (-) = calc. 379.02 [M-H]-, found 379.2 [M]-

CHNS [%] calc.: C: 53.70, H: 4.51
found: C:54.02, H: 4.51

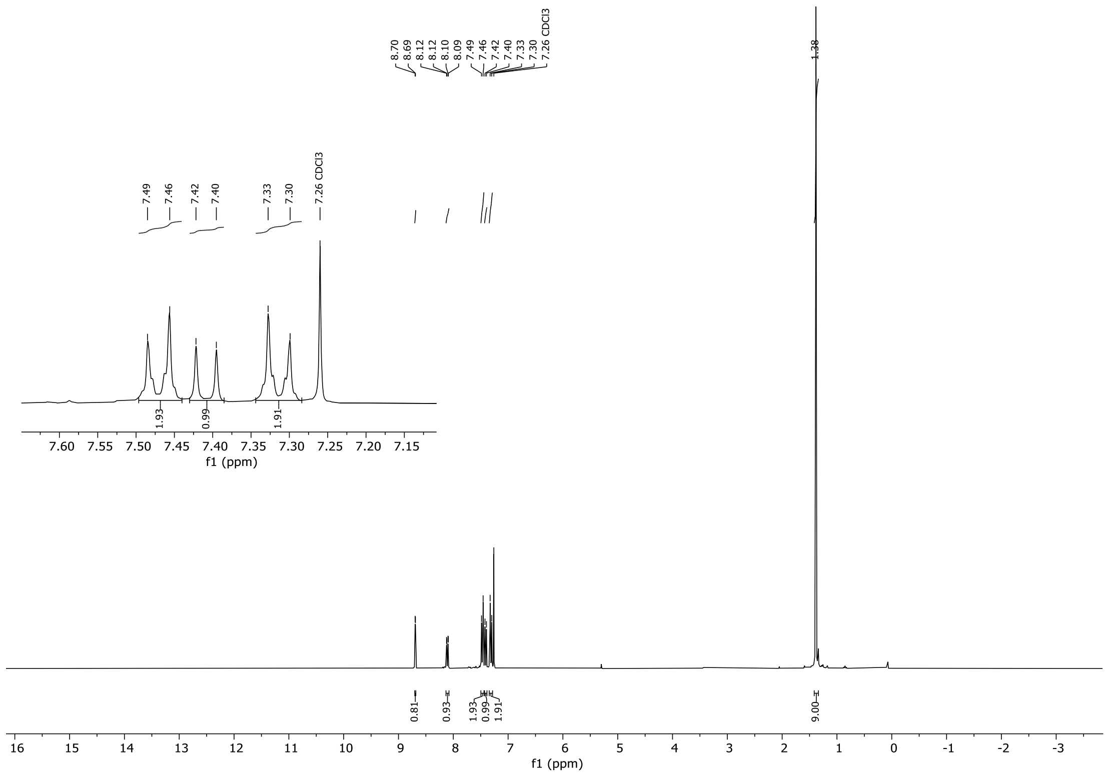

Figure S28: ^{1}H NMR spectrum (300 MHz) of compound **S8** in $CDCl_3$.

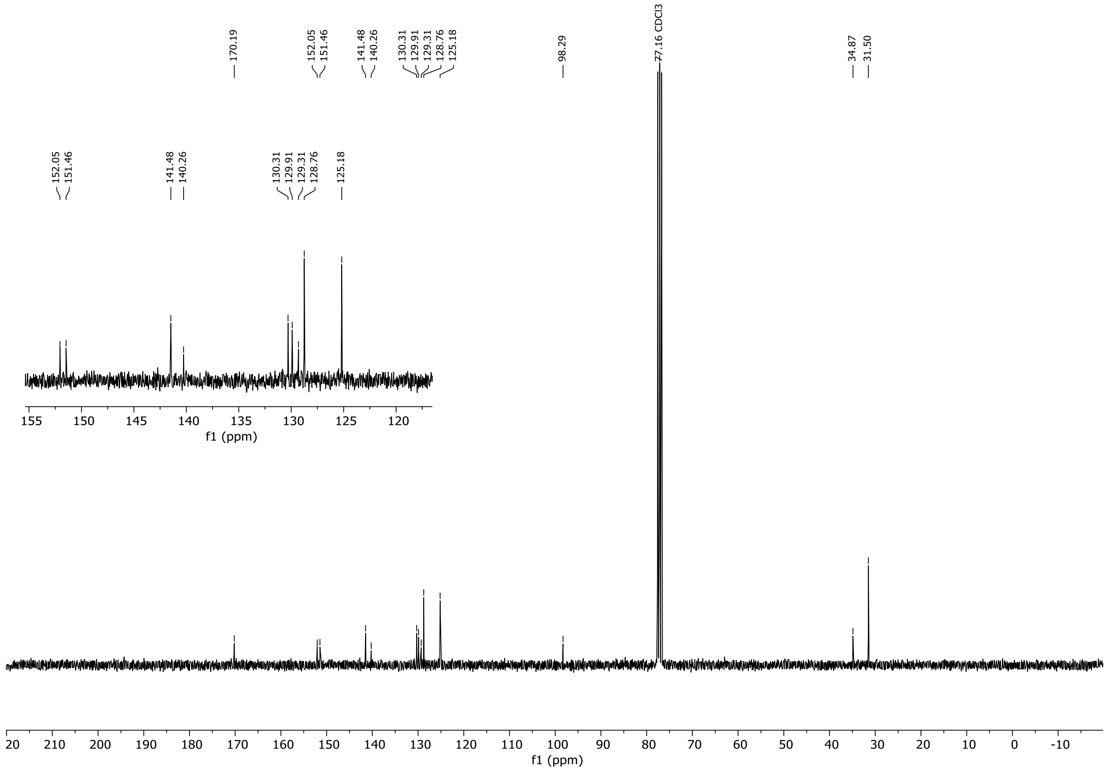

Figure S29: ^{13}C NMR spectrum (75 MHz) of compound **S8** in $CDCl_3$.

Synthesis of 3-(tert-butyl)-7-carboxydibenzo[b,d]iodol-5-ium trifluoromethanesulfonate (13)

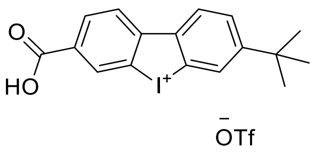

Chemical Formula: $C_{18}H_{16}F_3IO_5S$
Molecular Weight: 528,2807

Compound **S8** (130 mg, 0.342 mmol) was suspended in 0.68 ml DCM (0.5 M) and cooled to 0 °C. Afterwards, 115 mg (77% active oxidant, 0.513 mmol, 1.5 eq) of *meta*-chloroperoxybenzoic acid (*m*CPBA) were added and stirred for 30 min at this temperature. Triflic acid (HOTf) (30 µl, 0.342 mmol, 3 eq) was slowly added and the reaction mixture was warmed up to r.t and stirred overnight. The solvent was removed in vacuo and the resulting solid suspended in 10 ml diethyl ether and stirred for 15 min. The precipitate was filtered and the solid washed with small amounts of diethyl ether. Finally, 130 mg (72%) of a white solid were obtained.

^{1}H-NMR (300 MHz, DMSO-d_6, 300 MHz): δ = 13.61 (s, 1H), 8.76 (s, 1H), 8.64 – 8.40 (m, 2H), 8.38 – 8.14 (m, 2H), 7.95 (d, *J* = 7.5 Hz, 1H), 1.39 (s, 9H).) ppm.

^{13}C-NMR (75 MHz, DMSO-d_6, 75 MHz): δ = 165.6, 155.5, 145.3, 138.1, 132.2, 131.6, 131.2, 128.5, 127.4, 126.8, 126.6, 123.2, 121.6, 35.6, 30.8 ppm.

^{19}F-NMR (235 MHz, DMSO-d_6, 235 MHz): δ = -77.73 ppm.

ATR-IR [cm-1]: 1722 (s), 1593 (w), 1388 (w), 1251 (s), 1224 (s), 1215 (s), 1168 (s), 1109 (w), 1026 (vs), 985 (vw), 852 (w), 829 (m), 796 (m), 752 (w), 729 (w), 684 (m), 677 (m), 632 (vs), 603 (w), 576 (w), 543 (w), 513 (m), 432 (w).

FD-MS: m/z (+) = calc.: 379.0190 [M]+, found: 379.0190 [M]+

CHNS [%] calc.: C: 40.92, H: 3.05, N: 6.07
found: C: 38.97, H: 2.79, N: 6.32

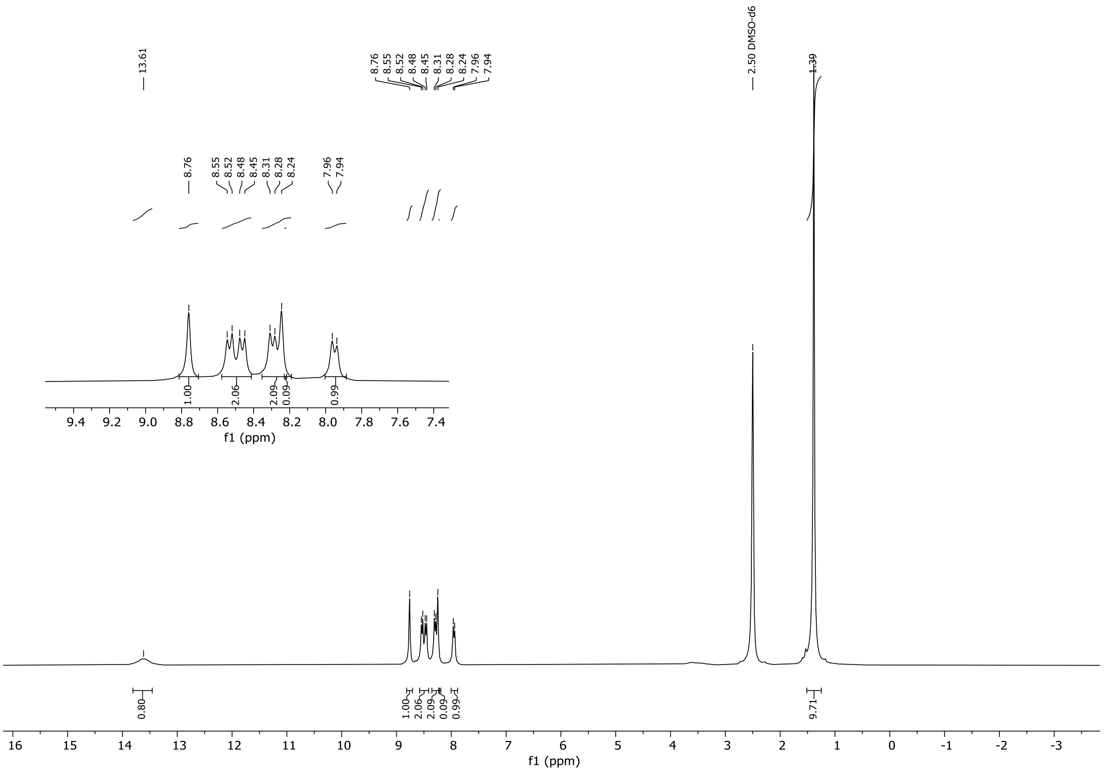

Figure S30: ^{1}H NMR spectrum (300 MHz) of compound **13** in DMSO-d_6.

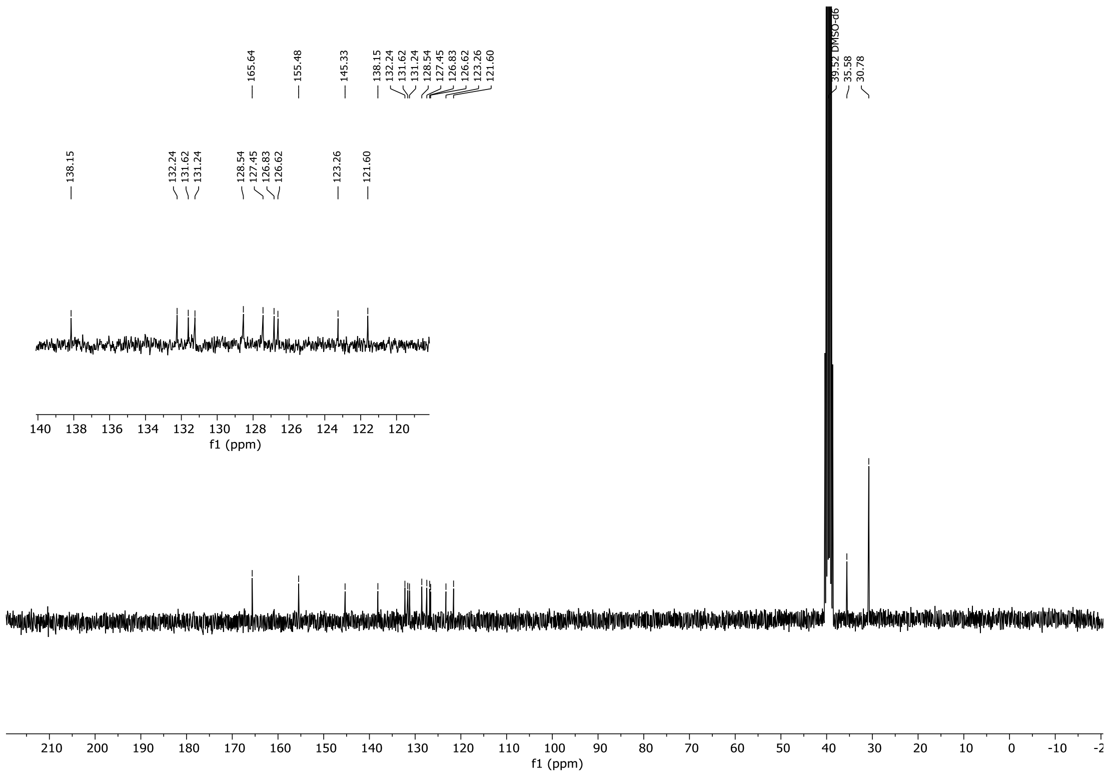

Figure S31: ^{13}C NMR spectrum (75 MHz) of compound **13** in DMSO-d_6.

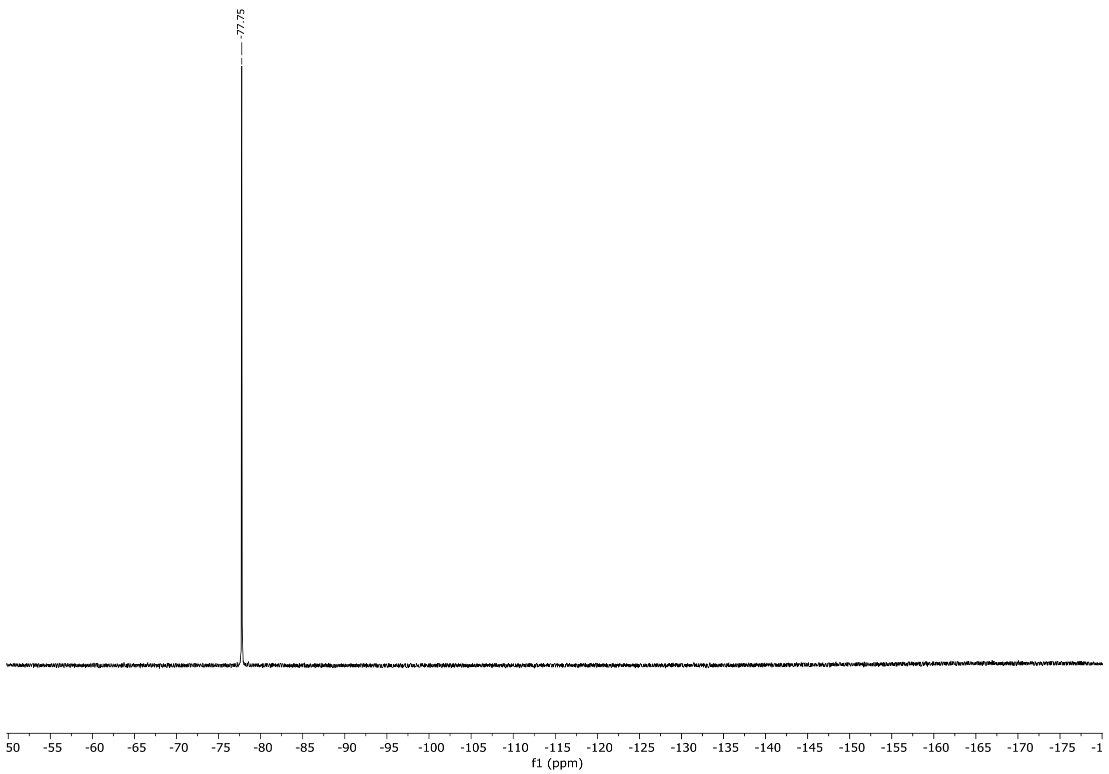

Figure S32: ^{19}F NMR (235 MHz) of compound **13** in DMSO-d_6.

Synthesis of 4-bromo-3-nitrobenzenesulfonyl chloride (16)

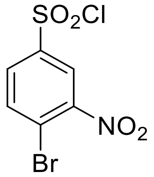

Chemical Formula: $C_6H_3BrClNO_4S$
Molecular Weight: 300,5070

In a round bottom flask, 4-bromobenzenesulfonyl chloride (15 g, 58.7 mmol, 1.0 eq.) was dissolved in conc. H_2SO_4 (250 ml, 4.57 mol, 77.9 eq.) and cooled to 0 °C with an ice bath. To that solution, conc. HNO_3 (60 ml, 800 mmol, 13.6 eq.) was added dropwise. After the addition, the reaction mixture was stirred at ambient temperature overnight. Then, the reaction was poured onto ice, and the aqueous phase was extracted with EtOAc. The combined organic phases were dried over $MgSO_4$, and the solvent was removed. The product was isolated as pale yellow solid (17.45 g, 99%).

^{1}H NMR (300 MHz, $CDCl_3$): δ = 8.50 (t, *J* = 1.3 Hz, 1H), 8.10 (s, 1H), 8.09 (s, 1H) ppm.

^{13}C NMR (75 MHz, $CDCl_3$): δ = 150.0, 144.1, 137.2, 130.6, 124.3, 123.0 ppm.

ESI-MS: m/z (-) = calc. 148.4 [M-2H]2-, found 148.9 [M-2H]2-

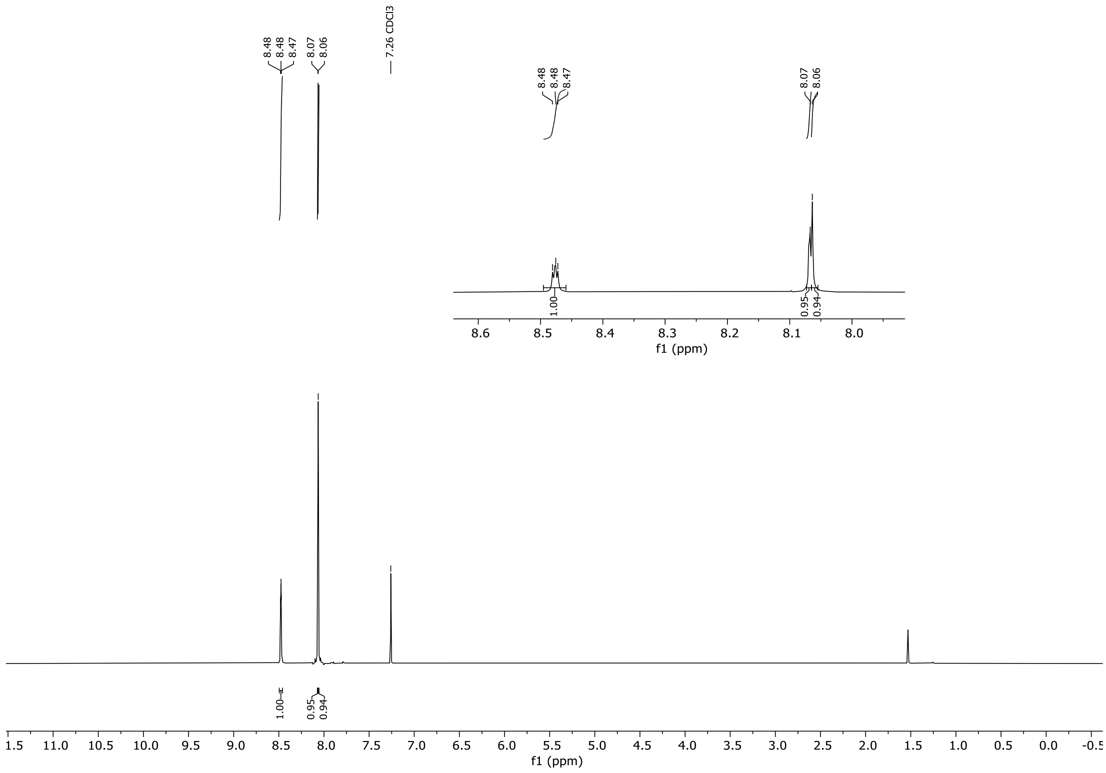

Figure S33: ^{1}H NMR spectrum (300 MHz) of compound **16** in $CDCl_3$.

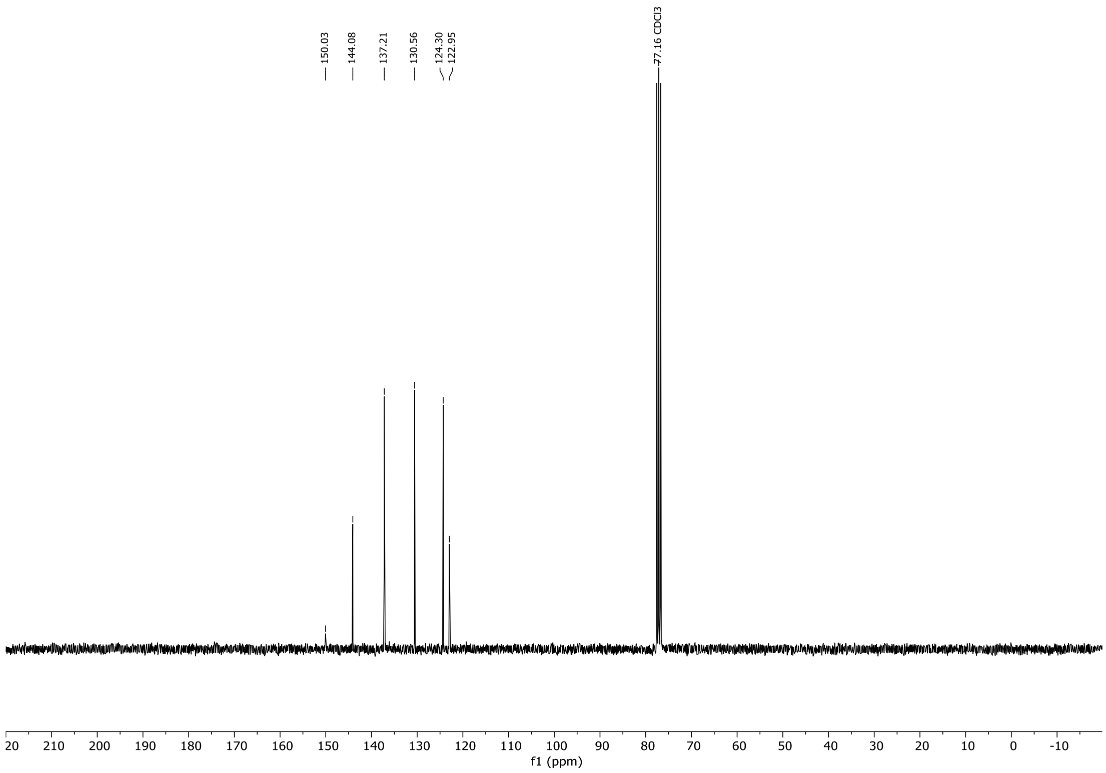

Figure S34: ^{13}C NMR spectrum (75 MHz) of compound **16** in $CDCl_3$.

Synthesis of neopentyl 4-bromo-3-nitrobenzenesulfonate (17)

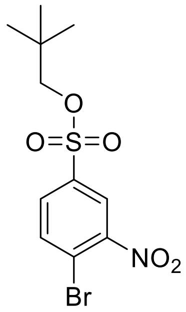

Chemical Formula: $C_{11}H_{14}BrNO_5S$
Molecular Weight: 352,1990

The synthesis follows a modified procedure of Tohnai et al.[15] In a round bottom flask, benzenesulfonyl chloride **16** (17.45 g, 58.07 mmol, 1.0 eq.) and 14.1 ml of pyridine (174.2 mmol, 3.0 eq.) were dissolved in 70 ml of chloroform (0.8 M). The solution was cooled to 0 °C with an ice bath, and 5.63 g neopentyl alcohol (63.88 mmol, 1.1 eq.) dissolved in 30 ml chloroform (2.2 M) were added over 1 hour while continuing cooling. After the addition was completed, the reaction mixture was stirred for additional 30 minutes at 0 °C followed by stirring at room temperature for 2 additional hours. Then, 20 ml of chloroform were added. The organic phase was acidified with HCl (1 M), washed with water, and dried over Na_2SO_4 afterwards. After removal of the solvent, the product was obtained as a colorless solid (18.34 g, 89%).

^{1}H NMR (300 MHz, $CDCl_3$): δ = 8.33 (d, J = 2.0 Hz, 1H), 7.97 (d, J = 8.4 Hz, 1H), 7.92 (dd, J = 8.4, 2.0 Hz, 1H), 3.79 (s, 2H), 0.93 (s, 9H) ppm.

^{13}C NMR (75 MHz, $CDCl_3$): δ = 150.0, 137.3, 136.6, 131.6, 125.0, 120.8, 81.0, 32.0, 26.1 ppm.

ATR-IR [cm^{-1}]: 2966.52 (w), 1589.34 (m), 1566.20 (w), 1531.48 (s), 1475.54 (w), 1465.90 (vw), 1456.26 (vw), 1355.96 (vs),1298.09 (vw), 1288.45 (vw), 1180.44 (vs), 1149.57 (w), 1124.50 (vw), 1099.43 (vw), 1033.85 (m), 948.98 (vs), 935.48 (vs), 889.18 (vs),

854.47 (vs), 833.25 (m), 825.53 (m), 767.67 (m), 750.31 (w), 661.58 (s), 624.94 (m), 596.00 (s), 561.29 (m), 528.50 (w), 459.06 (w), 449.41 (m).

ESI-MS: m/z (+) = calc. 353.2 [M+H]+, found 353.6 [M+H]+

CHNS [%] calc.: C: 37.51, H: 4.01, N: 3.98, S: 9.10
found: C: 37.82, H: 4.12, N: 3.99, S: 8.88

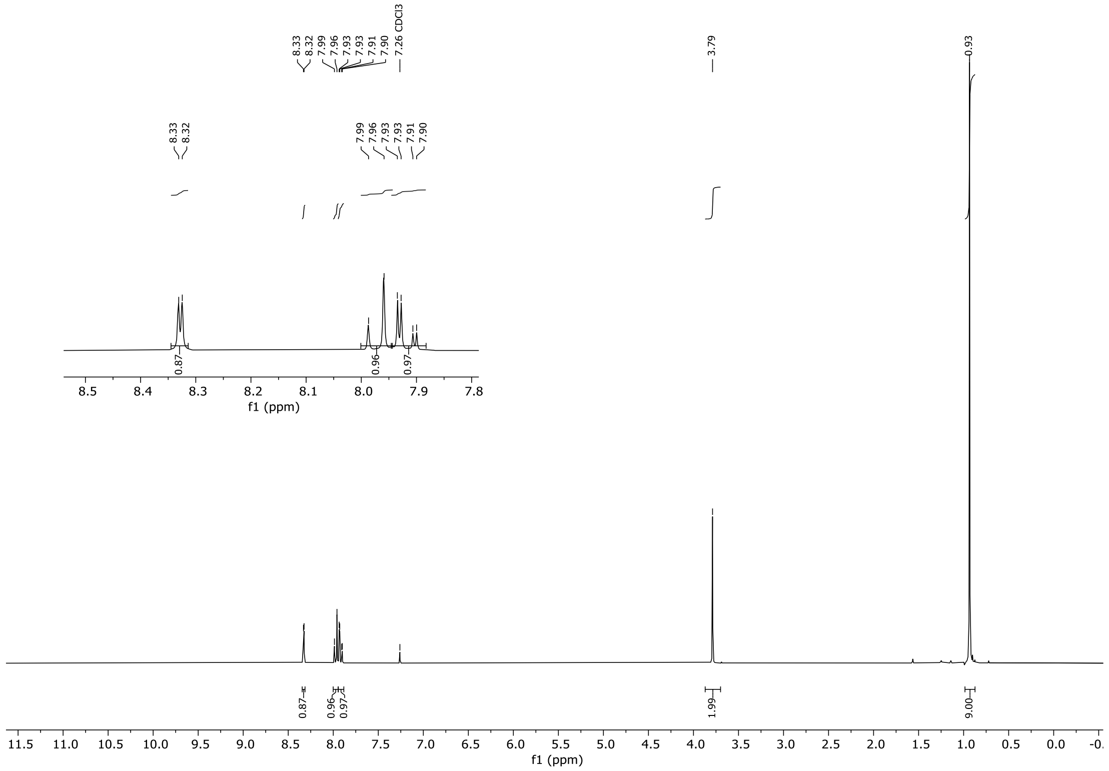

Figure S35: ^{1}H NMR spectrum (300 MHz) of compound **17** in $CDCl_3$.

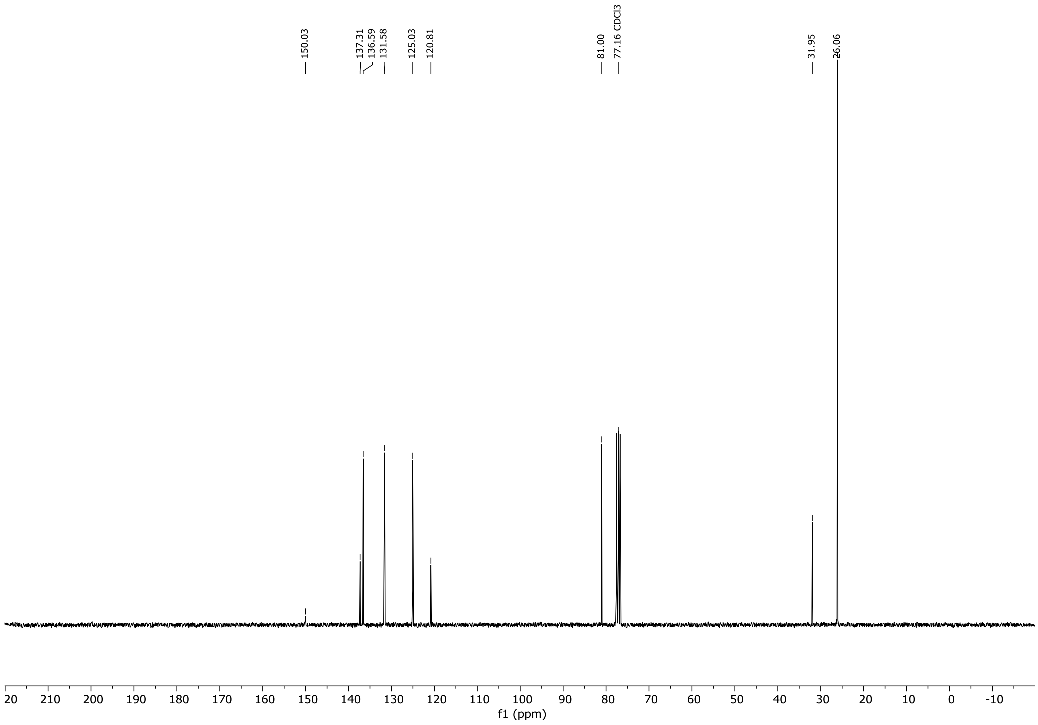

Figure S36: ^{13}C NMR spectrum (75 MHz) of compound **17** in $CDCl_3$.

Synthesis of 2-(3,5-di-tert-butylphenyl)-4,4,5,5-tetramethyl-1,3,2-dioxaborolane (19)

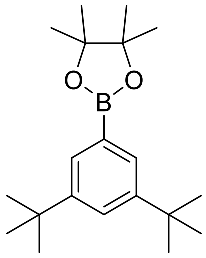

Chemical Formula: $C_{20}H_{33}BO_2$
Molecular Weight: 316,2920

In a flame dried Schlenk flask, 50 ml of dry 1,4-dioxane (0.5 M) were degassed by argon bubbling for 20 minutes and 1-bromo-3,5-di-tert-butylbenzene (7.0 g, 26.0 mmol, 1.0 eq.), bis(pinacolato)diborane (6.6 g, 26.0 mmol, 1.0 eq), bis(triphenylphosphine)-palladium(II)dichloride (0.547 g, 0.78 mmol, 3 mol%) and potassium acetate (7.73 g, 78.0 mmol, 3.0 eq.) were added. The suspension was heated to 110 °C and stirred at this temperature overnight. The next day, the mixture was filtered over celite with EtOAc as eluent and washed with water and brine. The combined organic phases were dried over Na_2SO_4 and the solvent was removed. The yellow solid was recrystallized from EtOAc to obtain the product as colorless crystals (5.35 g, 65%). The NMR data is in good agreement with the literature[16]

^{1}H NMR (300 MHz, $CDCl_3$): δ = 7.70 – 7.64 (m, 2H), 7.64 – 7.51 (m, 1H), 1.38 – 1.32 (m, 30H) ppm.

^{13}C NMR (75 MHz, $CDCl_3$): δ = 150.0, 128.9, 125.7, 83.7, 35.0, 31.7, 25.0 ppm.

ESI-MS: m/z (-) = calc. 395.2 [M+Br]-, found 394.9 [M+Br]-

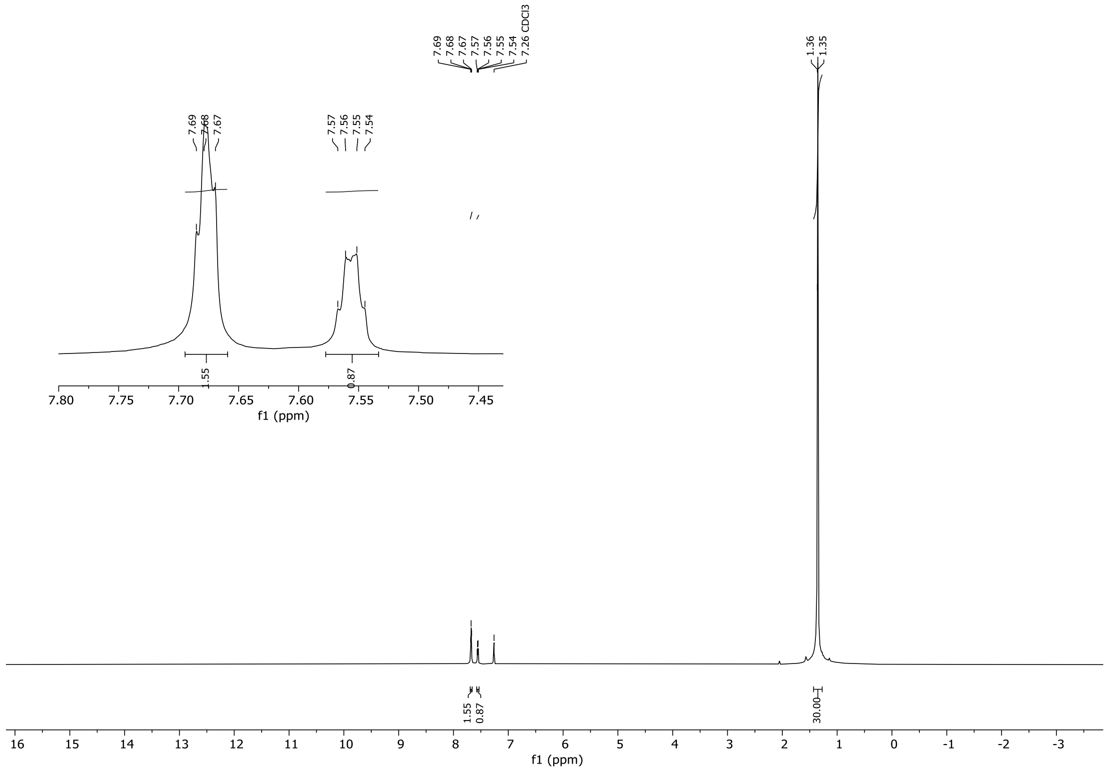

Figure S37: ^{1}H NMR spectrum (300 MHz) of compound **19** in $CDCl_3$.

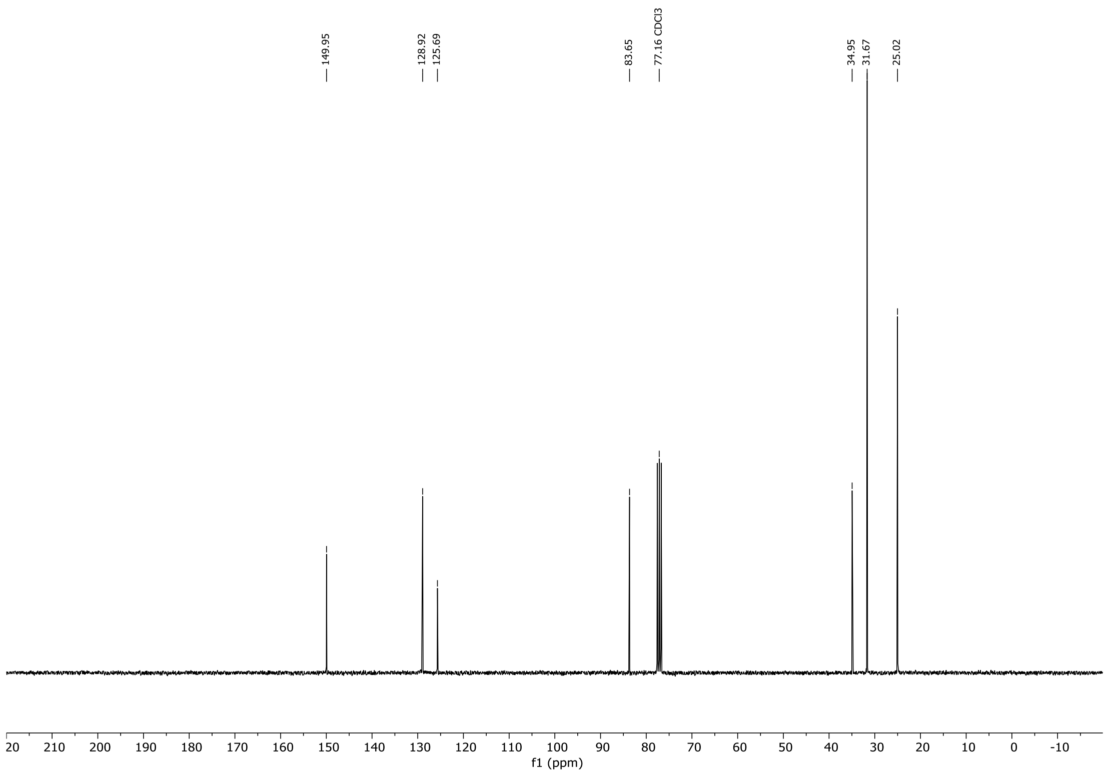

Figure S38: ^{13}C NMR spectrum (75 MHz) of compound **19** in $CDCl_3$.

General coupling procedure to 2-nitro-1,1'-biphenyls 20 and 21

In a Schlenk flask, the bromoarene (1.00 eq.), the boronic acid or acid ester (1.3 eq.) were suspended in 1,4-dioxane and water (4:1, 0.1 M) together with potassium carbonate (3.0 eq.) and $(PPh_3)_2PdCl_2$ (5 mol%). The solvent was degassed by argon bubbling for 20 minutes before it was heated to 70 °C. At this temperature, the reaction was stirred overnight. After cooling to r.t., the suspension was filtered over a plug of celite with EtOAc as eluent. The organic phase was washed twice with water and once with brine. After drying over Na_2SO_4 and filtration, the crude mixture was purified by silica column chromatography.

Synthesis of neopentyl 3',5'-dimethyl-2-nitro-[1,1'-biphenyl]-4-sulfonate (20)

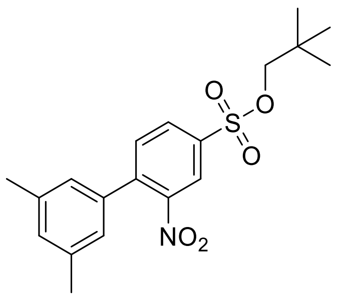

Chemical Formula: $C_{19}H_{23}NO_5S$
Molecular Weight: 377,4550

For the synthesis of **20**, bromo compound **17** (3.0 g, 8.52 mmol, 1.0 eq.), boronic acid **18** (1.66 g, 11.1 mmol, 1.3 eq.), potassium carbonate (3.53 g, 25.6 mmol, 3.0 eq.) and $(PPh_3)_2PdCl_2$ (299 mg, 0.426 mmol, 5 mol%) were reacted in 85 ml 1,4-dioxane and water (4:1, 0.1 M). After work up, the crude mixture was purified by silica column chromatography using pentane and EtOAc as eluent (20:1) to finally isolate the desired product as a yellow oil (2.43 g, 76%).

^{1}H NMR (300 MHz, $CDCl_3$): δ = 8.32 (d, J = 1.8 Hz, 1H), 8.09 (dd, J = 8.1, 1.9 Hz, 1H), 7.65 (d, J = 8.1 Hz, 1H), 7.10 (s, 1H), 6.95 (s, 2H), 3.82 (s, 2H), 2.36 (s, 6H), 0.97 (s,9H) ppm.

^{13}C NMR (75 MHz, $CDCl_3$): δ = 149., 141.7, 138.9, 136.4, 135.5, 133.3, 131.2, 130.8, 125.6, 123.7, 80.7, 32.0, 26.1, 21.4 ppm.

ATR-IR [cm^{-1}]: 2968.45 (vw), 1737.86 (vw), 1602.85 (vw), 1539.20 (s), 1357.89 (vs), 1182.36 (vw), 1153.43 (vw), 1118.71 (vw), 1103.28 (vw), 947.05 (s), 931.62 (s), 898.83 (m), 839.03 (vs), 763.81 (m), 754.17 (vw), 719.45 (vw), 694.37 (vw), 665.44 (m), 630.72 (m), 599.86 (s), 569.00 (vs), 545.85 (vw), 536.21 (vw), 528.50 (vw), 462.92 (vw), 433.98 (w).

FD-MS: m/z (+) = calc.: 377.1297 [M]+, found: 377.1283 [M]+

CHNS [%] calc.: C:60.46, H:6.14, N: 3.71, S: 8.50
found: C: 60.85,H: 6.18, N 3.40, S: 8.34

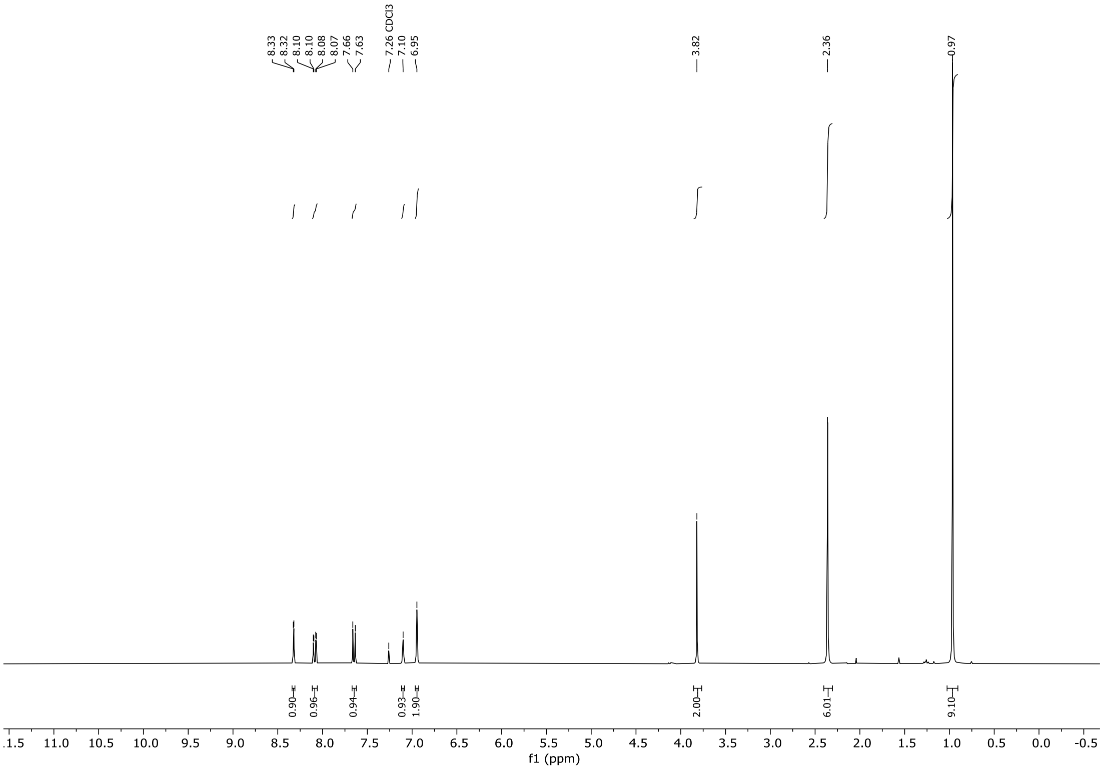

Figure S39: ^{1}H NMR spectrum (300 MHz) of compound **20** in $CDCl_3$.

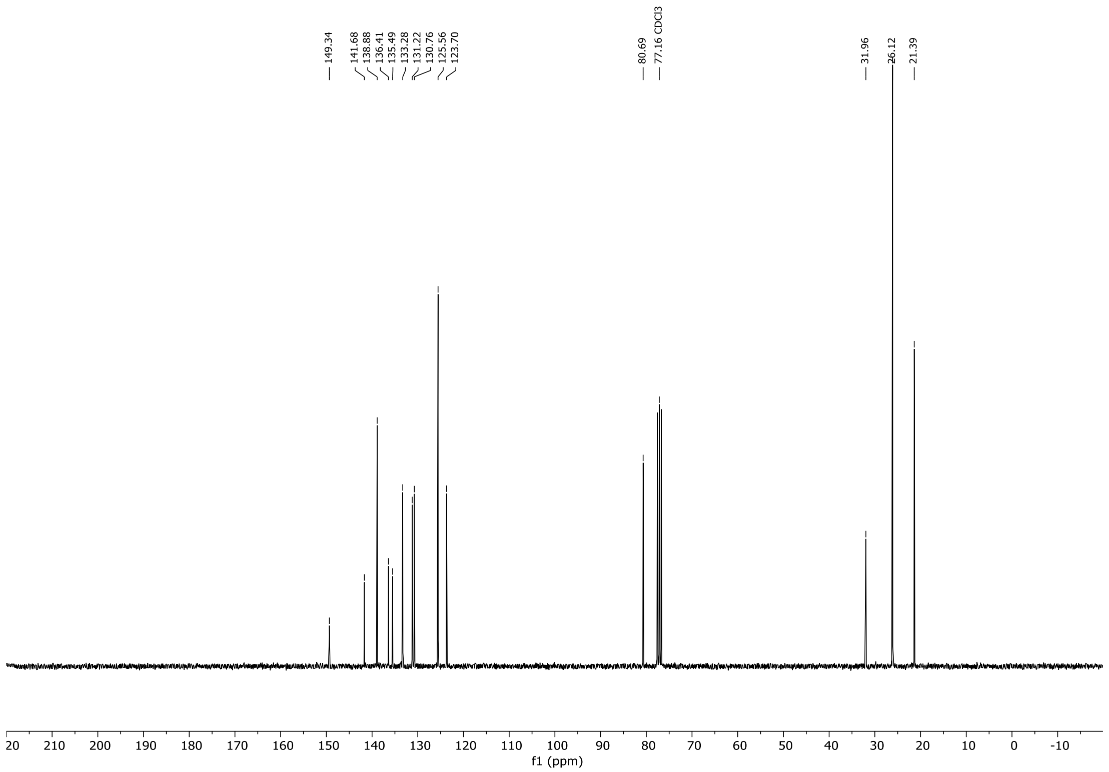

Figure S40: ^{13}C NMR spectrum (75 MHz) of compound **20** in $CDCl_3$.

Synthesis of neopentyl 3',5'-di-tert-butyl-2-nitro-[1,1'-biphenyl]-4-sulfonate (21)

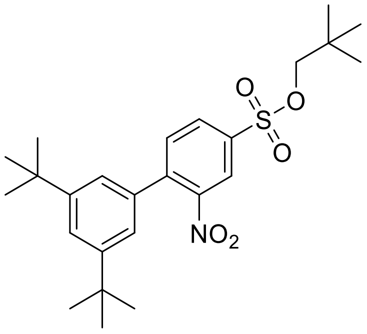

Chemical Formula: $C_{25}H_{35}NO_5S$
Molecular Weight: 461,6170

For the synthesis of **21**, bromo compound **17** (2.0 g, 5.68 mmol, 1.0 eq.), boronic acid ester **19** (2.34 g, 7.38 mmol, 1.3 eq.), potassium carbonate (2.36 g, 17.0 mmol, 3.0 eq.) and $(PPh_3)_2PdCl_2$ (199 mg, 0.284 mmol, 5 mol%) were reacted in 50 ml 1,4-dioxane and water (4:1, 0.1 M). After work up, the crude mixture was purified by silica column chromatography using pentane and EtOAc as eluent (20:1) to finally isolate the desired product as a yellow oil (2.5 g, 95%).

^{1}H NMR (300 MHz, $CDCl_3$): δ = 8.30 (d, *J* = 1.8 Hz, 1H), 8.09 (dd, *J* = 8.2, 1.9 Hz, 1H), 7.70 (d, *J* = 8.1 Hz, 1H), 7.52 (t, *J* = 1.8 Hz, 1H), 7.16 (d, *J* = 1.7 Hz, 2H), 3.82 (s, 2H), 1.34 (s, 18H), 0.97 (s, 9H) ppm.

^{13}C NMR (75 MHz, $CDCl_3$): δ = 151.8, 149.7, 142.3, 136.2, 134.6, 133.3, 130.7, 123.6, 122.3, 80.7, 35.2, 32.0, 31.5, 26.2 ppm.

ATR-IR [cm^{-1}]: 2961.70 (m), 2870.08 (vs), 1596.10 (vs), 1535.34 (w), 1476.51 (s), 1359.82 (w), 1247.94 (s), 1184.29 (vw), 1097.50 (vs), 956.69 (w), 936.44 (m), 897.86 (m), 880.50 (s), 835.18 (m), 795.64 (m), 771.53 (vs), 760.92 (s), 747.42 (vs), 715.59 (m), 665.44 (m), 638.44 (vs), 623.01 (vs), 599.86 (w), 578.64 (m), 543.93 (s), 460.99 (vs).

ESI-MS: m/z (-) = calc. 520.2 $[M+C_2H_4O_2-H]^-$, found 520.9 $[M+C_2H_4O-H]^-$

CHNS [%]: calc.: C: 65.05., H: 7.64, N: 3.03, S: 6.95
found: C: 65.17., H: 7.63, N: 2.94, S: 6.37

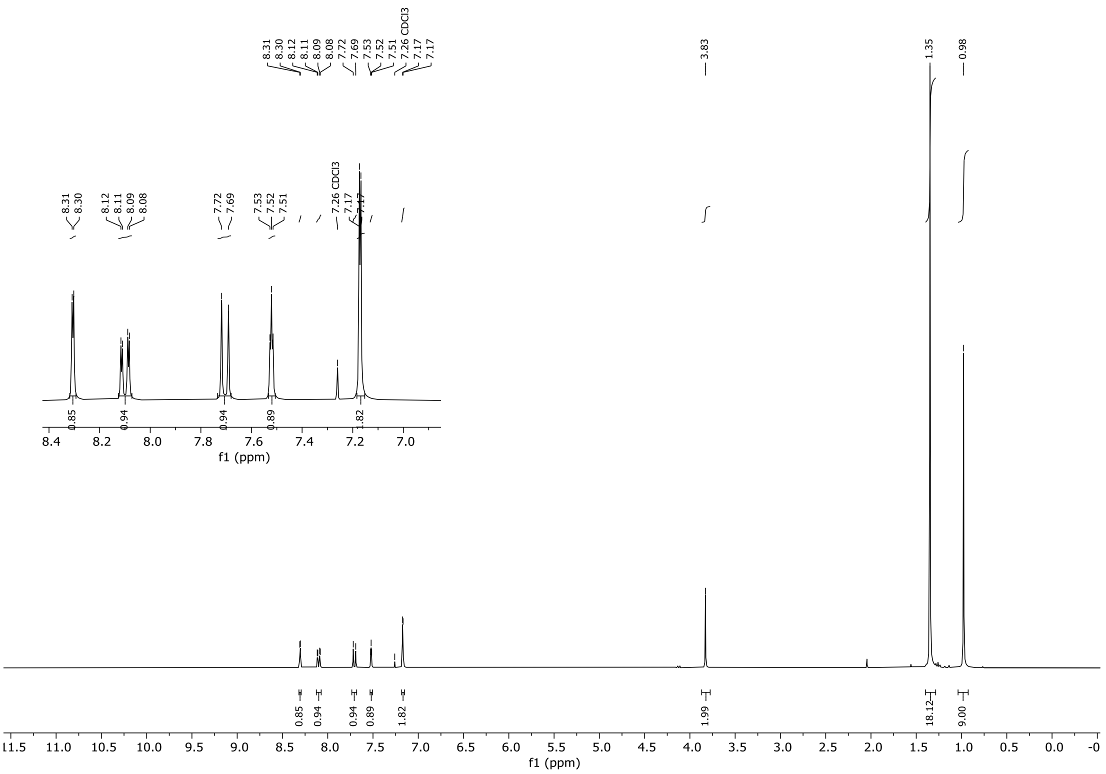

Figure S41: ^{1}H NMR spectrum (300 MHz) of compound **21** in $CDCl_3$.

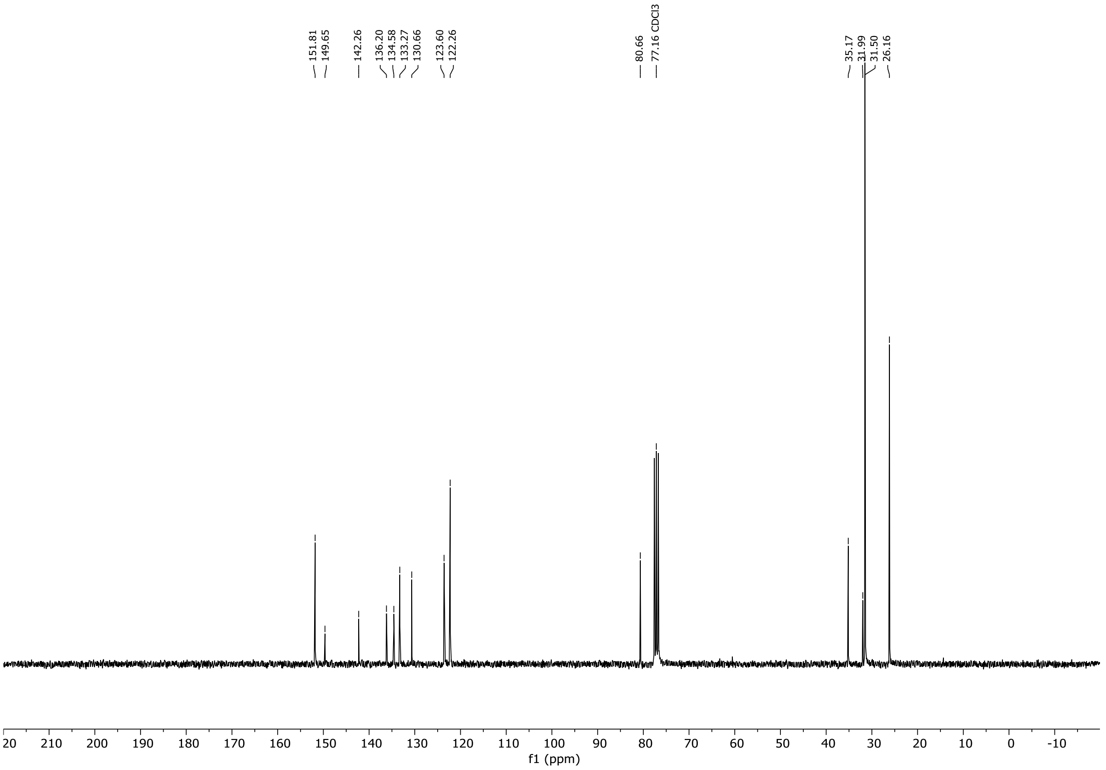

Figure S42: ^{13}C NMR spectrum (75 MHz) of compound **21** in $CDCl_3$.

General reduction procedure to amines 22 and 23

The nitro compound (1.0 eq.) was suspended in EtOH (0.1 M) and 2 mol% of Pd on charcoal (4% of Pd on charcoal) were added. The reaction was stirred with an hydrogen atmosphere until the reduction was completed (followed by TLC). The mixture was filtered over a plug of celite using EtOAc as eluent. The solvent was removed and the crude mixture was purified by silica column chromatography.

Synthesis of neopentyl 2-amino-3',5'-di-methyl-[1,1'-biphenyl]-4-sulfonate (22)

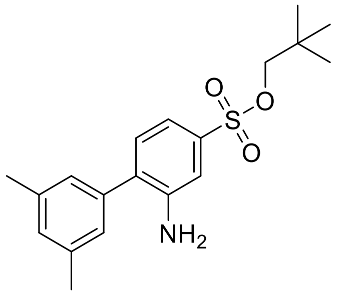

Chemical Formula: $C_{19}H_{25}NO_3S$
Molecular Weight: 347,4730

Nitro compound **20** (600 mg, 1.59 mmol, 1.0 eq.) and Pd/C (33.8 mg, 10%, 0.0318 mmol, 2 mol%) were reacted in EtOH (0.2 M) under an hydrogen atmosphere. After workup, the no further purification was necessary. Amine **22** was isolated as white solid (562.5 mg, 99%, 97% purity).

^{1}H NMR (300 MHz, DMSO-*d6*): δ = 7.29 (d, J = 1.9 Hz, 1H), 7.19 (d, J = 7.9 Hz, 1H), 7.10-6.98 (m, 4H), 5.36 (s, 2H), 3.71 (s, 2H), 2.32 (s, 6H), 0.88 (s, 9H) ppm.

^{13}C NMR (75 MHz, DMSO-*d6*): δ = 146.2, 138.0, 137.6, 134.5, 130.9, 130.7, 129.1, 126.1, 114.5, 113.0, 79.1, 31.3, 25.6, 20.9 ppm.

ATR-IR [cm^{-1}]: 3474.90 (vw), 3370.66 (vw), 2944.98 (vw), 1789.58 (w), 1694.01 (w), 1624.52 (m), 1572.39 (w), 1468.15 (w), 1407.34 (w), 1346.53 (s), 1320.46 (m), 1268.34 (m), 1172.78 (vs), 1103.28 (w), 939.33 (s), 914.26 (m), 854.47 (m), 831.32 (s), 817.82 (vs), 707.88 (w), 700.16 (m), 626.87 (m), 605.65 (m), 532.35 (w).

ESI-MS: m/z (+) = calc. 389.2 [$M+CH_3CN+H$]+, found 389.3 [$M+CH_3CN+H$]+

CHNS [%]: calc.: C: 65.68, H: 7.25, N: 4.03, S: 9.23
found: C: 65.96, H: 7.31, N: 4.00, S: 8.90

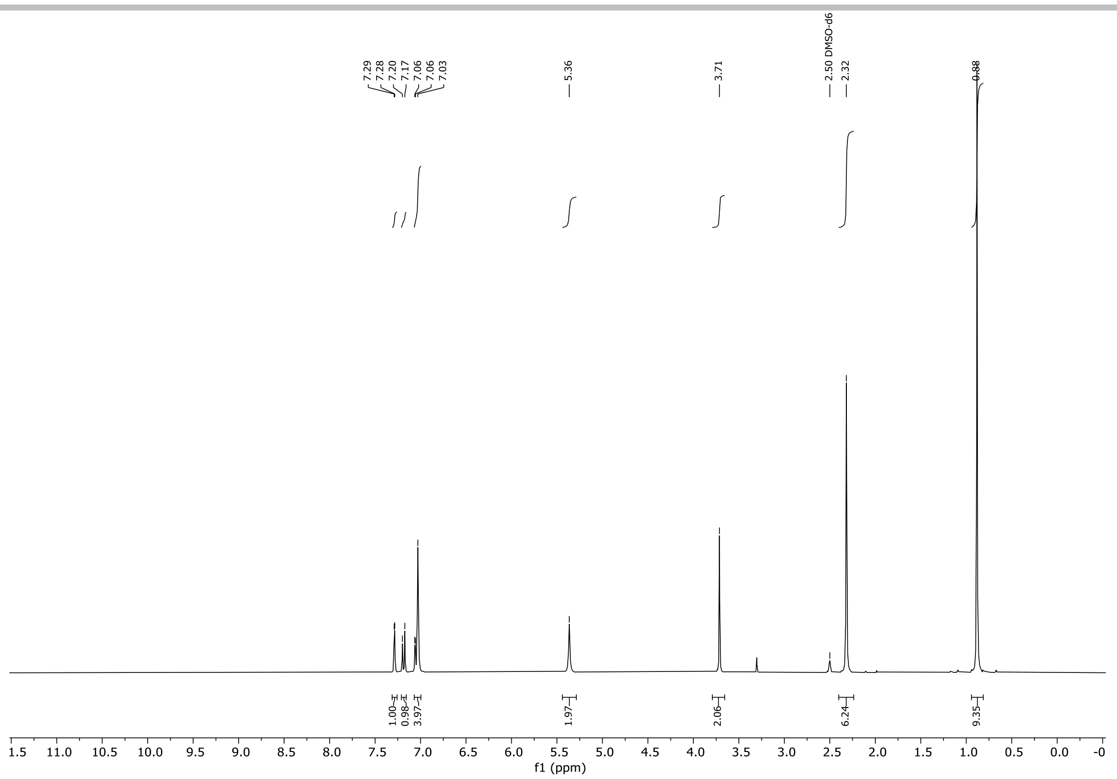

Figure S43: ^{1}H NMR spectrum (300 MHz) of compound **22** in DMSO-*d6*.

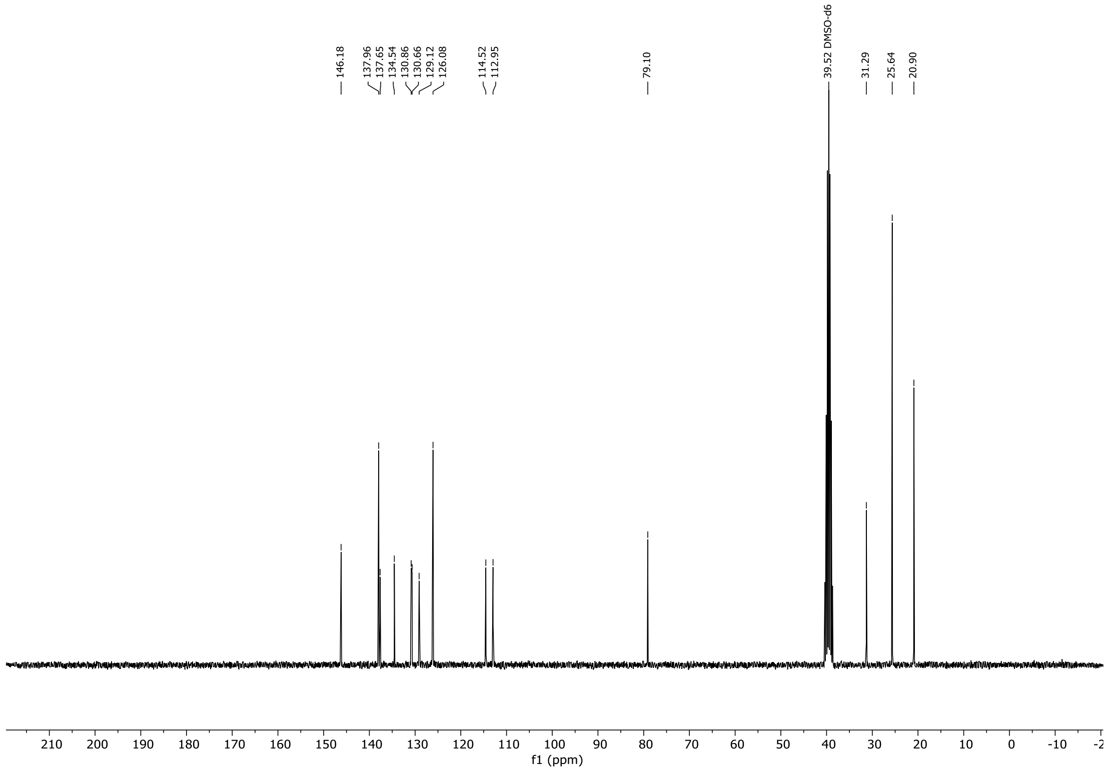

Figure S44: ^{13}C NMR spectrum (75 MHz) of compound **22** in DMSO-*d6*.

Synthesis of neopentyl 2-amino-3’,5’-di-tert-butyl-[1,1’-biphenyl]-4-sulfonate (23)

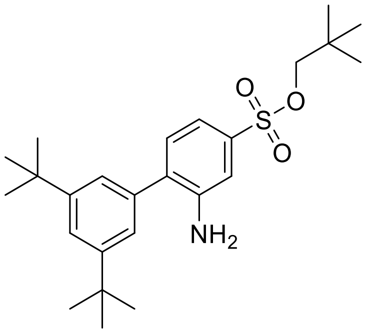

Chemical Formula: $C_{25}H_{37}NO_3S$
Molecular Weight: 431,6350

Nitro compound **21** (1.24 g, 2.69 mmol, 1.0 eq.) and Pd/C (143 mg, 4%, 0.0538 mmol, 2 mol%) were reacted in EtOH (0.2 M) under an hydrogen atmosphere. After workup, the crude mixture was purified by silica column chromatography using pentane and EtOAc as eluent (20:1). Amine **23** was isolated as white solid (1.05 g, 90%).

^{1}H NMR (300 MHz, DMSO-*d6*) δ = 7.44 (s, 1H), 7.33 – 7.19 (m, 4H), 7.06 (d, *J* = 11.2 Hz, 1H), 5.34 (s, 2H), 3.72 (s, 2H), 1.32 (s, 18H), 0.90 (s, 9H) ppm.

^{13}C NMR (75 MHz, DMSO-*d6*): δ = 150.9, 146.2, 137.0, 134.6, 131.3, 131.2, 122.4, 121.6, 114.7, 113.1, 79.1, 34.6, 31.4, 31.2, 25.7 ppm.

ATR-IR [cm^{-1}]: 3489.23 (vs), 3384.11 (vs), 961.70 (s), 1620.21 (s), 1592.24 (s), 1477.47 (s), 1409.00 (s), 1362.71 (m), 1349.21 (m), 1329.92 (m), 1315.45 (m), 1246.02 (s), 1175.61 (vw), 958.62 (w), 937.40 (m), 879.54 (s), 844.82 (m), 813.96 (m), 758.02 (vs), 717.52 (m), 696.30 (m), 624.94 (m), 604.68 (w), 526.57 (s).

ESI-MS: m/z (+) = calc. 449.3 [M+NH_4]+, found 449.9 [M+NH_4]+

CHNS [%]: calc.: C: 69.57, H: 8.64, N: 3.25, S: 7.43
found: C: 69.54, H: 8.47, N: 3.14, S: 6.77

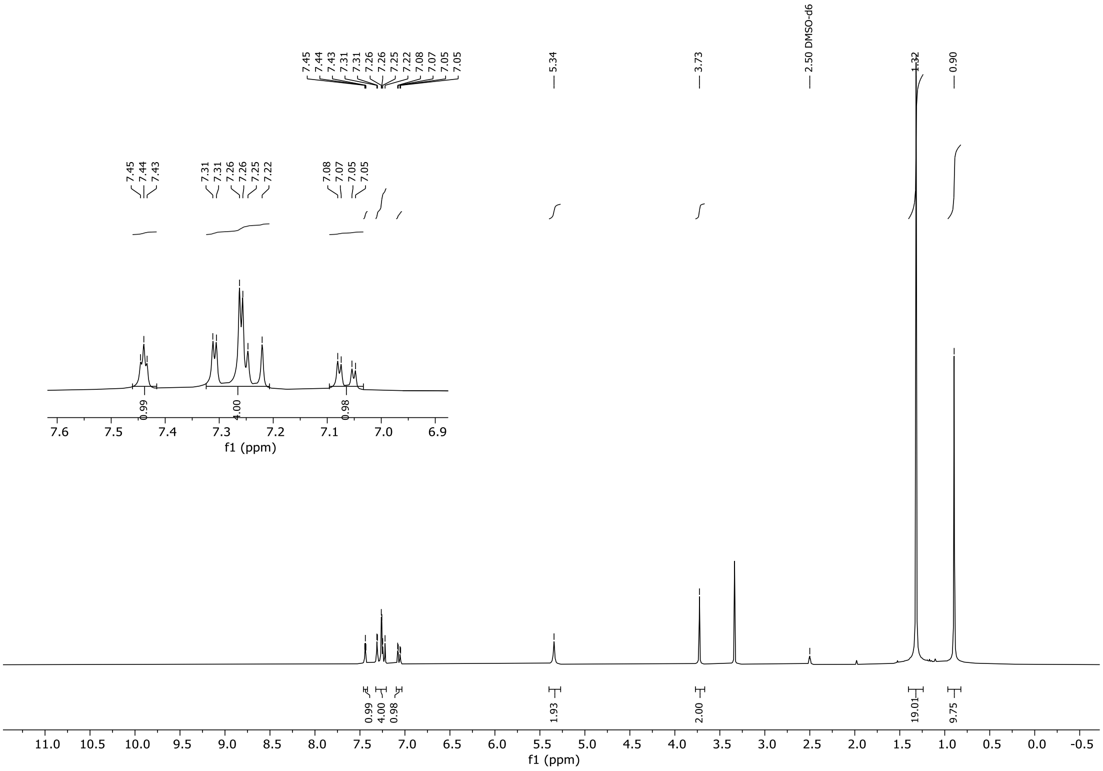

Figure S45: ^{1}H NMR spectrum (300 MHz) of compound **23** in DMSO-*d₆*.

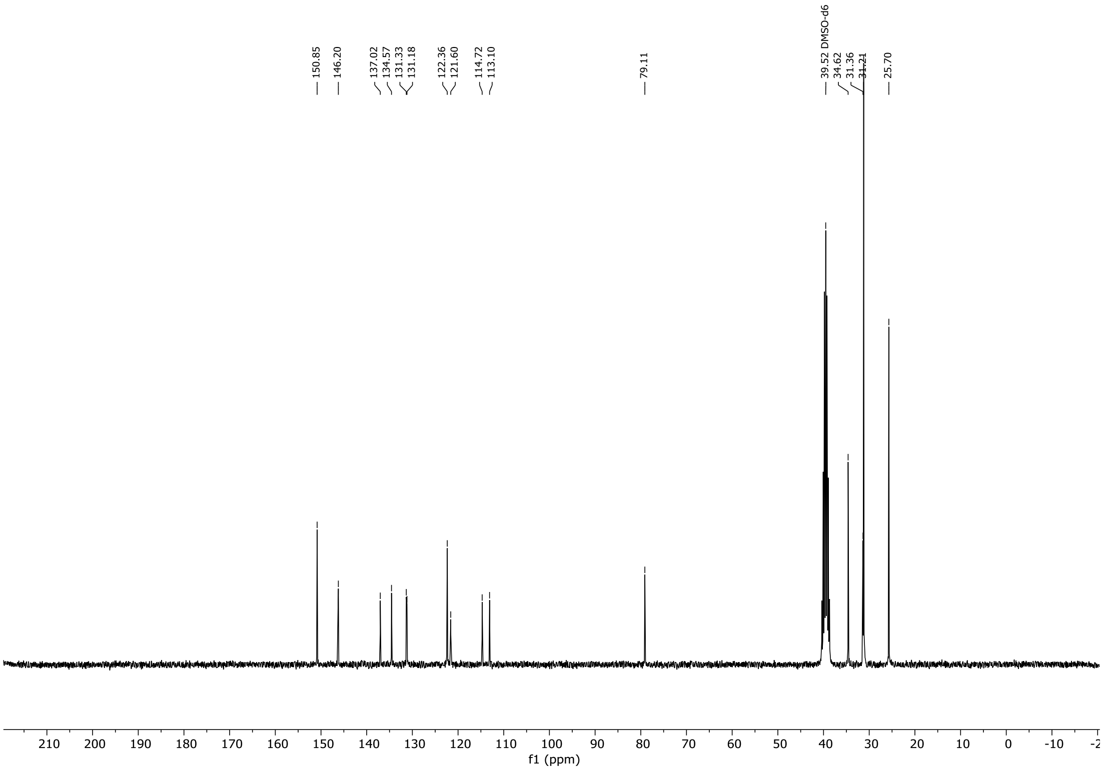

Figure S46: ^{13}C NMR spectrum (75 MHz) of compound **23** in DMSO-*d₆*.

General procedure for Sandmeyer type reaction

The Sandmeyer type reaction follows the procedure of Samanta et al.[17]

Synthesis of neopentyl neopentyl 3',5'-dimethyl-2-iodo-[1,1'-biphenyl]-4-sulfonate (24)

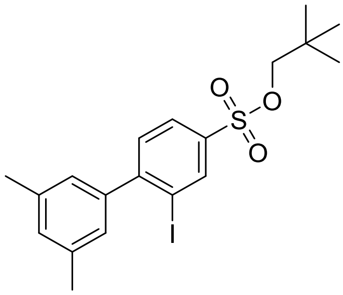

Chemical Formula: $C_{19}H_{23}IO_3S$
Molecular Weight: 458,3545

Amine **22** (1.00 g, 2.73 mmol, 1.0 eq.) was reacted in 14 ml of ACN (0.2 M) according to the procedure of Samanta et al.[17] The crude mixture was purified by silica column chromatography using pentane and EtOAc as eluent (10:1) resulting in 1.12 g (90%) of the desired product **24**.

^{1}H NMR (300 MHz, $CDCl_3$): δ = 8.45 (d, *J* = 1.8 Hz, 1H), 7.88 (dd, *J* = 8.0, 1.9 Hz, 1H), 7.44 (d, *J* = 8.0 Hz, 1H), 7.08 (s, 1H), 6.95 (s, 2H), 3.77 (s, 2H), 2.39 (s, 6H), 0.96 (s, 9H) ppm.

^{13}C NMR (75 MHz, $CDCl_3$): δ = 152.4, 142.6, 138.5, 138.0, 136.0, 130.4, 130.2, 127.3, 126.7, 98.6, 80.2, 31.9, 26.2, 21.5 ppm.

ATR-IR [cm^{-1}]: 2971.04 (vw), 1607.14 (vw), 1581.08 (vw), 1468.15 (w), 1367.53 (s), 1354.03 (w), 1340.53 (vs), 1178.51 (vs), 1107.14 (w), 1033.85 (w), 950.91 (vs), 939.33 (s), 920.05 (w), 871.82 (s), 860.25 (s), 846.75 (vs), 829.39 (s), 758.02 (w), 746.45 (m), 729.09 (m), 702.09 (m), 655.80 (m), 603.72 (vs), 542.00 (m), 526.57 (w), 459.09 (w), 435.91 (w), 408.91 (w).

FD-MS: m/z (+) = calc.: 458.0413, found: 458.0420

CHNS [%]: calc.: C: 49.79, H: 5.06, N: 0.00, S: 7.00
found: C: 50.13, H: 5.13, N: 0.00, S: 6.74

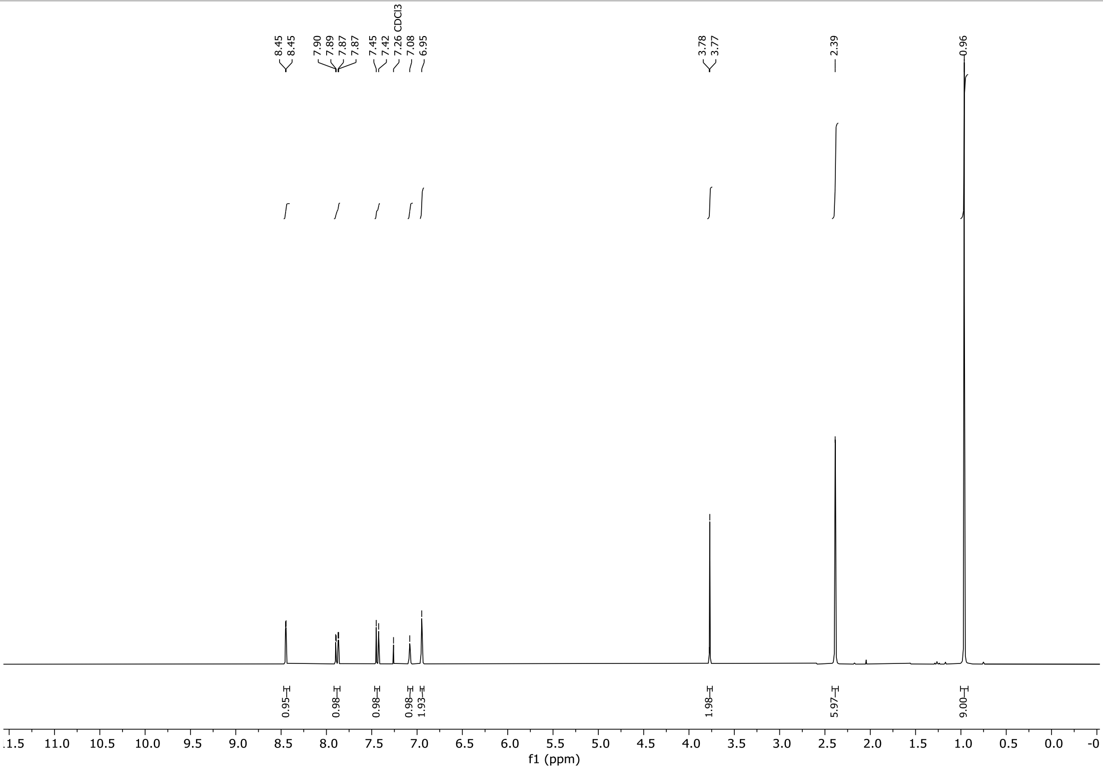

Figure S47: ^{1}H NMR spectrum (300 MHz) of compound **24** in $CDCl_3$.

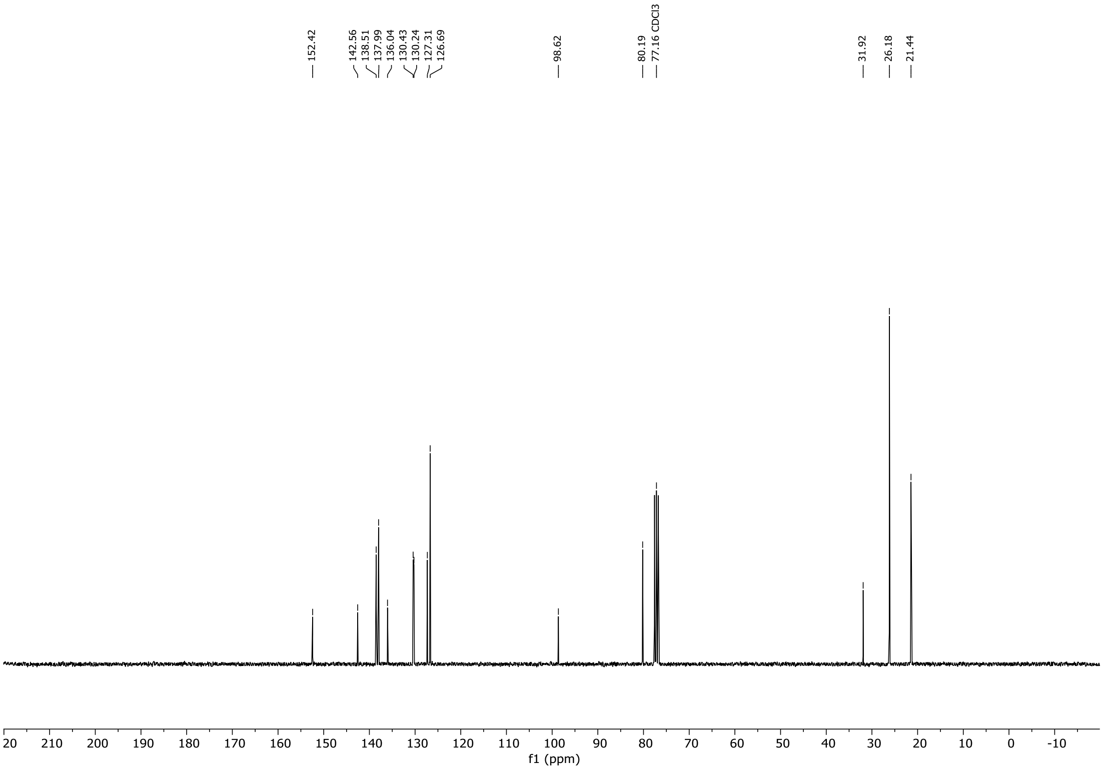

Figure S48: ^{13}C NMR spectrum (75 MHz) of compound **24** in $CDCl_3$.

Synthesis of neopentyl 3',5'-di-tert-butyl-2-iodo-[1,1'-biphenyl]-4-sulfonate (25)

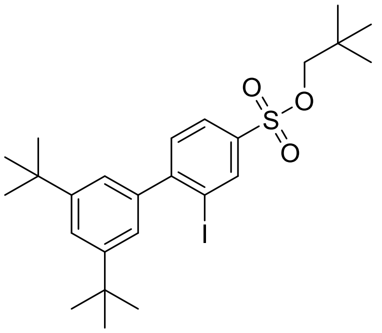

Chemical Formula: $C_{25}H_{35}IO_3S$
Molecular Weight: 542,5165

Amine **23** (800 mg, 1.85 mmol, 1.0 eq.) was reacted in 14 ml of ACN (0.2 M) according to the procedure of Samanta et al.[17] The crude mixture was purified by silica column chromatography using pentane and EtOAc as eluent (15:1) resulting in 886 mg (88%) of the desired product **25**.

^{1}H NMR (300 MHz, $CDCl_3$): δ = 8.48 (d, *J* = 1.8 Hz, 1H), 7.91 (dd, *J* = 8.0, 1.9 Hz, 1H), 7.55 – 7.46 (m, 2H), 7.20 (d, *J* = 1.7 Hz, 2H), 3.79 (s, 2H), 1.38 (s, 18H), 0.98 (s, 9H) ppm.

^{13}C NMR (75 MHz, $CDCl_3$): δ = 153.0, 150.8, 141.6, 138.7, 135.8, 130.6, 127.4, 123.5, 122.4, 98.8, 80.1, 35.1, 31.9, 31.6, 26.2 ppm.

ATR-IR [cm^{-1}]: 2954.95 (m), 2900.94 (s), 2866.22 (s), 1596.10 (s), 1578.74 (vs), 1474.58 (m), 1425.40 (s), 1354.03 (vw), 1248.91 (s), 1179.47 (vw), 1107.14 (m), 1029.02 (m), 958.62 (vw), 936.44 (w), 878.58 (m), 867.97 (s), 825.53 (vw), 798.53 (m), 729.09 (w), 712.70 (w), 654.83 (w), 630.72 (s), 597.93 (vw), 546.82 (m), 462.92 (s), 449.41 (vs).

ESI-MS: m/z (+) = calc. 606.2 [M+ACN+Na]+, found 604.4 [M+ACN+Na]+

CHNS [%]: calc.: C: 55.35, H: 6.50, N: 0.00, S: 5.91
found: C: 55.54, H: 6.38, N: 0.00, S: 5.56

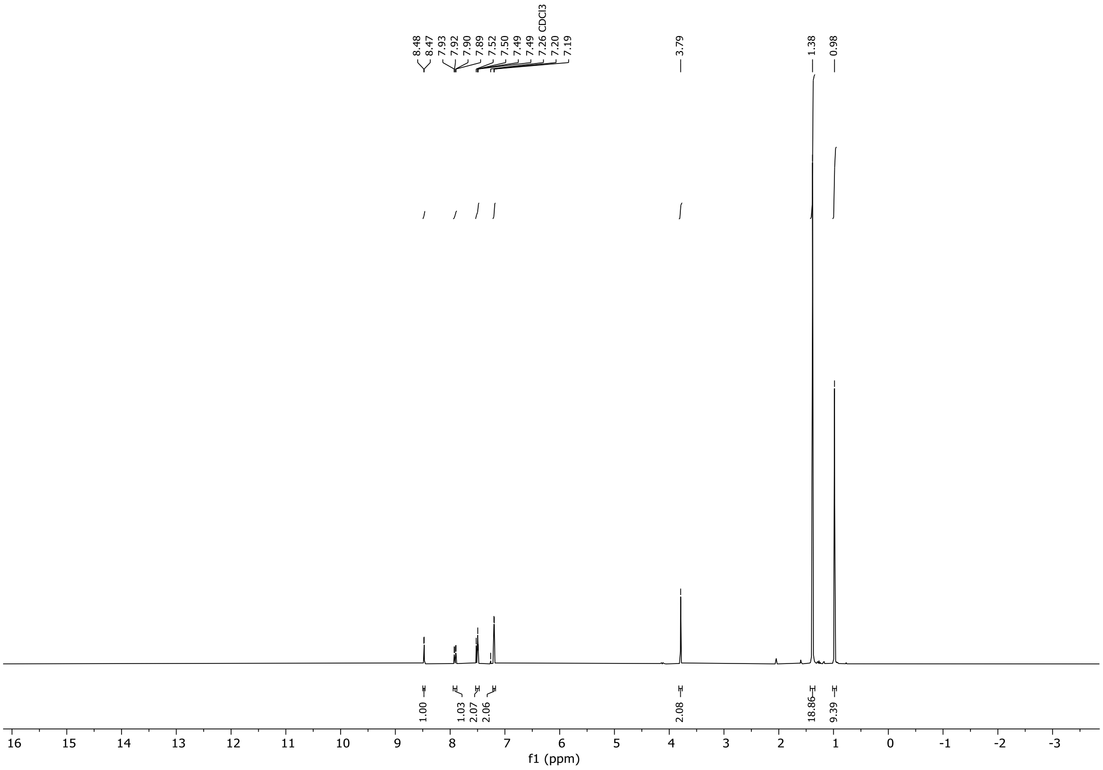

Figure S49: ^{1}H NMR spectrum (300 MHz) of compound **25** in $CDCl_3$.

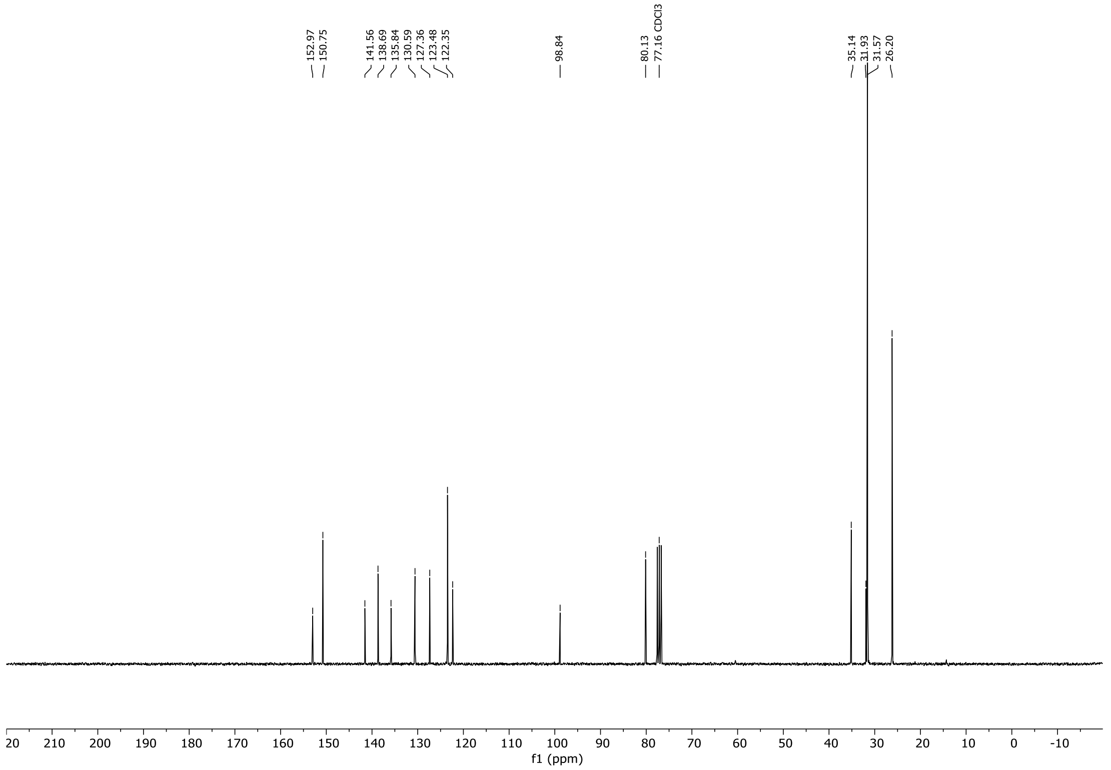

Figure S50: ^{13}C NMR spectrum (75 MHz) of compound **25** in $CDCl_3$.

General procedure for sulfonic acid 26 and 27

The sulfonic acid ester was suspended in TFA (0.2 M) and heated to 70 °C. At this temperature, the mixture was stirred overnight. After cooling to r.t., the volatile components were removed under reduced pressure and co evaporated twice with DCM. The obtained residue was redissolved in DCM and filtered through a Whatman filter. After removal of the solvent, the product was obtained and used without further purification.

Synthesis of 2-iodo-3',5'-dimethyl-[1,1'-biphenyl]-4-sulfonic acid (26)

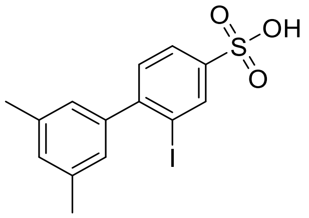

Chemical Formula: $C_{14}H_{13}IO_3S$
Molecular Weight: 388,2195

Sulfonic acid ester **24** (505 mg, 1.10 mmol, 1.0 eq.) was reacted in TFA (0.2 M). After workup, the product was obtained as a purple solid (423 mg, 99%).

^{1}H NMR (300 MHz, $CDCl_3$): δ = 9.18 (s, 1H), 8.41 (d, *J* = 1.8 Hz, 1H), 7.82 (dd, *J* = 8.0, 1.8 Hz, 1H), 7.24 (d, *J* = 8.0 Hz, 1H), 6.98 (s, 1H), 6.81 (s, 2H), 2.28 (s, 6H) ppm.

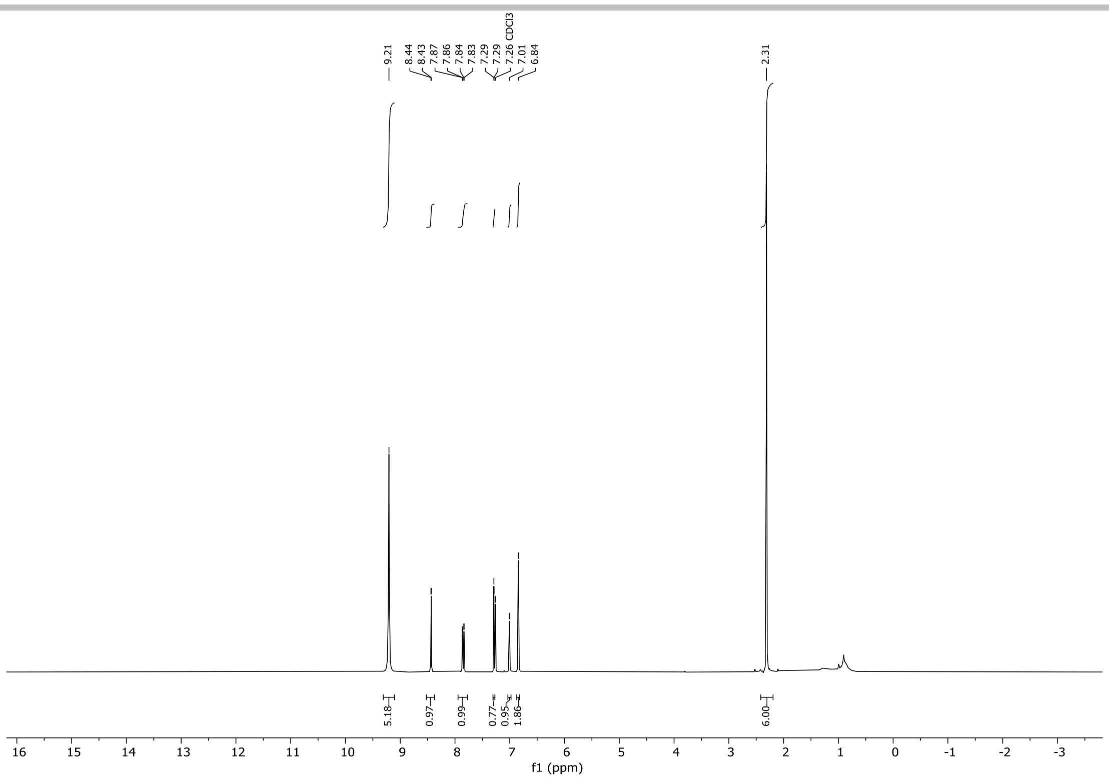

Figure S51: ^{1}H NMR spectrum (300 MHz) of compound **26** in $CDCl_3$.

Synthesis of 3',5'-di-tert-butyl-2-iodo-[1,1'-biphenyl]-4-sulfonic acid (27)

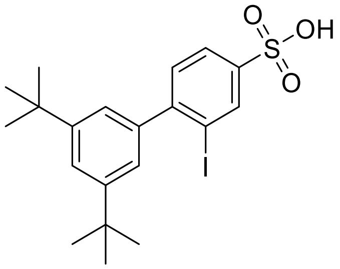

Chemical Formula: $C_{20}H_{25}IO_3S$
Molecular Weight: 472,3815

Sulfonic acid ester **24** (600 mg, 1.11 mmol, 1.0 eq.) was reacted in TFA (0.2 M). After workup, the product was obtained as a dark oil (218 mg, 42%).

^{1}H NMR (300 MHz, DMSO-*d*$_6$) δ = 8.15 (d, *J* = 1.7 Hz, 1H), 7.35 (m, 5H), 1.32 (s, 18H) ppm.

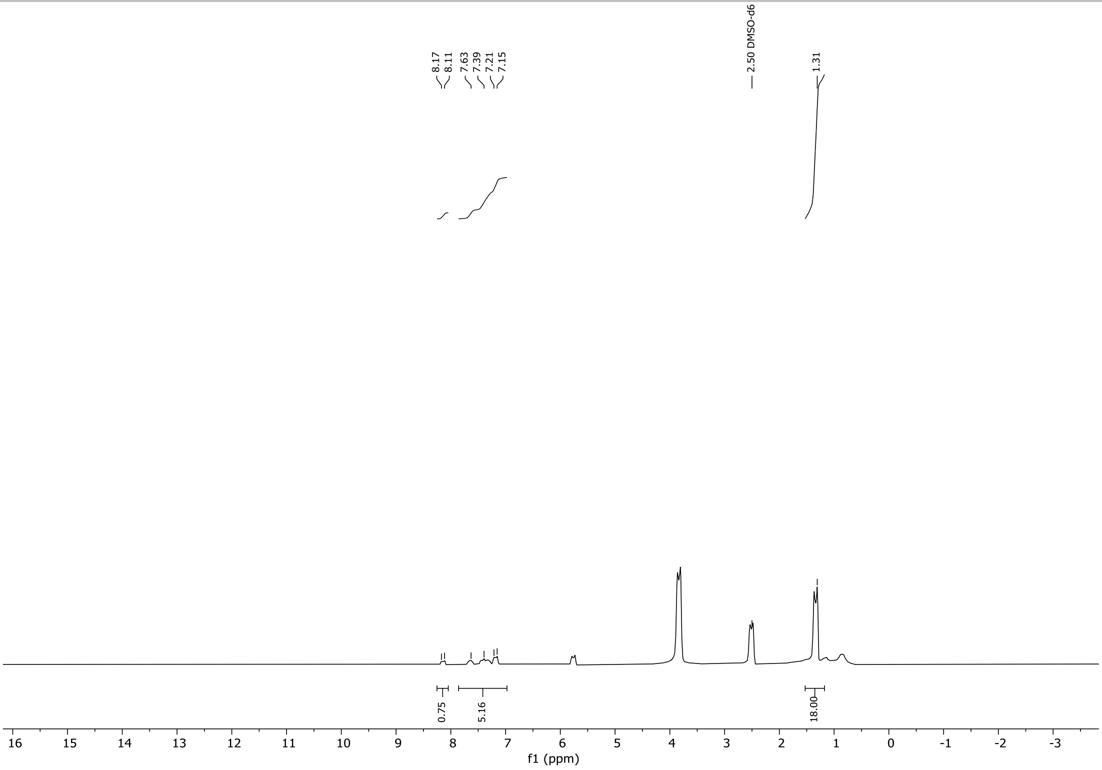

Figure S52: ^{1}H NMR spectrum (300 MHz) of compound **27** in DMSO-d_6.

General procedure for the oxidation to iodolium 28 and 29

The oxidation follows the procedure of Oloffson et al.[13] The iodo compound (1.0 eq.) was dissolved in DCM (0.2 M) and mCPBA (1.5 eq.) was added. The reaction mixture was stirred at r.t. for 10 minutes before it was cooled to 0 °C with an ice bath. To that, HOTf (3.0 eq.) was added, and the suspension was stirred overnight while warming to r.t.. The volatile components were removed under reduced pressure and the remaining solid was washed by stirring in DEE. Finally, the product was isolated by filtration.

Synthesis of 2,4-di-methyl-7-sulfodibenzo[b,d]iodol-5-ium trifluoromethanesulfonate (28)

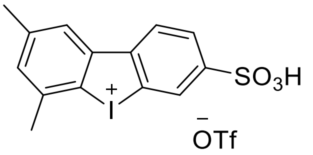

Chemical Formula: $C_{15}H_{12}F_3IO_6S_2$
Molecular Weight: 536,2747

Iodo compound **26** (300 mg, 0.75 mmol, 1.0 eq.) was dissolved in DCM (0.2 M) and reacted with mCPBA (250 mg, 1.12 mmol, 1.5 eq), cooled to 0 °C and HOTf (0.2 mL, 2.23 mmol, 3.0 eq.) was added. After stirring overnight and working up, the product was isolated as a white solid (294 mg, 74%).

^{1}H NMR (400 MHz, DMSO-d_6): δ = 8.61 (d, J = 1.5 Hz, 1H), 8.39 (d, J = 8.3 Hz, 1H), 8.15 (s, 1H), 8.00 (dd, J = 8.1, 1.5 Hz, 1H), 7.41 (s, 1H), 2.65 (s, 3H), 2.48 (s, 3H) ppm.[14]

^{19}F NMR (376 MHz, DMSO-d_6): δ = -77.72 ppm.

ATR-IR [cm^{-1}]: 1694.02 (vw), 1581.08 (vw), 1459.46 (vw), 1372.59 (w), 1274.95 (s), 1240.23 (s), 1224.80 (vs), 1193.94 (s), 1168.86 (vs), 1118.71 (s), 1041.56 (s), 1028.06 (vs), 1010.70 (m), 1001.06 (m), 860.25 (m), 844.82 (m), 742.59 (m), 661.58 (s), 636.51 (s), 624.94 (vs), 605.65 (vs), 590.22 (m), 576.72 (m), 543.93 (w), 528.50 (w), 516.92 (m), 491.85 (m), 451.34 (m), 406.98 (w).

ESI-MS: m/z (+) = calc. 387.0 [M-OTF-]+, found 387.1 [M-OTf-]+
m/z (-) = calc. 614.8 [M+Br-]-, found 614.9 [M+Br-]-

CHNS [%]: calc.: C: 33.59, H: 2.26, N: 0.00, S: 11.96
found: C: 36.29, H: 3.07, N: 0.00, S: 9.84

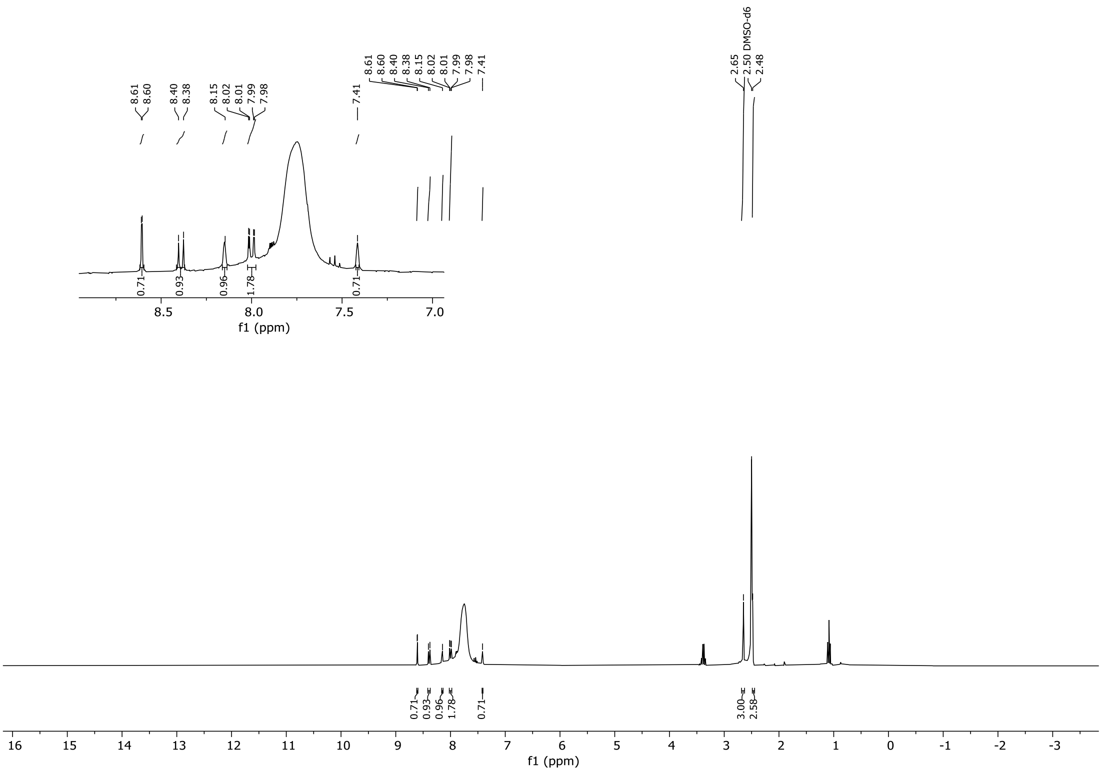

Figure S53: ^{1}H NMR spectrum (300 MHz) of compound **28** in DMSO-*d6*.

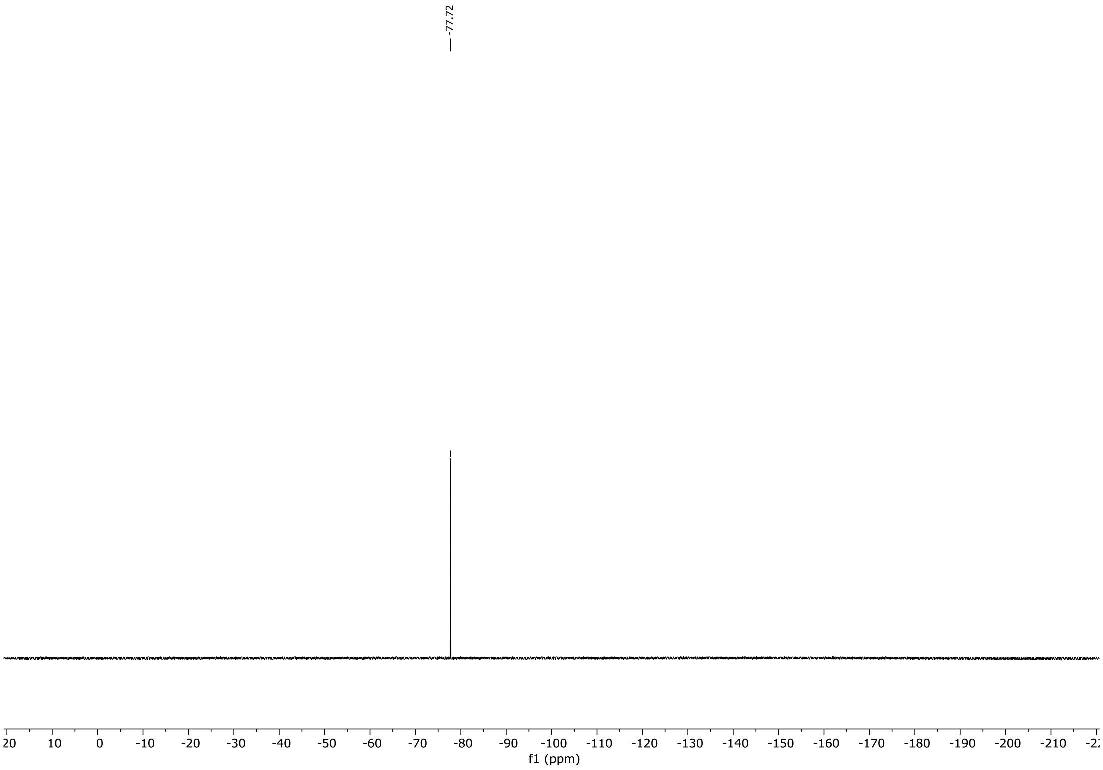

Figure S54: ^{19}F NMR spectrum (376 MHz) of compound **28** in DMSO-*d6*.

Synthesis of 2,4-di-tert-butyl-7-sulfodibenzo[b,d]iodol-5-ium trifluoromethanesulfonate (29)

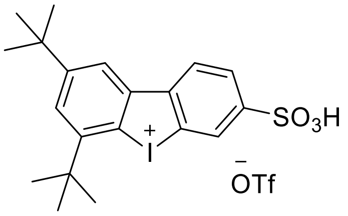

Chemical Formula: $C_{21}H_{24}F_3IO_6S_2$
Molecular Weight: 620,4367

Iodo compound **27** (4.32 g, 9.14 mmol, 1.0 eq.) was dissolved in DCM (0.2 M) and reacted with mCPBA (2.37 g, 13.7 mmol, 1.5 eq), cooled to 0 °C and HOTf (2.43 ml, 27.4 mmol, 3.0 eq.) was added. After stirring overnight and working up, the product was isolated as a white solid (4.33 g, 76%).

^{1}H NMR (300 MHz, DMSO-*d$_6$*): δ = 8.72 – 8.61 (m, 2H), 8.36 (d, *J* = 2.2 Hz, 1H), 8.01 (dd, *J* = 8.1, 1.6 Hz, 1H), 7.73 (d, *J* = 2.1 Hz, 1H), 1.52 (s, 9H), 1.42 (s, 9H) ppm.

^{13}C NMR (75 MHz, DMSO-*d$_6$*): δ = 154.0, 150.9, 150.0, 142.5, 141.7, 128.1, 127.8, 127.6, 126.4, 122.8, 121.6, 117.7, 35.5, 35.2, 31.0, 30.0 ppm.

^{19}F NMR (376 MHz, DMSO-*d$_6$*) δ = -75.80 ppm.

ATR-IR [cm^{-1}]: 3505.62 (s), 3447.76 (s), 2953.02 (m), 2873.94 (vs), 1478.44 (vs), 1465.9 (s), 1403.21 (s), 1367.53 (m), 1278.81 (vs), 1226.73 (w), 1166.93 (vw), 1138.00 (m), 1113.89 (vw), 1028.06 (vw), 1008.77 (w), 992.38 (m), 885.33 (m), 875.68 (m), 844.82 (m), 733.91 (m), 668.33 (w), 646.15 (m), 628.79 (m), 620.11 (w), 582.50 (m), 563.21 (m), 547.78 (m), 502.46 (s), 491.85 (s).

ESI-MS: m/z (+) = calc. 471.0 [M-OTf]+, found 471.1 [M-OTf]+
m/z (-) = calc. 148.9 [OTf]-, found 148.6 [OTf]-

CHNS [%]:calc.: C: 43.23, H: 4.93, S: 9.23 (+Et_2O)
found: C: 44.88, H: 4.49, S: 8.79 (+Et_2O)

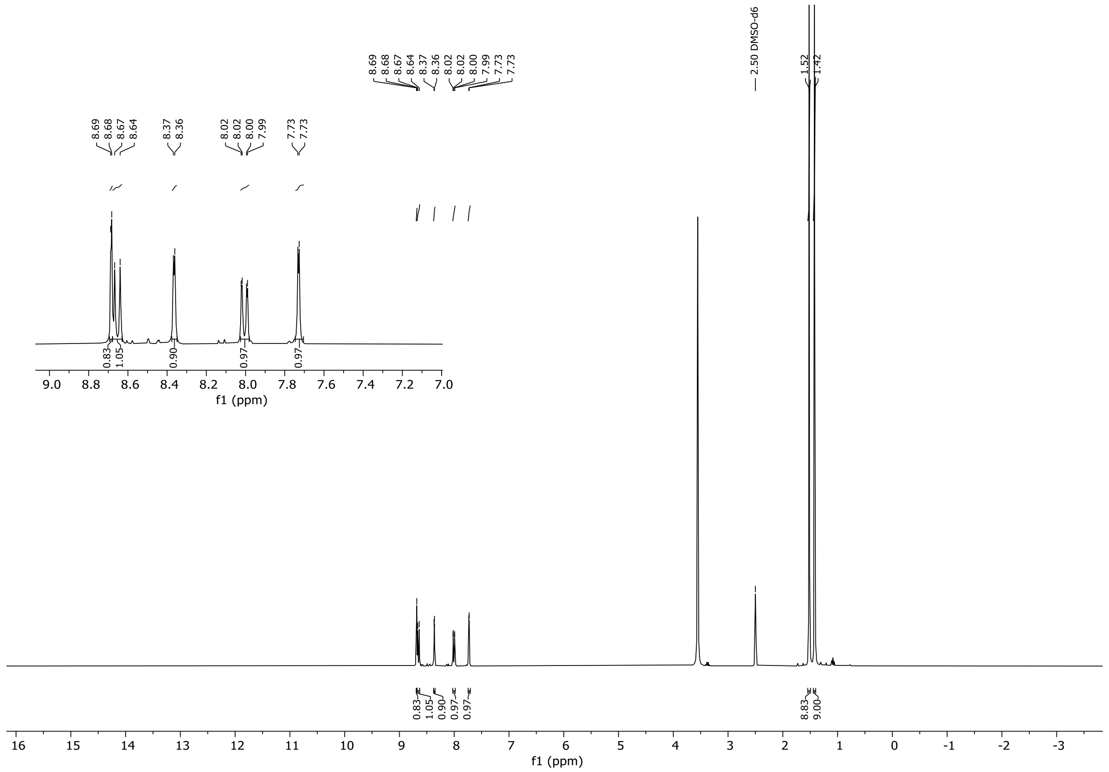

Figure S55: ^{1}H NMR spectrum (300 MHz) of compound **29** in DMSO-*d$_6$*.

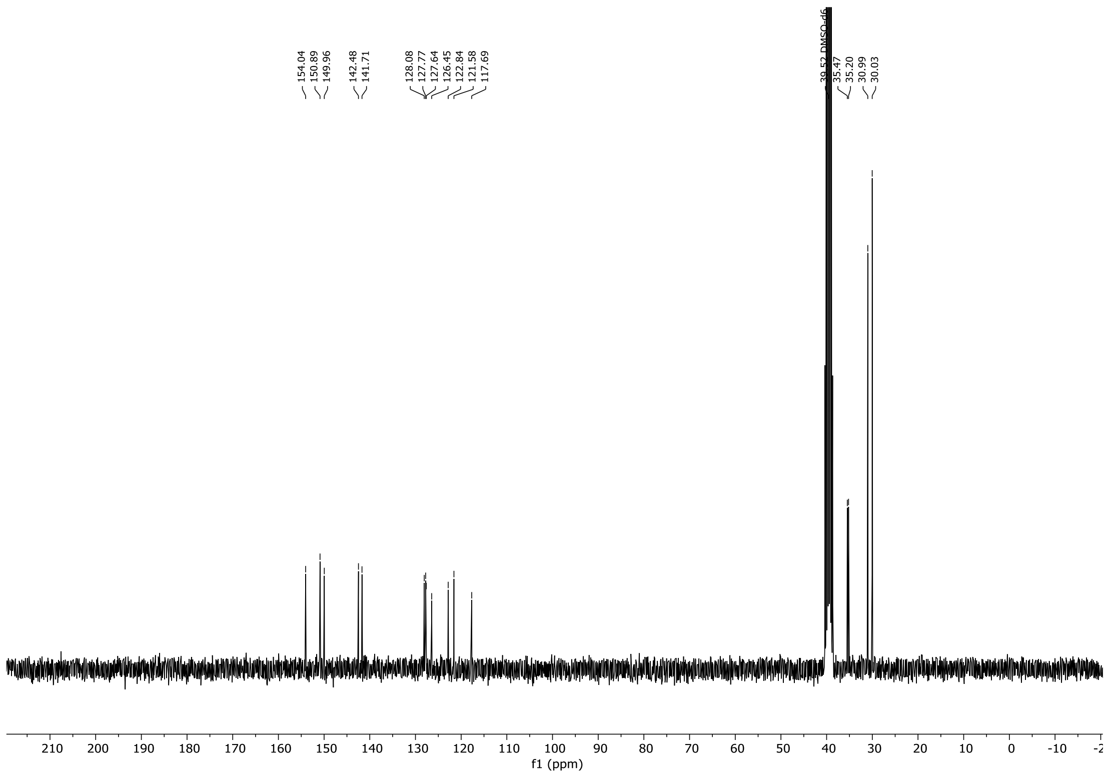

Figure S56: ^{13}C NMR spectrum (375 MHz) of compound **29** in DMSO-d_6.

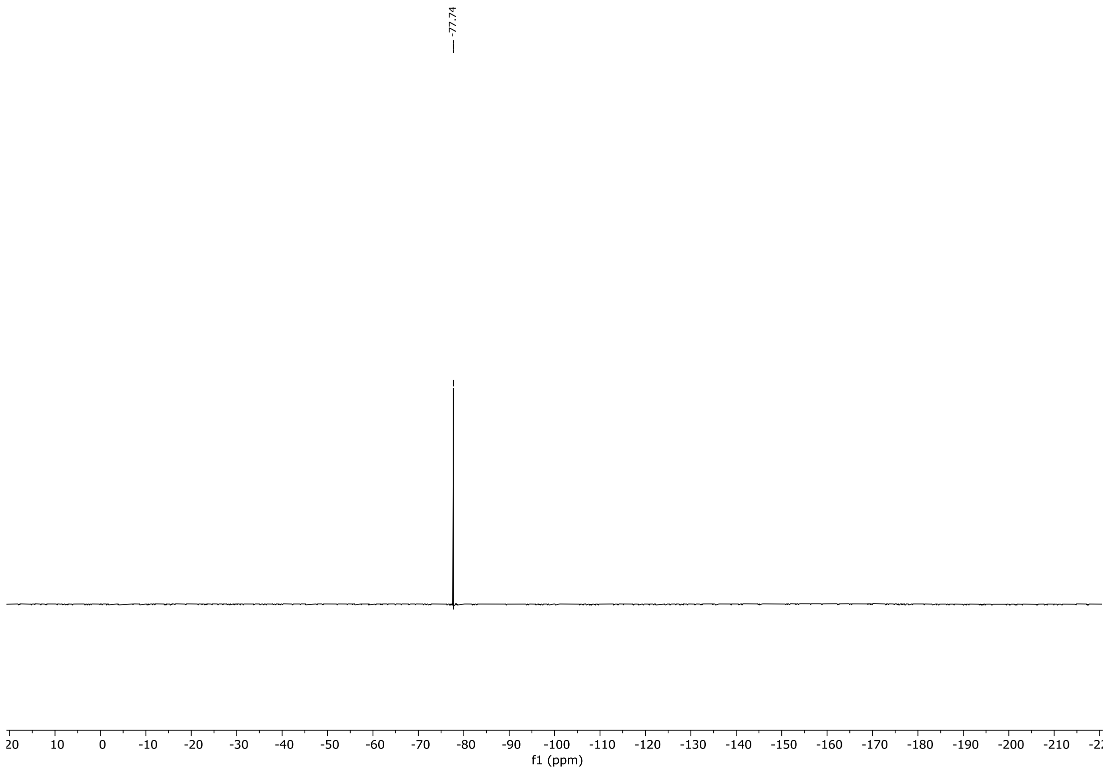

Figure S57: ^{19}F NMR spectrum (376 MHz) of compound **29** in DMSO-d_6.

General procedure to zwitterionic compounds 14 and 2

The iodolium salt (1.0 eq.) was suspended in pyridine (0.1 M) and stirred at r.t. over night. Then, the product was isolated via filtration and washed with water, EtOAc and DEE.

Synthesis of 6,8-dimethyldibenzo[b,d]iodol-5-ium-3-sulfonate (14)

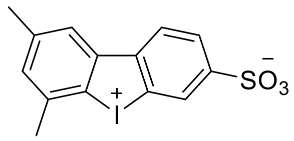

Chemical Formula: $C_{14}H_{11}IO_3S$
Molecular Weight: 386,2035

The iodolium salt **28** (170 mg, 0.317 mmol, 1.0 eq.) was suspended in pyridine (0.1 M). After stirring overnight and working up, the product was isolated as white solid (53.2 mg, 44%).

^{1}H NMR (400 MHz, DMSO-*d*$_6$): δ = 8.61 (d, *J* = 1.2 Hz, 1H), 8.40 (d, *J* = 8.1 Hz, 1H,), 8.16 (s, 1H), 8.00 (dd, *J* = 8.0, 1.2 Hz, 1H), 7.42 (s, 1H), 2.65 (s, 3H), 2.48 (s, 3H).[14]

ATR-IR [cm^{-1}]: 3502.73 (w), 3448.72 (w), 3101.61 (vw), 1645.16 (w), 1582.26 (w), 1458.18 (w), 1382.96 (m), 1367.53 (m), 1251.80 (w), 1220.94 (vs), 1176.58 (vs), 1145.72 (s), 1112.93 (vs), 1031.92 (vs), 1008.77 (s), 885.48 (w), 866.04 (m), 840.96 (s), 740.67 (m), 725.23 (vs), 661.58 (vs), 626.86 (vs), 607.58 (m), 594.07 (w), 551.61 (w), 522.58 (vw), 493.548 (w), 449.41 (m), 406.98 (m).

ESI-MS: m/z (-) = calc. 384.9 [M-H]-, found 384.9 [M-H]-
m/z (-) = calc. 386.9 [M+H]+, found 387.1 [M+H]+

CHNS [%]: calc.: C: 43.54, H: 2.87, N: 0.00, S: 8.30
found: C: 39.56, H: 3.52, N: 0.00, S: 7.25

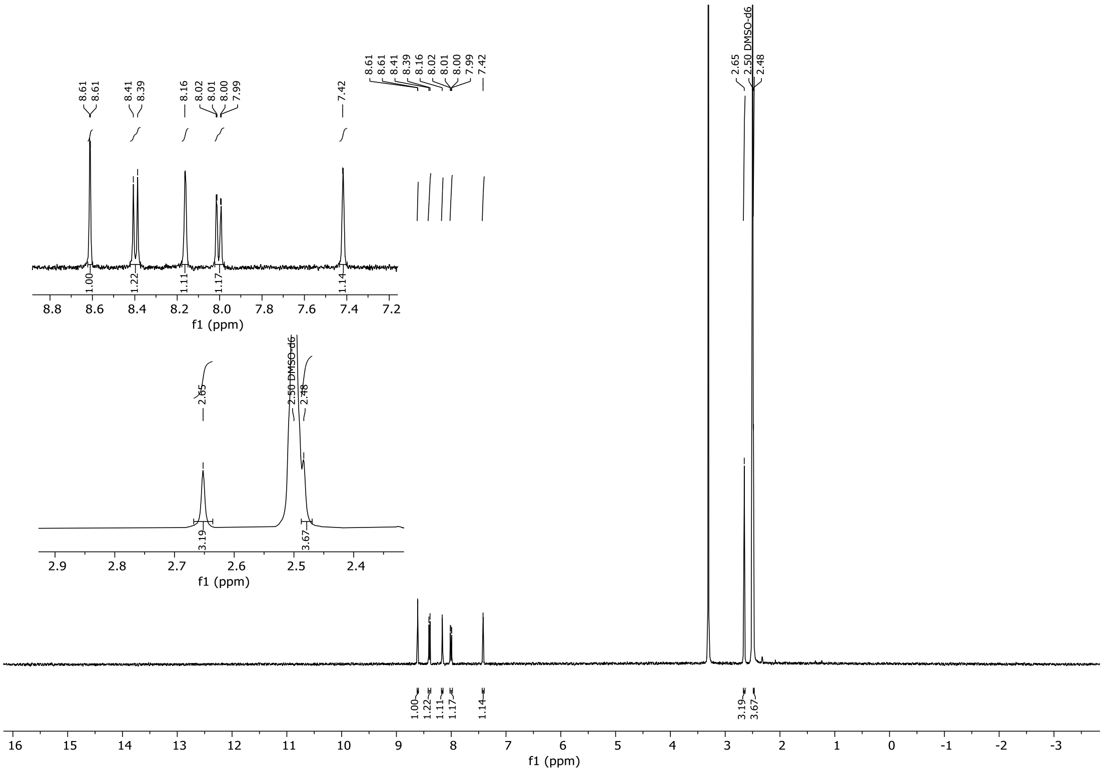

Figure S58: ^{1}H NMR spectrum (300 MHz) of compound **14** in DMSO-*d*$_6$.

Synthesis of 6,8-di-tert-butyldibenzo[b,d]iodol-5-ium-3-sulfonate (2)

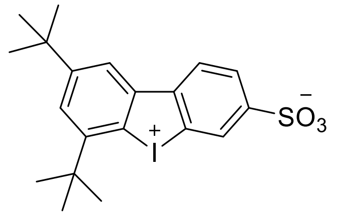

Chemical Formula: $C_{20}H_{23}IO_3S$
Molecular Weight: 470,3655

The iodolium salt **29** (3.0 g, 4.84 mmol, 1.0 eq.) was suspended in pyridine (0.1 M). After stirring overnight and working up, the product was isolated as white solid (2.28 g, quant.).

^{1}H NMR (300 MHz, DMSO-*d6*): δ = 8.69 (d, *J* = 1.6 Hz, 1H), 8.65 (d, *J* = 8.4 Hz, 1H), 8.36 (d, *J* = 2.2 Hz, 1H), 8.01 (dd, *J* = 8.2, 1.5 Hz, 1H), 7.73 (d, *J* = 2.1 Hz, 1H), 1.52 (s, 9H), 1.42 (s, 9H) ppm.

^{13}C NMR (75 MHz, DMSO-*d6*): δ = 154.1, 150.8, 150.0, 142.5, 141.8, 128.1, 127.8, 127.7, 126.5, 122.9, 121.6, 117.7, 35.5, 35.2, 31.0, 30.1 ppm.

ATR-IR [cm^{-1}]: 3507.55 (s), 3448.72 (s), 2953.02 (s), 1479.40 (vs), 1465.90 (s), 1403.21 (s), 1368.49 (m), 1359.82 (s), 1279.77 (vs), 1227.69 (w), 1167.90 (vw), 1138.00 (m), 1113.89 (vw), 1029.02 (vw), 1008.77 (w), 993.34 (s), 885.33 (m), 875.68 (m), 844.82 (w), 733.91 (m), 668.33 (w), 646.15 (m), 629.76 (w), 620.11 (w), 583.47 (m), 563.21 (m), 548.75 (w), 502.46 (s), 491.85 (s), 417.59 (vs).

ESI-MS: m/z (+) = calc. 488.1 [M+NH$_4$]+, found 488.1 [M+NH$_4$]+
calc. 963.1 [2M+Na]+, found 963.7 [2M+Na]+

CHNS [%]: calc.: C: 51.07, H: 4.93, N: 0.00, S: 6.82
found: C: 49.16, H: 4.94, N: 0.00, S: 6.34

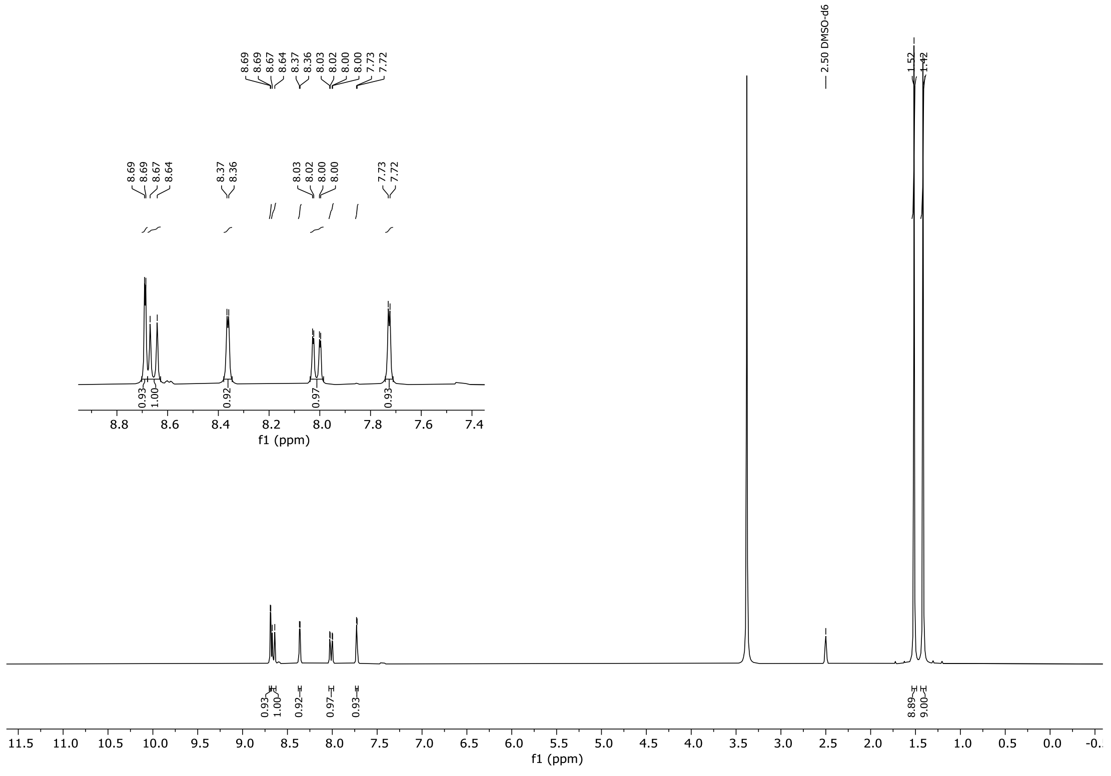

Figure S59: ^{1}H NMR spectrum (300 MHz) of compound **2** in DMSO-*d6*.

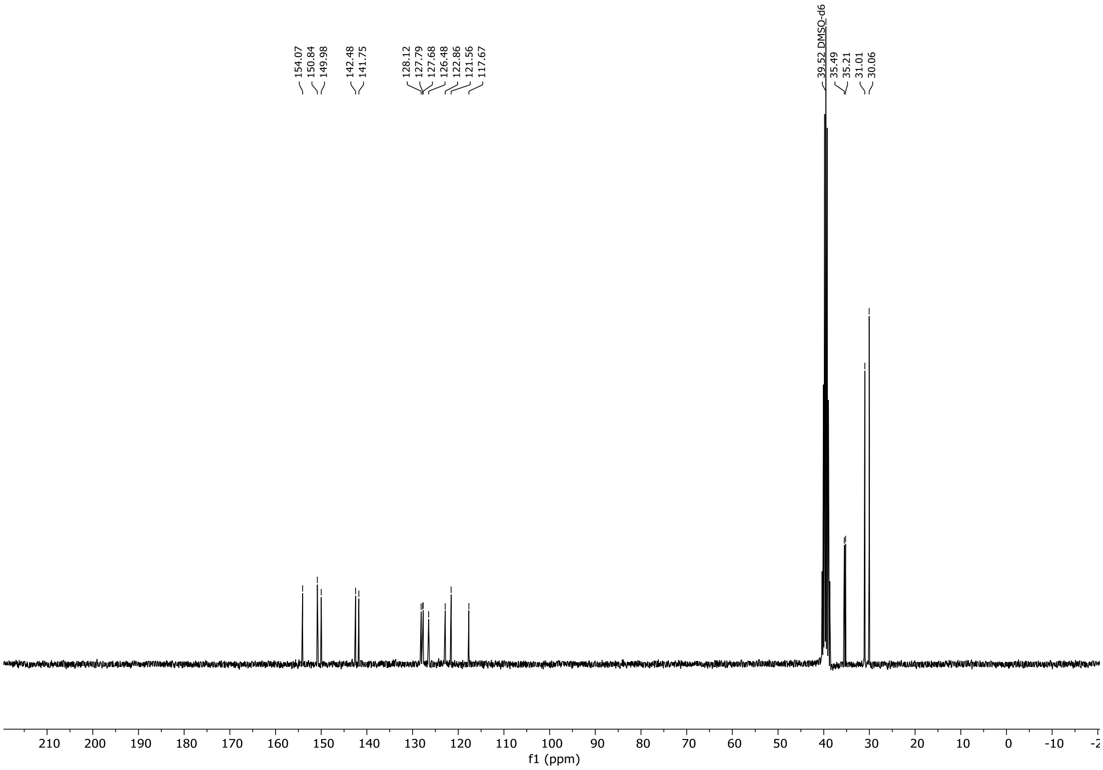

Figure S60: ^{13}C NMR spectrum (75 MHz) of compound **2** in DMSO-d_6.

Synthesis of 4-(tert-butyl)-2-nitro-1,1'-biphenyl (34)

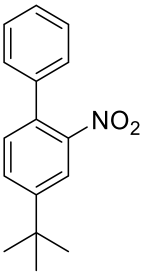

Chemical Formula: $C_{16}H_{17}NO_2$
Molecular Weight: 255,3170

Compound **33** (2.99 g, 11.6 mmol, 1.0 eq.) was suspended in a 1,4-dioxane-water mixture (2.5:1, 0.5 M) and the suspension was degassed by argon bubbling for 30 minutes. Phenylboronic acid (1.77 g, 14.5 mmol, 1.25 eq.), SPhos (194 mg, 0.464 mmol, 0.04 eq.), K_3PO_4 (4.81 g, 58.0 mmol, 5.0 eq.) and Pd_2dba_3 (107 mg, 0.116 mmol, 1 mol%) were added under argon and the mixture was stirred at 110 °C for 15 hours. After cooling down to r.t., the solvent was removed and resuspended in DEE. The organic phase was filtered over celite, then washed with water and brine, and finally dried over Na_2SO_4. The crude residue was purified by silica column chromatography with pentane and DEE (15:1) as eluent to obtain the desired product as orange oil (1.47 g, 50%).[18]

^{1}H NMR (300 MHz, $CDCl_3$): δ = 7.84 (d, *J* = 2.0 Hz, 1H), 7.63 (dd, *J* = 8.1, 2.1 Hz, 1H), 7.48 – 7.25 (m, 6H), 1.39 (s, 9H) ppm.

^{13}C NMR (75 MHz, $CDCl_3$): δ = 152.2, 149.3, 137.6, 133.5, 131.7, 129.6, 128.8, 128.1, 128.1, 121.1, 35.1, 31.2 ppm.

FD-MS: m/z (+) = calc.: 255.1259 [M]+, found: 255.1254 [M]+

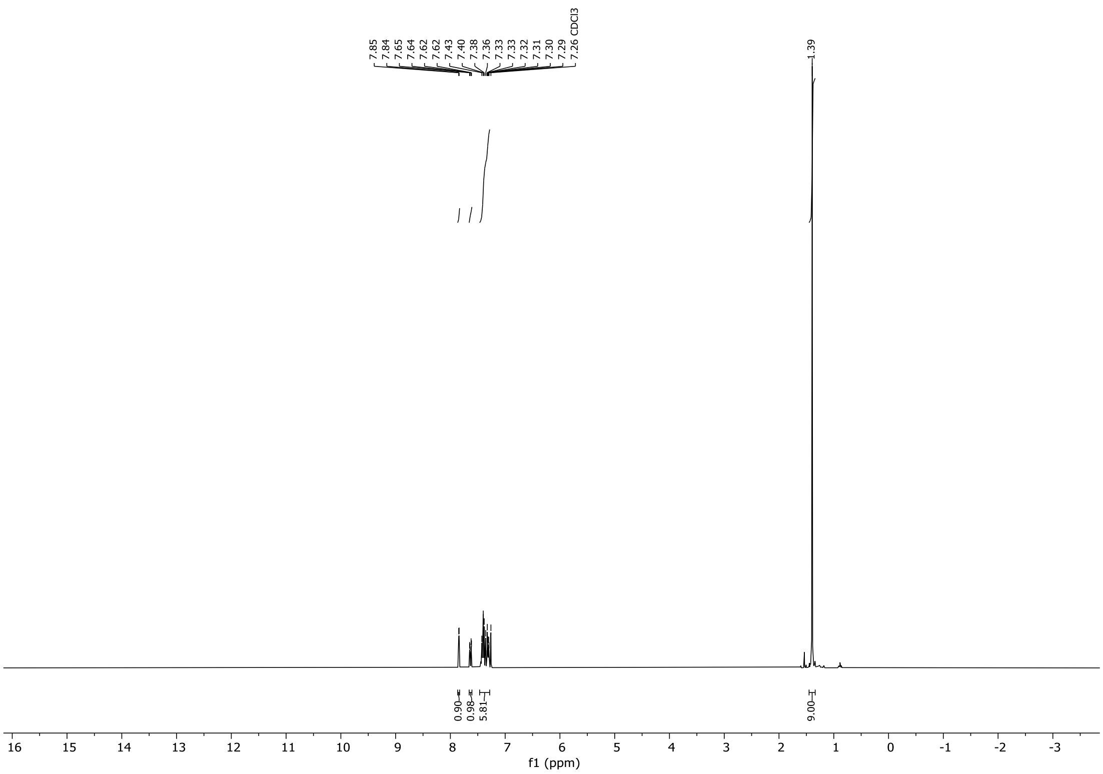

Figure S61: ^{1}H NMR spectrum (300 MHz) of compound **34** in $CDCl_3$.

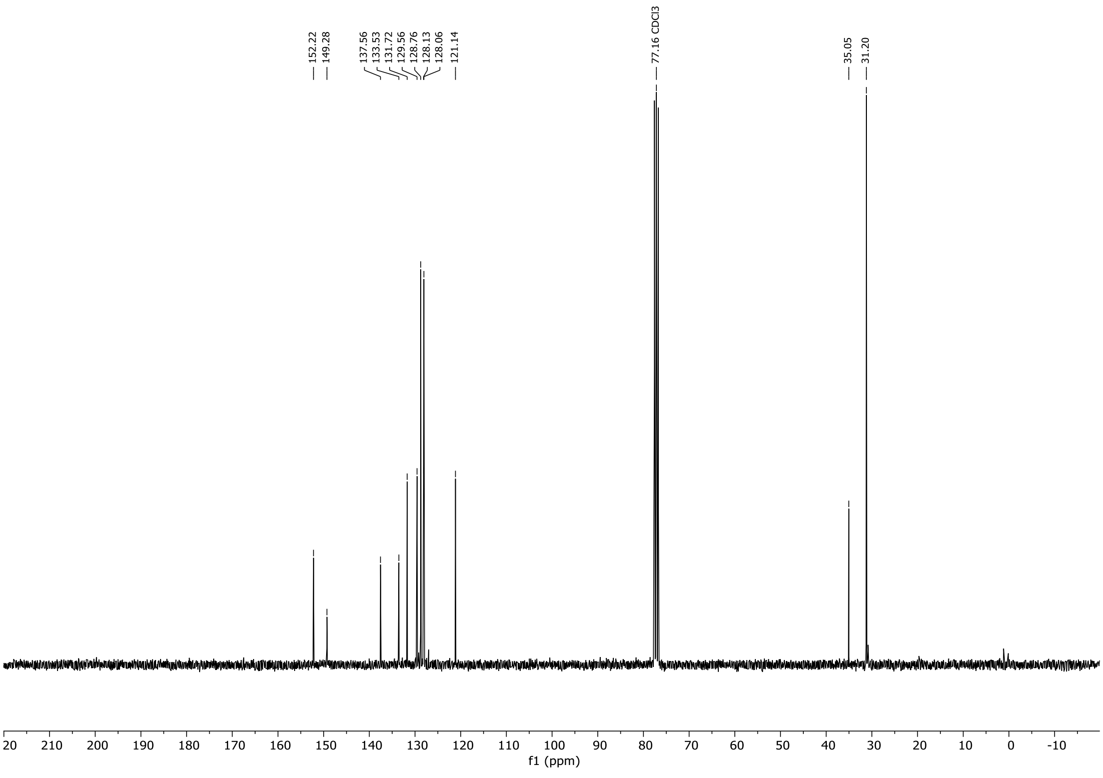

Figure S62: ^{13}C NMR spectrum (75 MHz) of compound **34** in $CDCl_3$.

Synthesis of 4-(tert-butyl)-[1,1'-biphenyl]-2-amine (35)

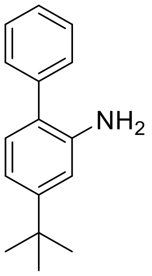

Chemical Formula: $C_{16}H_{19}N$
Molecular Weight: 225,3350

Compound **34** (1.57 g, 6.15 mmol, 1.0 eq.) were dissolved in 15 ml (0.4 M) EtOH and 400 mg (0.188 mmol) of 10% Pd/C were added. The reaction mixture was stirred at r.t. under a hydrogen atmosphere until complete conversion monitored via GC/MS. After completion, the mixture was filtered through a plug of celite and washed with EtOAc. The org. phase was washed with water and brine, dried over Na_2SO_4 and the solvent was removed in vacuo to obtain1.4 g (99%) of a white crystalline solid.[18]

^{1}H NMR (300 MHz, $CDCl_3$): δ = 7.51 – 7.40 (m, 4H), 7.37 – 7.29 (m, 1H), 7.09 (d, *J* = 8.0 Hz, 1H), 6.88 (ddd, *J* = 8.0, 1.9, 0.7 Hz, 1H), 6.82 (d, *J* = 1.9 Hz, 1H), 1.34 (s, 9H) ppm.

^{13}C NMR (75 MHz, $CDCl_3$): δ = 151.9, 143.1, 139.7, 130.3, 129.3, 128.9, 127.1, 125.2, 116.2, 113.1, 34.6, 31.5 ppm.

FD-MS: m/z (+) = calc.: 225.1517 [M]+, found: 225.1553 [M]+

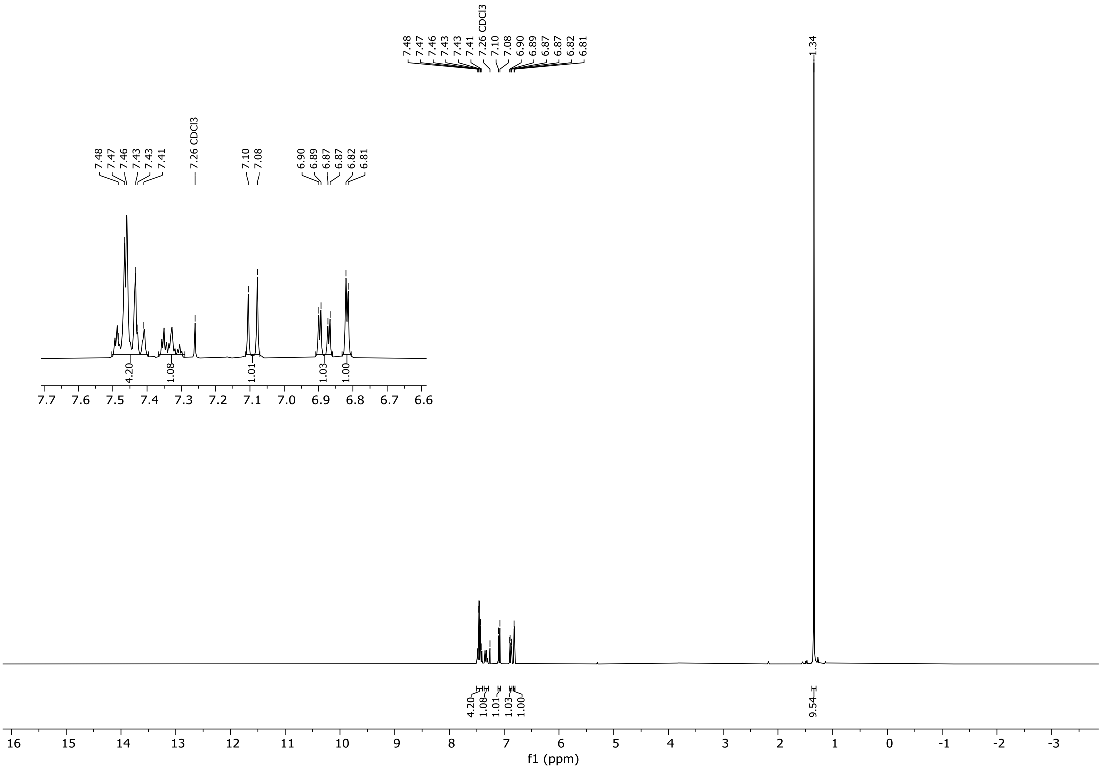

Figure S63: ^{1}H NMR spectrum (300 MHz) of compound **35** in $CDCl_3$.

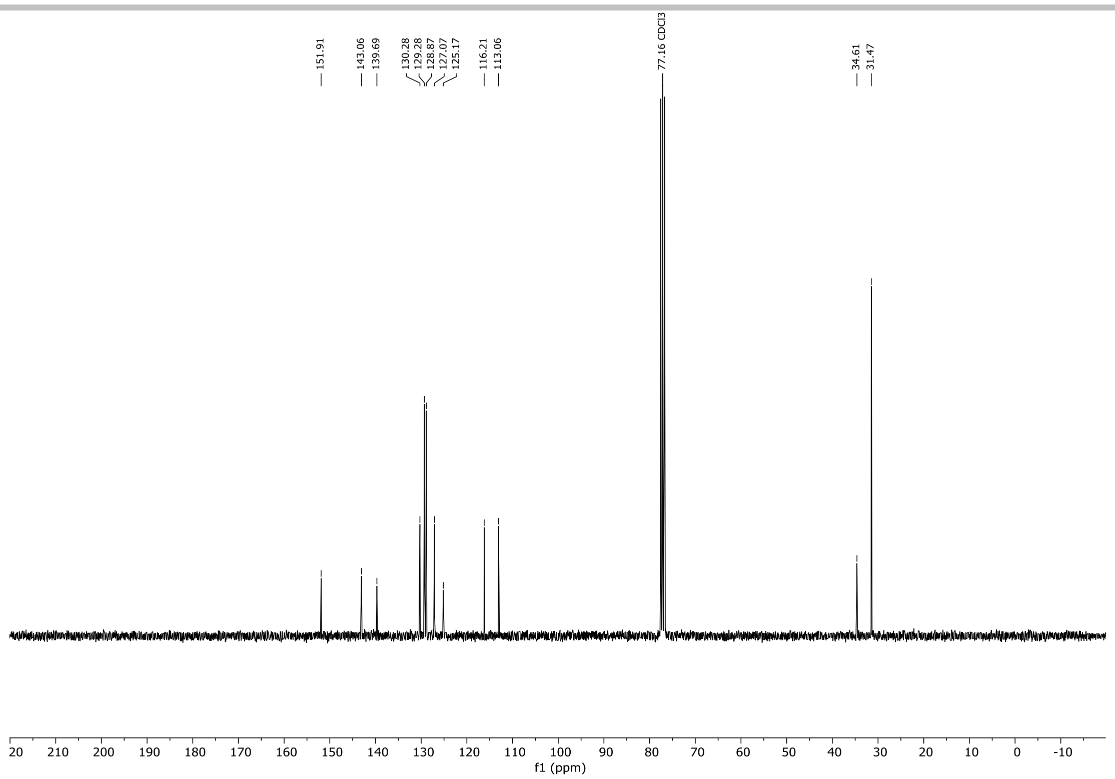

Figure S64: ^{13}C NMR spectrum (75 MHz) of compound **35** in $CDCl_3$.

Synthesis of 4-(tert-butyl)-2-iodo-1,1'-biphenyl (36)

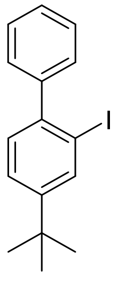

Chemical Formula: $C_{16}H_{17}I$
Molecular Weight: 336,2165

The reaction follows the procedure reported by Samanta et al.[17] Amine **35** (1.27 g, 5.64 mmol, 1.0 eq) was dissolved in 38 ml ACN (0.15 M) and 3.35 g (16.9 mmol, 3.0 eq) of pTsOH x H_2O were added at r.t forming a suspension. After stirring for 15 min, the reaction mixture was cooled to 0 °C and 778 mg (11.3 mmol, 2 eq) of sodium nitrite, dissolved in small amounts of water, were slowly added. After complete addition, 2.34 g (14.1 mmol, 2.5 eq.) of potassium iodide, dissolved in small amounts of water, were added dropwise to the obtained solution and stirred at r.t overnight. A saturated solution of $NaHCO_3$ (until pH = 9-10) and sodium sulfite were added, and the aq. phase extracted 3 times with 50 ml diethyl ether. The combined org. phases were washed with brine and dried over Na_2SO_4. The solvent was removed in vacuo and the crude compound purified via column chromatography using silica gel and pentane as eluent to obtain 1.8 g (95%) of an off-white solid. The obtained NMR spectra are in agreement with those reported in literature.[18]

^{1}H-NMR (300 MHz, $CDCl_3$): δ = 7.99 (d, J = 2.0 Hz, 1H), 7.49 – 7.36 (m, 6H), 7.29 (s, 1H), 1.39 (s, 9H) ppm.

^{13}C-NMR (75 MHz, $CDCl_3$): δ = 152.3, 144.2, 143.6, 136.7, 129.8, 129.5, 128.0, 127.6, 125.5, 98.9, 34.6, 31.4 ppm.

ESI-MS: m/z (-) = calc. 167.0 [M-2H]2-, found 167.0 [M-2H]2-

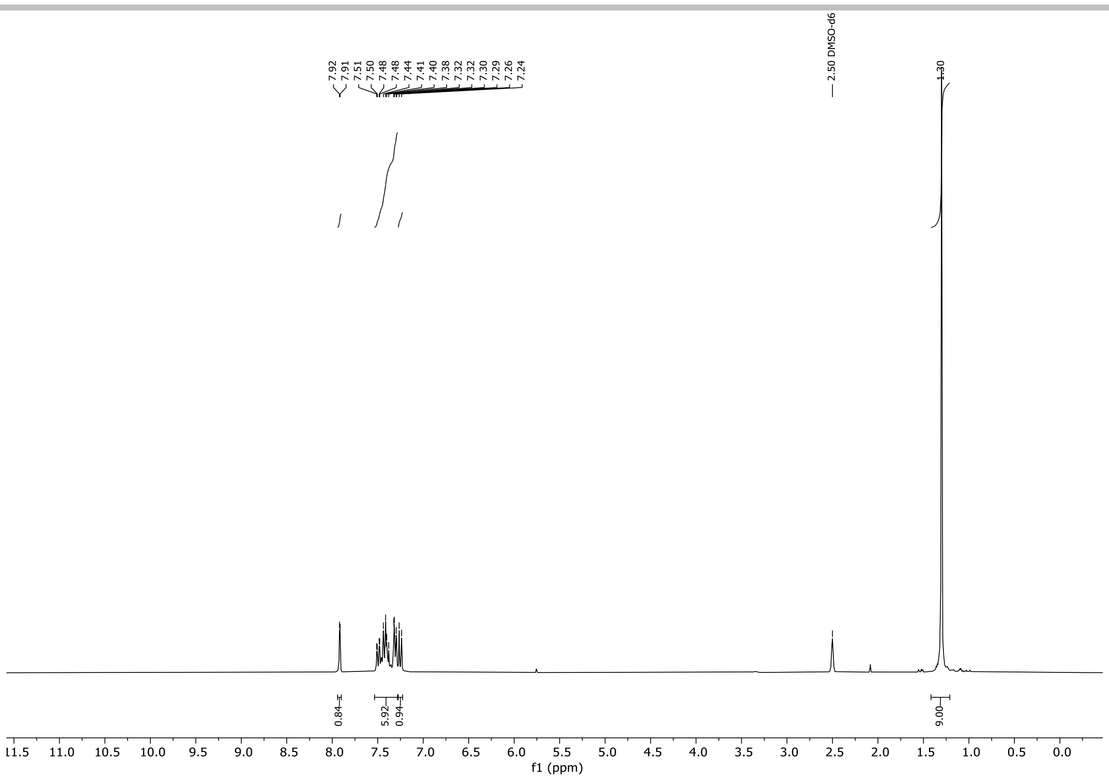

Figure S65: ^{1}H NMR spectrum (300 MHz) of compound **36** in $CDCl_3$.

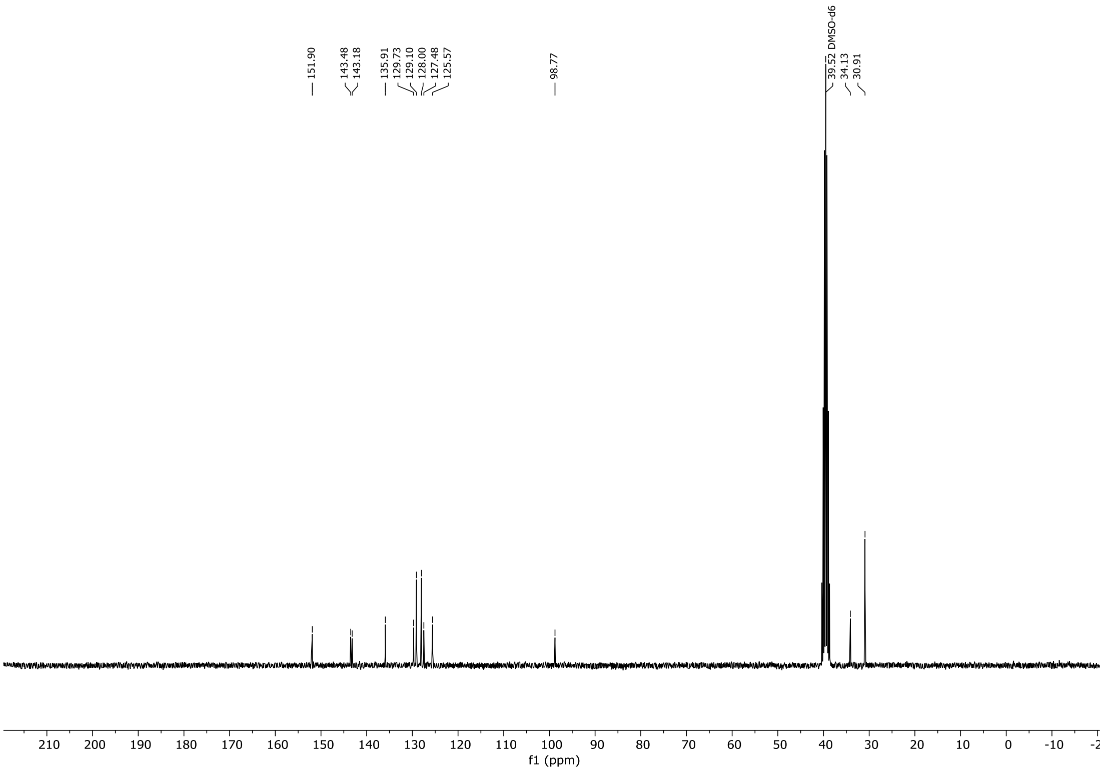

Figure S66: ^{13}C NMR spectrum (75 MHz) of compound **36** in $CDCl_3$.

Synthesis of 4'-(tert-butyl)-2'-iodo-[1,1'-biphenyl]-4-sulfonic acid (37)

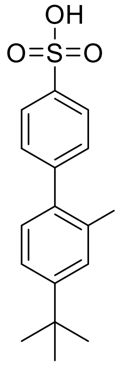

Chemical Formula: $C_{16}H_{17}IO_3S$
Molecular Weight: 416,2735

Chlorosulfonic acid (0.55 ml, 8.2 mmol, 4.0 eq) was dissolved in 10 ml chloroform and 690 mg (2.05 mmol, 1.0 eq) of compound **36** dissolved in 5 ml chloroform was added dropwise at r.t. The deep red solution was stirred for 3 h and poured onto ice. The formed emulsion was cleared by adding small amounts of ethanol. The aq. phase was extracted with DCM, dried over Na_2SO_4 and the solvent removed in vacuo. Finally, 740 mg (87%) of a sticky brown oil were obtained and used without further purification.

^{1}H-NMR (300 MHz, DMSO-*d*$_6$): δ = 7.91 (d, J = 2.0 Hz, 1H), 7.65 (d, J = 7.9 Hz, 2H), 7.50 (dd, J = 8.1, 2.0 Hz, 1H), 7.26 (dd, J = 16.6, 8.0 Hz, 3H), 1.29 (s, 9H) ppm.

^{13}C-NMR (75 MHz, DMSO-*d*$_6$): δ = 152.5, 147.4, 144.1, 143.2, 136.4, 130.2, 129.1, 126.1, 125.7, 99.7, 34.6, 31.4 ppm.

ATR-IR [cm^{-1}]: 2935 (b), 1702 (vb), 1599 (vw), 1476 (w), 1379 (w), 1174 (b), 1125 (m), 1037 (vw), 1033 (w), 1010 8w), 1001 (m), 994 (b), 824 (m), 732 (m), 636 (m), 606 (m), 563 (s), 512 (w).

ESI-MS: m/z (-) = calc: 414.98 [M-H]-, found: 415.2 [M-H]-

CHNS [%]: calc.: C: 46.17, H: 4.12, S: 7.70
found: C: 43.23, H: 4.69, S: 7.84

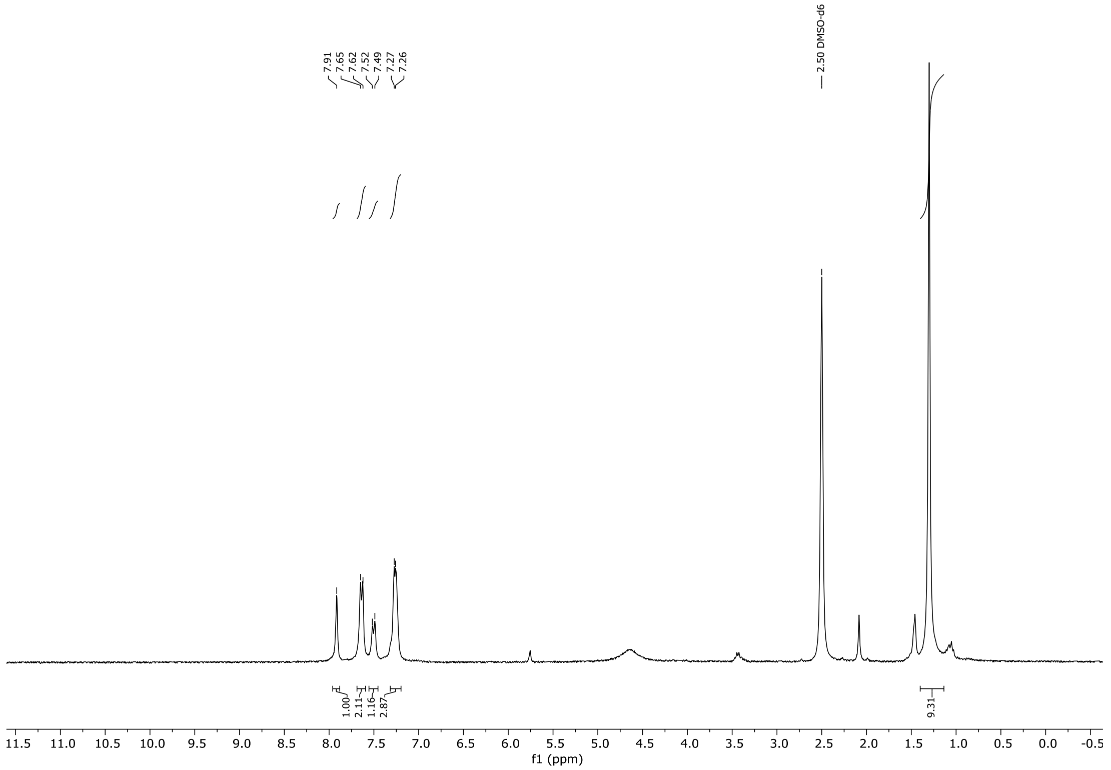

Figure S67: ^{1}H NMR spectrum (300 MHz) of compound **37** in DMSO-*d*$_6$.

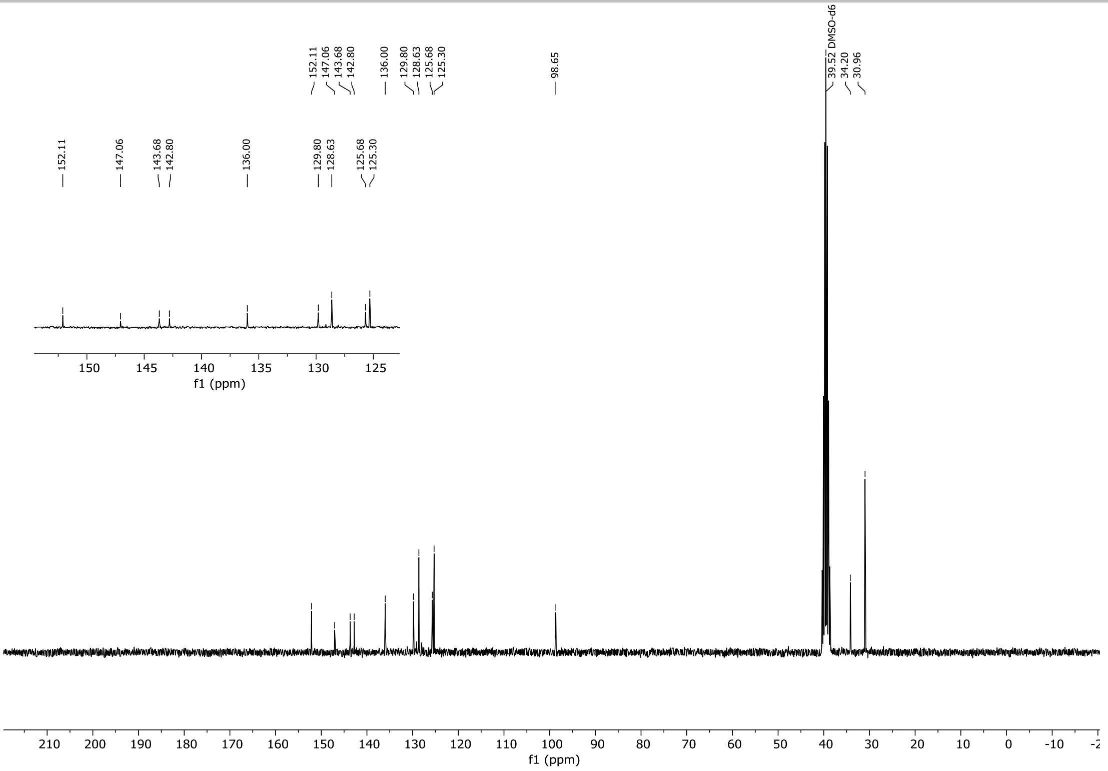

Figure S68: ^{13}C NMR spectrum (75 MHz) of compound **37** in DMSO-*d6*.

Synthesis of 3-(tert-butyl)-7-sulfodibenzo[b,d]iodol-5-ium trifluoromethanesulfonate (38)

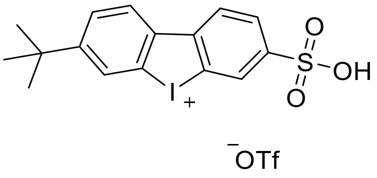

Chemical Formula: $C_{17}H_{16}F_3IO_6S_2$
Molecular Weight: 564,3287

Compound **37** (122 mg, 0.293 mmol) was suspended in DCM (0.6 ml, 0.5 M) and cooled to 0 °C. Afterwards, 98.5 mg (77% active oxidant, 0.439 mmol, 1.5 eq) of *meta*-chloroperoxybenzoic acid (*m*CPBA) were added and stirred for 30 min at this temperature. Triflic acid (HOTf) (78 µl, 0.879 mmol, 3 eq) was slowly added and the reaction mixture was warmed up to r.t and stirred overnight. The solvent was removed in vacuo and the resulting solid suspended in 5 ml diethyl ether and stirred for 15 min. The precipitate was filtered and the solid washed with small amounts of diethyl ether. Finally, 82 mg (50%) of an off-white solid were obtained.

^{1}H-NMR (300 MHz, DMSO-*d*$_6$): δ =8.49 (d, J = 1.5 Hz, 1H), 8.39 (dd, J = 8.3, 6.8 Hz, 2H), 8.23 (d, J = 1.8 Hz, 1H), 7.98 (dd, J = 8.0, 1.6 Hz, 1H), 7.91 (dd, J = 8.3, 1.8 Hz, 1H), 1.38 (s, 9H) ppm.

^{13}C-NMR (75 MHz, DMSO-*d*$_6$): δ =) 154.5, 150.3, 141.5, 138.7, 128.4, 127.7, 126.8, 126.3, 122.4, 121.1, 35.5, 30.9 ppm.

^{19}F-NMR (235 MHz, DMSO-*d*$_6$): δ = -77.75 ppm.

ATR-IR [cm^{-1}]: 3093 (vw), 2971 (vw), 1596 (w), 1391 (w), 138 (w), 1271 (m), 1246 (m), 1221 (s), 1171 (s) 1113 (w), 1085 (w), 1023 (s), 830 (m), 728 (wv), 699 (m), 650 (m), 637 (s), 617 (s), 606 (m), 582 (s), 544 (m), 517 (s).
FD-MS: m/z (+) = calc.: 414.9860 [M]+, found: 414.9852 [M]+

CHNS [%] calc.: C: 36.18, H: 2.86, S: 11.36
found: C: 36.15, H: 2.85, S: 10.46

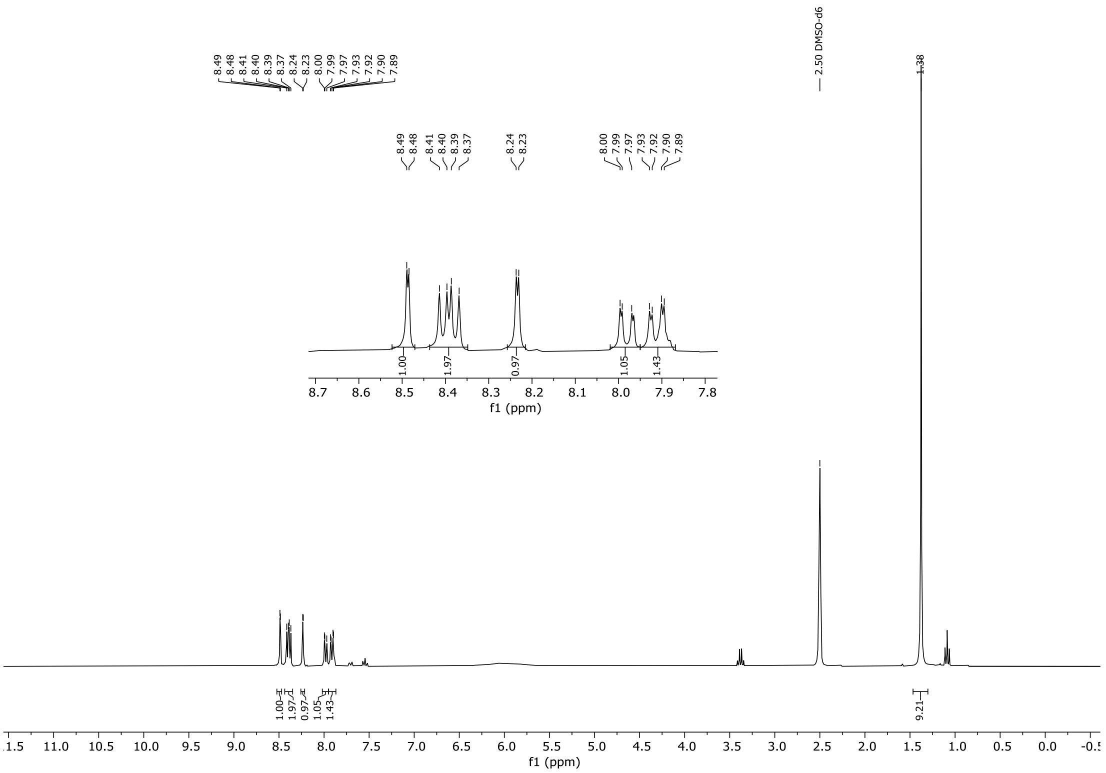

Figure S69: ^{1}H NMR spectrum (300 MHz) of compound **38** in DMSO-d_6.

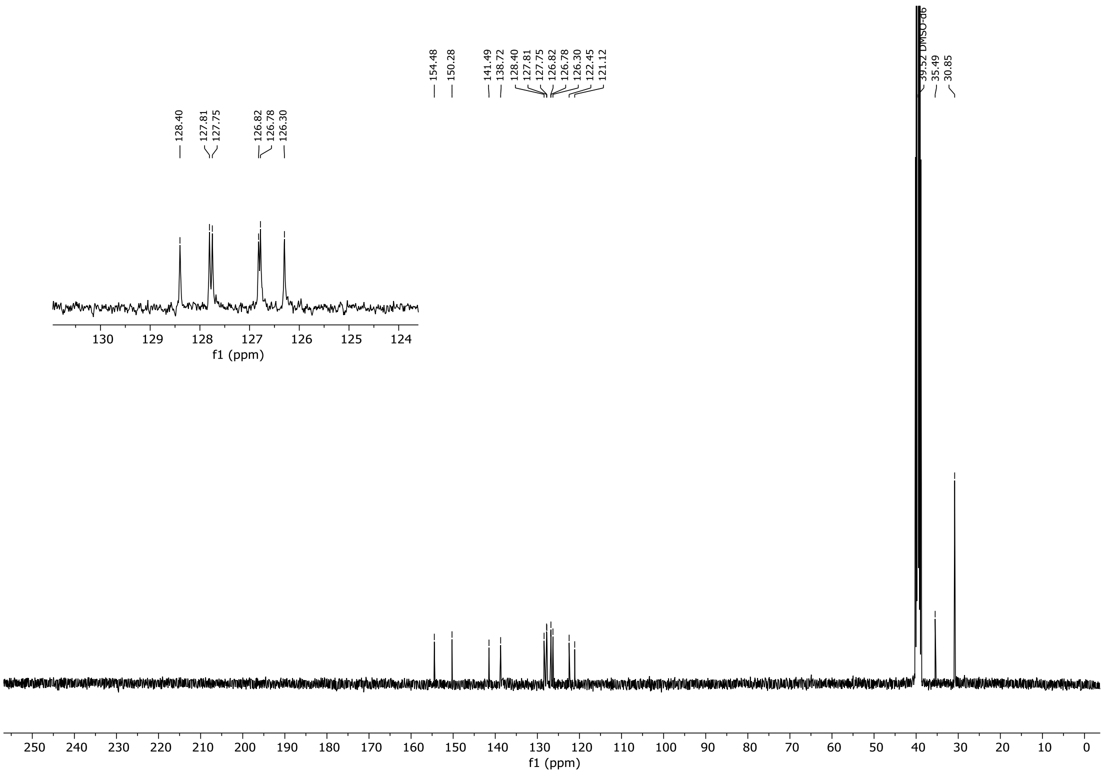

Figure S70: ^{13}C NMR spectrum (75 MHz) of compound **38** in DMSO-d_6.

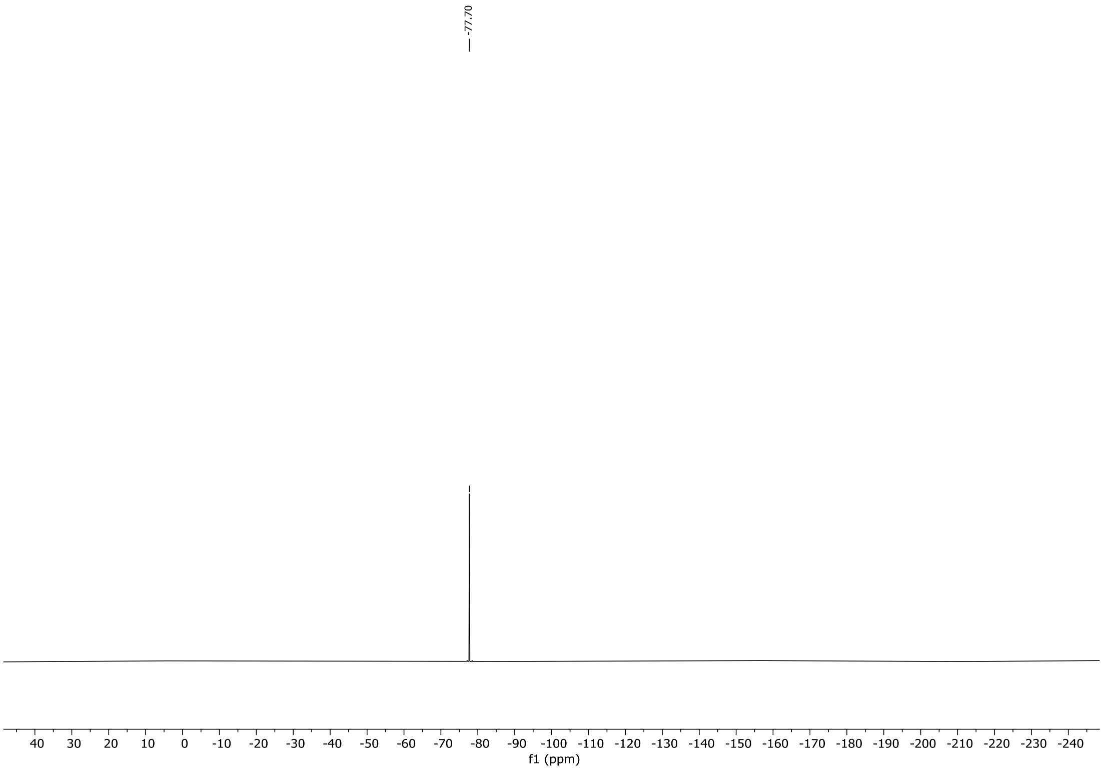

Figure S71: ^{19}F NMR spectrum (235 MHz) of compound **38** in DMSO-d_6.

Synthesis of 7-(tert-butyl)dibenzo[b,d]iodol-5-ium-3-sulfonate (1)

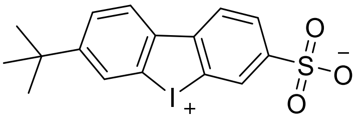

Chemical Formula: $C_{16}H_{15}IO_3S$
Molecular Weight: 414,2575

The iodolium salt **38** (100 mg, 0.177 mmol, 1 eq) was suspended in 2 ml pyridine and stirred at r.t overnight. (Note: It stayed a suspension over the whole reaction time.) The solid was filtered and washed several times with small amounts of methanol and DCM to remove both the pyridine salt and solvent traces of pyridine. After drying under high vacuum, 73 mg (99%) of a white solid were obtained.

^{1}H-NMR (300 MHz, DMSO-d_6): δ = 8.50 (s, 1H), 8.38 (t, J = 8.5 Hz, 2H), 8.28 (s, 1H), 7.94 (dd, J = 36.2, 8.2 Hz, 2H), 1.37 (s, 9H) ppm.

^{13}C-NMR (75 MHz, DMSO-d_6): δ = 154.9, 150.7, 141.9, 139.2, 128.8, 128.2, 127.4, 126.7, 123.0, 121.9, 36.0, 31.3 ppm.

ATR-IR [cm^{-1}]: 3095 (vw), 2963 (vw), 1597 (w), 1376 (w), 1228 (m), 1173 (s), 1115 (m), 1029 (m), 1017 (m), 841 (w), 825 (w), 733 (w), 702 (w), 653 (s), 640 (s), 579 (m), 570 (m), 556 (m), 516 (m), 474 (w).

HR-Mass: m/z (+) = calc.: 413.98 [M]+, found: 414.98 [M]+

CHNS [%]: calc.: C: 41.07, H: 3.29, N: 2.18, S: 9.97 (+PyHOTf)
found: C: 41.01, H: 3.28, N: 2.26, S: 9.84 (+PyHOTf)

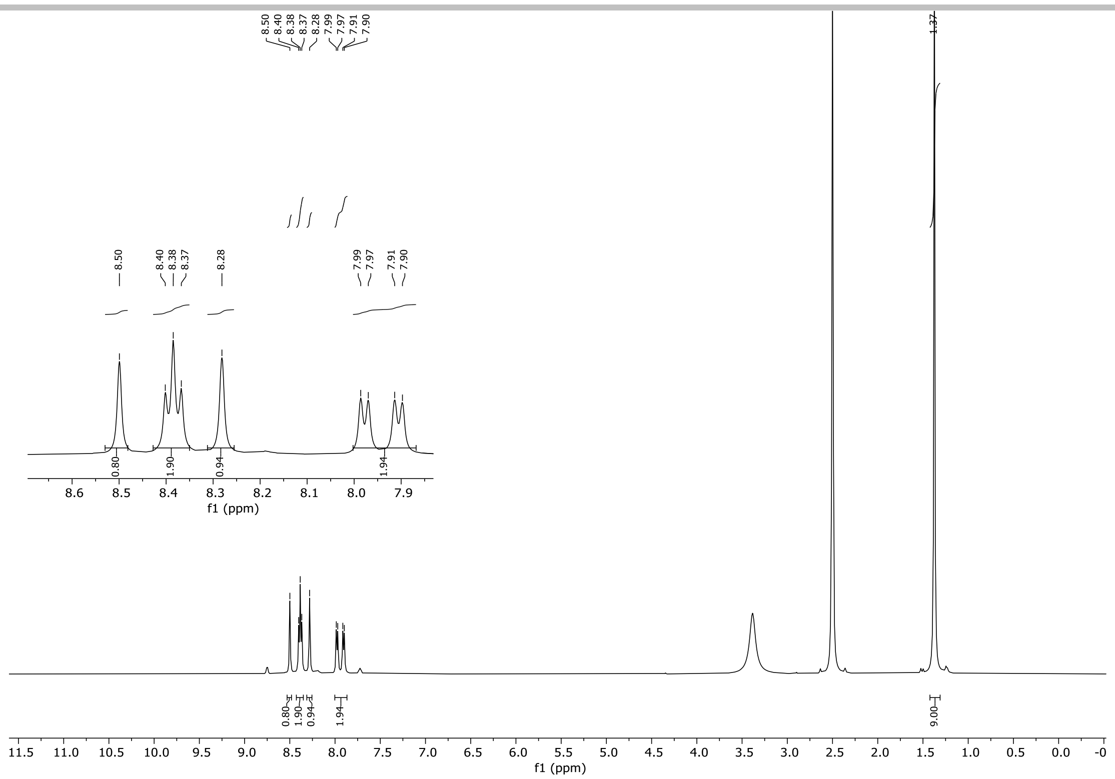

Figure S72: ^{1}H NMR spectrum (300 MHz) of compound **1** in DMSO-d_6.

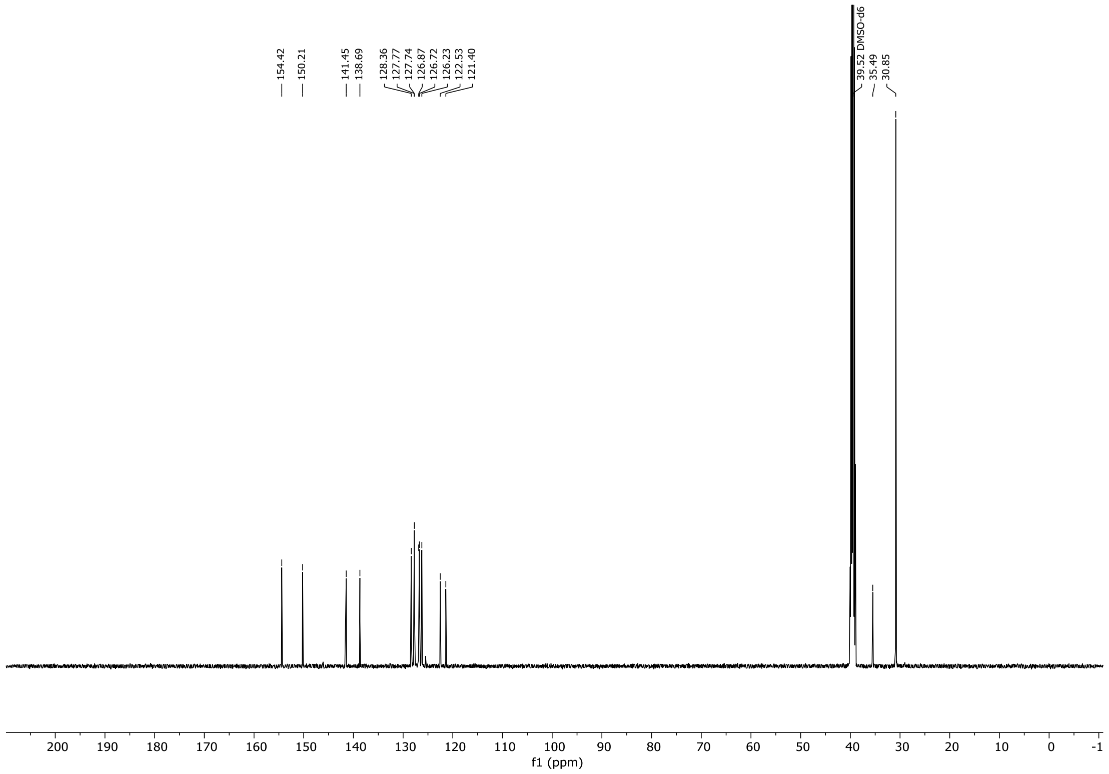

Figure S73: ^{13}C NMR spectrum (75 MHz) of compound **1** in DMSO-d_6.

Synthesis of (4-(2-ethylhexyl)phenyl)trimethylsilane (S9)

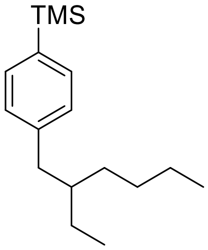

Chemical Formula: $C_{17}H_{30}Si$
Molecular Weight: 262,5120

Under an argon atmosphere and in an flame-dried Schlenk flask, (4-bromophenyl)tetramethylsilane (0.997 eq, 4.0 g, 17.5 mmol, 1.0 eq.) was dissolved in dry THF (0.5 M) and cooled to -78 °C. t-Butyllithium (2 eq, 18.4 ml, 35 mmol, 1.9 mol/l in hexane, 2.0 eq.) was added and the mixture was stirred at this temperature for one hour. After that, 2-ethylhexylbromide (3.38 g, 17.5 mmol, 1.0 eq.) was added and the reaction mixture was allowed to warm to r.t. overnight while stirring. The next day, water and DEE were added and the aqueous phase was extracted with DEE (2 x 75 mL). The combined organic phases were dried over $MgSO_4$, and the solvent was removed. After drying in high vacuum while heating to 80 °C, the product was obtained as a yellow oil (3.44 g, 75%).

^{1}H-NMR (300 MHz, $CDCl_3$): δ=7.45 (d, J = 8.1 Hz, 2H), 7.16 (d, J = 8.0 Hz, 2H), 2.63 – 2.39 (m, 2H), 1.60 (q, J = 5.7 Hz, 1H), 1.42 – 1.18 (m, 8H), 0.96 – 0.84 (m, 6H), 0.28 (s, 9H) ppm.

^{13}C NMR (75 MHz, $CDCl_3$): δ = 142.7, 137.1, 133.3, 128.8, 41.1, 40.2, 32.5, 29.0, 25.5, 23.2, 14.3, 10.9, -0.9 ppm.

ATR-IR [cm^{-1}]: 2955.91 (m), 2925.53 (m), 1600.92 (s), 1458.18 (s), 1395.98 (s), 1378.62 (s), 1246.98 (w), 1108.59 (m), 850.61 (w), 833.25 (vw), 751.76 (w), 726.68 (m), 661.10 (m), 661.10 (m), 614.81 (s), 531.39 (s).

ESI-MS: m/z (-) = calc.: 247.2 [M-Me]-, found: 248.8 [M-Me]-[19]

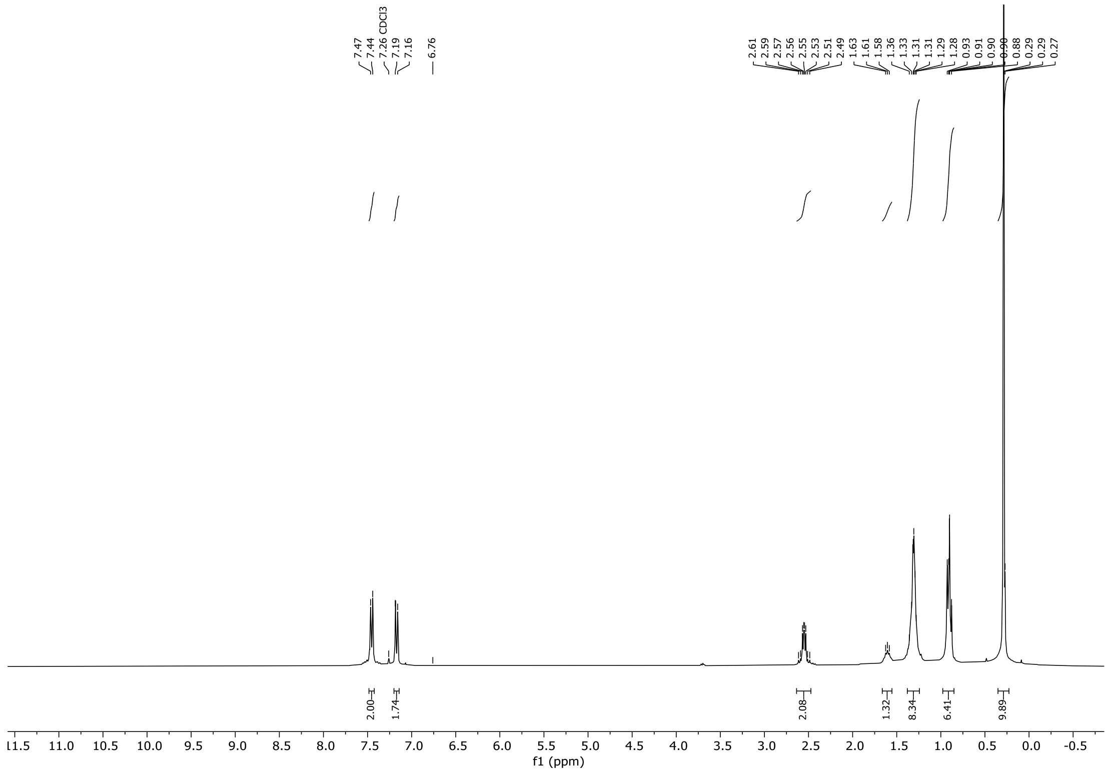

Figure S74: ^{1}H NMR spectrum (300 MHz) of compound **S9** in $CDCl_3$.

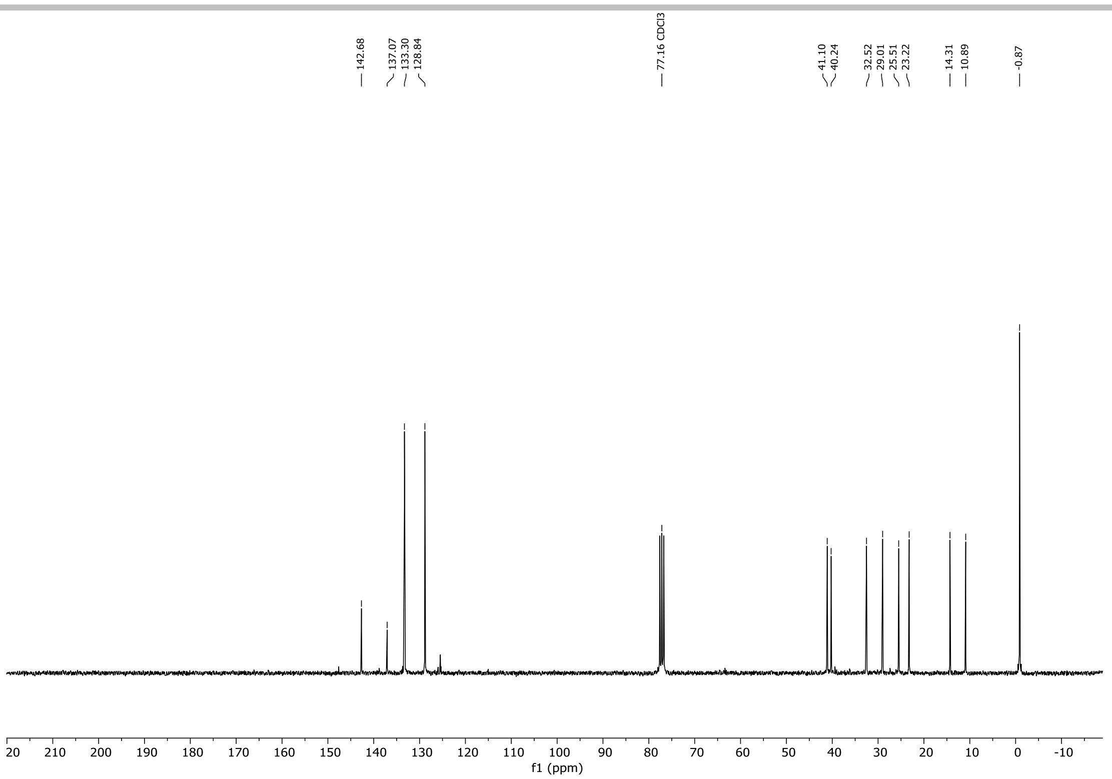

Figure S75: ^{13}C NMR spectrum (75 MHz) of compound **S9** in $CDCl_3$.

Synthesis of 1-(2-ethylhexyl)-4-iodobenzene (S10)

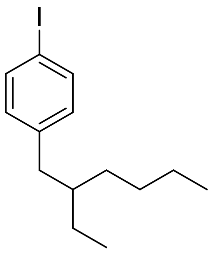

Chemical Formula: $C_{14}H_{21}I$
Molecular Weight: 316,2265

Under inert conditions, compound **S9** (2.1 g, 8.0 mmol, 1.0 eq.) was dissolved in dry DCM (0.1 M) at room temperature and Iodo monochloride (16 ml, 16.0 mmol, 1 M in DCM, 2.0 eq.) was added dropwise. The reaction mixture was stirred for 3 hours at 40 °C before it was quenched with a 100 ml of an aq. sat. solution of Na_2SO_3. The organic phase was washed with an aq. sat. $NaHCO_3$ solution and water, and was dried afterwards over Na_2SO_4. The solvent was removed, and the remaining oil was purified by vacuum distillation (1.2*10-2 mbar) at 140 °C to isolate a red oil. The oil was redissolved in EtAOc and washed with an aq. sat. solution of Na_2SO_4. After removal of the solvent, 1.27 g (50%) of iodo compound **S10** as a colorless oil were obtained.

^{1}H NMR (300 MHz, $CDCl_3$): δ = 7.58 (d, *J* = 8.3 Hz, 2H), 6.90 (d, *J* = 8.3 Hz, 2H), 2.47 (d, *J* = 7.1 Hz, 2H), 1.54 (dq, *J* = 13.0, 6.4 Hz, 1H), 1.36 – 1.15 (m, 8H), 0.87 (td, *J* = 7.4, 2.2 Hz, 6H) ppm.

^{13}C NMR (75 MHz, $CDCl_3$): δ = 141.6, 137.2, 131.4, 90.7, 41.1, 39.7, 32.4, 28.9, 25.5, 23.2, 14.3, 10.9 ppm.

ATR-IR [cm^{-1}]: 2956.39 (m), 2923.60 (w), 2870.08 (vs), 2855.61 (m), 1483.74 (w), 1464.94 (s), 1458.18 (m), 1456.74 (m), 1455.29 (s), 1399.36 (vs), 1060.85 (vs), 1005.88 (vw), 794.67 (m), 760.44 (s), 522.71 (vs), 519.82 (vs), 518.37 (vs).

GC/MS: m/z (+) = calc.: 316.1 [M]+, found: 316.1 [M]+

CHNS [%] calc.: C: 53.17, H: 6.69, N:0.00, S: 0.00
found: C: 52.84, H: 6.44, N: 0.37, S: 0.27

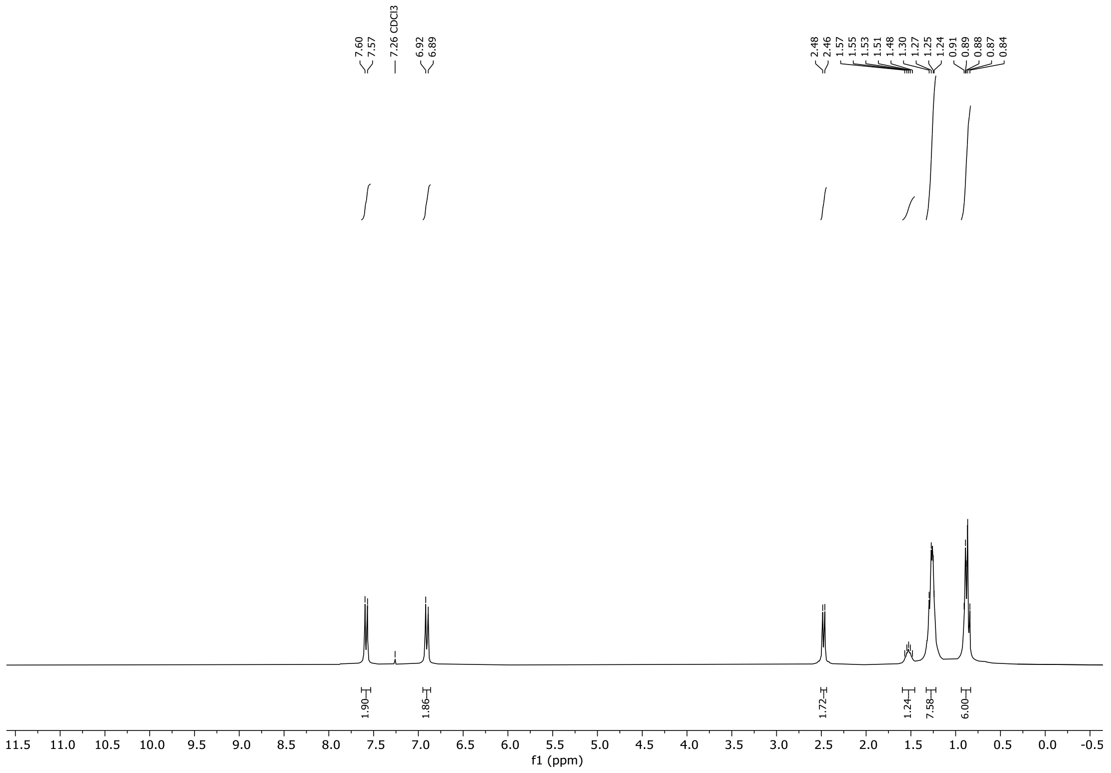

Figure S76: ^{1}H NMR spectrum (300 MHz) of compound **S10** in $CDCl_3$.

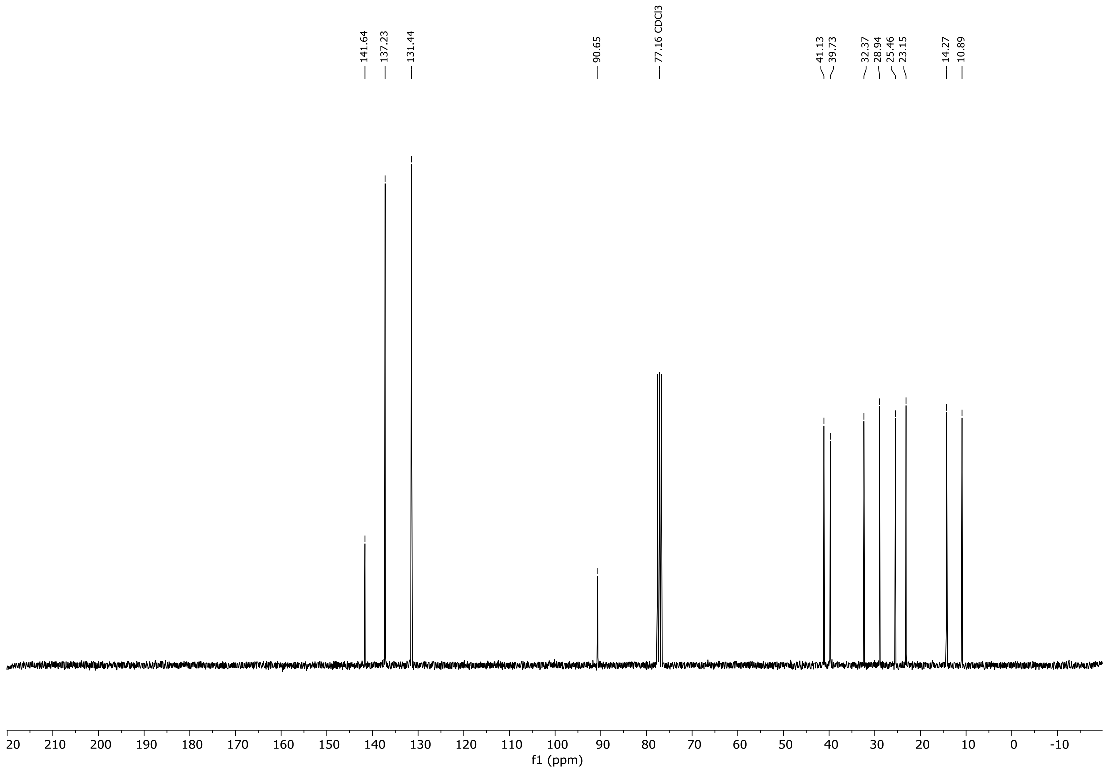

Figure S77: ^{13}C NMR spectrum (75 MHz) of compound **S10** in $CDCl_3$.

Synthesis of 2(4-(2-ethylhexyl)phenyl-4,4,5,5-tetramethyl-1,3,2-dioxaborolane (S11)

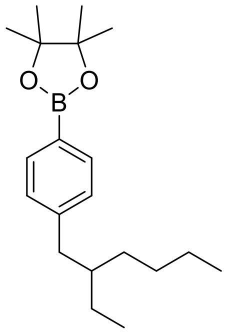

Chemical Formula: $C_{20}H_{33}BO_2$
Molecular Weight: 316,2920

Under inert conditions, dry 1,4-dioxane (0.5 M) and iodo compound **S10** (4.0 g, 12.6 mmol, 1.0 eq.) were degassed for 20 minutes. Bis(pinacolato)diborone (3.36 g, 13.2 mmol, 1.05 eq.), bis(triphenylphosphine)-palladium(II) dichloride (0.265 g, 0.378 mmol, 3 mol%) and potassium acetate (3.71 g, 37.8 mmol, 3.0 eq.) were added and the mixture was stirred at 110 °C overnight. After letting the reaction cool down to r.t., EtOAc was added, and the organic phase was washed thrice with brine before being dried over Na_2SO_4. The solvent was removed under reduced pressure, and the crude mixture was purified via silica column chromatography with 20:1 pentane: EtOAc as eluent. Finally, the product was obtained impurely as a brown oil (3.03 g, 76%) that was used without further purification.

^{1}H NMR (300 MHz, $CDCl_3$): δ = 7.72 (d, *J* = 8.2 Hz, 2H), 7.16 (d, *J* = 8.2 Hz, 2H), 2.54 (d, *J* = 7.2 Hz, 2H), 1.62 – 1.51(m, 1H), 1.34 (s, 12H), 1.31 – 1.20 (m, 10H), 0.95 – 0.78 (m, 8H) ppm.

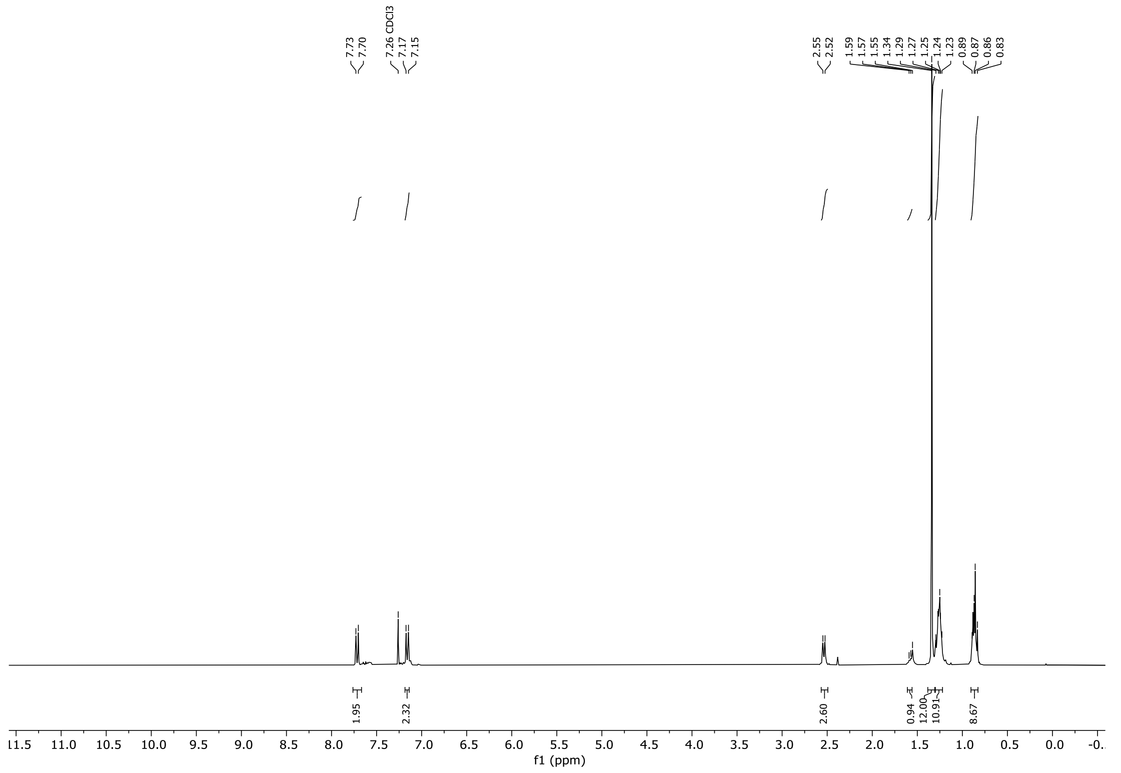

Figure S78: ^{1}H NMR spectrum (300 MHz) of compound **S11** in $CDCl_3$.

Synthesis of neopentyl 4'-bromo-2-nitro-[1,1'-biphenyl]-4-sulfonate (S12)

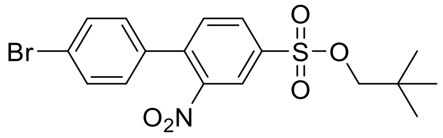

Chemical Formula: $C_{17}H_{18}BrNO_5S$
Molecular Weight: 428,2970

A water/1,4-dioxane mixture (1:4, 0.1 M) was degassed via argon bubbling for 30 minutes. Bromo compound **17** (9.02 g, 25.6 mmol, 1.0 eq.), 4-Bromophenylboronic acid (6.68 g, 33.3 mmol, 1.3 eq.), potassium carbonate (10.6 g, 76.8 mmol, 3.0 eq.) and bis(triphenylphosphine)palladium(II) dichloride (0.898 g, 1.28 mmol, 5 mol%) were added. The suspension was stirred at 70 °C for 13 hours. After cooling to ambient temperature, the ocher colored suspension was filtered with EtOAc over a plug of celite. It was washed with water (3 x 100 mL) and sat. NaCl solution (100 mL). The organic phase was dried over $MgSO_4$, and the solvent was removed. The crude mixture was purified via silica column chromatography with 10:1 to 1:1 pentane:EtOAc as eluent to yield the product as a colorless solid (1.34 g 12.2%).

^{1}H NMR (300 MHz, $CDCl_3$): δ = 8.38 (d, *J* = 1.8 Hz, 1H), 8.12 (dd, *J* = 8.1, 1.9 Hz, 1H), 7.68 – 7.53 (m, 3H), 7.21 (d, *J* = 8.6 Hz, 2H), 3.83 (s, 2H), 0.96 (s, 9H) ppm.

^{13}C NMR (75 MHz, $CDCl_3$): δ = 149.0, 140.2, 137.2, 134.6, 133.2, 132.4, 131.2, 129.5, 124.1, 124.0, 80.8, 32.0, 26.1 ppm.

ATR-IR [cm^{-1}]: 1588.86 (vs), 1526.18 (m), 1524.73 (m), 1521.84 (m), 1520.39 (m), 1497.73 (vs), 1478.44 (vs), 1468.79 (m), 1355.96 (vw), 1295.68 (vs), 1178.99 (vw), 1157.29 (s), 1112.93 (s), 1069.53 (m), 1015.52 (s), 1001.06 (m), 953.32 (vw), 940.78 (vw), 901.24 (m), 892.56 (vw), 850.61 (vw), 838.55 (m), 824.09 (vw), 779.72 (w), 763.33 (m), 758.51 (m), 741.63 (vs), 712.22 (s), 664.96 (vw), 633.13 (s), 627.35 (w), 601.79 (vw), 567.55 (w), 546.82 (vs), 537.18 (vw), 531.87 (w), 521.26 (s), 514.99 (m), 502.46 (vs), 472.08 (m), 462.92 (m), 453.75 (vs), 432.05 (vs), 430.61 (s), 417.59 (s), 415.66 (s).

ESI-MS: m/z (-) = calc. 426.0 [M-H]-, found 426.8 [M-H]-
m/z (+) = calc. 450.0 [M+Na]+, found 451.0 [M+Na]+

CHNS [%]: calc.: C: 47.67, H: 4.24, N:3.27, S: 7.49
found: C: 48.12, H: 4.19, N: 3.14, S: 7.07

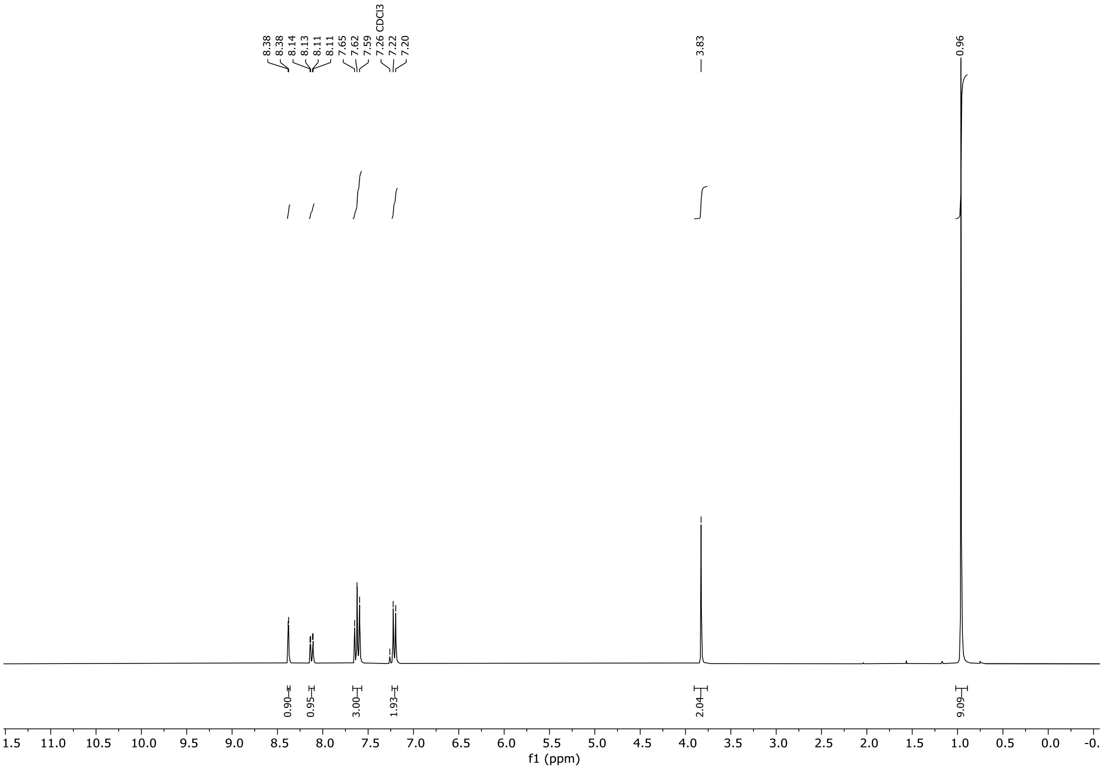

Figure S79: ^{1}H NMR spectrum (300 MHz) of compound **S12** in $CDCl_3$.

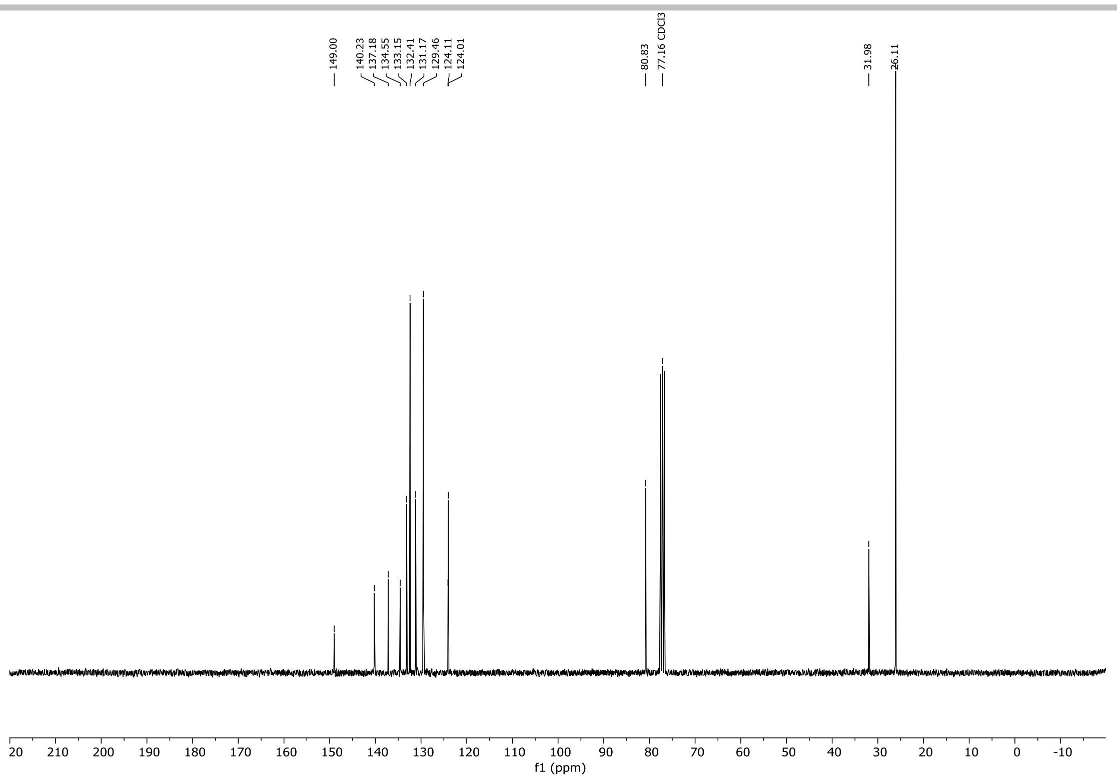

Figure S80: ^{13}C NMR spectrum (75 MHz) of compound **S12** in $CDCl_3$.

General coupling procedure to compounds S13 and S14

In a Schlenk flask, the boronic acid ester (1.3 eq or 1.5 eq.) was dissolved in water and 1,4-dioxane (1:4, 0.1 M) and the solution was degassed via argon bubbling for 20 minutes. Afterwards, the bromo compound (1.0 eq.), tris(dibenzylidenacetone) dipalladium(0) (3 mol%), SPhos (0.1 eq) and potassium phosphate (2.0 eq) were added. The mixture was heated to 70 °C overnight. After cooling to r.t., the reaction mixture was filtered over celite with EtOAc and the organic phase was washed with water (2 x 100 mL) brine (100 mL) before dried over Na_2SO_4. The crude mixture was purified via silica column chromatography.

Synthesis of neopentyl 4'-(2-ethylhexyl)-2-nitro-[1,1'-biphenyl]-4-sulfonate (S13)

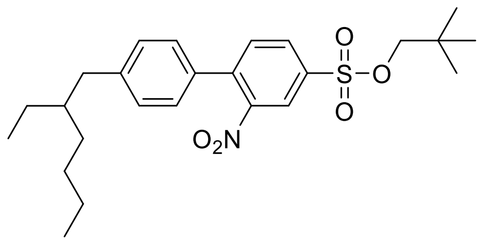

Chemical Formula: $C_{25}H_{35}NO_5S$
Molecular Weight: 461,6170

For the synthesis of biphenyl **S13**, 1.76 g (5.0 mmol, 1.0 eq.) of bromo compound **17** were reacted with 2.37 g (7.50 mmol, 1.5 eq.) of boronic acid ester **S11**, 136 mg (0.15 mmol, 3 mol%) of Pd_2dba_3, 209 mg (0.410 mmol, 0.1 eq.) SPhos and 2.12 g (10.0 mmol, 2.0 eq.) potassium phosphate. After the reaction and workup, the crude was purified by silica column chromatography using pentane and EtOAc as eluent (10:1) to give product **S13** as a yellow solid in 74% yield (1.7 g).

^{1}H NMR (300 MHz, DMSO-*d*$_6$): δ = 8.47 (d, *J* = 1.9 Hz, 1H), 8.23 (dd, *J* = 8.2, 1.9 Hz, 1H), 7.86 (d, *J* = 8.2 Hz, 1H), 7.37 – 7.22 (m, 4H), 3.90 (s, 2H), 2.56 (d, *J* = 7.1 Hz, 2H), 1.64 – 1.54 (m, 1H), 1.34 – 1.18 (m, 8H), 0.91 – 0.73 (m, 15H) ppm.

^{13}C NMR (75 MHz, DMSO-*d*$_6$) δ 148.9, 142.7, 140.0, 135.1, 133.5, 132.5, 131.1, 129.6, 127.6, 123.6, 80.5, 40.2, 39.1, 31.7, 31.5, 28.14, 25.5, 24.9, 22.4, 13.9, 10.5 ppm.

ATR-IR [cm^{-1}]: 2958,8 (s), 2925,53 (s), 1605,26 (s), 1533,41 (m), 1473,62 (vs), 1472,17 (vs), 1465,42 (vs), 1463,49 (vs), 1357,89 (w), 1181,4 (vw), 1148,61 (s), 956,69 (w), 938,37 (m), 891,6 (w), 846,75 (w), 834,21 (m), 809,14 (m), 773,94 (s), 756,58 (s), 669,78 (vs), 659,66 (m), 595,52 (vw), 567,07 (m), 547,78 (s), 530,91 (s).

ESI-MS: m/z (-) = calc. 460.2 [M-H]-, found 460.8 [M-H]-

CHNS [%]: calc.: C: 65.05, H: 7.64, N: 3.03, S: 6.95
found: C: 66.35, H: 7.62, N: 2.82, S: 6.13

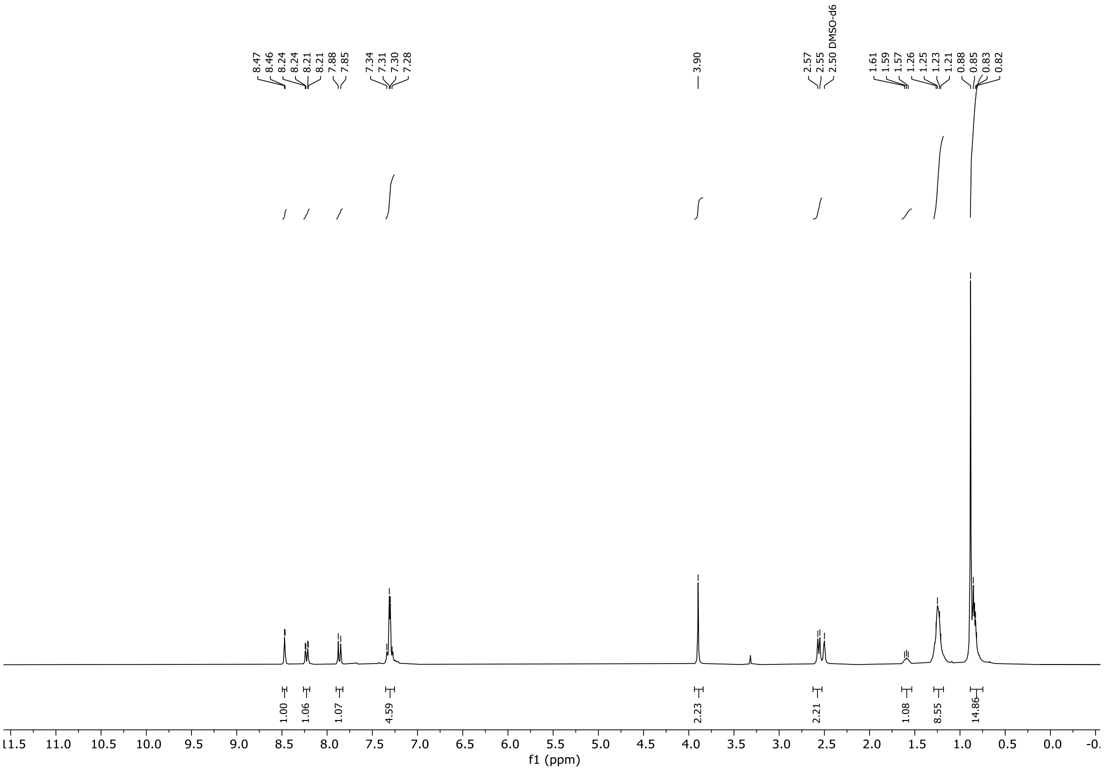

Figure S81: ^{1}H NMR spectrum (300 MHz) of compound **S13** in DMSO-*d*$_6$.

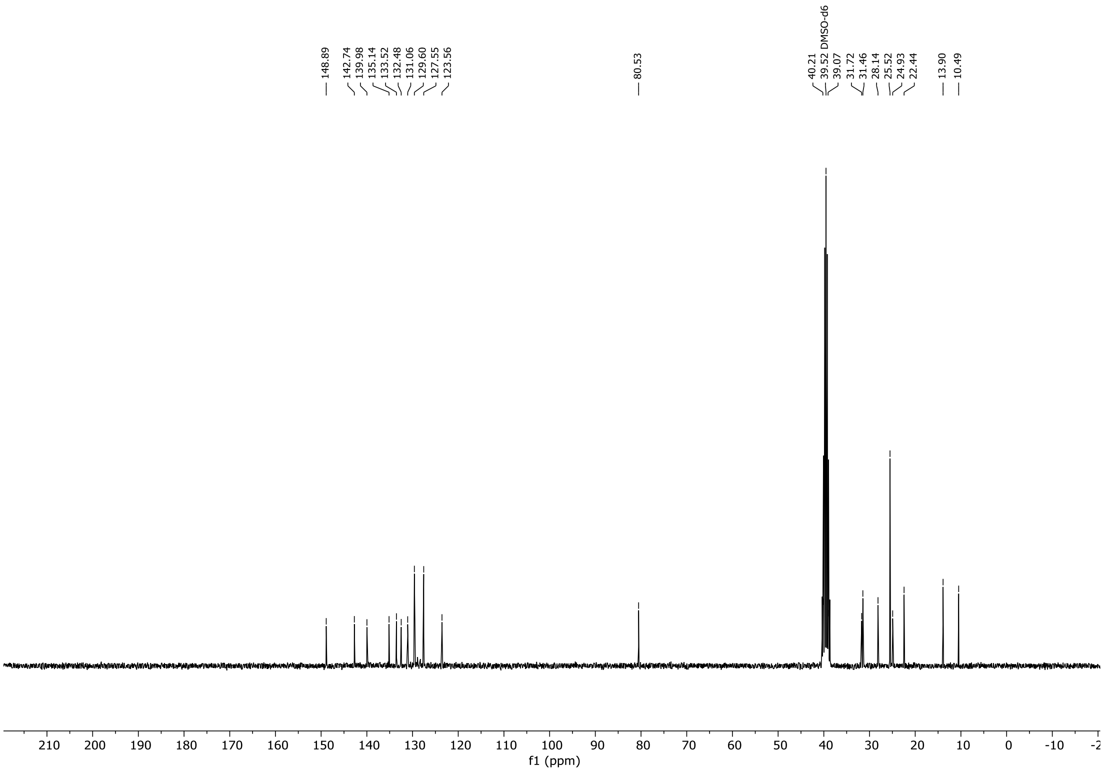

Figure S82: ^{13}C NMR spectrum (75 MHz) of compound **S13** in DMSO-*d6*.

Synthesis of neopentyl 3''-5''-di-tert-butyl-2-nitro-[1,1':4',1''-terphenyl]-4-sulfonate (S14)

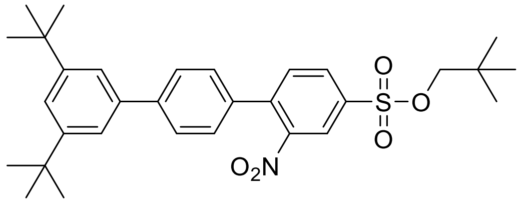

Chemical Formula: $C_{31}H_{39}NO_5S$
Molecular Weight: 537,7150

For the synthesis of terphenyl **S14**, 1.78 g (4.16 mmol, 1.0 eq.) of bromo compound **S12** were reacted with 1.71 g (5.41 mmol, 1.3 eq.) of boronic acid ester **19**, 114 mg (0.125 mmol, 3 mol%) of Pd_2dba_3, 171 mg (0.416 mmol, 0.1 eq.) SPhos and 1.77 g (8.32 mmol, 2.0 eq.) potassium phosphate. After the reaction and workup, the crude was purified by silica column chromatography using pentane and EtOAc as eluent (20:1) to give product **S14** as a pale-yellow solid in 26% yield (570 mg).

^{1}H NMR (300 MHz, $CDCl_3$): δ = 8.37 (d, *J* = 1.8 Hz, 1H), 8.14 (dd, *J* = 8.1, 1.9 Hz, 1H), 7.77 – 7.64 (m, 3H), 7.52 – 7.37 (m, 5H), 3.84 (s, 2H), 1.40 (s, 18H), 0.98 (s, 9H) ppm.

^{13}C NMR (75 MHz, $CDCl_3$): δ = 151.5, 149.3, 143.8, 141.1, 139.5, 136.6, 134.0, 133.3, 131.0, 128.2, 123.9, 122.2, 121.8, 80.8, 35.1, 32.0, 31.7, 26.1 ppm.

ATR-IR [cm^{-1}]: 2955.91 (s), 1596.58 (vs), 1545.46 (m), 1544.02 (m), 1542.57 (m), 1540.64 (s), 1539.20 (s), 1476.51 (vs), 1475.06 (vs), 1365.12 (m), 1361.74 (m), 1354.03 (w), 1248.43 (s), 1184.29 (vw), 1005.88 (vs), 952.84 (w), 935.48 (m), 894.97 (w), 877.13 (m), 856.39 (m), 848.20 (w), 841.93 (m), 827.94 (vw), 774.42 (m), 711.73 (m), 664.48 (m), 605.17 (vw), 602.76 (vw), 566.59 (m).

ESI-MS: m/z (+) = calc. 576.8 [M+K]+, found 577.0 [M+K]+

CHNS [%]: calc.: C: 69.24, H: 7.31, N: 2.60, S: 5.96
found: C: 69.36, H: 7.18, N: 2.52, S: 5.50

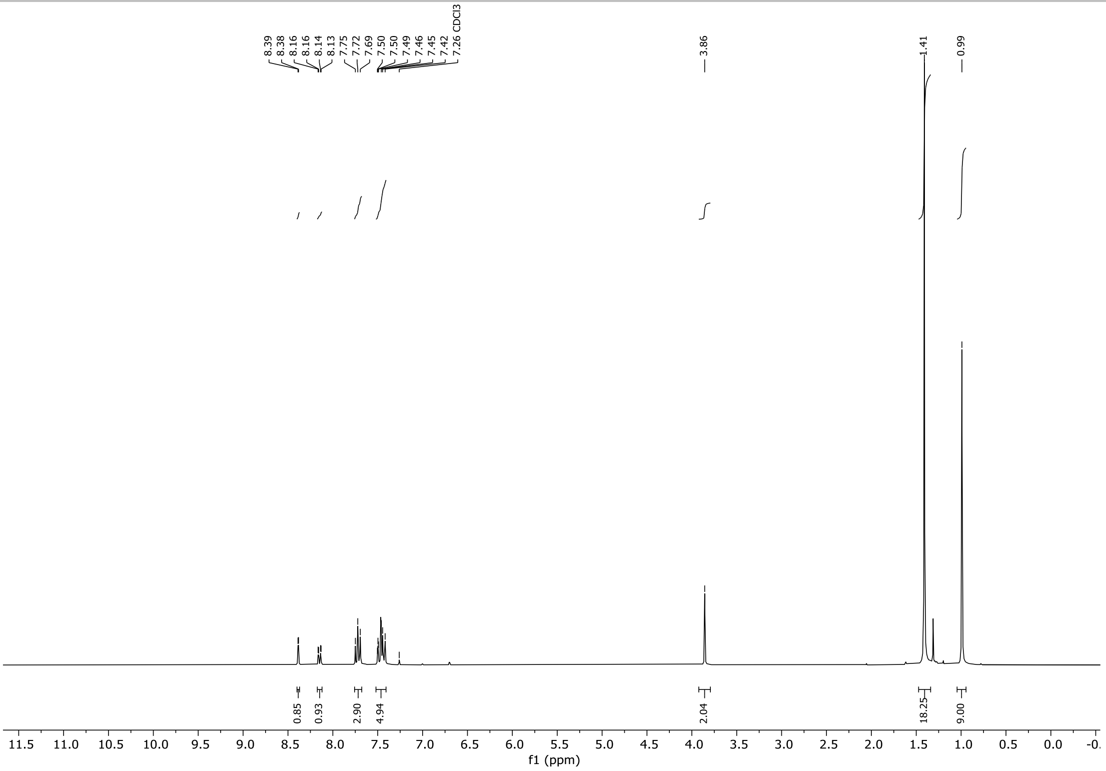

Figure S83: ^{1}H NMR spectrum (300 MHz) of compound **S14** in $CDCl_3$.

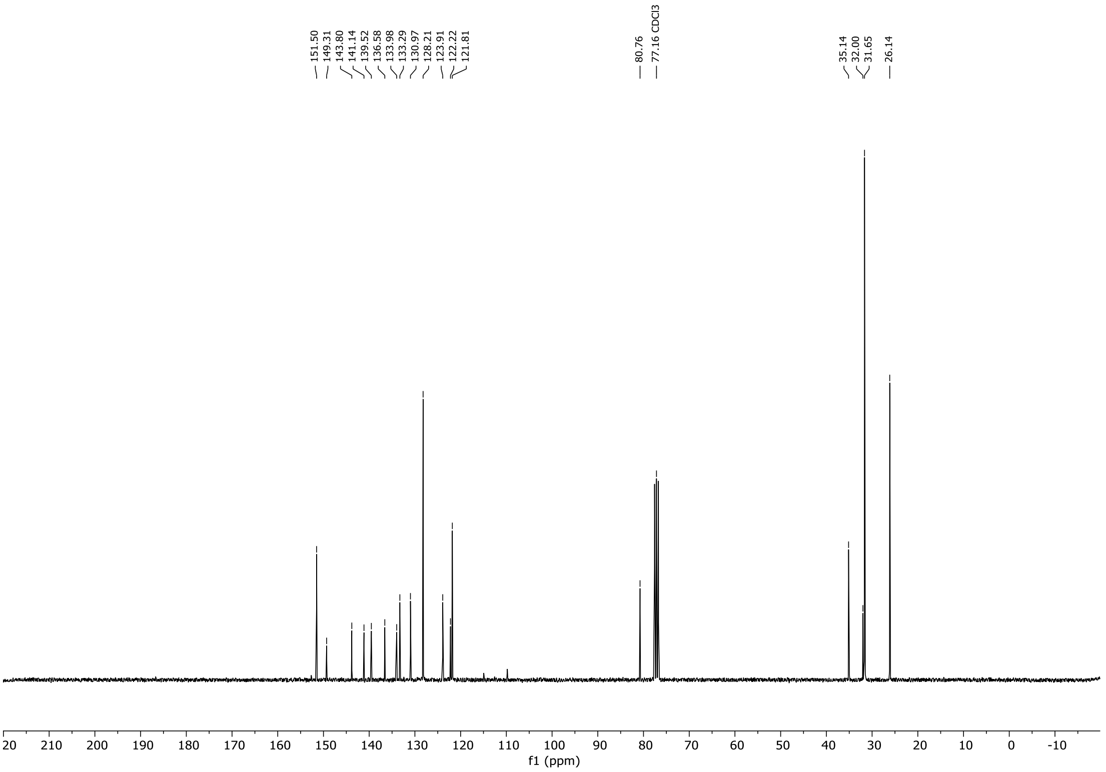

Figure S84: ^{13}C NMR spectrum (75 MHz) of compound **S14** in $CDCl_3$.

General reduction procedure to amines S15 and S16

The nitro compound (1.0 eq.) was suspended in EtOH (0.1 M) and 2 mol% of Pd on charcoal (4% of Pd on charcoal) were added. The reaction was stirred with a hydrogen atmosphere until the reduction was completed (followed by TLC). The mixture was filtered over a plug of celite using EtOAc as eluent. The solvent was removed, and the crude mixture was purified by silica column chromatography.

Synthesis of neopentyl 2-amino-4'-(2-ethylhexyl)-[1,1'-biphenyl]-4-sulfonate (S15)

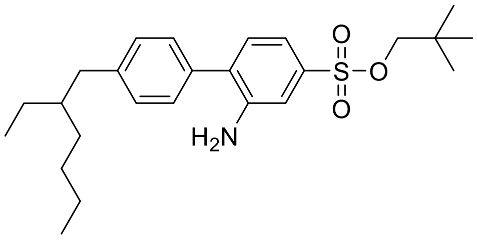

Chemical Formula: $C_{25}H_{37}NO_3S$
Molecular Weight: 431,6350

Nitro compound **S13** (700 mg, 1.52 mmol, 1.0 eq.) and Pd/C (81 mg, 4%, 0.03 mmol, 2 mol%) were reacted in EtOH (0.2 M) under an hydrogen atmosphere. The crude product was purified by silica column chromatography with pentane and EtOAc (10:1) was eluent. Amine **S15** was isolated as white solid (555 mg, 85%).

^{1}H NMR (300 MHz, DMSO-d_6): δ = 7.36 (d, *J* = 8.2 Hz, 2H), 7.32 – 7.17 (m, 4H), 7.05 (dd, *J* = 7.9, 2.0 Hz,1H), 5.38 (s, 2H), 3.71 (s, 2H), 2.54 (d, *J* = 6.1 Hz, 2H), 1.65 – 1.53 (m, 1H), 1.36 – 1.20 (m, 8H), 0.91 – 0.77 (m, 15H) ppm.

^{13}C NMR (75 MHz, DMSO-d_6): δ = 146.2, 140.8, 135.0, 134.5, 131.0, 130.4, 129.5, 128.2, 114.7, 113.1, 79.1, 40.3, 39.1, 31.7, 31.3, 28.2, 25.7, 24.8, 22.5, 14.0, 10.5 ppm.

ATR-IR [cm^{-1}]: 2956.87 (m), 2925.05 (m), 2870.56 (vs), 2857.06 (s), 1624.06 (m), 1616.83 (s), 1476.51 (m), 1464.94 (s), 1458.67 (s), 1456.74 (s), 1424.43 (m), 1401.77 (m), 1365.60 (s), 1349.69 (vw), 1323.65 (m), 1286.04 (s), 1173.20 (vw), 1002.50 (s), 962.96 (vw), 937.89 (m), 922.94 (m), 831.32 (vw), 799.49 (w), 756.10 (m), 712.70 (m), 659.66 (m), 622.53 (m), 600.34 (vw), 581.54 (m), 562.73 (vs), 538.14 (w), 482.69 (s), 479.31 (s), 476.90 (s), 463.40 (m), 461.95 (m), 460.02 (m), 451.83 (s), 450.86 (s), 422.89 (vs), 418.55 (s), 416.62 (s), 414.21 (vs), 411.32 (vs).

ESI-MS: m/z (+) = calc. 400.2 [M-CH_3OH+H]+, found 400.6 [M-CH_3OH+H]+

CHNS [%]: calc.: C: 69.57, H: 8.64, N: 3.25, S: 7.43
found: C: 69.84, H: 8.63, N: 3.15, S: 7.05

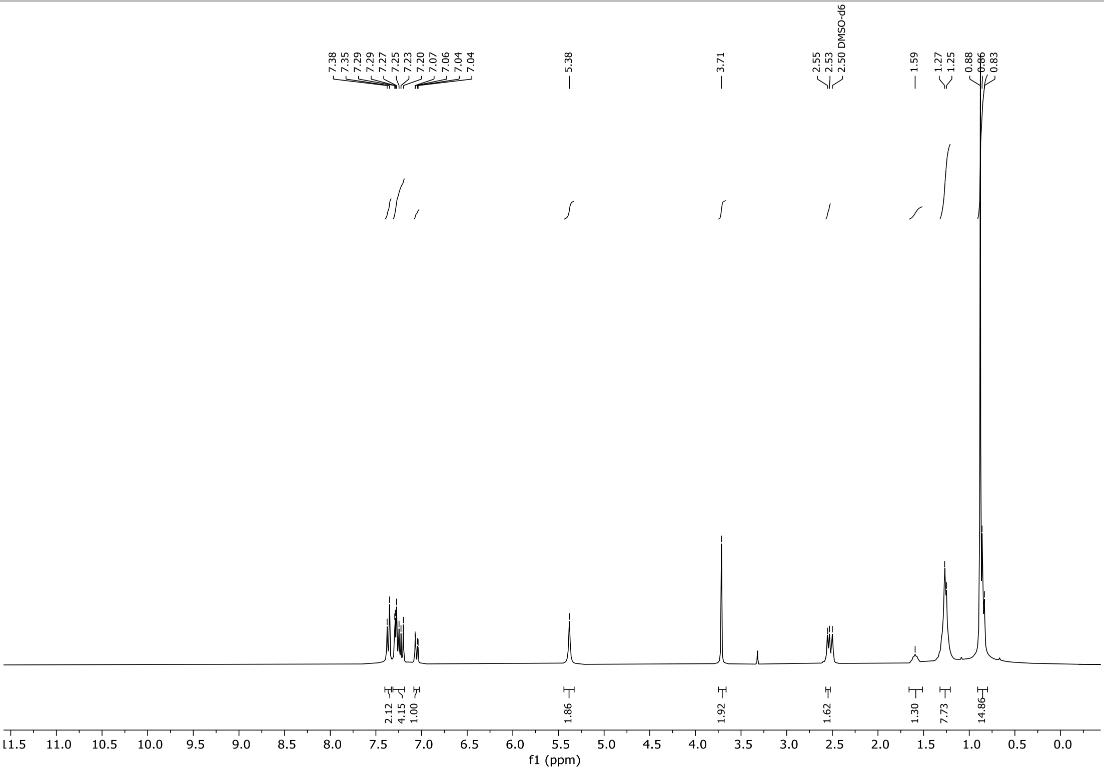

Figure S85: ^{1}H NMR spectrum (300 MHz) of compound **S15** in DMSO-d_6.

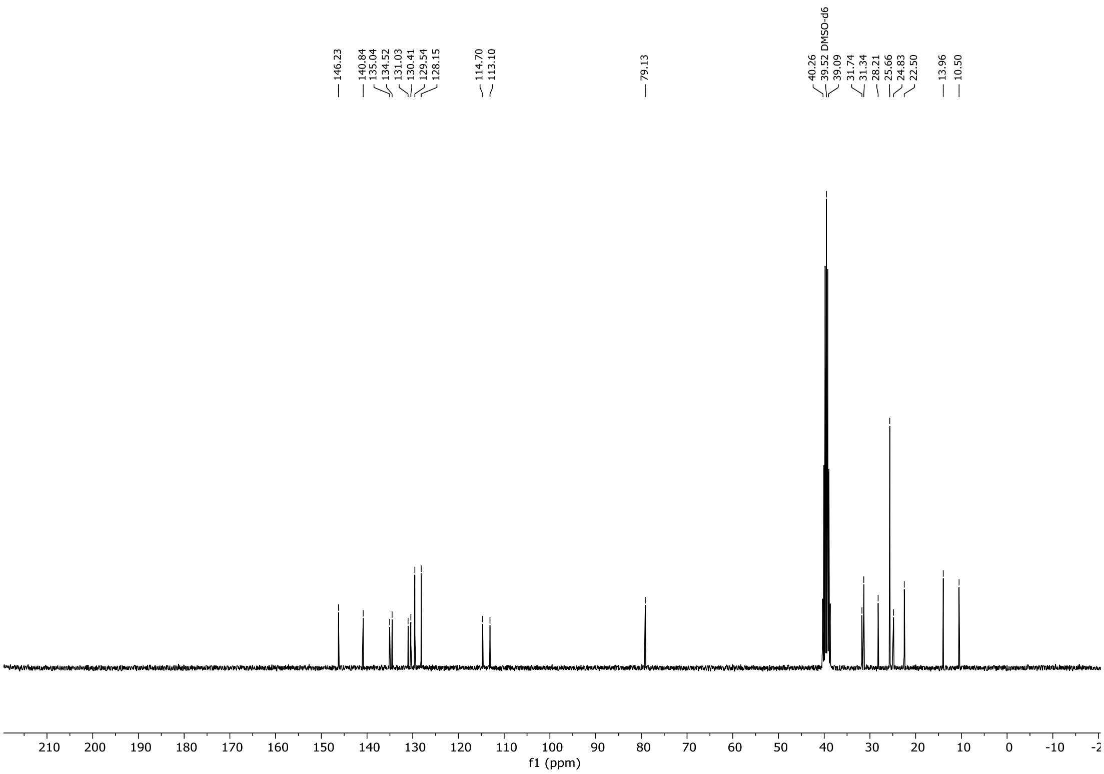

Figure S86: ^{13}C NMR spectrum (75 MHz) of compound **S15** in DMSO-d_6.

Synthesis of neopentyl 2-amino-3'',5''-di-tert-butyl-[1,1':4',1''-terphenyl]-4-sulfonate (S16)

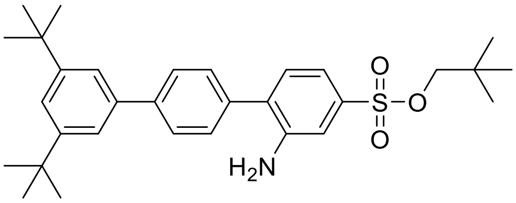

Chemical Formula: $C_{31}H_{41}NO_3S$
Molecular Weight: 507,7330

Nitro compound **S14** (755 mg, 1.4 mmol, 1.0 eq.) and Pd/C (75 mg, 4%, 0.03 mmol, 2 mol%) were reacted in EtOH (0.2 M) under a hydrogen atmosphere. The crude product was purified by silica column chromatography with pentane and EtOAc (5:1) was eluent. Amine **S16** was isolated as pale-yellow solid (662 mg, 93%).

^{1}H NMR (300 MHz, DMSO-*d*$_6$): δ = 7.75 (d, *J* = 8.4 Hz, 2H), 7.54 (d, *J* = 8.4 Hz, 2H), 7.47 (d, *J* = 1.7 Hz, 2H), 7.43 (t, *J* = 1.8 Hz, 1H), 7.32 (d, *J* = 1.9 Hz, 1H), 7.27 (d, *J* = 8.0 Hz, 1H), 7.09 (dd, *J* = 8.0, 1.9 Hz, 1H), 5.49 (s, 2H), 3.74 (s, 2H), 1.35 (s, 18H), 0.90 (s, 9H) ppm.

^{13}C NMR (75 MHz, DMSO-*d*$_6$): δ = 150.9, 146.4, 140.7, 139.2, 136.6, 134.8, 131.1, 130.1, 129.0, 127.5, 121.0, 114.7, 113.2, 79.2, 34.7, 31.4, 31.3, 25.7 ppm.

ATR-IR [cm^{-1}]: 2955,91 (m), 1619,24 (s), 1595,13 (s), 1475,54(s), 1420,57 (s), 1348,72 (w), 1321,24 (s), 1246,98 (s), 1173,68 (vw), 957,66 (w), 935,96 (m), 925,35 (m), 877,13 (s), 815,41 (m), 811,55 (w), 756,1 (vs), 725,72 (vs), 712,7 (m), 680,87 (m), 620,11 (m), 601,31 (vw), 581,54 (s), 534,76 (s), 460,5 (vs).

ESI-MS: m/z (+) = calc. 508.3 [M+H]+, found 508.4 [M+H]+

CHNS [%]: calc.: C: 73.33, H: 8.14, N: 2.76, S: 6.32
found: C: 73.22, H: 7.98, N: 2.70, S: 5.61

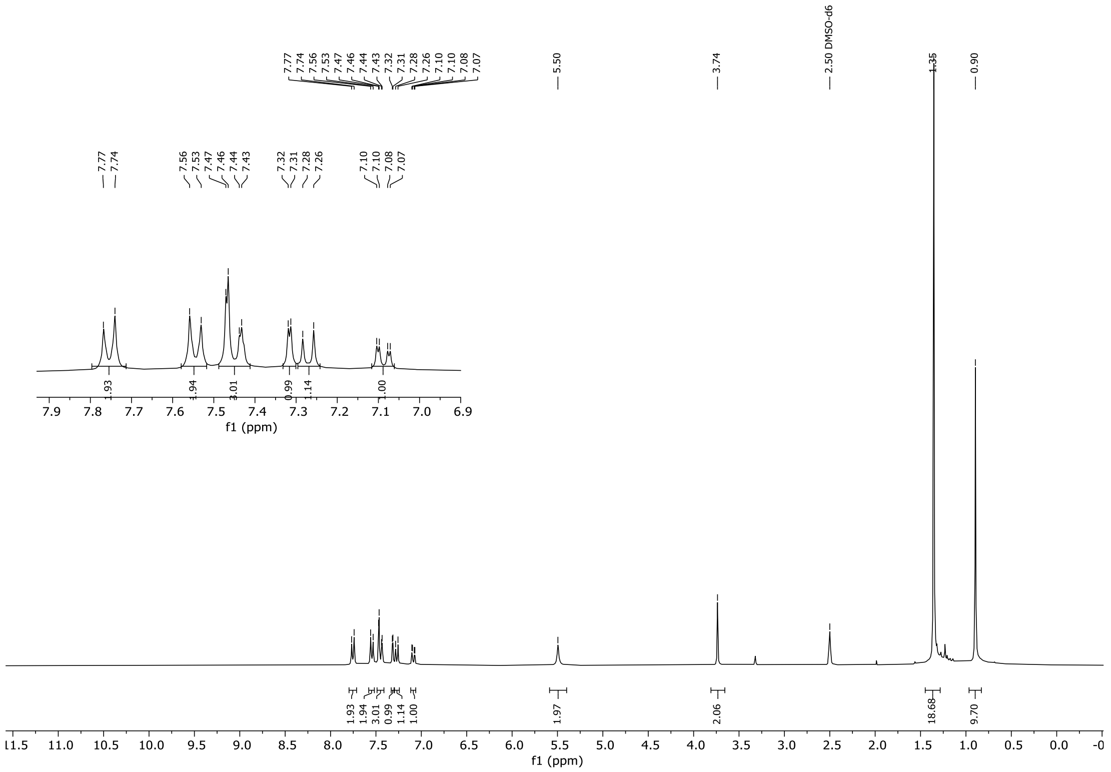

Figure S87: ^{1}H NMR spectrum (300 MHz) of compound **S16** in DMSO-*d*$_6$.

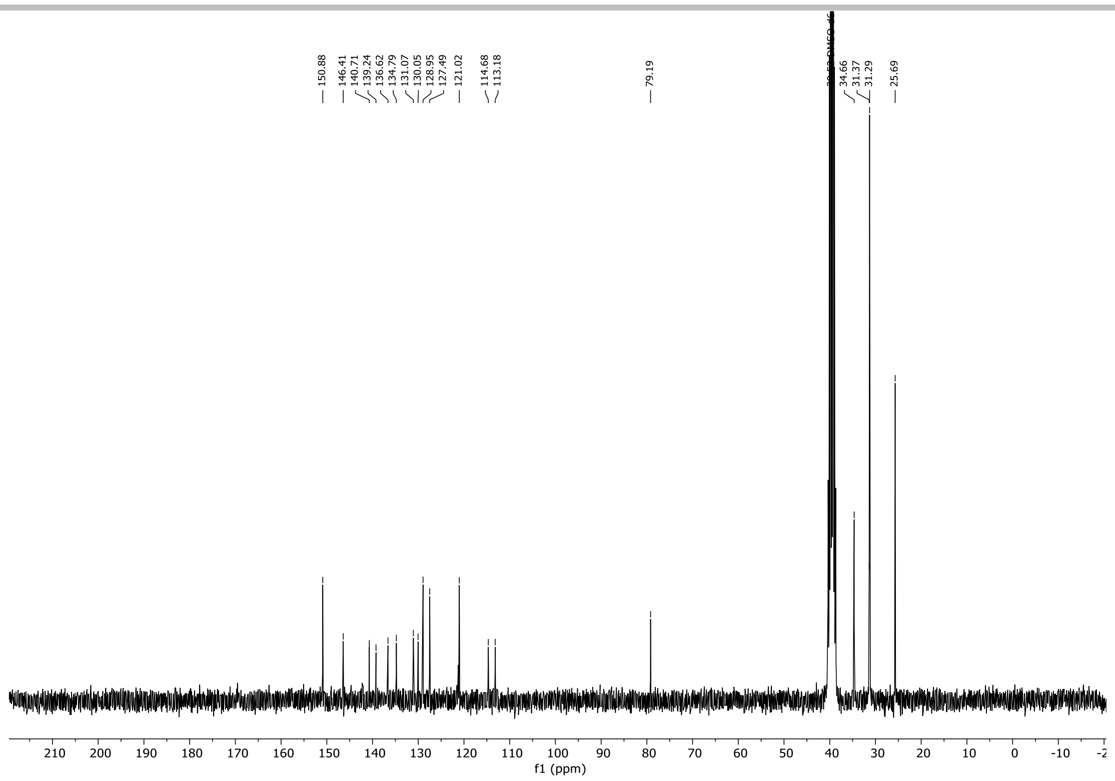

Figure S88: ^{13}C NMR spectrum (75 MHz) of compound **S16** in DMSO-d_6.

General Sandmeyer type reaction to iodo compounds S17 and S18

The Sandmeyer type reaction follows the procedure of Samanta et al.[17]

Synthesis of neopentyl 4'-(2-ethylhexyl)-2-iodo-[1,1'-biphenyl]-4-sulfonate (S17)

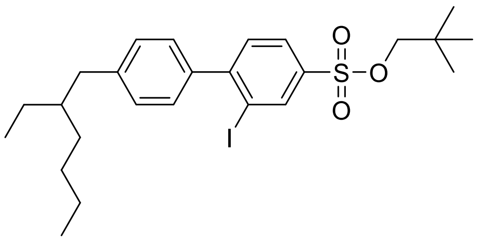

Chemical Formula: $C_{25}H_{35}IO_3S$
Molecular Weight: 542,5165

Amine **S15** (150 mg, 0.348 mmol, 1.0 eq.) was reacted in 2 ml of ACN (0.2 M) according to the procedure of Samanta et al.[17]. The crude mixture was purified by silica column chromatography using pentane and EtOAc as eluent (10:1) resulting in 113 mg (60%) of the desired product **S17**.

^{1}H NMR (300 MHz, DMSO-d_6): δ = 8.35 (d, J = 1.9 Hz, 1H), 7.96 (dd, J = 8.1, 2.0 Hz, 1H), 7.58 (d, J = 8.1 Hz, 1H), 7.49 – 7.03 (m, 4H), 3.82 (s, 2H), 2.57 (d, J = 7.1 Hz, 2H), 1.66 – 1.53 (m, 1H), 1.35 – 1.17 (m, 8H), 0.91 – 0.79 (m, 15H) ppm.

^{13}C NMR (75 MHz, DMSO-d_6): δ = 151.4, 141.7, 139.4, 137.5, 135.2, 131.0, 128.8, 128.6, 127.3, 99.5, 80.0, 40.2, 39.1, 31.7, 31.4, 28.2, 25.6, 24.9, 22.5, 14.0, 10.5 ppm.

ATR-IR [cm^{-1}]: 2958.32 (m), 2872.01 (s), 1476.51 (vs), 1464.94 (s), 1459.63 (m), 1457.22 (m), 1455.29 (s), 1359.82 (vw), 1345.35 (m), 1178.51 (vw), 1103.28 (m), 1000.57 (m), 954.76 (vw), 937.40 (vw), 851.09 (vw), 831.32 (w), 823.12 (w), 780.21 (m), 769.12 (s), 757.54

(m), 725.72 (vs), 661.58 (m), 654.35 (vw), 639.40 (m), 591.66 (vw), 558.87 (w), 534.76 (m), 532.35 (m), 524.64 (m), 461.47 (m), 452.79 (m), 445.07 (m), 438.81 (m), 437.36 (m), 435.43 (s), 433.02 (m), 431.57 (s), 406.50 (vs).

ESI-MS: m/z (+) = calc. 565.1 [M+Na]+, found 565.3 [M+Na]+

CHNS [%]: calc.: C: 55.35, H: 6.50, N: 0.00, S: 5.91
found: C: 55.75, H: 6.42, N: 0.11, S: 5.45

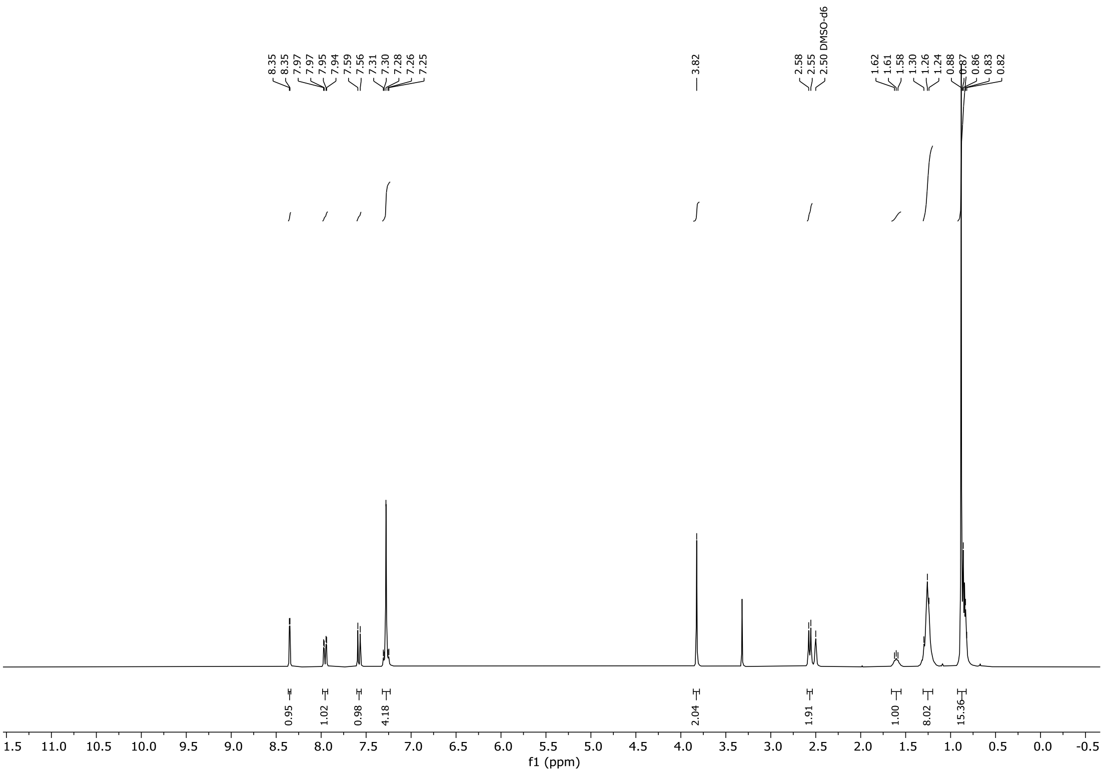

Figure S89: ^{1}H NMR spectrum (300 MHz) of compound **S17** in DMSO-d_6.

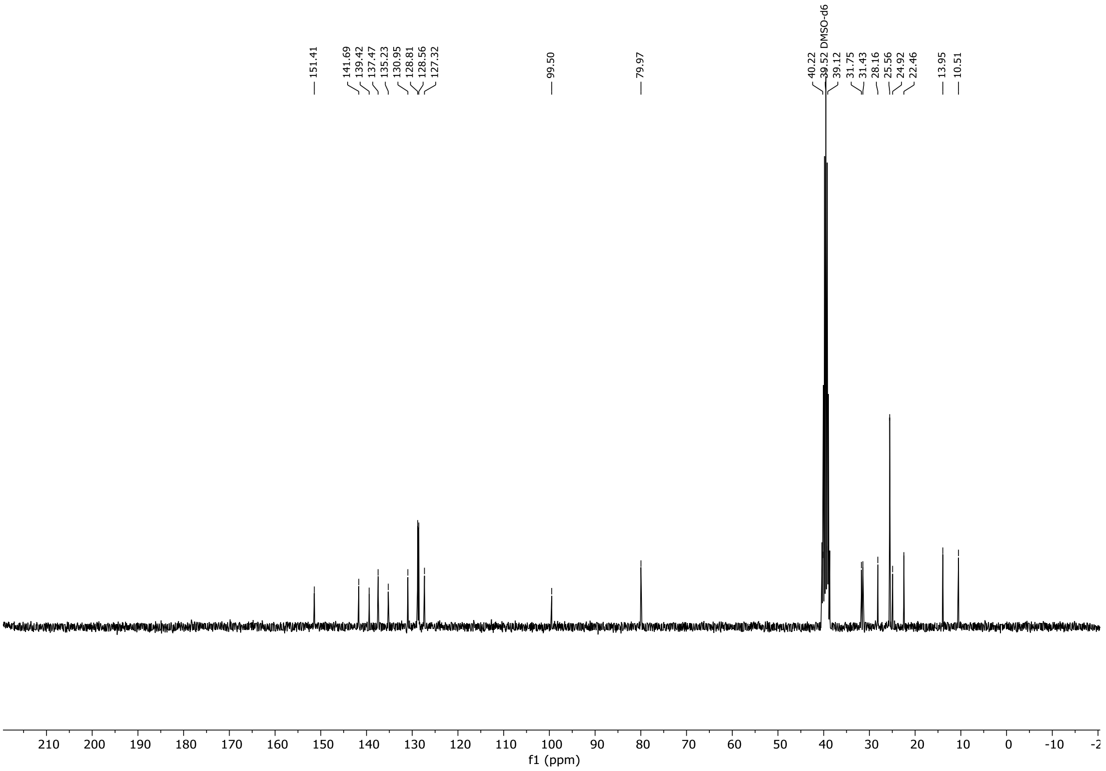

Figure S90: ^{13}C NMR spectrum (75 MHz) of compound **S17** in DMSO-d_6.

Synthesis of neopentyl 3'',5''-di-tert-butyl-2-iodo-[1,1':4',1''-terphenyl]-4-sulfonate (S18)

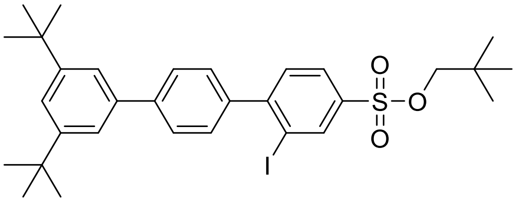

Chemical Formula: $C_{31}H_{39}IO_3S$
Molecular Weight: 618,6145

Amine **16** (350 mg, 0.689 mmol, 1.0 eq.) was reacted in 3.5 ml of ACN (0.2 M) according to the procedure of Samanta et al.[17] The crude mixture was purified by silica column chromatography using pentane and EtOAc as eluent (10:1) resulting in 343 mg (81%) of the desired product **S18**.

^{1}H NMR (300 MHz, $CDCl_3$): δ = 8.50 (d, *J* = 1.9 Hz, 1H), 7.93 (dd, *J* = 8.0, 1.9 Hz, 1H), 7.70 (d, *J* = 8.5 Hz, 2H), 7.56 – 7.38 (m, 6H), 3.80 (s, 2H), 1.41 (s, 18H), 0.98 (s, 9H) ppm.

^{13}C NMR (75 MHz, $CDCl_3$): δ = 151.9, 151.4, 142.9, 141.1, 139.9, 138.7, 136.2, 130.6, 129.3, 127.5, 127.4, 122.0, 121.8, 98.6, 80.3, 35.1, 32.0, 31.7, 26.2 ppm.

ATR-IR [cm^{-1}]: 1462.04 (vs), 1360.30 (vw), 1345.83 (s), 1247.46 (s), 1179.95 (vw), 1104.25 (s), 957.17 (w), 936.92 (m), 878.09 (s), 856.39 (w), 848.68 (w), 826.50 (vw), 801.91 (vs), 779.24 (m), 713.18 (m), 685.21 (m), 655.32 (m), 646.15 (vs), 605.65 (vw), 599.38 (vw), 572.86 (m), 539.59 (s), 442.66 (s).

FD-MS: m/z (+) = calc.: 618.1665 [M]+, found: 618.1830 [M]+

CHNS [%]: calc.: C: 60.19, H: 6.35, N: 0.00, S: 5.18
found: C: 60.27, H: 6.24, N: 0.12, S: 4.83

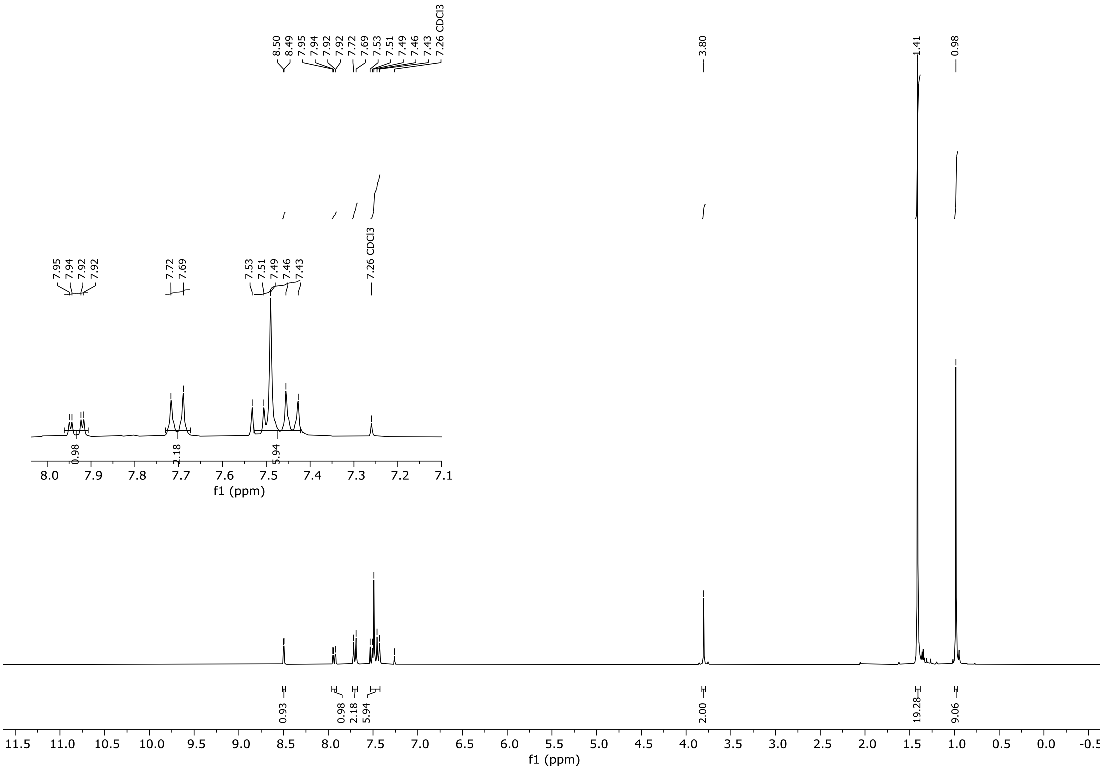

Figure S91: ^{1}H NMR spectrum (300 MHz) of compound **S18** in $CDCl_3$.

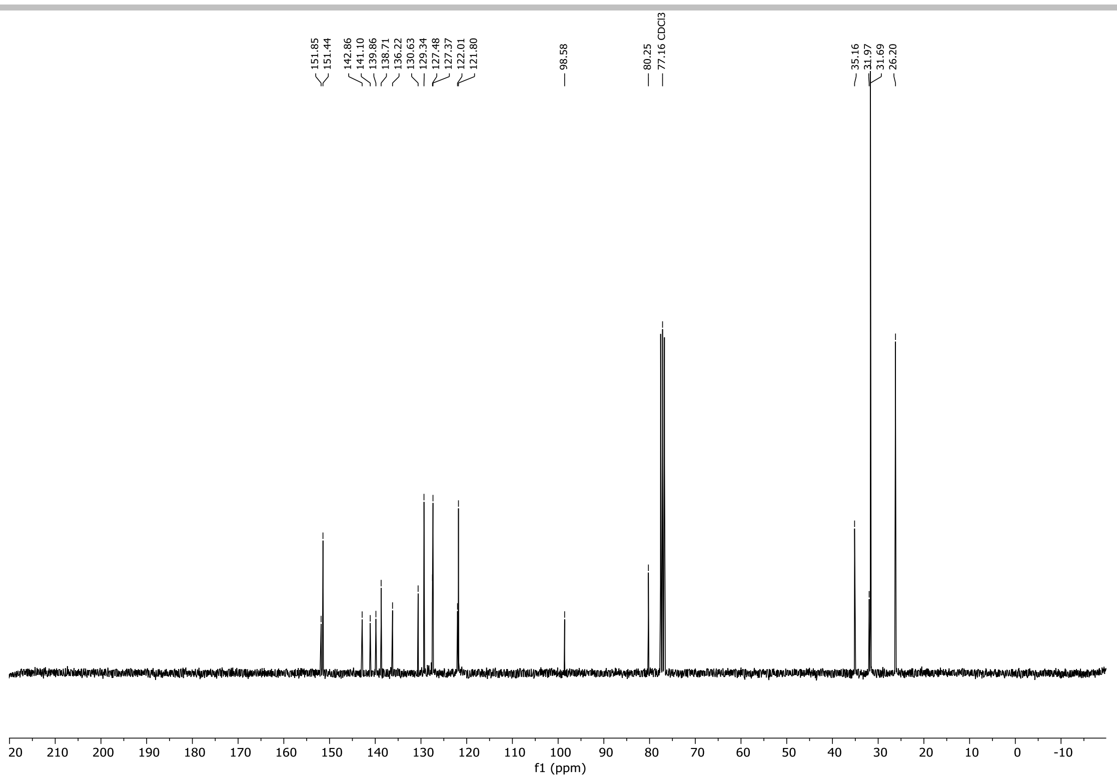

Figure S92: ^{13}C NMR spectrum (75 MHz) of compound **S18** in $CDCl_3$.

General reaction to sulfonic acids S19 and S20

The sulfonic acid ester was suspended in TFA (0.2 M) and heated to 70 °C. At this temperature, the mixture was stirred overnight. After cooling to r.t., the volatile components were removed under reduced pressure and co evaporated twice with DCM. The obtained residue was redissolved in DCM and filtered through a Whatman filter. After removal of the solvent, the product was obtained and used without further purification.

Synthesis of 4'-(2-ethylhexyl)-2-iodo-[1,1'-biphenyl]-4-sulfonic acid (S19)

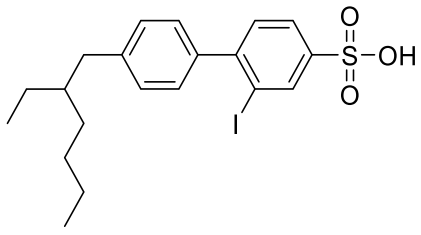

Chemical Formula: $C_{20}H_{25}IO_3S$
Molecular Weight: 472,3815

Sulfonic acid ester **S17** (300 mg, 0.553 mmol, 1.0 eq.) was reacted in TFA (0.2 M). After workup, **S19** was obtained as a purple oil with remaining acid used without further purification (quant.).

^{1}H NMR (400 MHz, DMSO-*d*$_6$): δ = 8.12 (s, 1H), 7.63 (dd, *J* = 7.8, 1.8 Hz, 1H), 7.36 – 7.13 (m, 5H), 2.55 (d, *J* = 7.1 Hz, 2H), 1.74 – 1.52 (m, 1H), 1.34 – 1.20 (m, 8H), 0.91 – 0.81 (m, 6H) ppm.

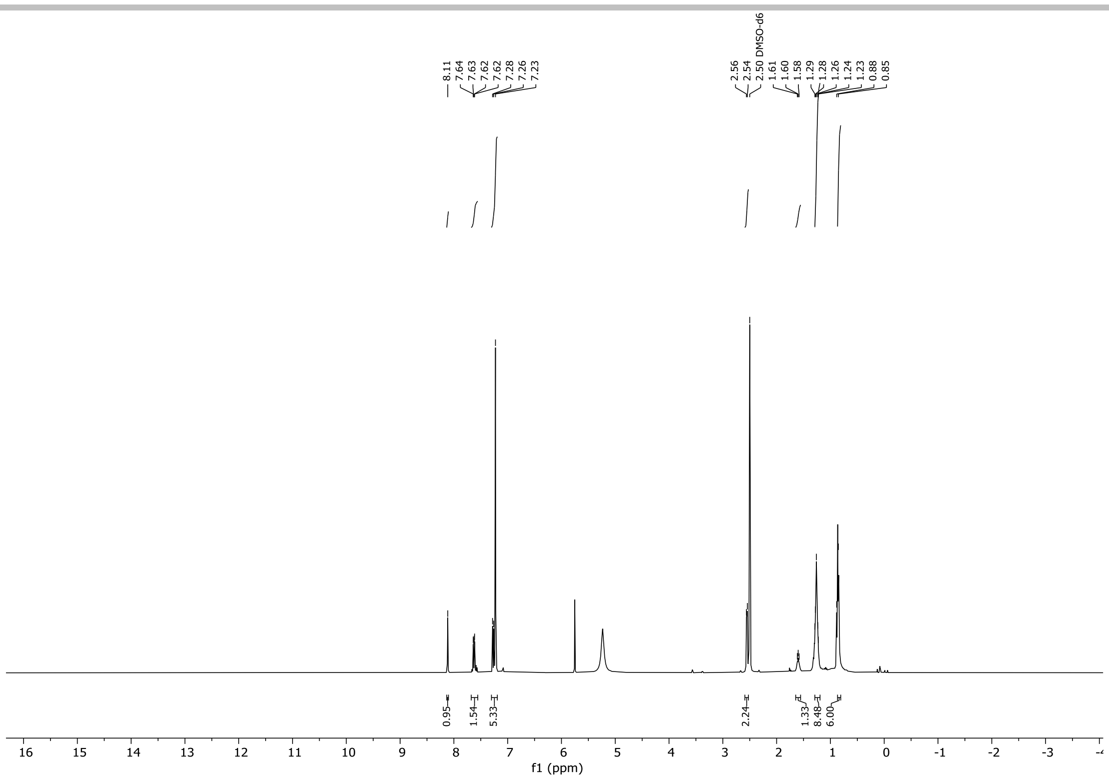

Figure S93: ^{1}H NMR spectrum (400 MHz) of compound **S19** in DMSO-d_6.

Synthesis of 3'',5''-di-tert-butyl-2-iodo-[1,1':4',1''-terphenyl]-4-sulfonic acid (S20)

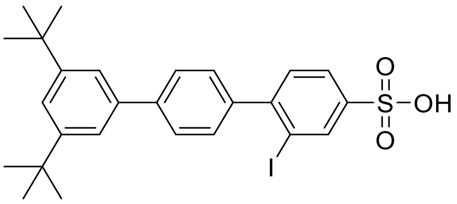

Chemical Formula: $C_{26}H_{29}IO_3S$
Molecular Weight: 548,4795

Sulfonic acid ester **S18** (77.6 mg, 0.125 mmol, 1.0 eq.) was reacted in TFA (0.2 M). After workup, **S20** was obtained as a purple oil with remaining acid used without further purification (quant.).

^{1}H NMR (300 MHz, DMSO-d_6): δ = 8.15 (d, J = 1.7 Hz, 1H), 7.73 (d, J = 8.4 Hz, 2H), 7.67 (dd, J = 7.8, 1.7 Hz, 1H), 7.49 (d, J = 1.8 Hz, 2H), 7.45 – 7.39 (m, 3H), 7.33 (d, J = 7.9 Hz, 1H), 1.36 (s, 18H) ppm.

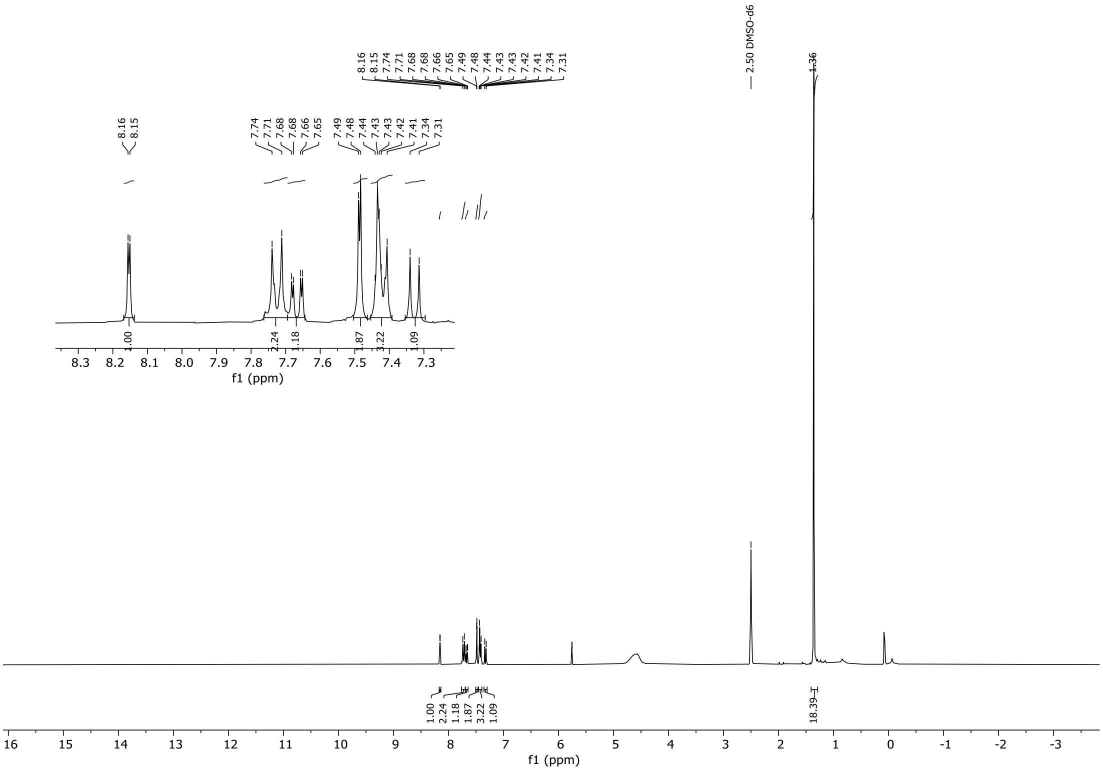

Figure S94: ^1H NMR spectrum (300 MHz) of compound **S20** in DMSO-d_6.

General oxidation procedure to iodolium salts S21 and S22

The oxidation follows the procedure of Oloffson et al.[13] The iodo compound (1.0 eq.) was dissolved in DCM (0.2 M) and mCPBA (1.5 eq.) was added. The reaction mixture was stirred at r.t. for 10 minutes before it was cooled to 0 °C with an ice bath. To that, HOTf (3.0 eq.) was added, and the suspension was stirred overnight while warming to r.t.. The volatile components were removed under reduced pressure and the remaining solid was washed by stirring in DEE. Finally, the product was isolated by filtration.

Synthesis of 3-(2-ethylhexyl)-7-sulfodibenzo[b,d]iodol-5-ium trifluoromethanesulfonate (S21)

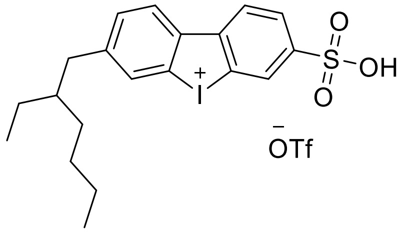

Chemical Formula: $C_{21}H_{24}F_3IO_6S_2$
Molecular Weight: 620,4367

For the oxidation according to the given procedure,[13] 183 mg (0.387 mmol, 1.0 eq.) of iodo compound **S19** were reacted with 100 mg (0.581 mmol, 1.5 eq.) mCPBA and 0.1 ml (1.16 mmol, 3.0 eq.) HOTf. After workup, iodolium salt **S21** was obtained as an off-white solid (181 mg, 75%).

^{1}H NMR (400 MHz, DMSO-d_6): δ = 8.49 (d, *J* = 1.5 Hz, 1H), 8.37 (t, *J* = 8.6 Hz, 2H), 8.02 – 7.94 (m, 2H), 7.66 (d, *J* = 8.1 Hz, 1H), 2.69 (d, *J* = 4.8 Hz, 2H), 1.71 – 1.51 (m, 1H), 1.28 (h, *J* = 5.7 Hz, 8H), 0.87 (m, 6H) ppm.

^{13}C NMR (101 MHz, DMSO-d_6): δ = 150.7, 145.8, 142.1, 139.4, 132.2, 130.9, 128.3, 128.2, 127.2, 126.7, 122.5, 121.5, 41.0, 32.2, 28.6, 25.3, 22.9, 14.4, 11.0 ppm.

^{19}F NMR (376 MHz, DMSO-d_6): δ = -77.75 ppm.

ATR-IR [cm^{-1}]: 418.55 (s), 514.03 (s), 562.25 (m), 598.90 (m), 617.22 (m), 650.01 (w), 668.33 (m), 682.80 (vs), 698.23 (m), 765.74 (s), 831.32 (m), 890.15 (s), 1016.49 (vw), 1030.95 (vw), 1110.03 (vw), 1170.79 (vw), 1223.83 (w), 1375.25 (m), 1454.33 (m), 1594.17 (s), 2856.58 (s), 2925.05 (m), 2953.98 (s), 3093.82 (vs), 3482.48 (vs), 3547.09 (vs).

ESI-MS: m/z (-) = calc. 148.9 [OTf]-, found 148.8 [OTf]-
m/z (+) = calc. 471.0 [M-OTf]+, found 471.0 [M-OTf]+

CHNS [%]: calc.: C: 43.23, H: 4.93, S: 9.23 (+Et_2O)
found: C: 48.63, H: 4.93, S: 6.91 (+Et_2O)

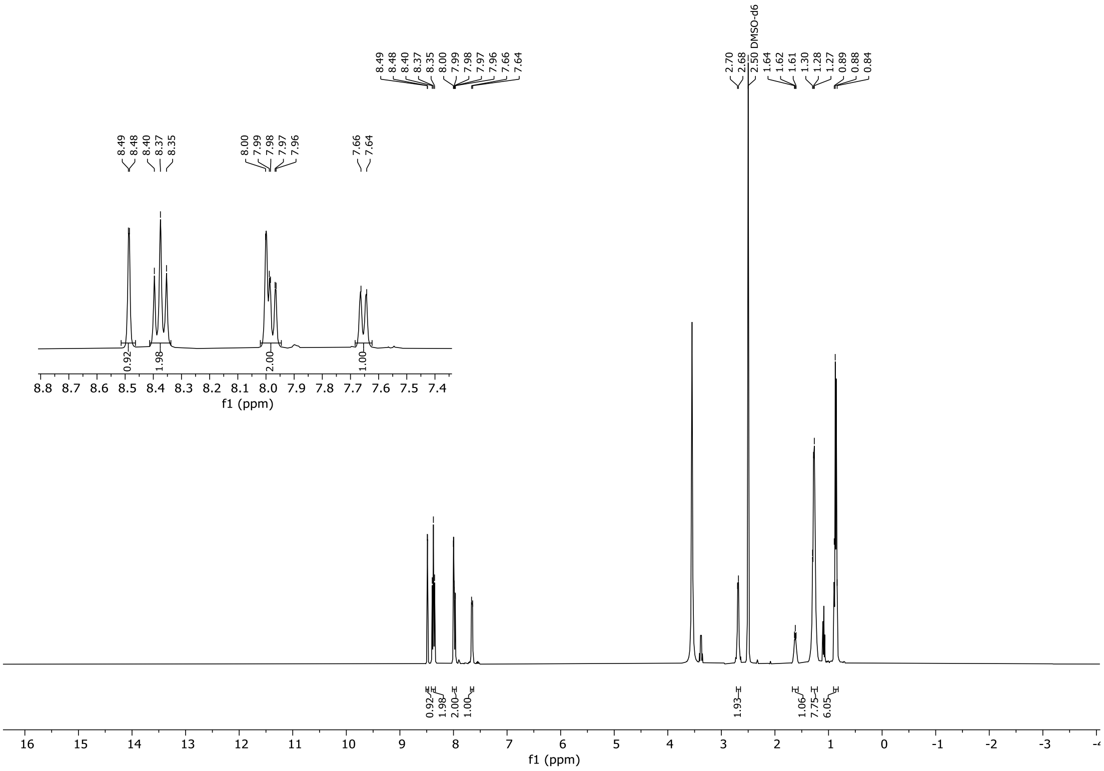

Figure S95: ^{1}H NMR spectrum (400 MHz) of compound **S21** in DMSO-d_6.

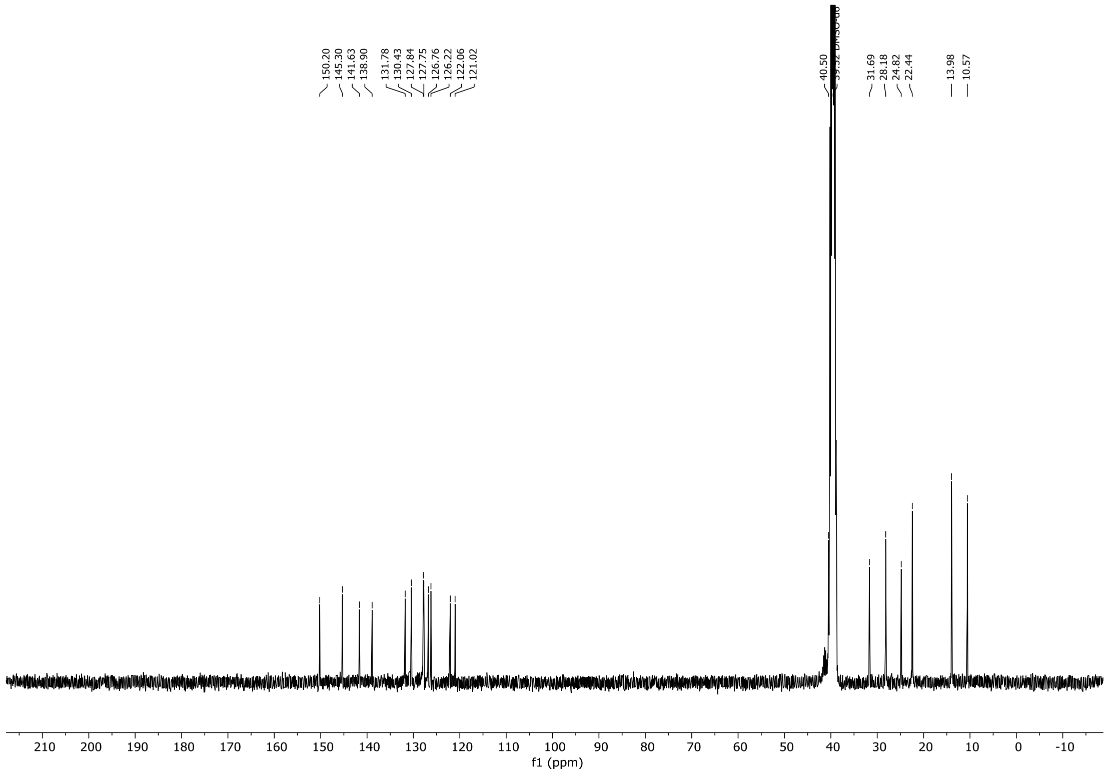

Figure S96: ^{13}C NMR spectrum (101 MHz) of compound **S21** in DMSO-d_6.

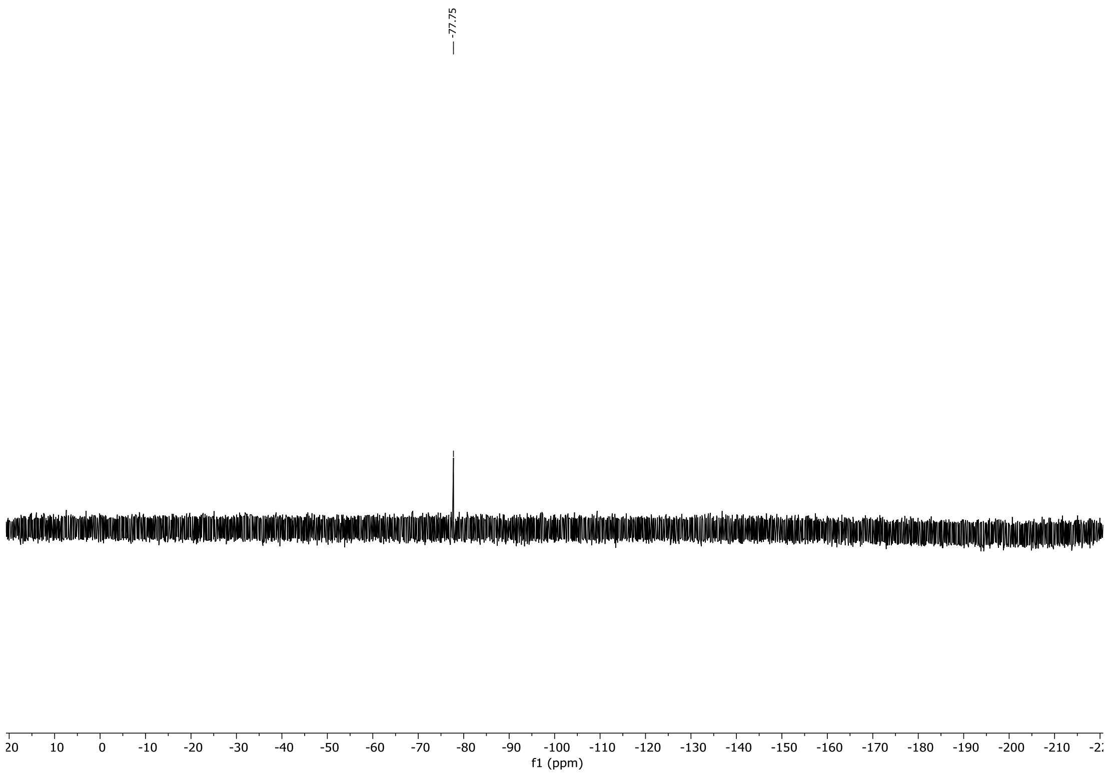

Figure S97: ^{19}F NMR spectrum (376 MHz) of compound **S21** in DMSO-d_6.

Synthesis of 3-(3,5-di-tert-butylphenyl)-7-sulfodibenzo[b,d]iodol-5-ium trifluoromethanesulfonate (S22)

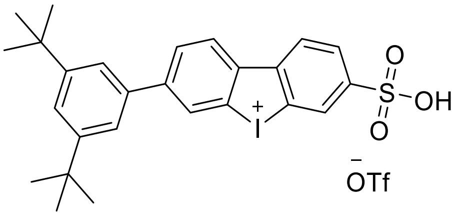

Chemical Formula: $C_{27}H_{28}F_3IO_6S_2$
Molecular Weight: 696,5347

For the oxidation according to the given procedure,[13] 242 mg (0.441 mmol, 1.0 eq.) of iodo compound **S20** were reacted with 114 mg (0.662 mmol, 1.5 eq.) mCPBA and 0.12 ml (1.32 mmol, 3.0 eq.) HOTf. After workup, iodolium salt **S22** was obtained as an off-white solid (132 mg, 43%).

^{1}H NMR (300 MHz, DMSO-*d6*): δ = 8.56 – 8.45 (m, 4H), 8.17 (dd, *J* = 8.3, 1.7 Hz, 1H), 8.02 (dd, *J* = 8.2, 1.6 Hz, 1H), 7.55 (d, *J* = 1.7 Hz, 2H), 7.52 (d, *J* = 1.8 Hz, 1H), 1.37 (s, 18H) ppm.[20]

^{19}F NMR (376 MHz, DMSO-*d6*): δ = -77.74 ppm.

ATR-IR [cm^{-1}]: 500.53 (s), 562.25 (s), 597.93 (s), 620.11 (vw), 633.62 (s), 652.90 (w), 702.09 (m), 778.28 (vs), 822.64 (m), 875.68 (m), 894.01 (vs), 1016.49 (vw), 1030.95 (vw), 1113.89 (w), 1167.90 (vw), 1229.62 (w), 1238.30 (w), 1362.71 (vs), 1372.35 (vs), 1593.20 m), 2952.05 (s).

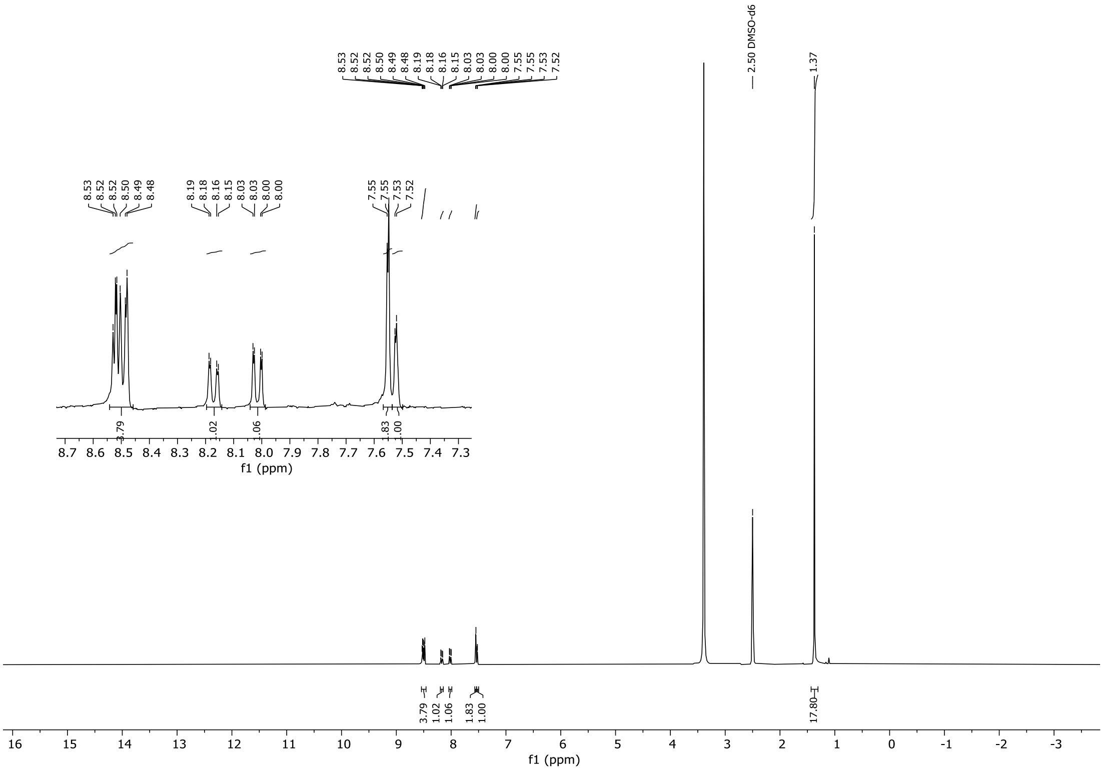

Figure S98: ^{1}H NMR spectrum (300 MHz) of compound **S22** in DMSO-*d6*.

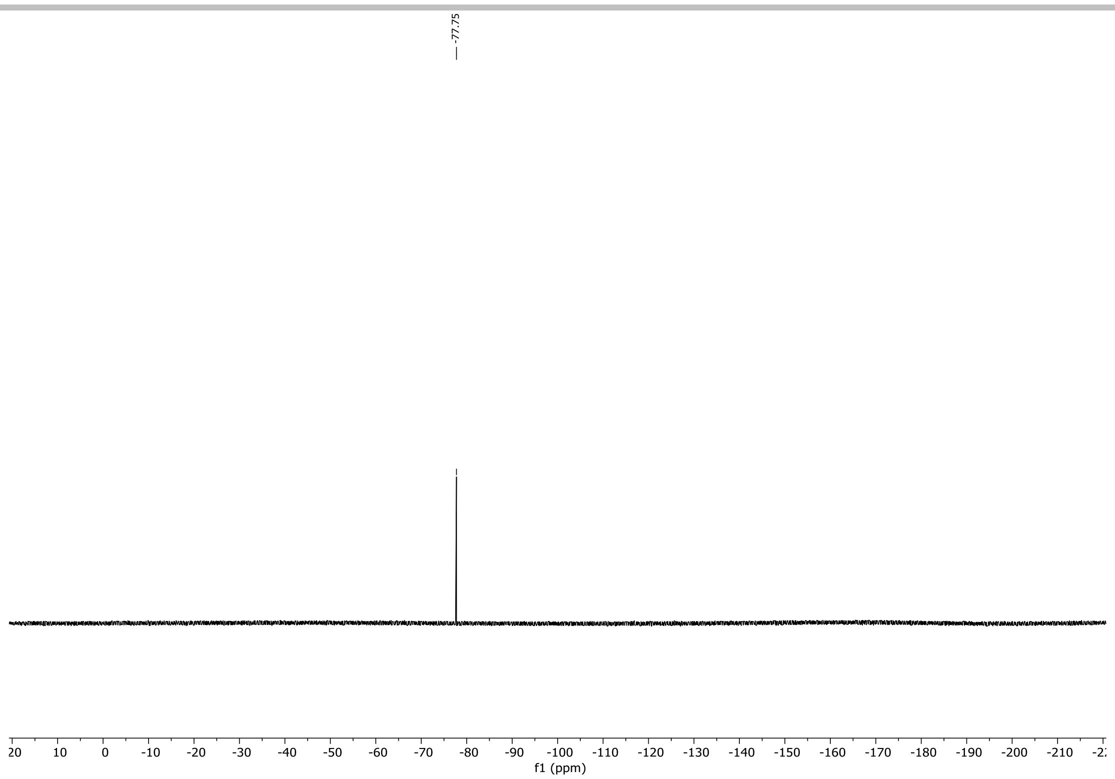

Figure S99: ^{19}F NMR spectrum (376 MHz) of compound **S22** in DMSO-d_6.

General deprotonation procedure to zwitterions 30 and 31

The iodolium salt (1.0 eq.) was suspended in pyridine (0.1 M) and stirred at r.t. over night. Then, the product was isolated via filtration and washed with water, EtOAc and DEE.

Synthesis of 7-(2-ethylhexyl)dibenzo[b,d]iodol-5-ium-3-sulfonate (30)

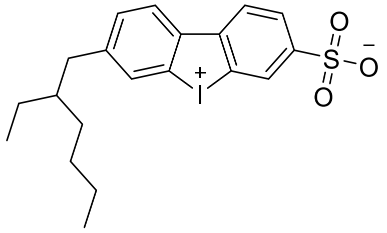

Chemical Formula: $C_{20}H_{23}IO_3S$
Molecular Weight: 470,3655

The iodolium salt **S21** (126 mg, 0.203 mmol, 1.0 eq.) was suspended in pyridine (0.1 M). After stirring overnight and working up, the product was isolated as off-white solid (74 mg, 98%).

^{1}H NMR (400 MHz, DMSO-d_6): δ = 8.49 (s, 1H), 8.38 (t, J = 8.5 Hz, 2H), 8.02 – 7.94 (m, 2H), 7.66 (d, J = 6.8 Hz, 1H), 2.69 (d, J = 4.5 Hz, 2H), 1.66 – 1.58 (m, 1H), 1.34 – 1.21 (m, 8H), 0.92 – 0.82 (m, 6H) ppm.[14]

ATR-IR [cm^{-1}]: 1456.74 (vs), 1375.25 (vs), 1225.28 (m), 1171.27 (vw), 1110.52 (m), 1031.43 (vw), 1016.97 (w), 828.91 (s), 668.33 (s), 650.49 (w), 618.19 (m), 598.90 (m), 565.63 (s), 560.80 (m), 452.79 (vs), 420.00 (s).

LID-MS: m/z (+) = calc.: 471.049 [M+H]+, found: 471.063 [M+H]+
calc.: 941.090 [2M+H]+, found: 941.082 [2M+H]+
calc.: 1411.131 [3M+H]+, found: 1411.107 [3M+H]+

CHNS [%]: calc.: C: 51.07, H: 4.93, N: 0.00, S: 6.82
found: C: 52.24, H: 5.24, N: 0.18, S: 6.06

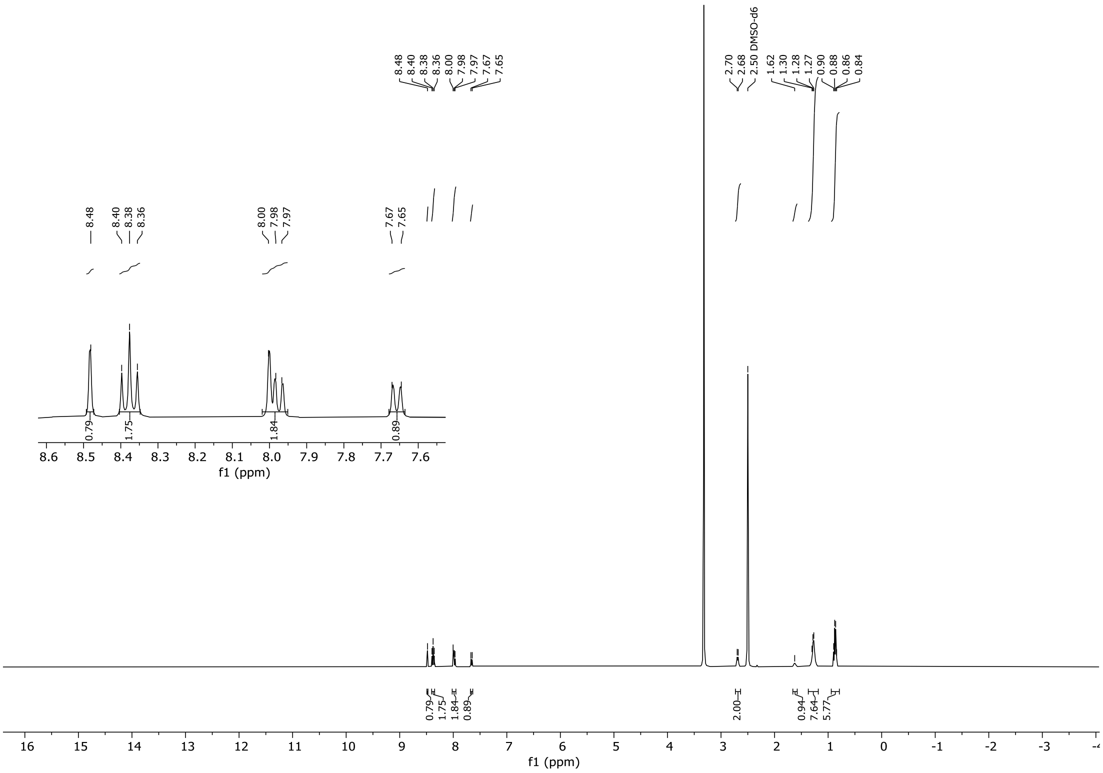

Figure S100: ^{1}H NMR (400 MHz) of compound **30** in DMSO-d_6.

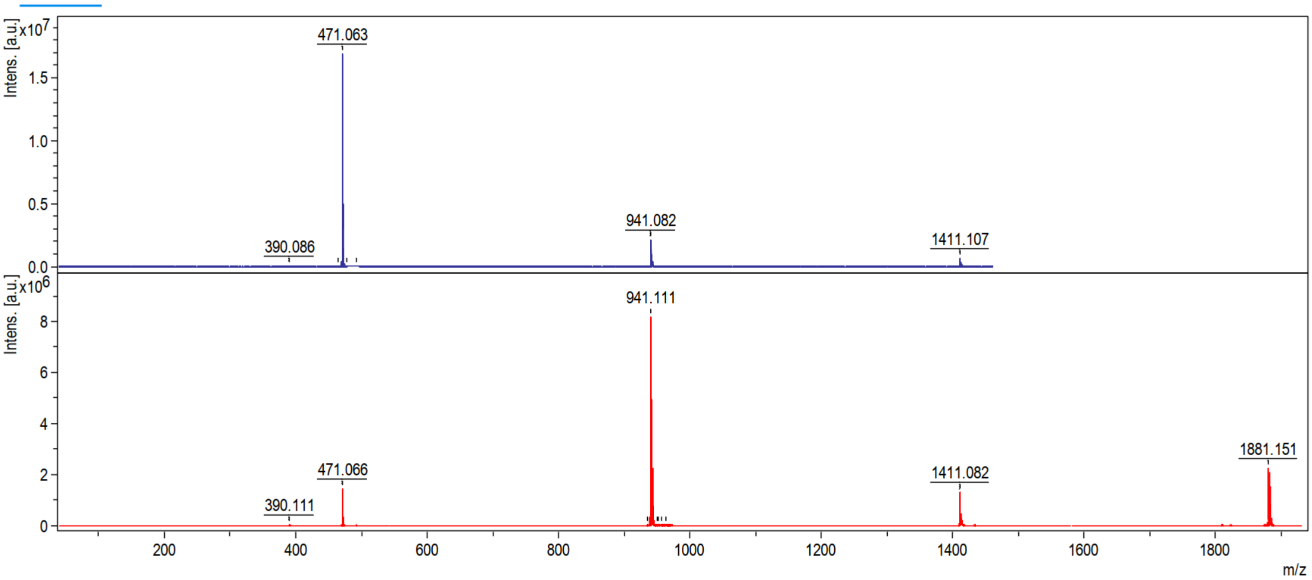

Figure S101: LID-MS spectra of compound **30** showing the monomer (471.063 m/z), dimer (941.062 m/z), trimer (1411.107 m/z) and tetramer (1881.151 m/z).

Synthesis of 7-(3,5-di-tert-butylphenyl)dibenzo[b,d]iodol-5-ium-3-sulfonate (31)

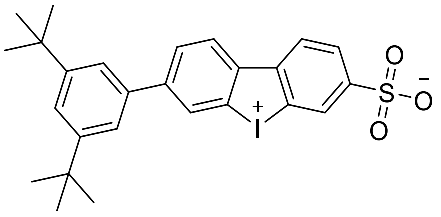

Chemical Formula: $C_{26}H_{27}IO_3S$
Molecular Weight: 546,4635

The iodolium salt **S22** (109 mg, 0.156 mmol, 1.0 eq.) was suspended in pyridine (0.1 M). After stirring overnight and working up, the product was isolated as off-white solid (40 mg, 47%).

^{1}H NMR (400 MHz, DMSO-d_6): δ = 8.54 – 8.50 (m, 2H), 8.48 (s, 1H), 8.17 (d, J = 8.4 Hz, 1H), 8.02 (d, J = 8.0 Hz, 1H), 7.60 – 7.50 (m, 4H), 1.37 (s, 18H) ppm.[20]

ATR-IR [cm^{-1}]: 1593.68 (vs), 1238.78 (m), 1230.10 (m), 1180.44 (m), 1167.90 (vw), 1139.93 (s), 1113.41 (m), 1031.43 (w), 1016.49 (w), 876.16 (m), 822.64 (m), 702.57 (m), 653.39 (w), 633.13 (vs), 619.63 (vw), 608.54 (vs), 605.65 (vs), 597.93 (m), 562.73 (s), 501.49 (m).

CID-MS: m/z (+) = calc.: 547.080 [M+H]+, found: 547.077 [M+H]+
calc.: 1093.2 [2M+H]+, found: 1094.0 [2M+H]+

CHNS [%]: calc.: C: 57.15, H: 4.98, N: 0.00, S: 5.87
found: C: 56.22, H: 4.86, N: 0.23, S: 5.35

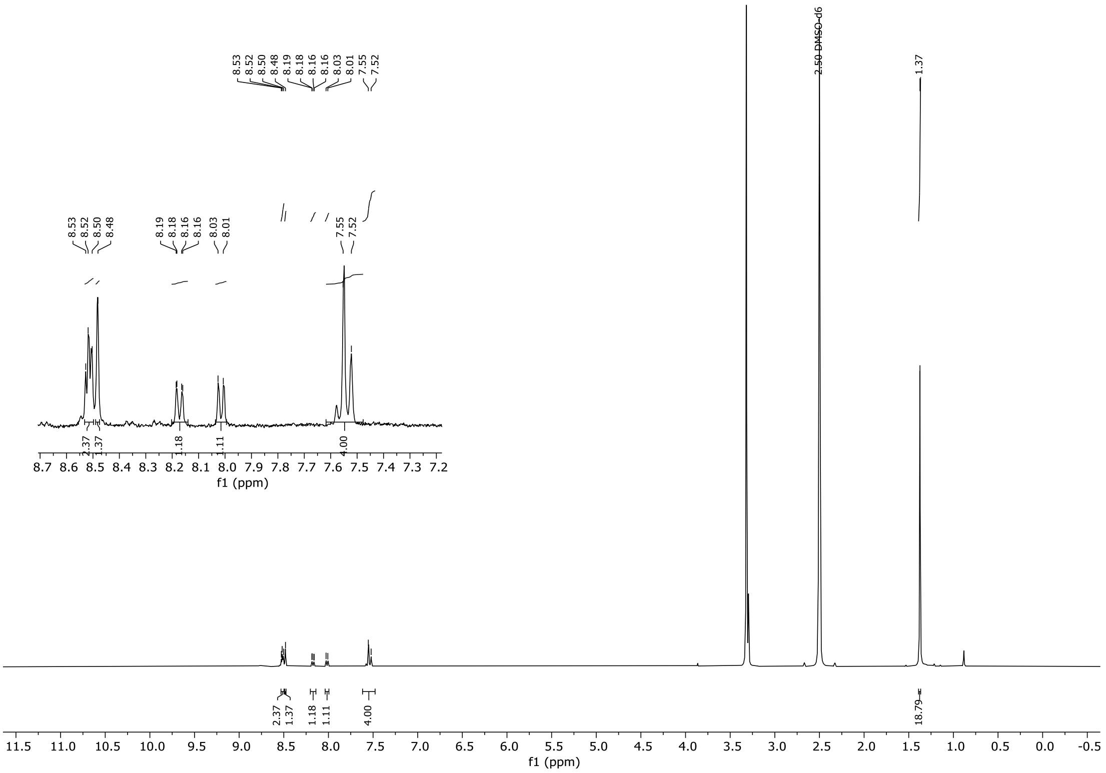

Figure S102: ^{1}H NMR spectrum (400 MHz) of compound **31** in DMSO-d_6.

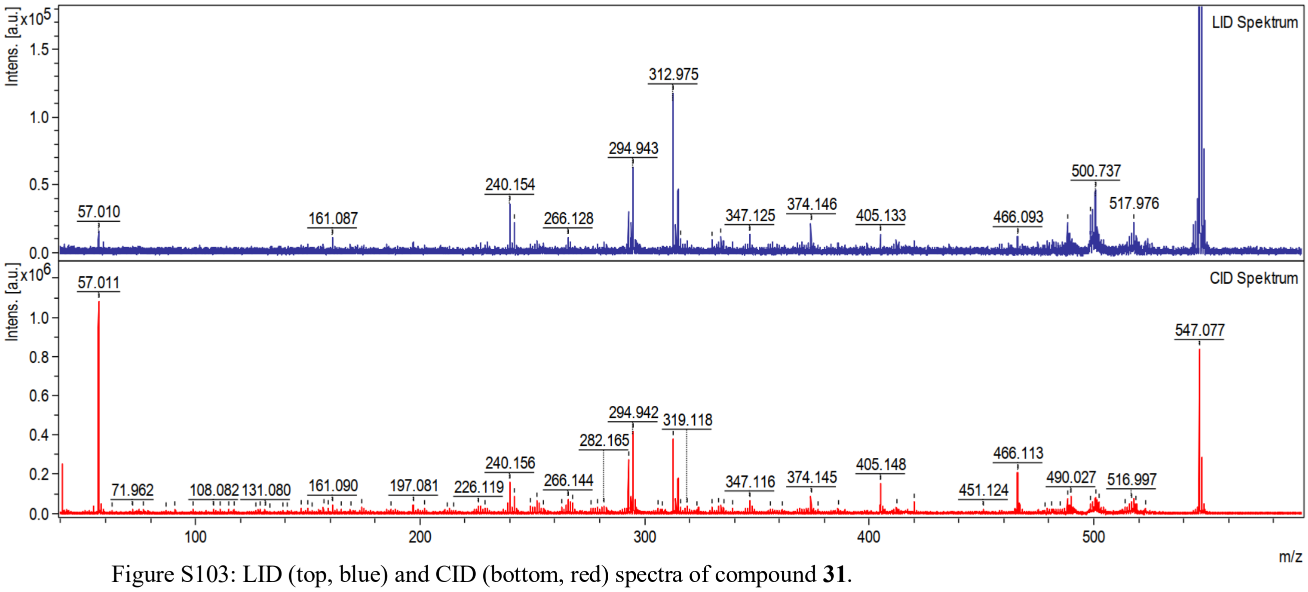

Figure S103: LID (top, blue) and CID (bottom, red) spectra of compound **31**.

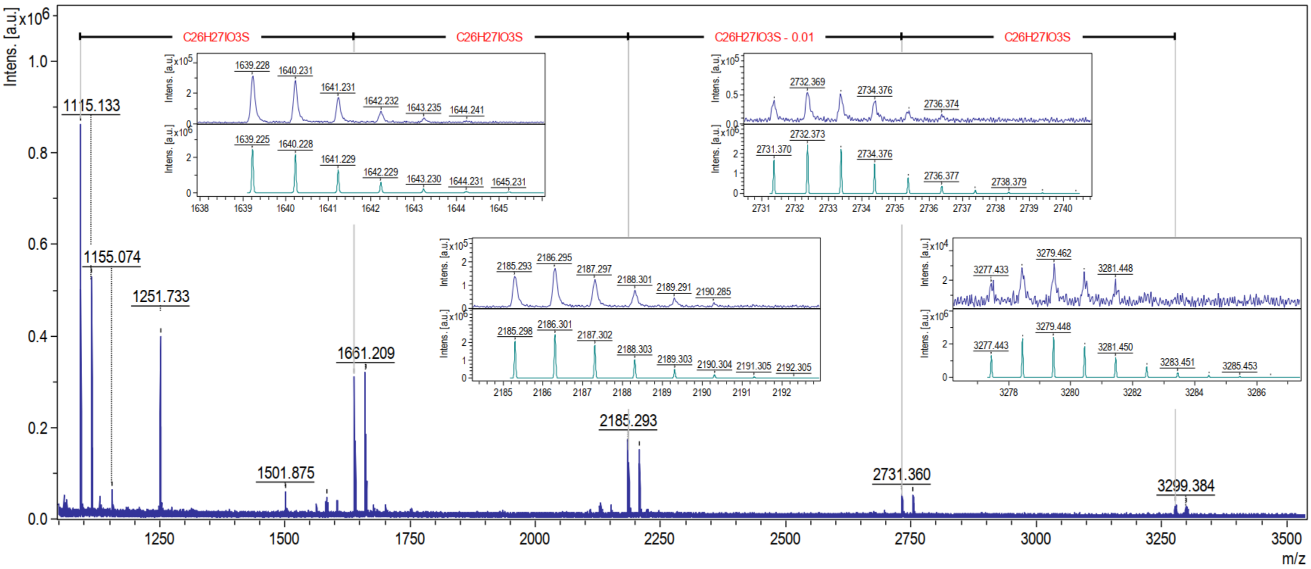

Figure S104: Mass spectrum of compound **31** showing the dimer (1115.133 m/z), dimer (1661.209 m/z), trimer (2185.293 m/z) and higher clusters. In the boxes, the measured isotope pattern (top, blue) is compared to the simulated isotope pattern (bottom, turquoise) which are in good agreement.

2. X-Ray Data

2.1 Zwitterion 1

Table S1. Crystal data and structure refinement for compound **1**.

Compound	**1**
CCDC No.	2166084
Empirical formula	$C_{18}H_{21}IO_4S_2$
Formula weight [g/mol]	492.37
Crystal system	Monoclinic
Space group	$P2_1/n$ (14)
Lattice parameters [Å]	
a	17.249(2)
b	6.0048(8)
c	18.479(3)
a	90
b	91.764(13)
g	90
Density [g/cm^3]	1.709
Crystal size [mm^3]	0.403 x 0.128 x 0.079
Volume [Å^3]	1913.1(5)
Z	4
Temperature [K]	170(2)
Diffraction Device	XtaLAB Mini (ROW)
Radiation Type	0.71073 Å (Mo K/ fine-focus sealed X-ray tube)
F(000)	984
Absorption coefficient [mm^{-1}]	1.912
Absorption correction	Analytical
Measurement range	2.2 - 25.0
Index range	-10 < h < 20
	-6 < k < 7
	-20 < l < 21
Measured reflexes	6827
Independent	3358
Observed	2317
R(int)	0.0794
Completeness (%) / theta (°)	99.9 / 24.996
Transmission (min / max)	0.726 / 0.894
R1 (observed/all)	0.0622 / 0.0884
wR2 (observed/all)	0.1480 / 0.1798
GooF = S	1.033
Rest electron density max./min. [e-/Å³]	-1.827 / 1.416

Table S2: Atomic coordinates and equivalent isotropic displacement parameters [Å^2] for **1**. U(eq) is defined as one third of the trace of the orthogonalized Uij tensor.

	x	y	z	U(eq)
I(1)	0.51744(3)	0.26572(8)	0.30715(3)	0.0305(2)
S(1)	0.61174(12)	0.1953(3)	0.61216(10)	0.0299(5)
O(1)	0.5366(4)	0.0824(11)	0.6140(3)	0.0506(17)
O(2)	0.6758(4)	0.0410(9)	0.6040(3)	0.0462(16)
O(3)	0.6250(4)	0.3521(9)	0.6710(3)	0.0435(16)
C(1)	0.6070(5)	0.3553(12)	0.5306(4)	0.0293(18)
C(2)	0.6449(5)	0.5528(13)	0.5253(4)	0.0309(18)
C(3)	0.6487(5)	0.6638(14)	0.4586(5)	0.036(2)
C(4)	0.6147(5)	0.5693(13)	0.3963(4)	0.0291(18)
C(5)	0.5743(4)	0.3718(13)	0.4036(4)	0.0291(18)
C(6)	0.5692(5)	0.2567(11)	0.4697(4)	0.0264(18)
C(7)	0.6204(4)	0.6539(13)	0.3215(4)	0.0300(18)
C(8)	0.5775(5)	0.5417(13)	0.2672(4)	0.0320(19)
C(9)	0.5784(5)	0.5988(13)	0.1941(4)	0.0341(19)
C(10)	0.6255(5)	0.7760(12)	0.1719(5)	0.033(2)
C(11)	0.6695(5)	0.8844(16)	0.2256(5)	0.041(2)
C(12)	0.6680(5)	0.8298(14)	0.2985(5)	0.036(2)
C(13)	0.6305(5)	0.8362(14)	0.0915(4)	0.035(2)
C(14)	0.7020(6)	0.7128(15)	0.0608(5)	0.044(2)
C(15)	0.5575(6)	0.7567(14)	0.0479(5)	0.042(2)
C(16)	0.6375(6)	1.0890(15)	0.0808(5)	0.048(2)
S(2)	0.37239(14)	0.2471(4)	0.14766(12)	0.0376(6)
O(4)	0.4571(4)	0.2154(9)	0.1701(3)	0.0403(15)
C(17)	0.3555(5)	0.5402(15)	0.1448(5)	0.050(2)
C(18)	0.3165(5)	0.1826(16)	0.2242(5)	0.044(2)
H(2)	0.669137	0.616646	0.567211	0.037
H(3)	0.674500	0.803201	0.455869	0.044
H(6)	0.541764	0.120066	0.473183	0.032
H(9)	0.547490	0.518732	0.159526	0.041
H(11)	0.702463	1.002411	0.211494	0.049
H(12)	0.698914	0.910587	0.332869	0.043
H(14A)	0.706172	0.748121	0.009273	0.066
H(14B)	0.695513	0.551782	0.066676	0.066
H(14C)	0.749244	0.761185	0.087106	0.066
H(15A)	0.559820	0.810343	-0.002027	0.062
H(15B)	0.510980	0.815743	0.070193	0.062
H(15C)	0.555670	0.593539	0.048069	0.062
H(16A)	0.641691	1.121822	0.029097	0.072
H(16B)	0.683825	1.143703	0.107144	0.072
H(16C)	0.591411	1.162699	0.099225	0.072
H(17A)	0.299760	0.568499	0.138129	0.075
H(17B)	0.383097	0.605306	0.104309	0.075
H(17C)	0.374307	0.607830	0.190264	0.075
H(18A)	0.261408	0.176596	0.209568	0.066

H(18B)	0.324497	0.297685	0.261250	0.066

Table S3: Anisotropic displacement parameters [Å^2] for **1**. The anisotropic displacement factor exponent takes the form: $-2\pi^2$[h a*2U^{11} + ...+ 2 h k a* b* U^{12}]

	U^{11}	U^{22}	U^{33}	U^{23}	U^{13}	U^{12}
I(1)	0.0276(4)	0.0440(4)	0.0194(3)	0.0041(2)	-0.0075(2)	-0.0029(2)
S(1)	0.0332(12)	0.0389(11)	0.0169(11)	0.0023(8)	-0.0105(8)	-0.0012(9)
O(1)	0.048(4)	0.076(4)	0.028(3)	0.022(3)	-0.012(3)	-0.027(4)
O(2)	0.050(4)	0.054(3)	0.034(4)	-0.001(3)	-0.011(3)	0.019(3)
O(3)	0.067(5)	0.040(3)	0.023(3)	-0.004(3)	-0.010(3)	0.002(3)
C(1)	0.031(5)	0.029(4)	0.028(4)	0.000(3)	-0.009(3)	0.003(4)
C(2)	0.030(5)	0.044(4)	0.019(4)	-0.006(4)	0.002(3)	-0.006(4)
C(3)	0.033(5)	0.041(4)	0.035(5)	-0.007(4)	-0.008(4)	-0.005(4)
C(4)	0.031(5)	0.033(4)	0.023(4)	0.002(3)	0.001(3)	0.006(4)
C(5)	0.026(4)	0.045(5)	0.015(4)	0.006(3)	-0.009(3)	-0.001(4)
C(6)	0.024(5)	0.030(4)	0.026(5)	-0.005(3)	-0.005(3)	-0.004(3)
C(7)	0.020(4)	0.042(4)	0.027(5)	0.006(4)	-0.009(3)	0.001(4)
C(8)	0.024(4)	0.040(4)	0.032(5)	0.002(4)	-0.004(3)	-0.001(4)
C(9)	0.030(5)	0.042(4)	0.030(5)	0.004(4)	-0.009(4)	-0.003(4)
C(10)	0.035(5)	0.034(4)	0.029(5)	0.011(4)	-0.002(4)	0.003(4)
C(11)	0.031(5)	0.060(6)	0.031(5)	0.007(4)	-0.006(4)	0.007(4)
C(12)	0.040(5)	0.039(4)	0.029(5)	-0.004(4)	-0.012(4)	-0.012(4)
C(13)	0.034(5)	0.048(5)	0.023(5)	0.013(4)	-0.008(4)	-0.006(4)
C(14)	0.040(6)	0.070(6)	0.021(5)	-0.001(4)	-0.005(4)	0.002(5)
C(15)	0.038(6)	0.070(6)	0.017(5)	0.014(4)	0.000(4)	-0.009(4)
C(16)	0.055(6)	0.059(6)	0.030(5)	0.012(4)	-0.002(4)	0.009(5)
S(2)	0.0382(13)	0.0561(14)	0.0180(11)	-0.0015(9)	-0.0075(9)	-0.0039(10)
O(4)	0.038(4)	0.059(4)	0.023(3)	-0.003(3)	-0.007(3)	0.003(3)
C(17)	0.038(6)	0.073(6)	0.038(6)	0.008(5)	-0.013(4)	0.001(5)
C(18)	0.029(5)	0.070(6)	0.032(5)	0.011(5)	-0.005(4)	-0.012(5)

2.2 Zwitterion 2

Single crystal obtained by slow evaporation of DMSO

Table S4: Crystal data and structure refinement for compound **2**.

Compound	**2**
CCDC No.	2522401
Empirical formula	C20 H25 I O4 S
Formula weight [g/mol]	488.36
Crystal system	Triclinic
Space group	P-1 (2)
Lattice parameters [Å]	
a	5.8608(4)
b	9.5638(7)
c	17.8231(14)
a	101.714(6)

b	98.323(6)
g	96.209(6)
Density [g/cm3]	1,693
Crystal size [mm3]	0.383 x 0.142 x 0.050
Volume [Å3]	958.10(12)
Z	2
Temperature [K]	170(2)
Diffraction Device	XtaLAB Mini (ROW)
Radiation Type	0.71073 Å (Mo K/ fine-focus sealed X-ray tube)
F(000)	492
Absorption coefficient [mm-1]	1.803
Absorption correction	Gaussian
Measurement range	02/03/2025
Index range	-6 < h < 6
	-11 < k < 10
	-19 < l < 21
Measured reflexes	5096
Independent	3357
Observed	2843
R(int)	0.0353
Completeness (%) / theta (°)	99.9 / 24.997
Transmission (min / max)	0.711 / 0.926
R1 (observed/all)	0.0485 / 0.0607
wR2 (observed/all)	0.1201 / 0.1296
GooF = S	1.056
Rest electron density max./min. [e-/Å³]	-0.903 / 1.793

Table S5: Atomic coordinates and equivalent isotropic displacement parameters [Å^2] for **2**. U(eq) is defined as one third of the trace of the orthogonalized Uij tensor.

	x	y	z	U(eq)
I(1)	0.91018(6)	0.84773(4)	0.35481(2)	0.02297(17)
S(1)	0.2785(3)	0.80814(16)	0.57166(9)	0.0225(4)
O(1)	0.4721(8)	0.8878(6)	0.6293(3)	0.0397(12)
O(2)	0.1323(7)	0.9043(4)	0.5420(2)	0.0241(9)
O(3)	0.1473(9)	0.6932(5)	0.5958(3)	0.0387(12)
C(1)	0.6624(10)	0.7424(6)	0.4050(3)	0.0198(13)
O(4)	0.8885(9)	0.8314(7)	0.7086(3)	0.0458(14)
C(2)	0.5786(10)	0.8071(6)	0.4699(3)	0.0205(13)
C(3)	0.3997(10)	0.7264(6)	0.4908(3)	0.0198(13)
C(4)	0.3118(11)	0.5868(6)	0.4493(4)	0.0240(14)
C(5)	0.4048(11)	0.5254(7)	0.3862(4)	0.0278(15)
C(6)	0.5857(11)	0.6034(6)	0.3622(4)	0.0212(13)
C(7)	0.6899(11)	0.5526(6)	0.2937(4)	0.0215(13)
C(8)	0.6269(10)	0.4153(6)	0.2465(3)	0.0205(13)
C(9)	0.7174(10)	0.3766(6)	0.1799(4)	0.0199(13)
C(10)	0.8737(10)	0.4788(6)	0.1607(4)	0.0222(13)
C(11)	0.9475(10)	0.6182(6)	0.2056(4)	0.0204(13)

C(12)	0.8515(10)	0.6493(6)	0.2732(3)	0.0179(12)
C(13)	0.6423(11)	0.2301(6)	0.1233(4)	0.0235(14)
C(14)	0.5274(13)	0.1198(7)	0.1624(4)	0.0329(16)
C(15)	0.4649(12)	0.2520(8)	0.0559(4)	0.0348(17)
C(16)	0.8479(12)	0.1722(7)	0.0914(4)	0.0324(16)
C(17)	1.1080(10)	0.7247(6)	0.1760(4)	0.0232(14)
C(18)	1.2924(12)	0.8162(8)	0.2402(4)	0.0347(17)
C(19)	0.9586(13)	0.8206(9)	0.1399(5)	0.049(2)
C(20)	1.2349(16)	0.6488(8)	0.1153(5)	0.057(3)
HO1	0.775(10)	0.836(11)	0.679(5)	0.069
HO2	0.963(14)	0.782(9)	0.682(5)	0.069
H(2)	0.640846	0.901965	0.498438	0.025
H(4)	0.186928	0.533714	0.464624	0.029
H(5)	0.345851	0.429424	0.35868	0.033
H(8)	0.519715	0.347381	0.260588	0.025
H(10)	0.933972	0.451764	0.113991	0.027
H(14A)	0.382584	0.150196	0.177144	0.049
H(14B)	0.633362	0.113129	0.209	0.049
H(14C)	0.492419	0.025349	0.12615	0.049
H(15A)	0.538288	0.321066	0.029729	0.052
H(15B)	0.331376	0.289288	0.07617	0.052
H(15C)	0.411803	0.159552	0.018702	0.052
H(16A)	0.79542	0.077347	0.056458	0.049
H(16B)	0.965594	0.163035	0.134534	0.049
H(16C)	0.915557	0.238671	0.062712	0.049
H(18A)	1.409617	0.867102	0.217524	0.052
H(18B)	1.367015	0.75437	0.271	0.052
H(18C)	1.220429	0.886579	0.273803	0.052
H(19A)	1.057994	0.893306	0.123032	0.073
H(19B)	0.871845	0.868681	0.178189	0.073
H(19C)	0.84896	0.762105	0.094853	0.073
H(20A)	1.123833	0.606193	0.067321	0.085
H(20B)	1.308381	0.572538	0.134427	0.085
H(20C)	1.35471	0.718412	0.104686	0.085

Table S6: Anisotropic displacement parameters [Å^2] for **2**. The anisotropic displacement factor exponent takes the form: $-2\pi^2$[h a*2U^{11} + ...+ 2 h k a* b* U^{12}]

	U^{11}	U^{22}	U^{33}	U^{23}	U^{13}	U^{12}
I(1)	0.0249(3)	0.0180(2)	0.0239(3)	0.00087(16)	0.00981(17)	-0.00602(16)
S(1)	0.0278(8)	0.0213(7)	0.0202(8)	0.0050(6)	0.0096(6)	0.0027(6)
O(1)	0.036(3)	0.055(3)	0.022(3)	-0.003(2)	0.001(2)	0.011(2)
O(2)	0.029(2)	0.020(2)	0.025(2)	0.0041(18)	0.0110(19)	0.0054(18)
O(3)	0.059(3)	0.027(2)	0.040(3)	0.016(2)	0.031(3)	0.007(2)
C(1)	0.018(3)	0.020(3)	0.021(3)	0.005(3)	0.005(2)	-0.001(2)
O(4)	0.037(3)	0.062(4)	0.037(3)	0.003(3)	0.009(2)	0.013(3)
C(2)	0.022(3)	0.019(3)	0.019(3)	0.004(3)	0.004(3)	-0.001(2)

C(3)	0.023(3)	0.019(3)	0.018(3)	0.005(2)	0.004(2)	0.005(2)
C(4)	0.028(3)	0.021(3)	0.025(3)	0.006(3)	0.011(3)	0.000(3)
C(5)	0.030(4)	0.020(3)	0.034(4)	0.005(3)	0.010(3)	-0.001(3)
C(6)	0.026(3)	0.017(3)	0.020(3)	0.002(2)	0.006(3)	-0.002(3)
C(7)	0.025(3)	0.014(3)	0.027(3)	0.006(3)	0.008(3)	0.002(2)
C(8)	0.023(3)	0.018(3)	0.020(3)	0.004(2)	0.007(3)	-0.003(2)
C(9)	0.017(3)	0.020(3)	0.025(3)	0.010(3)	0.002(3)	0.004(2)
C(10)	0.024(3)	0.021(3)	0.020(3)	0.002(3)	0.010(3)	-0.005(3)
C(11)	0.019(3)	0.018(3)	0.025(3)	0.005(3)	0.007(3)	0.001(2)
C(12)	0.024(3)	0.012(3)	0.015(3)	0.001(2)	0.002(2)	0.000(2)
C(13)	0.027(3)	0.019(3)	0.022(3)	0.000(3)	0.010(3)	-0.003(3)
C(14)	0.044(4)	0.023(3)	0.029(4)	0.002(3)	0.013(3)	-0.010(3)
C(15)	0.035(4)	0.034(4)	0.029(4)	0.003(3)	0.000(3)	-0.006(3)
C(16)	0.031(4)	0.022(3)	0.043(4)	0.000(3)	0.016(3)	-0.001(3)
C(17)	0.025(3)	0.022(3)	0.023(3)	0.006(3)	0.010(3)	-0.005(3)
C(18)	0.027(4)	0.039(4)	0.037(4)	0.015(3)	0.004(3)	-0.011(3)
C(19)	0.035(4)	0.057(5)	0.061(6)	0.042(5)	-0.001(4)	-0.006(4)
C(20)	0.082(6)	0.028(4)	0.059(6)	-0.006(4)	0.053(5)	-0.023(4)

Single crystal obtained by slow evaporation of MeOH

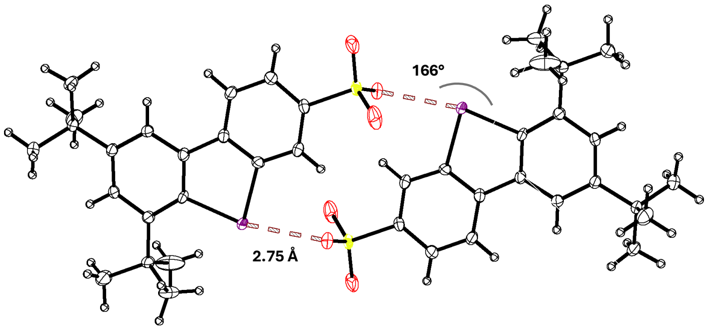

Figure S105: X-ray structural analysis of the self-assembled dimer of compound **2** obtained by the slow evaporation of MeOH. Carbon atoms are shown in grey, iodine in purple, hydrogen in white, oxygen in red, and sulphur in yellow. Halogen bonds are highlighted in red including their bond angles and binding distances. Thermal ellipsoids at 50% probability level.

Table S7: Crystal data and structure refinement for compound **2**.

Compound	**2**
CCDC No.	2522402
Empirical formula	C20 H25 I O4 S
Formula weight [g/mol]	488.36
Crystal system	Triclinic
Space group	P-1 (2)
Lattice parameters [Å]	
a	5.8797(3)
b	9.5774(3)
c	17.8053(6)
a	101.636(3)

b	98.375(4)
g	96.296(4)
Density [g/cm3]	1.687
Crystal size [mm3]	0.451 x 0.391 x 0.195
Volume [Å3]	961.52(7)
Z	2
Temperature [K]	175(2)
Diffraction Device	XtaLAB Mini (ROW)
Radiation Type	0.71073 Å (Mo K/ fine-f
F(000)	492
Absorption coefficient [mm-1]	1.797
Absorption correction	Gaussian
Measurement range	2.7 - 27.1
Index range	-7 < h < 7
	-12 < k < 12
	-22 < l < 22
Measured reflexes	20490
Independent	4264
Observed	4050
R(int)	0.036
Completeness (%) / theta (°)	99.9 / 25.242
Transmission (min / max)	0.613 / 0.983
R1 (observed/all)	0.0214 / 0.0233
wR2 (observed/all)	0.0515 / 0.0524
GooF = S	1.1
Rest electron density max./min. [e-/Å³]	-0.484 / 0.447

Table S8: Atomic coordinates and equivalent isotropic displacement parameters [Å^2] for **2**. U(eq) is defined as one third of the trace of the orthogonalized Uij tensor.

	x	y	z	U(eq)
I(1)	0.90856(2)	0.84766(2)	0.35502(2)	0.01816(5)
S(1)	0.27797(9)	0.80804(6)	0.57161(3)	0.01824(11)
O(1)	0.1331(3)	0.90486(16)	0.54164(9)	0.0216(3)
C(1)	0.3995(4)	0.7260(2)	0.49131(12)	0.0170(4)
O(2)	0.4717(3)	0.8872(2)	0.62932(10)	0.0361(4)
C(2)	0.3114(4)	0.5874(2)	0.44977(13)	0.0213(4)
O(3)	0.1474(4)	0.69336(18)	0.59510(11)	0.0365(4)
C(3)	0.4053(4)	0.5262(2)	0.38632(13)	0.0213(4)
O(4)	0.8877(4)	0.8328(3)	0.70797(12)	0.0445(5)
C(4)	0.5856(4)	0.6034(2)	0.36256(12)	0.0169(4)
C(5)	0.6621(3)	0.7429(2)	0.40517(12)	0.0170(4)
C(6)	0.5786(4)	0.8066(2)	0.46961(12)	0.0175(4)
C(7)	0.6886(4)	0.5526(2)	0.29384(12)	0.0164(4)
C(8)	0.6248(4)	0.4149(2)	0.24675(12)	0.0175(4)
C(9)	0.7169(4)	0.3769(2)	0.17970(12)	0.0169(4)

C(10)	0.8726(4)	0.4794(2)	0.16091(12)	0.0182(4)
C(11)	0.9466(4)	0.6178(2)	0.20570(12)	0.0163(4)
C(12)	0.8508(4)	0.6492(2)	0.27343(12)	0.0156(4)
C(13)	0.6406(4)	0.2306(2)	0.12338(12)	0.0186(4)
C(14)	0.5274(4)	0.1201(2)	0.16220(14)	0.0276(5)
C(15)	0.4668(4)	0.2522(3)	0.05584(14)	0.0313(5)
C(16)	0.8484(4)	0.1722(3)	0.09221(16)	0.0299(5)
C(17)	1.1048(4)	0.7250(2)	0.17649(12)	0.0190(4)
C(18)	0.9560(5)	0.8229(3)	0.1411(2)	0.0481(8)
C(19)	1.2329(7)	0.6485(3)	0.1156(2)	0.0578(10)
C(20)	1.2915(4)	0.8155(3)	0.24104(15)	0.0310(5)
H(6)	0.640547	0.90148	0.497998	0.021
H(8)	0.517827	0.346926	0.260745	0.021
H(10)	0.932563	0.452686	0.114135	0.022
H(14A)	0.485166	0.027171	0.12488	0.041
H(14B)	0.387411	0.152437	0.179574	0.041
H(14C)	0.636674	0.109574	0.207139	0.041
H(15A)	0.412489	0.159668	0.019014	0.047
H(15B)	0.541838	0.320068	0.029358	0.047
H(15C)	0.334261	0.290899	0.075728	0.047
H(16A)	0.798064	0.075361	0.05932	0.045
H(16B)	0.967736	0.167775	0.1359	0.045
H(16C)	0.912469	0.23591	0.061402	0.045
H(18A)	1.054628	0.892547	0.121986	0.072
H(18B)	0.877252	0.874501	0.180693	0.072
H(18C)	0.839944	0.765061	0.097692	0.072
H(19A)	1.346897	0.718941	0.10299	0.087
H(19B)	1.121286	0.601376	0.068451	0.087
H(19C)	1.312814	0.576035	0.136111	0.087
H(20A)	1.418365	0.856345	0.218348	0.046
H(20B)	1.35149	0.754978	0.275436	0.046
H(20C)	1.225254	0.893612	0.271044	0.046

Table S9: Anisotropic displacement parameters [Å^2] for **2**. The anisotropic displacement factor exponent takes the form: $-2\pi^2$[h a*2U^{11} + ...+ 2 h k a* b* U^{12}]

	U^{11}	U^{22}	U^{33}	U^{23}	U^{13}	U^{12}
I(1)	0.02152(8)	0.01449(8)	0.01550(8)	-0.00281(5)	0.00605(5)	-0.00263(5)
S(1)	0.0244(3)	0.0182(3)	0.0131(2)	0.00156(19)	0.0068(2)	0.0062(2)
O(1)	0.0271(8)	0.0186(8)	0.0190(8)	0.0008(6)	0.0049(6)	0.0081(6)
C(1)	0.0216(10)	0.0174(10)	0.0133(10)	0.0028(8)	0.0056(8)	0.0064(8)
O(2)	0.0303(9)	0.0533(12)	0.0175(9)	-0.0092(8)	-0.0017(7)	0.0140(9)
C(2)	0.0253(11)	0.0183(10)	0.0214(11)	0.0036(9)	0.0098(9)	0.0018(9)
O(3)	0.0603(12)	0.0234(9)	0.0376(10)	0.0120(8)	0.0348(9)	0.0126(8)
C(3)	0.0298(11)	0.0137(10)	0.0192(11)	-0.0001(8)	0.0085(9)	-0.0010(9)
O(4)	0.0364(11)	0.0614(14)	0.0315(11)	-0.0049(10)	0.0066(8)	0.0164(10)
C(4)	0.0220(10)	0.0144(10)	0.0152(10)	0.0027(8)	0.0050(8)	0.0050(8)
C(5)	0.0172(9)	0.0181(10)	0.0150(10)	0.0024(8)	0.0045(8)	-0.0002(8)

C(6)	0.0217(10)	0.0156(10)	0.0130(10)	-0.0007(8)	0.0015(8)	0.0026(8)
C(7)	0.0215(10)	0.0150(10)	0.0125(10)	0.0017(8)	0.0040(8)	0.0026(8)
C(8)	0.0210(10)	0.0146(10)	0.0171(10)	0.0027(8)	0.0064(8)	0.0004(8)
C(9)	0.0193(10)	0.0143(10)	0.0156(10)	0.0004(8)	0.0027(8)	0.0015(8)
C(10)	0.0232(10)	0.0172(10)	0.0141(10)	0.0000(8)	0.0084(8)	0.0014(8)
C(11)	0.0184(10)	0.0156(10)	0.0145(10)	0.0032(8)	0.0032(8)	0.0010(8)
C(12)	0.0204(10)	0.0104(9)	0.0139(10)	-0.0013(7)	0.0019(8)	0.0014(8)
C(13)	0.0213(10)	0.0150(10)	0.0171(10)	-0.0021(8)	0.0060(8)	-0.0008(8)
C(14)	0.0379(13)	0.0161(11)	0.0258(12)	-0.0020(9)	0.0117(10)	-0.0054(10)
C(15)	0.0329(13)	0.0295(13)	0.0243(13)	-0.0012(10)	-0.0031(10)	-0.0020(11)
C(16)	0.0300(12)	0.0199(11)	0.0376(14)	-0.0045(10)	0.0145(11)	0.0023(10)
C(17)	0.0224(10)	0.0164(10)	0.0177(10)	0.0030(8)	0.0067(8)	-0.0021(8)
C(18)	0.0307(14)	0.0554(19)	0.066(2)	0.0463(17)	-0.0055(14)	-0.0053(13)
C(19)	0.084(2)	0.0307(15)	0.056(2)	-0.0117(14)	0.0554(19)	-0.0215(16)
C(20)	0.0261(12)	0.0345(14)	0.0295(13)	0.0101(11)	0.0027(10)	-0.0109(10)

2.3 Zwitterion 14

Table S10: Crystal data and structure refinement for compound **14**.

Compound	**14**
CCDC No	2522403
Empirical formula	$C_{16}H_{19}IO_5S_2$
Formula weight [g/mol]	482.33
Crystal system	Orthorhombic
Space group	Pnma (62)
Lattice parameters [Å]	
a	13.79300(10)
b	6.91450(10)
c	18.4855(2)
a	90
b	90
g	90
Density [g/cm3]	1.817
Crystal size [mm3]	0.610 x 0.160 x 0.120
Volume [Å3]	1762.99(3)
Z	4
Temperature [K]	169.99(10)
Diffraction Device	XtaLAB Synergy, Dualflex, HyPix
Radiation Type	1.54184 Å (Cu K/ micro-focus sealed X-ray tube)
F(000)	960
Absorption coefficient [mm-1]	16.701
Absorption correction	Gaussian
Measurement range	4.0 - 66.5
Index range	-16 < h < 16
	-8 < k < 7

	-21 < l < 21
Measured reflexes	20169
Independent	1697
Observed	1663
R(int)	0.0833
Completeness (%) / theta (°)	100.0 / 66.461
Transmission (min / max)	0.006 / 0.542
R1 (observed/all)	0.0369 / 0.0373
wR2 (observed/all)	0.1015 / 0.1023
GooF = S	0.927
Rest electron density max./min. [e-/Å³]	-1.333 / 1.044

Table S11: Atomic coordinates and equivalent isotropic displacement parameters [Å^2] for **14**. U(eq) is defined as one third of the trace of the orthogonalized Uij tensor.

	x	y	z	U(eq)
O(1)	0.2508(3)	0.75	0.2914(2)	0.0399(8)
I(1)	0.32904(2)	0.75	0.44028(2)	0.02079(18)
S(2)	0.21111(8)	0.75	0.74198(5)	0.0292(3)
O(3)	0.15558(17)	0.5755(4)	0.72746(14)	0.0411(6)
O(4)	0.2581(3)	0.75	0.81183(18)	0.0464(9)
O(2)	0.1388(2)	0.75	0.48139(17)	0.0373(8)
C(1)	0.0456(3)	0.5530(5)	0.38067(19)	0.0464(8)
C(009)	0.4822(3)	0.75	0.44029(18)	0.0203(10)
C(10)	0.6807(3)	0.75	0.4578(3)	0.0262(10)
C(11)	0.6388(3)	0.75	0.3893(2)	0.0240(8)
C(12)	0.5391(3)	0.75	0.3781(2)	0.0224(8)
C(13)	0.4977(3)	0.75	0.3032(2)	0.0300(10)
C(14)	0.7887(3)	0.75	0.4664(3)	0.0336(10)
S(1)	0.04262(11)	0.75	0.44300(5)	0.0367(4)
C(2)	0.3511(4)	0.75	0.5527(2)	0.0222(9)
C(3)	0.2775(3)	0.75	0.6039(2)	0.0221(8)
C(4)	0.3055(4)	0.75	0.6754(3)	0.0238(9)
C(5)	0.4027(3)	0.75	0.6960(2)	0.0271(9)
C(6)	0.4740(3)	0.75	0.6437(2)	0.0239(8)
C(7)	0.4501(3)	0.75	0.5704(2)	0.0216(8)
C(8)	0.5193(3)	0.75	0.5104(2)	0.0204(8)
C(9)	0.6212(3)	0.75	0.5190(2)	0.0236(8)
H(1)	0.267(3)	0.651(4)	0.272(2)	0.06
H(1A)	-0.014733	0.550822	0.352663	0.07
H(1B)	0.052357	0.431225	0.407322	0.07
H(1C)	0.100759	0.568757	0.34779	0.07
H(11)	0.680257	0.75	0.348209	0.029
H(13A)	0.550186	0.733481	0.267944	0.045
H(13B)	0.464614	0.873095	0.294228	0.045
H(13C)	0.45135	0.643424	0.298302	0.045
H(14A)	0.817827	0.832782	0.429295	0.05
H(14B)	0.813247	0.617768	0.461151	0.05

H(14C)	0.80558	0.799451	0.514477	0.05
H(3)	0.211051	0.75	0.590283	0.027
H(5)	0.419777	0.75	0.745777	0.033
H(6)	0.540264	0.75	0.657811	0.029
H(9)	0.648937	0.75	0.565997	0.028

Table S12: Anisotropic displacement parameters [Å^2] for **14**. The anisotropic displacement factor exponent takes the form: -$2\pi^2$[h a*2U^{11} + ...+ 2 h k a* b* U^{12}]

	U^{11}	U^{22}	U^{33}	U^{23}	U^{13}	U^{12}
O(1)	0.045(2)	0.036(2)	0.0391(19)	0	-0.0016(16)	0
I(1)	0.0217(3)	0.0180(3)	0.0227(3)	0	-0.00185(7)	0
S(2)	0.0314(6)	0.0286(6)	0.0275(5)	0	0.0061(4)	0
O(3)	0.0385(12)	0.0371(15)	0.0475(14)	0.0039(11)	0.0091(10)	-0.0130(11)
O(4)	0.044(2)	0.067(3)	0.0284(16)	0	0.0061(14)	0
O(2)	0.0259(15)	0.053(2)	0.0334(16)	0	-0.0028(14)	0
C(1)	0.056(2)	0.0375(19)	0.0451(18)	0.0014(15)	-0.0113(15)	-0.0095(17)
C(009)	0.018(2)	0.015(2)	0.028(2)	0	0.0015(13)	0
C(10)	0.030(3)	0.009(2)	0.040(2)	0	0.0005(18)	0
C(11)	0.027(2)	0.0155(19)	0.030(2)	0	0.0057(18)	0
C(12)	0.030(2)	0.0100(18)	0.0276(19)	0	0.0015(16)	0
C(13)	0.031(2)	0.032(2)	0.027(2)	0	0.0019(16)	0
C(14)	0.024(2)	0.034(3)	0.042(3)	0	-0.001(2)	0
S(1)	0.0232(7)	0.0566(10)	0.0303(7)	0	-0.0020(3)	0
C(2)	0.027(2)	0.021(3)	0.0185(19)	0	-0.0024(15)	0
C(3)	0.023(2)	0.0159(19)	0.0274(18)	0	-0.0001(15)	0
C(4)	0.0265(19)	0.017(2)	0.028(2)	0	0.0054(18)	0
C(5)	0.034(2)	0.025(2)	0.0220(19)	0	-0.0019(16)	0
C(6)	0.0242(19)	0.0184(19)	0.029(2)	0	-0.0036(16)	0
C(7)	0.022(2)	0.014(2)	0.0279(18)	0	-0.0015(16)	0
C(8)	0.025(2)	0.0078(19)	0.028(2)	0	0.0012(16)	0
C(9)	0.024(2)	0.017(2)	0.0295(19)	0	-0.0051(16)	0

3. DOSY Experiments

Diffusion ordered NMR spectroscopy (DOSY) was performed on a 400 MHz *Agilent* MR400-DD2 equipped with a 5 mm OneNMR probe at 298.15 K in DMSO-d_6. The hydrodynamic radius r_H of compound **1** was calculated according to the Stokes Einstein equation for cigarshaped compounds (1) using the average of the obtained diffusion coefficients *D* from all DOSYs.

$$r_H = \frac{k_B \cdot T}{f_S \cdot c \cdot \pi \cdot \eta \cdot D \cdot 10^{-3}} \quad (1)$$

The equation takes the correction factors f_s (form factor for cigar shaped molecules)[21] and c (correction factor for small molecules)[22] into account with r as the van der Waals radius of the solvent/solute, a as the long diameter and b as the short diameter

$$c = \frac{6}{1 + 0.695\,(\frac{r_{solvent}}{r_{solute}})^{2.234}} \quad (2)$$

$$f_s = \frac{\sqrt{1-(\frac{b}{a})^2}}{(\frac{b}{a})^{\frac{2}{3}} \ln \frac{1+\sqrt{1-(\frac{b}{a})^2}}{(\frac{b}{a})}} \quad (3)$$

Table S10: Recorded DOSY diffusion coefficients (D) of **1** at different concentrations in DMSO-d_6. The signal of the tert-butyl group of **1** at 1.34 ppm was followed.

Concentration	ppm	D x 10^{-10} [$m^2 s^{-1}$]	r_H [Å]
20 mM	1.34	1.78 ±0.04	6.51
10 mM	1.34	1.75±0.07	6.63
6.7 mM	1.34	1.90±0.03	6.10

Table S11: Recorded DOSY diffusion coefficients (D) of **2** at different concentrations in DMSO-d_6. The signal of one tert-butyl group of **2** at 1.43 ppm was followed.

Concentration	ppm	D x 10^{-10} [$m^2 s^{-1}$]	r_H [Å]
20 mM	1.43	1.65 ±0.04	7.97
10 mM	1.43	1.70±0.07	7.73
6.7 mM	1.43	1.65±0.03	7.97

4. Computational Details

All DFT calculations were performed using Gausian16[23] and modeled with M06-2X density functional[24] including the Grimme D3 dispersion correction.[25,26] The used basis set was def2-TZVP for all atoms except halogens which were calculated using def2-TZVPD including the corresponding pseudopotential for iodine.[27–30] Calculation in various solvents were performed with the SMD-18[31] solvation model. For all structures, frequency calculations were performed to obtain thermodynamic data. All structures were confirmed as minima via the absence of imaginary frequencies. The output files obtained from Gaussian were analyzed using GoodVibes;[32] this includes the application of Grimme's entropy correction for low-frequencies.[33] Optimized energy values (Eopt) and Gibbs free energies (ΔG) are given in Hartree below.

Figure S2 depicts all calculated iodolium derivatives for the determination of the best self-assembled structure and Table S6 summarizes all geometric parameters calculated in the gas phase and in solution.

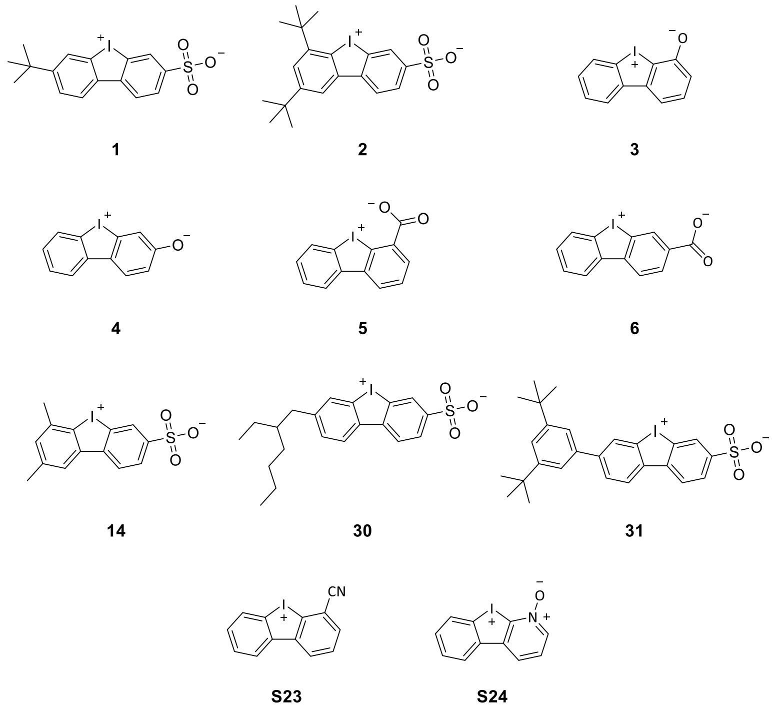

Figure S106: Calculated iodolium compounds using M06-2X/def2-TZVP(D). Substituents were introduced either in *ortho* or *meta* position (relative to the iodine) to determine the best geometric alignment.

Table S12: Geometric parameters of the calculated iodolium dimers **1-6**, **14**, **30**, **31**, **S23** and **S24** obtained from both gas phase calculations and in solution. ∡C-I···LB angles are given in °, dI···LB distances in Å, and Gibbs free binding energies (including Grimme's low-frequency entropy correction) in kcal·mol^{-1}.

Dimer	$∡_{C-I\cdots LB}$[a]	$d_{I\cdots LB}$[a]	ΔG[a]	ΔG_{MeCN}[b]	ΔG_{DCM}[b]
**1**	168	2.5	-50.6	-7.3	-11.5
**2**	168	2.5	-48.3	-8.2	-11.3
**3**	152	2.7	-8.3	24.7	1.6
**4**	150	2.9	-8.2	4.4	3.1
**5**	143	2.9	-4.5	7.0	5.1
**6**	164	2.4	-60.9	-13.1	-17.2
**14**	166	2.5	-48.9	-8.8	-10.7
**30**	166	2.5	-49.6	-8.2	-13.2
**31**	166	2.5	-51.5	-7.5	-11.3
**S23**	165	3.2	33.6	/	/
**S24**	158	2.9	34.8	/	/

[a] Results obtained from the gas-phase calculated with M06-2x / def2TZVP(D). [b] Binding energies obtained from solution calculated with SMD-18 using parameters either for acetonitrile (MeCN) or dichloromethane (DCM).

Gas-Phase Calculation:

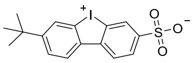

1

Eopt -1540.0888
ΔG -1539.8591

C	2.941905	-0.43942	0.000698
C	1.576532	-0.2815	-0.00123
C	0.898201	0.935587	-0.0031
C	1.716874	2.062733	-0.00315
C	3.09753	1.943824	-0.00134
C	3.742762	0.705782	0.000695
H	3.389929	-1.42382	0.002237
H	1.265016	3.045828	-0.0045
H	3.683216	2.851497	-0.00145
C	-0.5601	0.922631	-0.00434
C	-1.40039	2.041821	-0.00745
C	-1.21203	-0.30533	-0.00362
C	-2.77292	1.884199	-0.00885
H	-0.96534	3.033174	-0.00965
C	-2.56536	-0.52357	-0.00507
C	-3.35483	0.617854	-0.00645
H	-3.42793	2.746274	-0.01428
H	-3.04838	-1.4927	-0.00768
I	0.212666	-1.87207	-0.0017
S	-5.16167	0.436848	0.004414
O	-5.55252	1.183103	-1.16847
O	-5.33794	-0.99761	-0.07678
O	-5.51763	1.038771	1.268107
C	5.261961	0.551751	0.003161
C	5.971054	1.90581	0.00174
H	5.723674	2.489804	-0.8864
H	5.721576	2.492793	0.887309
H	7.049558	1.745607	0.003314
C	5.693272	-0.22566	-1.24853
H	5.259396	-1.22617	-1.27439
H	5.391734	0.299573	-2.15609
H	6.778996	-0.33463	-1.25966
C	5.689413	-0.22107	1.25902
H	5.385294	0.307598	2.163716
H	5.255151	-1.22136	1.287288
H	6.775076	-0.33024	1.273778

1 Dimer
Eopt -3080.2900
ΔG -3079.7988

C	-6.70025	-0.4569	-0.15292
C	-5.4096	0.02788	-0.17704
C	-5.09039	1.356908	0.096257
C	-6.14622	2.210051	0.399443
C	-7.44907	1.737502	0.423401
C	-7.75788	0.403906	0.149364
H	-6.89548	-1.5004	-0.3671
H	-5.95494	3.252295	0.619203
H	-8.23913	2.434098	0.663697
C	-3.67566	1.724895	0.022704

C	-3.1566	3.003375	0.231166
C	-2.76229	0.726737	-0.31201
C	-1.80065	3.241938	0.096833
H	-3.82212	3.815392	0.492348
C	-1.41468	0.928238	-0.49449
C	-0.9421	2.213135	-0.26978
H	-1.39069	4.231809	0.245782
H	-0.76137	0.135239	-0.82862
I	-3.70076	-1.12716	-0.54148
S	0.803581	2.538635	-0.47023
O	0.920968	3.95765	-0.66141
O	1.396816	2.084423	0.820432
O	1.241271	1.684912	-1.552
C	-9.18425	-0.14127	0.168481
C	-10.2069	0.938406	0.521904
H	-10.0256	1.355284	1.514171
H	-10.2006	1.752669	-0.20487
H	-11.206	0.501083	0.522527
C	-9.28371	-1.26329	1.211741
H	-8.61185	-2.09079	0.981495
H	-9.03379	-0.89105	2.206509
H	-10.3016	-1.65686	1.237257
C	-9.53016	-0.70129	-1.2186
H	-9.46021	0.077647	-1.97963
H	-8.86206	-1.51466	-1.50379
H	-10.5498	-1.09109	-1.21786
C	6.700214	0.456982	0.153244
C	5.409584	-0.02785	0.177196
C	5.090447	-1.35686	-0.0963
C	6.14633	-2.20992	-0.39951
C	7.449163	-1.73731	-0.4233
C	7.757901	-0.40374	-0.14908
H	6.89539	1.500461	0.367586
H	5.955128	-3.25215	-0.6194
H	8.239276	-2.43385	-0.66361
C	3.675717	-1.72487	-0.02292
C	3.156709	-3.00335	-0.23153
C	2.762275	-0.72677	0.311795
C	1.800773	-3.24199	-0.09729
H	3.822279	-3.81532	-0.49273
C	1.414648	-0.92837	0.494151
C	0.942133	-2.21329	0.269348
H	1.39089	-4.23188	-0.24633
H	0.76129	-0.13543	0.82831
I	3.700646	1.127069	0.5416
S	-0.80354	-2.5388	0.469867
O	-0.92082	-3.95784	0.660965
O	-1.2411	-1.68516	1.551766
O	-1.3969	-2.08449	-0.82068
C	9.184249	0.141516	-0.16805
C	10.20694	-0.93807	-0.52152
H	10.20061	-1.75243	0.20515
H	10.02581	-1.35482	-1.51386
H	11.20608	-0.5007	-0.522
C	9.530049	0.701425	1.219094
H	8.861903	1.514744	1.504316
H	9.460092	-0.07758	1.980055
H	10.54968	1.091266	1.218449
C	9.283697	1.263636	-1.2112
H	9.033855	0.891476	-2.20602
H	8.611761	2.09107	-0.98091
H	10.30155	1.657281	-1.23662

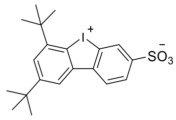

2

Eopt -1697.3205
ΔG -1696.9826

C	3.633562	0.690860	-0.005177
C	2.966245	1.914907	-0.005801
C	1.586640	1.979390	-0.003864
C	0.822378	0.805877	-0.001930
C	1.553141	-0.373853	-0.002563
C	2.920714	-0.498109	-0.004678
C	-0.637973	0.725256	-0.001069
C	-1.258908	-0.529346	-0.000528
I	0.242301	-2.011247	-0.002419
C	-1.472121	1.837166	-0.000646
C	-2.853193	1.705976	0.000254
C	-3.395673	0.421749	0.001135
C	-2.631156	-0.749317	0.000873
H	3.560877	2.819659	-0.010698
H	-1.020824	2.819801	-0.001077
H	3.464885	-1.434233	-0.008716
O	5.784583	1.409592	-1.168789
O	5.724014	-0.782897	-0.081458
H	1.092389	2.941986	-0.004987
S	5.446857	0.635527	0.003018
O	5.764217	1.258332	1.267284
H	-4.467296	0.321310	0.002021
C	-3.308664	-2.128887	0.002542
C	-3.724333	2.961327	0.000213
C	-3.409267	3.790553	1.253628
H	-2.365114	4.103019	1.281150

H	-4.027343	4.689850	1.266012
H	-3.616488	3.219338	2.160027
C	-3.410621	3.789526	-1.254203
H	-3.618873	3.217567	-2.159911
H	-4.028723	4.688800	-1.266658
H	-2.366491	4.101920	-1.283192
C	-5.217476	2.633941	0.001140
H	-5.508384	2.067792	-0.885840
H	-5.507485	2.068598	0.888928
H	-5.787785	3.563273	0.000983
C	-4.836437	-2.004040	0.003742
H	-5.201432	-1.485306	0.891028
H	-5.202975	-1.487154	-0.883990
H	-5.267622	-3.005065	0.005201
C	-2.932401	-2.914994	-1.262238
H	-3.207012	-2.355321	-2.157255
H	-1.872334	-3.154826	-1.335907
H	-3.471167	-3.863590	-1.272399
C	-2.930036	-2.912388	1.268265
H	-1.869593	-3.150907	1.340786
H	-3.203894	-2.351220	2.162570
H	-3.467981	-3.861423	1.280978

2 Dimer
Eopt -3394.7499
ΔG -3394.0432

C	0.747768	-2.434726	-1.405224
C	1.346678	-3.300475	-0.495194
C	2.504070	-2.931525	0.165251
C	3.072572	-1.670895	-0.042171
C	2.423798	-0.843018	-0.950893
C	1.296448	-1.189543	-1.663614
C	4.272593	-1.149753	0.621757
C	4.661823	0.161954	0.326467
I	3.303569	1.039939	-1.021923
C	5.035428	-1.888378	1.517574
C	6.160841	-1.341569	2.115691
C	6.503516	-0.029089	1.792711
C	5.781367	0.767027	0.899754
H	0.884895	-4.260589	-0.305277
H	4.743395	-2.904982	1.744307
H	0.861939	-0.517232	-2.385740
O	-0.333675	-3.672975	-3.401362
O	-1.393601	-1.634045	-2.588163
H	2.958830	-3.619267	0.865234
S	-0.751972	-2.930108	-2.250418
O	-1.524190	-3.648282	-1.254823
H	7.376049	0.395646	2.258882
C	6.234833	2.203648	0.592747
C	6.977669	-2.185215	3.094410
C	6.080863	-2.609234	4.266226
H	5.232536	-3.205907	3.929735
H	6.653842	-3.210954	4.974306
H	5.692688	-1.735644	4.792658
C	7.496463	-3.435764	2.370396
H	8.132445	-3.159357	1.527807
H	8.083502	-4.047348	3.058214
H	6.679702	-4.049749	1.989797
C	8.177590	-1.424859	3.659384
H	8.871194	-1.122628	2.872526
H	7.868548	-0.535664	4.212109
H	8.720709	-2.072085	4.349171
C	7.467670	2.594008	1.416117
H	7.266816	2.559533	2.488223
H	8.323942	1.954503	1.197335
H	7.747526	3.616666	1.163045
C	6.631332	2.329003	-0.886073
H	7.443258	1.639746	-1.123009
H	5.813283	2.125935	-1.575660
H	6.973439	3.345632	-1.086831
C	5.132730	3.216220	0.945782
H	4.253761	3.175633	0.302715
H	4.800072	3.078980	1.975839
H	5.530136	4.227583	0.847700
C	-0.747904	2.434864	-1.405258
C	-1.346688	3.300489	-0.495000
C	-2.504112	2.931554	0.165368
C	-3.072726	1.671003	-0.042284
C	-2.424092	0.843241	-0.951201
C	-1.296736	1.189826	-1.663935
C	-4.272673	1.149782	0.621730
C	-4.661824	-0.161953	0.326481
I	-3.303693	-1.039770	-1.022055
C	-5.035534	1.888315	1.517610
C	-6.160817	1.341369	2.115833
C	-6.503386	0.028842	1.792880
C	-5.781255	-0.767172	0.899832
H	-0.884708	4.260464	-0.304864
H	-4.743618	2.904964	1.744297
H	-0.862418	0.517618	-2.386290
O	0.333946	3.672823	-3.401416
O	1.393849	1.634210	-2.587602
H	-2.958807	3.619178	0.865508
S	0.751998	2.930263	-2.250187
O	1.523905	3.648645	-1.254543

H	-7.375808	-0.395998	2.259160
C	-6.234644	-2.203782	0.592656
C	-6.977620	2.184907	3.094671
C	-6.080691	2.608994	4.266370
H	-5.232467	3.205748	3.929758
H	-6.653625	3.210651	4.974540
H	-5.692358	1.735433	4.792733
C	-7.496647	3.435418	2.370765
H	-8.132705	3.158969	1.528247
H	-8.083671	4.046907	3.058681
H	-6.680011	4.049521	1.990088
C	-8.177385	1.424417	3.659796
H	-8.871032	1.122072	2.873019
H	-7.868177	0.535282	4.212523
H	-8.720512	2.071597	4.349620
C	-7.466855	-2.594624	1.416735
H	-7.265222	-2.560525	2.488712
H	-8.323369	-1.955156	1.198816
H	-7.746777	-3.617208	1.163446
C	-6.632041	-2.328599	-0.885965
H	-7.444757	-1.639938	-1.121923
H	-5.814592	-2.124262	-1.575899
H	-6.973346	-3.345419	-1.087139
C	-5.132140	-3.216369	0.944516
H	-4.254331	-3.176562	0.299805
H	-4.797623	-3.078597	1.973896
H	-5.529955	-4.227697	0.847765

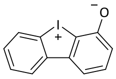

3

Eopt -834.2299
ΔG -834.1152

C	-0.64452	-3.40073	0
C	-1.94907	-2.94075	0
C	-2.30364	-1.58444	0
C	-1.27299	-0.64839	0
C	0	-1.17569	0
C	0.476899	-2.50036	0
C	-1.34324	0.81386	0
C	-0.13466	1.521319	0
I	1.521239	0.22547	0
C	-2.51407	1.57175	0
C	-2.45842	2.95486	0
C	-1.23668	3.620131	0
C	-0.05069	2.899297	0
H	-0.433	-4.46221	0
H	-2.746	-3.67484	0
H	-3.34021	-1.27925	0
H	-3.46893	1.062458	0
H	-3.37753	3.525452	0
H	-1.2036	4.701147	0
H	0.903828	3.40746	0
O	1.703274	-2.7519	0

3 Dimer
Eopt -1668.4971
ΔG -1668.2436

C	2.118182	3.498637	0.000033
C	3.473967	3.771595	0.000008
C	4.451207	2.775181	0.000013
C	4.033672	1.44701	0.000002
C	2.666439	1.225554	0.000049
C	1.616469	2.162471	0.000116
C	4.870418	0.24704	-5.7E-05
C	4.209929	-0.98518	-8.5E-05
I	2.124794	-0.78244	0.000066
C	6.265102	0.224321	-0.00012
C	6.946991	-0.98005	-0.00021
C	6.257666	-2.18883	-0.00022
C	4.870348	-2.19962	-0.00017
H	1.391443	4.300831	0.000013
H	3.790988	4.807503	-2.2E-05
H	5.501947	3.028007	-1.8E-05
H	6.810664	1.158877	-0.00012
H	8.028909	-0.98096	-0.00025
H	6.799303	-3.12509	-0.00028
H	4.326733	-3.13487	-0.00017
C	-2.11825	-3.4986	0.000042
C	-3.47407	-3.77155	0.000016
C	-4.45136	-2.7752	-2E-06
C	-4.03387	-1.44699	-1E-06
C	-2.66666	-1.22559	0.000034
C	-1.61664	-2.16243	0.000048
C	-4.87049	-0.24693	-5.2E-05
C	-4.20985	0.985236	-9.4E-05
I	-2.1247	0.782233	0.000115
C	-6.26516	-0.22407	-0.00013
C	-6.94693	0.980368	-0.00023
C	-6.25748	2.189066	-0.00026
C	-4.87016	2.199721	-0.00021
H	-1.39155	-4.30082	0.000069
H	-3.79106	-4.80747	0.000012
H	-5.50208	-3.02808	-2.5E-05
H	-6.81081	-1.15858	-0.00012
H	-8.02885	0.981384	-0.00029

H	-6.79902	3.125385	-0.00033
H	-4.32646	3.134924	-0.00021
O	-0.39866	-1.81437	0.000101
O	0.398429	1.814713	0.000029

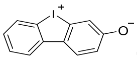
4

Eopt -834.2137
ΔG -834.1005

C	3.454131	0.567514	-0.00012
C	2.872495	1.901755	-7.6E-05
C	1.531174	2.128747	-1.5E-05
C	0.575813	1.080129	0.000002
C	1.170442	-0.18617	-5.3E-05
C	2.47387	-0.52711	-0.00011
C	-0.85238	1.205498	0.000062
C	-1.64005	0.04706	0.000067
I	-0.41389	-1.65052	-1.8E-05
C	-1.58612	2.405489	0.000117
C	-2.96696	2.400394	0.000173
C	-3.69383	1.210923	0.000175
C	-3.01646	-7.7E-05	0.000121
H	3.571829	2.727033	-9.4E-05
H	1.166323	3.149749	0.000021
H	-1.05032	3.345256	0.000117
H	-3.49519	3.344891	0.000215
H	-4.77443	1.222324	0.000218
H	-3.55326	-0.93851	0.000121
H	2.851242	-1.5383	-0.00016
O	4.660903	0.345005	-0.0002

4 Dimer
Eopt -1668.4676
ΔG -1668.2140

C	-0.42387	2.80907	0.975037
C	-1.31042	3.561469	0.121704
C	-2.41074	3.002458	-0.46556
C	-2.75584	1.642922	-0.29103
C	-1.90829	0.943782	0.564977
C	-0.83287	1.449469	1.232069
C	-3.8762	0.944523	-0.88002
C	-4.02547	-0.41487	-0.58538
I	-2.45999	-1.11148	0.615938
C	-4.85295	1.497266	-1.71777
C	-5.88886	0.721764	-2.20608
C	-5.99486	-0.62835	-1.88355
C	-5.04473	-1.21452	-1.05894
H	-1.05972	4.599538	-0.05238
H	-3.03596	3.614808	-1.10523
H	-4.79333	2.545082	-1.98095
H	-6.63056	1.175537	-2.8502
H	-6.81022	-1.22367	-2.27024
H	-5.10701	-2.26281	-0.79986
C	0.423938	-2.80919	0.974872
C	1.310605	-3.56155	0.121652
C	2.410972	-3.00252	-0.46551
C	2.756	-1.64297	-0.29101
C	1.908334	-0.94384	0.564902
C	0.832882	-1.44956	1.231915
C	3.8763	-0.94448	-0.87997
C	4.025417	0.414929	-0.58539
I	2.459883	1.111459	0.615929
C	4.853115	-1.49717	-1.71768
C	5.888961	-0.7216	-2.206
C	5.994834	0.628534	-1.88352
C	5.044631	1.214638	-1.05894
H	1.059958	-4.59962	-0.05248
H	3.036245	-3.61488	-1.10512
H	4.793578	-2.545	-1.98081
H	6.630715	-1.17533	-2.85009
H	6.810141	1.223925	-2.27021
H	5.106822	2.262949	-0.7999
H	-0.28126	0.904535	1.980548
H	0.280992	-0.90447	1.98008
O	-0.6436	-3.25717	1.450039
O	0.643558	3.257042	1.450357

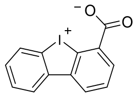
5

Eopt -947.6081
ΔG -947.4830

C	0.172609	3.253354	0
C	1.557235	3.100101	0
C	2.155164	1.843681	0
C	1.363591	0.694219	0
C	0	0.917752	0
C	-0.63817	2.125776	0
C	1.784403	-0.71431	0
C	0.760844	-1.66779	0
I	-1.17174	-0.77118	0
C	3.106061	-1.15611	0
C	3.384633	-2.51257	0
C	2.353618	-3.44608	0
C	1.030674	-3.02487	0
H	-0.29607	4.228877	0

H	2.187946	3.979343	0
H	3.234516	1.762337	0
H	3.916066	-0.43792	0
H	4.413128	-2.84801	0
H	2.580307	-4.504	0
H	0.229869	-3.75395	0
C	-2.16201	2.135358	0
O	-2.74842	3.194263	0
O	-2.67349	0.950082	0

5 Dimer

Eopt -1895.2467

ΔG -1894.9731

C	4.746347	-3.53935	0.000816
C	5.391605	-2.3088	0.000895
C	4.647915	-1.12788	0.000507
C	3.269954	-1.27751	0.000046
C	5.145114	0.253737	0.000551
C	4.172172	1.258001	0.00014
I	2.200081	0.486301	-0.00051
C	6.487086	0.63044	0.000982
C	6.830513	1.971874	0.001
C	5.846287	2.955352	0.000611
C	4.504092	2.601619	0.000182
H	5.339375	-4.44443	0.001122
H	6.47333	-2.26899	0.001263
H	7.261865	-0.12565	0.001303
H	7.874228	2.256765	0.001327
H	6.124891	4.000799	0.000646
H	3.735415	3.364486	-8.8E-05
C	3.356961	-3.63038	0.000359
C	2.592352	-2.47206	-4.3E-05
H	2.846181	-4.58449	0.000308
C	-2.59202	2.471962	-3.8E-05
C	-3.2698	1.277501	0.000065
C	-3.35645	3.630398	0.000373
C	-4.64778	1.128089	0.000546
I	-2.20021	-0.48649	-0.00047
C	-4.74585	3.539577	0.000866
H	-2.84552	4.584425	0.000292
C	-5.39129	2.309116	0.000954
C	-5.1452	-0.25345	0.000568
C	-4.17242	-1.25788	0.000124
H	-5.33875	4.444738	0.001189
H	-6.47302	2.269483	0.00134
C	-6.48724	-0.62991	0.000983
C	-4.5046	-2.60143	0.000094
C	-6.83092	-1.97127	0.000952

H	-7.26188	0.126328	0.001331
C	-5.84685	-2.95492	0.000511
H	-3.73611	-3.36448	-0.00025
H	-7.87468	-2.25599	0.001275
H	-6.12564	-4.00032	0.000494
C	1.07315	-2.45002	-0.00054
C	-1.07282	2.449658	-0.00062
O	0.589413	-1.26189	-0.00094
O	0.447928	-3.49468	-0.00052
O	-0.44738	3.494188	-0.00073
O	-0.58933	1.261439	-0.00095

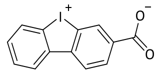

6

Eopt -947.5488

ΔG -947.4270

C	-2.859506	0.515278	-0.000091
C	-2.390473	1.825243	-0.000285
C	-1.035245	2.110855	-0.000430
C	-0.097784	1.074251	-0.000187
C	-0.641955	-0.208782	-0.000115
C	-1.963082	-0.550073	-0.000081
C	1.353647	1.219484	-0.000041
C	2.137233	0.066892	0.000113
I	0.936766	-1.644255	-0.000106
C	2.053528	2.429056	-0.000026
C	3.436776	2.445541	0.000205
C	4.170324	1.263114	0.000390
C	3.516079	0.040049	0.000310
H	-3.138101	2.608615	-0.000126
H	1.501380	3.359003	-0.000146
H	3.955257	3.394792	0.000258
H	5.251241	1.288515	0.000541
H	4.070303	-0.887874	0.000368
H	-2.377645	-1.551598	-0.000213
C	-4.394210	0.185023	0.000163
O	-5.107361	1.190330	0.000105
O	-4.634059	-1.027753	0.000639
H	-0.691643	3.137877	-0.000545

6 Dimer

Eopt -1895.2265

ΔG -1894.9509

C	-0.860334	-2.309904	-0.351925
C	-1.676896	-3.195262	0.347985
C	-2.975044	-2.848048	0.685054
C	-3.476802	-1.576725	0.386617
C	-2.617070	-0.717240	-0.289358

C	-1.358063	-1.065937	-0.711706
C	-4.809081	-1.066132	0.722377
C	-5.068196	0.258509	0.368808
I	-3.398715	1.215245	-0.509587
C	-5.819810	-1.794402	1.349523
C	-7.043391	-1.201251	1.608248
C	-7.279398	0.122526	1.250386
C	-6.285381	0.864082	0.628020
H	-1.266239	-4.156180	0.629210
H	-5.650157	-2.825209	1.631784
H	-7.823072	-1.773323	2.093250
H	-8.239199	0.577223	1.456629
H	-6.467144	1.895366	0.352508
C	0.860324	2.309892	-0.351721
C	1.676961	3.195350	0.347971
C	2.975112	2.848134	0.685043
C	3.476825	1.576755	0.386764
C	2.617027	0.717190	-0.289026
C	1.357973	1.065825	-0.711262
C	4.809100	1.066166	0.722539
C	5.068165	-0.258527	0.369127
I	3.398699	-1.215279	-0.509278
C	5.819865	1.794476	1.349582
C	7.043428	1.201312	1.608363
C	7.279385	-0.122515	1.250653
C	6.285336	-0.864109	0.628384
H	1.266375	4.156346	0.629034
H	5.650250	2.825322	1.631724
H	7.823136	1.773415	2.093285
H	8.239172	-0.577222	1.456939
H	6.467063	-1.895428	0.352984
H	-0.777812	-0.377479	-1.291285
H	0.777610	0.377224	-1.290544
C	-0.605947	2.627506	-0.613307
C	0.605965	-2.627487	-0.613400
O	-1.094048	3.628605	-0.115691
O	1.094030	-3.628604	-0.115781
O	1.229740	-1.738948	-1.306399
O	-1.229667	1.739011	-1.306418
H	3.600112	3.556560	1.213015
H	-3.599993	-3.556411	1.213171

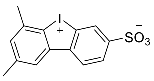

14

Eopt -1461.4800
ΔG -1461.3070

C	2.719710	0.544674	-0.007339
C	2.183612	1.831260	-0.009154
C	0.817771	2.037650	-0.006805
C	-0.062244	0.949358	-0.003442
C	0.544017	-0.302158	-0.003514
C	1.889398	-0.567243	-0.005860
C	1.520184	1.016508	-0.001450
C	-2.234386	-0.179980	0.000174
I	-0.930904	-1.818242	-0.001444
C	-2.284701	2.180610	-0.000589
C	-3.673585	2.130549	0.001789
C	-4.314442	0.891941	0.003403
C	-3.608853	-0.308140	0.002664
H	2.869084	2.669301	-0.015175
H	-1.786000	3.141872	-0.001656
H	2.336980	-1.553212	-0.009364
O	4.937065	1.039518	-1.163988
O	4.644643	-1.139490	-0.086960
H	0.419076	3.044054	-0.008610
S	4.518716	0.299688	0.003484
O	4.894291	0.880320	1.271509
H	-5.397227	0.853332	0.005348
C	-4.290260	-1.643359	0.004547
H	-4.014539	-2.222388	-0.880476
H	-4.011732	-2.221271	0.889423
H	-5.371683	-1.529285	0.006188
C	-4.468055	3.406173	0.002618
H	-4.229004	4.006815	0.881070
H	-4.233436	4.005172	-0.878156
H	-5.537834	3.207606	0.005483

14 Dimer

Eopt -2923.0690
ΔG -2922.6920

C	0.775056	2.437993	-0.847387
C	1.398834	3.321396	0.028246
C	2.573999	2.964061	0.663214
C	3.134123	1.699254	0.464628
C	2.457810	0.849419	-0.407638
C	1.312972	1.185405	-1.095414
C	4.353872	1.192132	1.096162
C	4.710942	-0.117238	0.793908
I	3.341208	-1.043723	-0.487102
C	5.178172	1.911097	1.962967
C	6.314039	1.329245	2.501978
C	6.625435	0.007084	2.169508
C	5.835477	-0.746366	1.313434
H	0.943953	4.286450	0.209655
H	4.933143	2.934827	2.216257

C	-0.775100	-2.437910	-0.847810
C	-1.398351	-3.321074	0.028426
C	-2.573422	-2.963777	0.663599
C	-3.133855	-1.699162	0.464732
C	-2.457971	-0.849482	-0.408034
C	-1.313392	-1.185541	-1.096207
C	-4.353556	-1.192098	1.096408
C	-4.710975	0.117103	0.793839
I	-3.341534	1.043578	-0.487483
C	-5.177522	-1.910983	1.963596
C	-6.313399	-1.329213	2.502674
C	-6.625142	-0.007213	2.169887
C	-5.835516	0.746158	1.313439
H	-0.943138	-4.285919	0.210108
H	-4.932230	-2.934590	2.217128
H	0.859432	0.501959	-1.794467
H	-0.860406	-0.502362	-1.795910
O	1.493850	-3.650599	-0.663108
O	-1.494074	3.650312	-0.662220
O	-1.389815	1.632477	-1.991093
O	1.389814	-1.632250	-1.991214
H	-3.053872	-3.661843	1.336234
H	3.054799	3.662270	1.335451
S	-0.740250	2.929166	-1.667525
S	0.740119	-2.928999	-1.668158
O	-0.339483	3.665507	-2.828826
O	0.339281	-3.664829	-2.829756
H	-7.514223	0.446644	2.594291
H	7.514509	-0.446838	2.593859
C	6.168461	-2.166185	0.958328
H	5.354776	-2.840701	1.237198
H	6.332136	-2.270425	-0.117200
H	7.069896	-2.498298	1.468789
C	7.212488	2.095888	3.431767
H	8.215752	2.184961	3.012299
H	6.830360	3.098527	3.614577
H	7.303098	1.584459	4.391126
C	-6.168843	2.165819	0.958024
H	-5.355233	2.840564	1.236561
H	-6.332746	2.269743	-0.117500
H	-7.070240	2.497894	1.468575
C	-7.211484	-2.095770	3.432885
H	-8.214878	-2.185002	3.013761
H	-6.829211	-3.098344	3.615750
H	-7.301825	-1.584181	4.392185

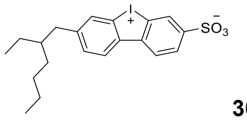

30

Eopt -1697.3156
ΔG -1696.9769

C	-2.102240	2.195838	0.113318
C	-2.704460	1.227154	-0.695447
C	-1.910805	0.199486	-1.194867
C	-0.573433	0.196317	-0.858251
C	0.062764	1.137276	-0.055141
C	-0.758336	2.159510	0.429944
H	-2.712788	3.002979	0.500506
H	-2.344222	-0.568445	-1.822178
H	-0.329962	2.930036	1.056970
C	2.153881	-0.098599	-0.363618
C	3.482489	-0.411884	-0.238344
C	4.230076	0.455780	0.545634
C	3.634773	1.564740	1.143623
C	2.288633	1.830132	0.980750
C	1.489937	0.983786	0.203554
H	3.976791	-1.258613	-0.697922
H	4.259502	2.219453	1.738001
H	1.843248	2.696809	1.452416
I	0.792674	-1.253414	-1.501706
S	5.994668	0.110445	0.798450
O	6.032107	-0.240209	2.199288
O	6.610500	1.371667	0.459400
O	6.240942	-0.978043	-0.123436
C	-4.178678	1.283408	-0.982544
H	-4.419422	0.619994	-1.818670
H	-4.427688	2.298407	-1.296924
C	-5.050834	0.894782	0.225457
H	-4.836175	1.606004	1.034200
C	-6.538024	1.012751	-0.119800
H	-6.790303	0.239543	-0.854347
H	-7.116310	0.783119	0.776550
C	-6.966155	2.381153	-0.638996
H	-6.578096	2.584303	-1.637267
H	-8.052293	2.449324	-0.694802
H	-6.618273	3.175954	0.025230
C	-4.697663	-0.508799	0.723261
H	-4.824519	-1.213995	-0.106946
H	-3.636571	-0.537553	0.993089
C	-5.510992	-0.976093	1.928604
H	-5.576188	-0.157236	2.652319
H	-6.535882	-1.203222	1.622659
C	-4.917493	-2.205610	2.613031

H	-3.920379	-1.960742	2.990750
H	-5.525374	-2.452342	3.486104
C	-4.832251	-3.422199	1.698077
H	-5.809682	-3.653880	1.268747
H	-4.138168	-3.254985	0.872867
H	-4.489189	-4.301961	2.242348

30 Dimer
Eopt -3394.7412
ΔG -3394.0326

C	6.03144	1.294426	2.328752
C	6.589764	0.986506	1.084405
C	5.891241	0.131443	0.239266
C	4.676468	-0.384193	0.654203
C	4.105527	-0.082239	1.887979
C	4.814989	0.776241	2.729447
H	6.573591	1.953591	2.996726
H	6.303898	-0.118947	-0.73097
H	4.416612	1.031438	3.702722
C	2.285246	-1.576432	1.237792
C	1.09922	-2.260723	1.381847
C	0.3643	-2.011262	2.52977
C	0.837593	-1.137652	3.503518
C	2.057918	-0.507075	3.343841
C	2.816911	-0.707409	2.187754
H	0.762627	-2.959437	0.63384
H	0.23152	-0.954524	4.381178
H	2.42054	0.163009	4.112096
I	3.466368	-1.655871	-0.486692
S	-1.211354	-2.828947	2.767213
O	-2.069883	-1.827708	3.364732
O	-0.936977	-3.999077	3.545648
O	-1.646698	-3.14123	1.379329
C	7.899906	1.596715	0.669946
H	8.299768	1.059852	-0.195699
H	8.61389	1.466476	1.485213
C	7.79651	3.091896	0.318738
H	7.453101	3.622682	1.216903
C	9.167259	3.657087	-0.064147
H	9.478491	3.207973	-1.014155
H	9.057705	4.726447	-0.252656
C	10.255746	3.455836	0.984858
H	10.553874	2.411118	1.073872
H	11.14837	4.026863	0.729691
H	9.916004	3.792598	1.967346
C	6.765046	3.320654	-0.78854
H	7.053313	2.72281	-1.66122
H	5.795256	2.93384	-0.458891
C	6.587804	4.781932	-1.195934
H	6.545436	5.400066	-0.293037
H	7.458888	5.120798	-1.763727
C	5.332016	5.024561	-2.030438
H	4.451202	4.757622	-1.438903
H	5.249033	6.092813	-2.243535
C	5.320033	4.245691	-3.341133
H	6.218563	4.456944	-3.925536
H	5.281383	3.169525	-3.167575
H	4.455405	4.510253	-3.949868
C	-6.031447	1.294384	-2.328787
C	-6.589768	0.986494	-1.084433
C	-5.891246	0.131443	-0.239278
C	-4.676477	-0.384208	-0.654207
C	-4.105543	-0.082285	-1.887992
C	-4.815002	0.77618	-2.729477
H	-6.573593	1.953539	-2.996776
H	-6.303904	-0.118928	0.730964
H	-4.416629	1.031351	-3.702761
C	-2.285264	-1.576478	-1.237786
C	-1.099238	-2.26077	-1.381841
C	-0.364322	-2.011322	-2.529773
C	-0.837629	-1.137736	-3.503534
C	-2.057955	-0.507163	-3.343859
C	-2.816934	-0.707472	-2.18776
H	-0.762644	-2.959478	-0.633832
H	-0.231564	-0.954618	-4.381202
H	-2.420586	0.162906	-4.112124
I	-3.466364	-1.655846	0.486726
S	1.21136	-2.82897	-2.7672
O	2.069876	-1.827663	-3.364644
O	0.937063	-3.999064	-3.545716
O	1.646665	-3.14129	-1.37932
C	-7.899898	1.596725	-0.669976
H	-8.299764	1.05988	0.195679
H	-8.613889	1.466488	-1.485237
C	-7.796475	3.091909	-0.31878
H	-7.453035	3.622683	-1.21694
C	-9.16722	3.657133	0.064076
H	-9.478484	3.208023	1.014074
H	-9.057643	4.72649	0.252592
C	-10.255688	3.455912	-0.984954
H	-10.553833	2.4112	-1.073982
H	-11.148307	4.026955	-0.729801
H	-9.915918	3.792674	-1.967433
C	-6.765037	3.320649	0.788523
H	-7.05331	2.722761	1.661172

H	-5.79523	2.933868	0.458884
C	-6.587848	4.781914	1.195989
H	-6.54551	5.400097	0.293125
H	-7.458947	5.120714	1.7638
C	-5.332062	5.024549	2.030497
H	-4.45124	4.757709	1.438927
H	-5.24914	6.09279	2.243677
C	-5.32001	4.245582	3.341132
H	-6.218529	4.456752	3.925581
H	-5.281322	3.16943	3.16749
H	-4.455373	4.510131	3.949858

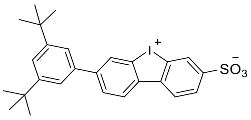

31

Eopt -1928.3648
ΔG -1927.9495

C	5.550276	0.648503	-0.284719
C	4.923444	1.765086	-0.835035
C	3.546362	1.859057	-0.890149
C	2.746204	0.825083	-0.390068
C	3.4417	-0.254838	0.142798
C	4.802014	-0.403374	0.224975
C	1.288888	0.786963	-0.380002
C	0.655456	-0.331764	0.155109
I	2.072932	-1.709227	0.844733
C	0.433524	1.780631	-0.862718
C	-0.937825	1.629371	-0.796842
C	-1.534754	0.487214	-0.250513
C	-0.705235	-0.521496	0.240632
H	5.547086	2.562072	-1.219895
H	0.855534	2.674938	-1.301381
H	-1.573843	2.406469	-1.200372
H	-1.13366	-1.405354	0.693382
H	5.319405	-1.256001	0.646713
O	7.715657	0.609787	-1.625225
O	7.589785	-0.726147	0.421542
H	3.076663	2.733937	-1.321308
S	7.362395	0.548227	-0.226204
O	7.707686	1.715301	0.551149
C	-3.005726	0.343296	-0.187571
C	-3.605689	-0.884734	-0.448539
C	-3.802773	1.437303	0.13469
C	-4.987224	-1.037968	-0.393068
H	-2.981632	-1.723823	-0.733082
C	-5.187511	1.323157	0.203831
H	-3.326741	2.383416	0.36259
C	-5.755589	0.07802	-0.063728
H	-6.828139	-0.025159	-0.015536
C	-6.024029	2.547845	0.575168
C	-5.605238	-2.401089	-0.70579
C	-5.252015	-2.797566	-2.146264
H	-4.173518	-2.868781	-2.291336
H	-5.688183	-3.769849	-2.383888
H	-5.640436	-2.064259	-2.854896
C	-5.041507	-3.44991	0.263297
H	-5.481057	-4.426953	0.053513
H	-3.959035	-3.544385	0.168745
H	-5.270846	-3.184696	1.296823
C	-7.127787	-2.393672	-0.569097
H	-7.440071	-2.135316	0.444428
H	-7.591489	-1.692368	-1.264993
H	-7.514977	-3.388448	-0.793545
C	-7.522688	2.246619	0.584127
H	-7.873581	1.918336	-0.395973
H	-7.775631	1.480044	1.318776
H	-8.070708	3.15223	0.847519
C	-5.620741	3.030554	1.975705
H	-4.568094	3.312215	2.017323
H	-6.213218	3.904286	2.254071
H	-5.791743	2.248931	2.717867
C	-5.764554	3.66712	-0.443148
H	-4.715902	3.966225	-0.454665
H	-6.036743	3.345106	-1.449751
H	-6.360087	4.54661	-0.190907

31 Dimer
Eopt -3856.8398
ΔG -3855.9798

C	-0.342044	-2.206272	-2.517554
C	-0.78826	-1.30273	-3.476267
C	-1.991531	-0.642027	-3.307815
C	-2.758343	-0.842276	-2.157132
C	-2.253543	-1.742834	-1.221343
C	-1.086742	-2.456928	-1.375587
C	-4.028876	-0.186095	-1.848952
C	-4.614037	-0.497717	-0.622919
I	-3.439098	-1.817872	0.500678
C	-4.705826	0.715024	-2.670985
C	-5.905829	1.265526	-2.264009
C	-6.479806	0.944832	-1.028513
C	-5.810522	0.045985	-0.198595
H	-0.173961	-1.119921	-4.348204
H	-4.297822	0.977295	-3.638293
H	-6.430724	1.943196	-2.924955

H	-6.222777	-0.195004	0.773368
H	-0.769944	-3.17729	-0.639105
O	0.906604	-4.240074	-3.512021
O	1.659589	-3.354958	-1.372253
H	-2.3335	0.051754	-4.064389
S	1.213417	-3.060427	-2.7611
O	2.083379	-2.086985	-3.386914
C	-7.766022	1.543737	-0.606908
C	-8.715363	0.777666	0.062651
C	-8.039084	2.882105	-0.873007
C	-9.928603	1.323843	0.468853
H	-8.509286	-0.271082	0.240577
C	-9.238926	3.465858	-0.479835
H	-7.284621	3.47973	-1.370428
C	-10.16765	2.668501	0.187947
H	-11.10323	3.106892	0.497677
C	-9.48762	4.944728	-0.778861
C	-10.946841	0.438941	1.188777
C	-11.352388	-0.719653	0.266679
H	-10.49514	-1.336211	-0.005204
H	-12.080985	-1.359477	0.768753
H	-11.802211	-0.34172	-0.653034
C	-10.314342	-0.127128	2.467959
H	-11.034357	-0.75715	2.994029
H	-9.436633	-0.735612	2.247887
H	-10.008799	0.678697	3.137521
C	-12.21032	1.205922	1.579009
H	-11.98655	2.032949	2.255415
H	-12.726901	1.603355	0.703472
H	-12.897288	0.531876	2.092704
C	-10.8616	5.412063	-0.298638
H	-11.667285	4.857743	-0.783512
H	-10.968371	5.305101	0.7823
H	-10.989431	6.467746	-0.542461
C	-8.417697	5.790492	-0.073923
H	-7.414268	5.535449	-0.41612
H	-8.584204	6.850252	-0.278068
H	-8.455257	5.638097	1.006053
C	-9.403248	5.178758	-2.293758
H	-8.42135	4.913992	-2.687415
H	-10.151058	4.582169	-2.819081
H	-9.582245	6.231879	-2.52004
C	0.342024	-2.206424	2.517351
C	0.788076	-1.302739	3.475998
C	1.991298	-0.641928	3.307556
C	2.758199	-0.842214	2.15695
C	2.253532	-1.74289	1.221188
C	1.086806	-2.457098	1.375431
C	4.028723	-0.185993	1.848828
C	4.614025	-0.497718	0.622895
I	3.439208	-1.817924	-0.500766
C	4.705552	0.715244	2.670834
C	5.90558	1.265745	2.263933
C	6.479702	0.944935	1.028535
C	5.810536	0.045982	0.198638
H	0.173705	-1.119925	4.347881
H	4.297428	0.977608	3.638067
H	6.430377	1.943506	2.924864
H	6.222906	-0.195091	-0.773256
H	0.770109	-3.17754	0.638982
O	-0.90621	-4.240538	3.511624
O	-1.659598	-3.355208	1.372091
H	2.333149	0.051954	4.06409
S	-1.2133	-3.060818	2.760932
O	-2.083313	-2.087607	3.387023
C	7.765951	1.543817	0.607
C	8.715376	0.777687	-0.062367
C	8.038956	2.882222	0.872971
C	9.92865	1.323838	-0.468504
H	8.509336	-0.271086	-0.240191
C	9.238824	3.465956	0.479849
H	7.284435	3.479888	1.370256
C	10.167637	2.668535	-0.187737
H	11.103241	3.106908	-0.497423
C	9.487456	4.944864	0.778737
C	10.946975	0.438865	-1.188217
C	11.352446	-0.719612	-0.265941
H	10.495179	-1.336136	0.005956
H	12.081083	-1.3595	-0.767876
H	11.802199	-0.341562	0.653759
C	10.314606	-0.127367	-2.467391
H	11.034682	-0.757436	-2.993324
H	9.436892	-0.735844	-2.247323
H	10.009104	0.678372	-3.137074
C	12.210485	1.205811	-1.578418
H	11.986777	2.03275	-2.254951
H	12.726975	1.603359	-0.702879
H	12.897511	0.531708	-2.09196
C	10.8615	5.412166	0.298662
H	11.667124	4.857935	0.78374
H	10.96844	5.305053	-0.782244
H	10.989268	6.467886	0.542357
C	8.417628	5.790539	0.07355
H	7.414151	5.535535	0.415633

H	8.584102	6.850323	0.277597
H	8.455342	5.638022	-1.006404
C	9.402856	5.179059	2.293595
H	8.420907	4.914303	2.687134
H	10.150608	4.582552	2.819094
H	9.581785	6.232211	2.519788

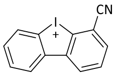

S23

Eopt -851.6354
ΔG -851.5143

C	-0.346558	2.430839	0
C	0.000000	1.087596	0
C	0.691445	3.356858	0
C	1.297898	0.600723	0
I	-1.404373	-0.449354	0
C	2.008847	2.911654	0
H	0.458635	4.412821	0
C	2.313947	1.561111	0
C	1.470504	-0.852329	0
C	0.322524	-1.643434	0
H	2.81048	3.637184	0
H	3.348693	1.245443	0
C	2.688351	-1.531894	0
C	0.305164	-3.020557	0
C	2.716697	-2.916095	0
H	3.614927	-0.973347	0
C	1.539864	-3.657203	0
H	-0.613428	-3.590372	0
H	3.669375	-3.427705	0
H	1.575983	-4.738065	0
C	-1.732796	2.784397	0
N	-2.869745	2.939973	0

S23 Dimer
Eopt -1703.2414
ΔG -1702.9752

C	4.252141	3.831125	0.09802
C	5.209885	2.834033	0.075723
C	4.841955	1.484801	0.037351
C	3.480008	1.211684	0.023918
C	5.762894	0.34862	0.008704
C	5.209885	-0.929348	-0.03027
I	3.11388	-0.842273	-0.032154
C	7.155693	0.422735	0.015766
C	7.915873	-0.734025	-0.015061
C	7.312914	-1.986824	-0.053459
C	5.928656	-2.104128	-0.061529
H	4.559274	4.867406	0.12763
H	6.258592	3.099474	0.087751
H	7.645781	1.386731	0.045424
H	8.994786	-0.661008	-0.00919
H	7.91883	-2.882405	-0.07739
H	5.455305	-3.075566	-0.091223
C	2.897735	3.521949	0.082002
C	2.492586	2.190301	0.04404
H	2.149523	4.302415	0.098277
C	-2.492586	-2.190301	0.04404
C	-3.480008	-1.211684	0.023918
C	-2.897735	-3.521949	0.082002
C	-4.841955	-1.484801	0.037351
I	-3.11388	0.842273	-0.032154
C	-4.252141	-3.831125	0.09802
H	-2.149523	-4.302415	0.098277
C	-5.209885	-2.834033	0.075723
C	-5.762894	-0.34862	0.008704
C	-5.209885	0.929348	-0.03027
H	-4.559274	-4.867406	0.12763
H	-6.258592	-3.099474	0.087751
C	-7.155693	-0.422735	0.015766
C	-5.928656	2.104128	-0.061529
C	-7.915873	0.734025	-0.015061
H	-7.645781	-1.386731	0.045424
C	-7.312914	1.986824	-0.053459
H	-5.455305	3.075566	-0.091223
H	-8.994786	0.661008	-0.00919
H	-7.91883	2.882405	-0.07739
C	1.103572	1.845356	0.023446
N	-0.002986	1.545612	0.004429
C	-1.103572	-1.845356	0.023446
N	0.002986	-1.545612	0.004429

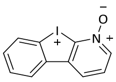

S24

Eopt -850.5968
ΔG -850.4805

C	0	1.132306	0
C	0.610665	3.337152	0
C	1.279918	0.630931	0
I	-1.522605	-0.224334	0
C	1.932933	2.927076	0
H	0.272002	4.362191	0
C	2.295146	1.586424	0
C	1.348934	-0.831491	0
C	0.138078	-1.533621	0
H	2.694306	3.694434	0

H	3.336422	1.296816	0
C	2.517444	-1.588643	0
C	0.035799	-2.906688	0
C	2.450575	-2.972179	0
H	3.477826	-1.089613	0
C	1.224649	-3.627287	0
H	-0.917871	-3.415955	0
H	3.36486	-3.549508	0
H	1.185365	-4.708076	0
O	-1.627044	2.684169	0
N	-0.386188	2.414598	0

S24 Dimer
Eopt -1701.1610
ΔG -1700.9056

C	3.550519	3.634985	-0.000218
C	2.547590	4.591421	-0.000163
C	1.219597	4.165552	-0.000089
C	1.024814	2.799572	-0.000082
C	-0.008254	4.958706	0.000002
C	-1.219597	4.260245	0.000089
I	-0.924246	2.170119	0.00004
C	-0.078071	6.349878	0.000023
C	-1.310064	6.982957	0.000125
C	-2.490076	6.247492	0.00021
C	-2.460428	4.857389	0.000196
H	4.590189	3.930944	-0.000267
H	2.79374	5.644261	-0.000175
H	0.832117	6.935505	-0.000039
H	-1.353685	8.063425	0.000139
H	-3.445719	6.754236	0.000289
H	-3.378944	4.287092	0.000263
C	3.261569	2.281393	-0.000197
H	3.996022	1.490747	-0.000217
C	-1.024814	-2.799572	-0.000082
C	-3.261569	-2.281393	-0.000197
C	-1.219597	-4.165552	-0.000089
I	0.924246	-2.170119	0.00004
C	-3.550519	-3.634985	-0.000218
H	-3.996022	-1.490747	-0.000217
C	-2.54759	-4.591421	-0.000163
C	0.008254	-4.958706	0.000002
C	1.219597	-4.260245	0.000089
H	-4.590189	-3.930944	-0.000267
H	-2.79374	-5.644261	-0.000175
C	0.078071	-6.349878	0.000023
C	2.460428	-4.857389	0.000196
C	1.310064	-6.982957	0.000125
H	-0.832117	-6.935505	-0.000039
C	2.490076	-6.247492	0.00021
H	3.378944	-4.287092	0.000263
H	1.353685	-8.063425	0.000139
H	3.445719	-6.754236	0.000289
O	-1.63815	-0.616206	-0.000044
O	1.63815	0.616206	-0.000044
N	1.977103	1.859752	-0.000163
N	-1.977103	-1.859752	-0.000163

Calculation in Acetonitrile:

1 Monomer
Eopt -1540.1559
ΔG -1539.9260

C	2.938635	-0.44587	0.002437
C	1.577427	-0.27317	0.000004
C	0.905691	0.946627	-0.00216
C	1.723887	2.073118	-0.00107
C	3.104673	1.944392	-0.00016
C	3.740752	0.700931	0.001173
H	3.37963	-1.43376	0.004914
H	1.278257	3.059633	-0.00076
H	3.695273	2.849205	-0.00016
C	-0.55541	0.932185	-0.00497
C	-1.38797	2.049708	-0.01052
C	-1.19707	-0.3064	-0.00491
C	-2.76602	1.89707	-0.0129
H	-0.95458	3.041255	-0.01471
C	-2.55523	-0.50856	-0.01012
C	-3.34662	0.635285	-0.01209
H	-3.40749	2.767908	-0.01957
H	-2.99923	-1.49551	-0.01622
I	0.207149	-1.8603	-0.00186
S	-5.13119	0.432515	0.005352
O	-5.66215	1.771479	-0.17973
O	-5.40339	-0.47293	-1.0988
O	-5.41479	-0.14119	1.311087
C	5.258548	0.539806	0.003071
C	5.974268	1.889237	-0.01679
H	5.724126	2.464748	-0.91033
H	5.729804	2.488414	0.86254
H	7.052027	1.718574	-0.01779
C	5.682622	-0.25866	-1.23713
H	5.244901	-1.25824	-1.24271
H	5.381317	0.255173	-2.15244
H	6.769147	-0.36768	-1.2462
C	5.681644	-0.22218	1.266189

H	5.38414	0.320637	2.165889
H	5.238719	-1.21885	1.302702
H	6.767704	-0.33587	1.276919

1 Dimer
Eopt -3080.3533
ΔG -3079.9637

C	6.786877	0.487223	-0.142891
C	5.50878	-0.02316	-0.163356
C	5.189189	-1.35348	0.103375
C	6.253101	-2.198616	0.403383
C	7.55106	-1.71103	0.427676
C	7.848289	-0.37293	0.157489
H	6.97048	1.533083	-0.355361
H	6.069979	-3.243703	0.618228
H	8.347332	-2.402263	0.664452
C	3.778854	-1.732731	0.027681
C	3.266836	-3.014202	0.237556
C	2.856742	-0.741955	-0.306895
C	1.911349	-3.258785	0.100686
H	3.936302	-3.822412	0.501234
C	1.508698	-0.946492	-0.482442
C	1.042972	-2.235914	-0.264709
H	1.522143	-4.255938	0.257203
H	0.853299	-0.148046	-0.799737
I	3.794396	1.10863	-0.546502
S	-0.70385	-2.559527	-0.45062
O	-0.820434	-3.980298	-0.693867
O	-1.299463	-2.148943	0.836784
O	-1.149671	-1.70429	-1.535924
C	9.269832	0.183083	0.176721
C	10.301135	-0.891248	0.516946
H	10.127473	-1.317988	1.506931
H	10.295833	-1.701346	-0.215205
H	11.295852	-0.442397	0.514477
C	9.360273	1.297819	1.227755
H	8.677296	2.11892	1.004331
H	9.122556	0.913816	2.222139
H	10.375612	1.699613	1.248748
C	9.602901	0.760021	-1.205953
H	9.538905	-0.013106	-1.974729
H	8.925771	1.571436	-1.477344
H	10.620473	1.156609	-1.201918
C	-6.786887	-0.487198	0.142884
C	-5.508786	0.023175	0.163354
C	-5.189184	1.353496	-0.103357
C	-6.25309	2.198645	-0.403353
C	-7.551054	1.711068	-0.427654
C	-7.848293	0.372967	-0.157484
H	-6.970497	-1.533061	0.355334
H	-6.069961	3.243734	-0.618182
H	-8.347319	2.402312	-0.66442
C	-3.778847	1.732736	-0.027659
C	-3.266821	3.014206	-0.237513
C	-2.856742	0.741947	0.306898
C	-1.911332	3.258778	-0.100642
H	-3.936282	3.822427	-0.501171
C	-1.508695	0.94647	0.482438
C	-1.042961	2.235893	0.264729
H	-1.522119	4.255931	-0.25714
H	-0.853295	0.148016	0.799712
I	-3.794411	-1.108634	0.546485
S	0.703867	2.55948	0.450618
O	0.820479	3.980249	0.693869
O	1.149697	1.70423	1.535909
O	1.29945	2.148893	-0.836797
C	-9.269839	-0.183039	-0.176724
C	-10.301133	0.891279	-0.517022
H	-10.295839	1.701416	0.215087
H	-10.127455	1.317966	-1.507028
H	-11.295852	0.442432	-0.514544
C	-9.602948	-0.759917	1.205965
H	-8.925836	-1.57133	1.477406
H	-9.538957	0.013242	1.974711
H	-10.620525	-1.156489	1.201923
C	-9.360258	-1.297821	-1.227711
H	-9.122515	-0.913863	-2.222107
H	-8.677288	-2.118913	-1.004232
H	-10.375597	-1.699613	-1.248712

2 Monomer
Eopt -1697.3888
ΔG -1697.0504

C	3.62755	0.6748560	-0.0076870
C	2.967474	1.9032080	-0.0093070
C	1.587886	1.9708860	-0.0089710
C	0.823907	0.7986960	-0.0070440
C	1.536966	-0.3919780	-0.0069040
C	2.91131	-0.5099270	-0.0073240
C	-0.639499	0.7298780	-0.0048350
C	-1.259726	-0.5237870	-0.0023040
I	0.230339	-2.0140170	-0.0057210
C	-1.46571	1.8462110	-0.0037570
C	-2.848677	1.7111610	-0.0000770
C	-3.391756	0.4273060	0.0028320
C	-2.629	-0.7466710	0.0019290

H	3.547268	2.8175870	-0.0120120
H	-1.014937	2.8296980	-0.0056170
H	3.424567	-1.4618380	-0.0081150
O	5.810523	1.4083530	-1.1630140
O	5.767921	-0.7713800	-0.0703880
H	1.098125	2.9356310	-0.0104190
S	5.421898	0.6378770	0.0065940
O	5.784173	1.2681460	1.2656160
H	-4.464021	0.3252640	0.0061380
C	-3.302644	-2.1272850	0.0063590
C	-3.720891	2.9656540	0.0007600
C	-3.400884	3.7972180	1.2504990
H	-2.355441	4.1086850	1.2699980
H	-4.021025	4.6960940	1.2606400
H	-3.607381	3.2277560	2.1593010
C	-3.408072	3.7934460	-1.2532890
H	-3.620438	3.2214710	-2.1591580
H	-4.027719	4.6926790	-1.2622680
H	-2.362539	4.1041040	-1.2799940
C	-5.212995	2.6374600	0.0055690
H	-5.504641	2.0678070	-0.8794500
H	-5.499446	2.0700540	0.8937260
H	-5.78095	3.5692650	0.0060410
C	-4.829	-2.0004130	0.0096830
H	-5.190476	-1.4782520	0.8969880
H	-5.194811	-1.4829130	-0.8785750
H	-5.255849	-3.0037050	0.0133650
C	-2.925161	-2.9141430	-1.2562360
H	-3.184039	-2.3499230	-2.1536720
H	-1.86899	-3.1785900	-1.3230720
H	-3.478596	-3.8545910	-1.2664560
C	-2.9192	-2.9083520	1.2707680
H	-1.862232	-3.1701380	1.3351300
H	-3.176202	-2.3409400	2.1667340
H	-3.470793	-3.8498130	1.2867640

2 Dimer
Eopt -3394.8174
ΔG -3394.1129

C	-0.679904	2.4339530	-1.5743280
C	-1.190266	3.2938290	-0.6062380
C	-2.292728	2.9291680	0.1459970
C	-2.895314	1.6816690	-0.0374020
C	-2.335944	0.8595380	-1.0095890
C	-1.260871	1.1960370	-1.8018950
C	-4.053478	1.1738650	0.7042250
C	-4.507622	-0.1168880	0.4106560
I	-3.276668	-0.9946280	-1.0471620
C	-4.730383	1.9080010	1.6696680
C	-5.829298	1.3704690	2.3274940
C	-6.238863	0.0809100	1.9887890
C	-5.606845	-0.7106850	1.0239860
H	-0.714187	4.2509710	-0.4374430
H	-4.392205	2.9089890	1.9037660
H	-0.891809	0.5250620	-2.5620770
O	0.20028	3.6141230	-3.7080650
O	1.38382	1.6451450	-2.9067560
H	-2.679462	3.6108420	0.8915460
S	0.732006	2.9192210	-2.5568360
O	1.558936	3.7292940	-1.6795280
H	-7.092625	-0.3319630	2.4995830
C	-6.148441	-2.1043500	0.6719710
C	-6.547701	2.2045550	3.3875880
C	-5.557879	2.5512180	4.5082060
H	-4.71127	3.1276720	4.1320460
H	-6.061074	3.1486210	5.2716260
H	-5.173275	1.6446600	4.9806920
C	-7.059213	3.5011190	2.7449040
H	-7.760773	3.2831680	1.9366450
H	-7.576447	4.1034690	3.4949210
H	-6.241784	4.0982950	2.3379240
C	-7.736609	1.4674270	4.0019880
H	-8.489159	1.2177960	3.2509670
H	-7.427501	0.5471500	4.5022460
H	-8.207297	2.1114060	4.7468160
C	-7.237161	-2.5449890	1.6558730
H	-6.859138	-2.5932160	2.6793050
H	-8.103475	-1.8831550	1.6317230
H	-7.576154	-3.5416650	1.3720230
C	-6.780968	-2.0622840	-0.7266640
H	-7.623328	-1.3685300	-0.7447610
H	-6.078689	-1.7547780	-1.5038060
H	-7.145435	-3.0574590	-0.9898220
C	-5.041599	-3.1725490	0.7179140
H	-4.429812	-3.2159150	-0.1856670
H	-4.385345	-3.0398550	1.5800200
H	-5.503107	-4.1583990	0.7901010
C	0.679948	-2.4332700	-1.5754030
C	1.18994	-3.2933320	-0.6072960
C	2.292336	-2.9289670	0.1451950
C	2.895159	-1.6815540	-0.0379270
C	2.336099	-0.8591720	-1.0100970
C	1.261184	-1.1954210	-1.8027200
C	4.0533	-1.1740700	0.7039570
C	4.507639	0.1166890	0.4107380

I	3.276976	0.9949390	-1.0470050
C	4.730032	-1.9085160	1.6692850
C	5.828957	-1.3712720	2.3273410
C	6.238696	-0.0816720	1.9890020
C	5.606849	0.7102160	1.0243310
H	0.713622	-4.2503870	-0.4386910
H	4.391725	-2.9095280	1.9030910
H	0.892512	-0.5243490	-2.5630170
O	-0.2001	-3.6123490	-3.7098320
O	-1.38387	-1.6439680	-2.9074220
H	2.678811	-3.6107940	0.8907380
S	-0.73189	-2.9181570	-2.5582060
O	-1.558736	-3.7288090	-1.6813590
H	7.092452	0.3309960	2.4999790
C	6.14862	2.1038800	0.6726670
C	6.547185	-2.2057060	3.3872790
C	5.557283	-2.5524350	4.5078080
H	4.710599	-3.1286950	4.1315230
H	6.060373	-3.1500570	5.2711260
H	5.172803	-1.6459100	4.9804560
C	7.058492	-3.5022200	2.7443330
H	7.760123	-3.2842190	1.9361480
H	7.575588	-4.1048270	3.4942390
H	6.240973	-4.0991600	2.3371890
C	7.736202	-1.4689030	4.0018570
H	8.48883	-1.2192800	3.2509110
H	7.427241	-0.5486540	4.5022590
H	8.206742	-2.1130970	4.7465930
C	7.236989	2.5443270	1.6570440
H	6.858578	2.5924110	2.6803410
H	8.103282	1.8824550	1.6331300
H	7.576143	3.5410240	1.3734690
C	6.781641	2.0618420	-0.7257520
H	7.624011	1.3680930	-0.7435770
H	6.079588	1.7543370	-1.5031070
H	7.146185	3.0570250	-0.9887730
C	5.041784	3.1721230	0.7181970
H	4.430764	3.2158680	-0.1858930
H	4.384822	3.0391590	1.5797210
H	5.503263	4.1579290	0.7911310

3 Monomer
Eopt -834.2558
ΔG -834.1409

C	0.745434	3.393578	0
C	2.036443	2.888774	0
C	2.330306	1.522194	0
C	1.263007	0.624649	0
C	0	1.185823	0
C	-0.40269	2.537183	0
C	1.314196	-0.84033	0
C	0.100141	-1.53264	0
I	-1.53051	-0.20968	0
C	2.475536	-1.61334	0
C	2.394466	-2.99631	0
C	1.161411	-3.64169	0
C	-0.01728	-2.90691	0
H	0.57751	4.464321	0
H	2.861591	3.591939	0
H	3.352356	1.169886	0
H	3.440231	-1.12188	0
H	3.304092	-3.58252	0
H	1.110671	-4.72253	0
H	-0.97977	-3.40008	0
O	-1.61947	2.873504	0

3 Dimer
Eopt -1668.4959
ΔG -1668.2424

C	-2.12052	3.499067	0.000244
C	-3.47643	3.771445	0.000118
C	-4.45289	2.774415	-0.00002
C	-4.03456	1.446549	-2.5E-05
C	-2.66718	1.225426	0.000108
C	-1.61833	2.163432	0.000243
C	-4.87128	0.24653	-0.00016
C	-4.2109	-0.98565	-0.00012
I	-2.12577	-0.78254	0.000102
C	-6.26599	0.223896	-0.00032
C	-6.94797	-0.98038	-0.00043
C	-6.2587	-2.18921	-0.00038
C	-4.87144	-2.20008	-0.00023
H	-1.39435	4.30176	0.000349
H	-3.79398	4.807202	0.000127
H	-5.50378	3.026555	-0.00011
H	-6.8115	1.158461	-0.00037
H	-8.0299	-0.9813	-0.00056
H	-6.80038	-3.12545	-0.00047
H	-4.32795	-3.13541	-0.0002
C	2.120528	-3.49907	0.000178
C	3.476431	-3.77145	0.000067
C	4.452891	-2.77441	-4.9E-05
C	4.034561	-1.44655	-4.6E-05
C	2.667176	-1.22543	0.000075
C	1.618336	-2.16344	0.000185
C	4.871284	-0.24653	-0.00016

C	4.210894	0.985654	-0.00012
I	2.125772	0.782542	0.000088
C	6.265993	-0.22389	-0.00031
C	6.947969	0.980388	-0.0004
C	6.258695	2.189216	-0.00035
C	4.871432	2.20008	-0.00021
H	1.394351	-4.30176	0.000265
H	3.793985	-4.8072	0.000069
H	5.503782	-3.02655	-0.00012
H	6.811502	-1.15846	-0.00036
H	8.029895	0.981307	-0.00051
H	6.800378	3.125454	-0.00042
H	4.327943	3.135407	-0.00017
O	0.39938	-1.81665	0.000277
O	-0.39938	1.816641	0.000348

4 Monomer
Eopt -834.2453
ΔG -834.1314

C	3.432512	0.578093	-0.0001
C	2.856191	1.902675	-7.2E-05
C	1.506415	2.13466	-1.4E-05
C	0.567636	1.08345	0.000006
C	1.149195	-0.1854	-4.8E-05
C	2.471368	-0.50627	-0.00011
C	-0.87529	1.203368	0.000065
C	-1.64187	0.035785	0.000068
I	-0.39198	-1.64501	-2.2E-05
C	-1.60841	2.397688	0.000121
C	-2.9923	2.377324	0.000177
C	-3.70145	1.17812	0.000177
C	-3.01768	-0.03071	0.000122
H	3.546367	2.737893	-9.4E-05
H	1.140053	3.155191	0.000014
H	-1.07965	3.342372	0.000122
H	-3.53292	3.315096	0.00022
H	-4.78308	1.177721	0.00022
H	-3.54667	-0.974	0.000119
H	2.833947	-1.52482	-0.00015
O	4.664845	0.367901	-0.0002

4 Dimer
Eopt -1668.5093
ΔG -1668.2557

C	-0.41481	-2.85079	-1.0744
C	-1.2226	-3.57451	-0.12854
C	-2.28379	-3.00618	0.527509
C	-2.64948	-1.65858	0.331925
C	-1.8657	-0.97959	-0.59705
C	-0.83003	-1.49592	-1.32495
C	-3.7417	-0.94954	0.973134
C	-3.92478	0.397125	0.65045
I	-2.44909	1.055114	-0.68066
C	-4.6554	-1.48723	1.886912
C	-5.6637	-0.70125	2.418752
C	-5.79974	0.638873	2.064536
C	-4.91324	1.211304	1.161598
H	-0.95789	-4.60724	0.064226
H	-2.85414	-3.60113	1.231941
H	-4.57077	-2.5275	2.174404
H	-6.35915	-1.13978	3.122869
H	-6.59263	1.241118	2.487367
H	-5.00054	2.251357	0.876562
C	0.414601	2.850353	-1.07497
C	1.222052	3.574219	-0.12893
C	2.28321	3.006081	0.527331
C	2.649191	1.658555	0.33179
C	1.865772	0.979444	-0.5974
C	0.830186	1.495601	-1.32556
C	3.741429	0.949728	0.97321
C	3.92484	-0.39689	0.650556
I	2.449457	-1.05518	-0.68074
C	4.65486	1.487612	1.887143
C	5.663228	0.701827	2.419157
C	5.799599	-0.63826	2.064966
C	4.913378	-1.21088	1.161871
H	0.957081	4.606882	0.0638
H	2.853285	3.601121	1.231907
H	4.56999	2.527858	2.174614
H	6.358474	1.140503	3.123393
H	6.59254	-1.24035	2.487934
H	5.000948	-2.25091	0.876844
H	-0.32941	-0.95137	-2.1124
H	0.329989	0.951091	-2.11331
O	-0.60377	3.333564	-1.63847
O	0.60354	-3.33419	-1.63777

5 Monomer
Eopt -947.6368
ΔG -947.5119

C	0.255713	3.250052	0
C	1.635196	3.055604	0
C	2.190209	1.781854	0
C	1.359827	0.658847	0
C	0	0.918707	0
C	-0.59725	2.153165	0
C	1.755535	-0.75573	0

C	0.721499	-1.69563	0
I	-1.17383	-0.77309	0
C	3.068017	-1.2276	0
C	3.312166	-2.59142	0
C	2.261206	-3.50441	0
C	0.945434	-3.05978	0
H	-0.16753	4.246147	0
H	2.292218	3.91566	0
H	3.265816	1.660711	0
H	3.894579	-0.52819	0
H	4.333365	-2.94976	0
H	2.46532	-4.56727	0
H	0.125081	-3.76631	0
C	-2.11402	2.239781	0
O	-2.64311	3.345328	0
O	-2.70156	1.107419	0

5 Dimer
Eopt -1895.2849
ΔG -1895.0126

C	4.844289	-3.509	0.000786
C	5.465025	-2.26668	0.000785
C	4.695626	-1.10156	0.000416
C	3.320062	-1.27711	0.000064
C	5.175737	0.285277	0.000375
C	4.196691	1.282469	-1.4E-05
I	2.244067	0.485161	-0.00047
C	6.51429	0.677258	0.000702
C	6.839687	2.024014	0.000632
C	5.844224	2.997316	0.000236
C	4.504588	2.631014	-8.9E-05
H	5.453988	-4.40321	0.00107
H	6.545543	-2.20159	0.001074
H	7.297673	-0.07041	0.001018
H	7.880344	2.321246	0.000889
H	6.110395	4.04639	0.00018
H	3.728795	3.386532	-0.0004
C	3.456632	-3.62597	0.000433
C	2.663665	-2.48494	0.000067
H	2.978718	-4.59697	0.000444
C	-2.66374	2.484958	0.000105
C	-3.32009	1.277109	0.000123
C	-3.45675	3.625964	0.000453
C	-4.69565	1.101514	0.000455
I	-2.24404	-0.48512	-0.00032
C	-4.8444	3.50895	0.000795
H	-2.97887	4.596979	0.000453
C	-5.46509	2.266609	0.000797
C	-5.17572	-0.28534	0.000397
C	-4.19664	-1.2825	0.000015
H	-5.45413	4.40314	0.001057
H	-6.54561	2.201475	0.001064
C	-6.51425	-0.67737	0.000676
C	-4.50448	-2.63106	-0.0001
C	-6.8396	-2.02414	0.000567
H	-7.29767	0.070267	0.000979
C	-5.84411	-2.99741	0.000178
H	-3.72866	-3.38654	-0.0004
H	-7.88025	-2.32141	0.000785
H	-6.11024	-4.04649	0.000094
C	1.146223	-2.50584	-0.00032
C	-1.1463	2.505919	-0.00028
O	0.611428	-1.34852	-0.00062
O	0.560623	-3.58376	-0.00043
O	-0.56075	3.583868	-0.00025
O	-0.61145	1.348625	-0.00071

6 Monomer
Eopt -947.6084
ΔG -947.4854

C	-2.85815	0.503223	-1.5E-05
C	-2.38465	1.813939	-0.00014
C	-1.02981	2.100974	-8.2E-05
C	-0.09315	1.066189	0.000057
C	-0.61781	-0.22422	0.000193
C	-1.95153	-0.55232	0.000149
C	1.360818	1.220106	0.000016
C	2.141953	0.067066	-2.8E-05
I	0.931759	-1.64004	-0.00004
C	2.054553	2.43144	0.000085
C	3.439282	2.44271	0.000068
C	4.169043	1.257576	0.000002
C	3.518671	0.030432	-1.1E-05
H	-3.10982	2.616681	-0.0003
H	1.502166	3.362058	0.000154
H	3.961118	3.390475	0.000107
H	5.250657	1.281357	-2.3E-05
H	4.071997	-0.89859	-4.7E-05
H	-2.31759	-1.56986	0.000357
C	-4.36896	0.199025	0
O	-5.11798	1.192588	-9.6E-05
O	-4.67388	-1.00823	0.000129
H	-0.68847	3.128223	-0.00015

6 Dimer
Eopt -1895.2663
ΔG -1894.9917

C	-0.86605	-2.31304	-0.40473
C	-1.66346	-3.19377	0.325377
C	-2.94594	-2.84551	0.717038
C	-3.45714	-1.57636	0.428578
C	-2.62006	-0.72534	-0.28706
C	-1.37127	-1.06471	-0.74534
C	-4.77634	-1.06563	0.807783
C	-5.06653	0.248833	0.441511
I	-3.45501	1.178876	-0.53121
C	-5.7568	-1.78311	1.494046
C	-6.97077	-1.18531	1.789132
C	-7.22922	0.129806	1.411618
C	-6.26917	0.866458	0.730734
H	-1.26094	-4.16305	0.588606
H	-5.56795	-2.80589	1.793973
H	-7.72701	-1.74864	2.32018
H	-8.18178	0.585848	1.648051
H	-6.46494	1.890361	0.438998
C	0.866113	2.312955	-0.4057
C	1.66321	3.193602	0.324854
C	2.945608	2.845421	0.716808
C	3.456976	1.576341	0.428315
C	2.620177	0.725356	-0.28768
C	1.371573	1.064763	-0.74647
C	4.776147	1.065704	0.807771
C	5.066537	-0.2487	0.441444
I	3.455169	-1.1789	-0.53138
C	5.75641	1.783233	1.49426
C	6.970384	1.185532	1.78952
C	7.229032	-0.12953	1.411961
C	6.269182	-0.86623	0.730856
H	1.26048	4.162779	0.588163
H	5.56741	2.805977	1.794222
H	7.726476	1.748901	2.320738
H	8.181601	-0.58549	1.648523
H	6.465104	-1.8901	0.439085
H	-0.80963	-0.37164	-1.34233
H	0.810324	0.371884	-1.34408
C	-0.57587	2.657173	-0.75985
C	0.575936	-2.65733	-0.75879
O	-1.05248	3.700431	-0.31347
O	1.052578	-3.70046	-0.31215
O	1.188892	-1.79303	-1.46754
O	-1.18889	1.792632	-1.46825
H	3.552348	3.551504	1.269204
H	-3.55288	-3.55163	1.26917

14 Monomer
Eopt -1461.5469
ΔG -1461.3722

C	2.713586	0.5257580	-0.0085730
C	2.185327	1.8159340	-0.0099160
C	0.819799	2.0258040	-0.0084450
C	-0.060298	0.9401040	-0.0054930
C	0.525271	-0.3214320	-0.0053040
C	1.879004	-0.5802590	-0.0069610
C	-1.520495	1.0217830	-0.0027710
C	-2.235232	-0.1726640	-0.0001250
I	-0.939975	-1.8169670	-0.0019480
C	-2.275252	2.1907710	-0.0015930
C	-3.666195	2.1381880	0.0021170
C	-4.308535	0.8996990	0.0047340
C	-3.607722	-0.3054090	0.0037740
H	2.856795	2.6653660	-0.0134340
H	-1.774872	3.1519600	-0.0032530
H	2.291182	-1.5799890	-0.0078720
O	4.959315	1.0349420	-1.1631730
O	4.694547	-1.1328950	-0.0775630
H	0.424889	3.0331710	-0.0099940
S	4.494852	0.3041620	0.0042250
O	4.919686	0.8897790	1.2651740
H	-5.391772	0.8617320	0.0078150
C	-4.282222	-1.6414970	0.0070830
H	-4.002296	-2.2182680	-0.8791930
H	-3.9985	-2.2156710	0.8938320
H	-5.364198	-1.5290470	0.0092700
C	-4.460269	3.4123320	0.0034350
H	-4.21673	4.0129690	0.8815600
H	-4.22252	4.0111550	-0.8775150
H	-5.529924	3.2100040	0.0071530

14 Dimer
Eopt -2923.1334
ΔG -2922.7578

C	0.697765	2.4466030	-0.9994690
C	1.228932	3.3272110	-0.0620040
C	2.344623	2.9767680	0.6776060
C	2.939871	1.7236490	0.5125920
C	2.358686	0.8794860	-0.4320040
C	1.271223	1.2019230	-1.2114180
C	4.106723	1.2252670	1.2425460
C	4.529253	-0.0655510	0.9460870
I	3.311993	-0.9792280	-0.4820440
C	4.830356	1.9347290	2.2021370
C	5.926781	1.3568210	2.8254020
C	6.30246	0.0530220	2.4837720

C	5.614731	-0.6951790	1.5370600
H	0.759588	4.2900830	0.0925480
H	4.537289	2.9441850	2.4621280
C	-0.697719	-2.4467160	-0.9990810
C	-1.229449	-3.3275370	-0.0621200
C	-2.34527	-2.9770540	0.6772630
C	-2.94019	-1.7237440	0.5124530
C	-2.358532	-0.8794350	-0.4317110
C	-1.270802	-1.2018300	-1.2107740
C	-4.107111	-1.2252970	1.2422500
C	-4.529265	0.0657010	0.9460370
I	-3.311611	0.9794050	-0.4817380
C	-4.831129	-1.9348430	2.2014880
C	-5.927556	-1.3568370	2.8246620
C	-6.302847	-0.0528570	2.4832940
C	-5.614733	0.6954240	1.5369270
H	-0.760445	-4.2906070	0.0922420
H	-4.538365	-2.9444400	2.4612760
H	0.888268	0.5171930	-1.9519260
H	-0.887313	-0.5168730	-1.9507790
O	1.54579	-3.7338430	-1.0832430
O	-1.545492	3.7340990	-1.0842450
O	-1.384744	1.6386070	-2.2942060
O	1.384745	-1.6388800	-2.2940860
H	-2.75145	-3.6742000	1.3983730
H	2.750414	3.6737790	1.3990650
S	-0.730646	2.9175250	-1.9656310
S	0.730836	-2.9177540	-1.9649830
O	-0.217309	3.6010820	-3.1317720
O	0.217625	-3.6019070	-3.1308320
H	-7.161369	0.3949570	2.9720210
H	7.160977	-0.3947210	2.9725740
C	6.011598	-2.0924660	1.1700320
H	5.203995	-2.7968730	1.3890410
H	6.243952	-2.1634870	0.1036770
H	6.891602	-2.4052570	1.7285090
C	6.71431	2.1108700	3.8576480
H	7.754776	2.2149650	3.5442780
H	6.300882	3.1048860	4.0207950
H	6.713161	1.5735450	4.8077680
C	-6.011158	2.0929160	1.1701990
H	-5.203554	2.7970870	1.3899510
H	-6.242884	2.1643960	0.1037380
H	-6.891435	2.4056450	1.7282810
C	-6.715491	-2.1109630	3.8565420
H	-7.755969	-2.2145160	3.5430360
H	-6.302478	-3.1052060	4.0193560
H	-6.714212	-1.5740000	4.8068670

30 Monomer

Eopt -1697.3859

ΔG -1697.0470

C	2.106674	-2.1662290	0.1279840
C	2.698321	-1.2044060	-0.6976830
C	1.904288	-0.1791600	-1.2068780
C	0.572068	-0.1806680	-0.8593420
C	-0.056271	-1.1138570	-0.0420870
C	0.764133	-2.1301350	0.4542710
H	2.722435	-2.9671200	0.5201600
H	2.332403	0.5807940	-1.8476900
H	0.341156	-2.8940920	1.0937670
C	-2.141183	0.1194380	-0.3761960
C	-3.480123	0.4181960	-0.2482980
C	-4.224131	-0.4397370	0.5454210
C	-3.624318	-1.5355560	1.1643250
C	-2.276755	-1.7954290	1.0044330
C	-1.485918	-0.9562470	0.2135530
H	-3.946624	1.2656780	-0.7315280
H	-4.226702	-2.1938160	1.7776860
H	-1.828208	-2.6513790	1.4914420
I	-0.802526	1.2575950	-1.5143880
S	-5.975155	-0.1342830	0.7963900
O	-6.089386	0.1520810	2.2171320
O	-6.620594	-1.3742850	0.3985980
O	-6.283739	0.9971420	-0.0617090
C	4.168364	-1.2625790	-1.0019650
H	4.398712	-0.5841190	-1.8280940
H	4.405753	-2.2758700	-1.3310280
C	5.054171	-0.9011440	0.2043960
H	4.850839	-1.6315710	0.9981890
C	6.53646	-1.0069350	-0.1656080
H	6.772703	-0.2192450	-0.8903480
H	7.12982	-0.7950260	0.7259910
C	6.959953	-2.3625180	-0.7199850
H	6.550737	-2.5471280	-1.7140040
H	8.046695	-2.4229740	-0.7983950
H	6.62779	-3.1721570	-0.0646790
C	4.708259	0.4915370	0.7376490
H	4.831688	1.2157590	-0.0758770
H	3.648682	0.5160140	1.0141920
C	5.527989	0.9240670	1.9517140
H	5.585404	0.0878560	2.6565720
H	6.554985	1.1494530	1.6503880
C	4.947318	2.1428890	2.6661170
H	3.945984	1.8994380	3.0349670

H	5.558174	2.3633840	3.5453550
C	4.87511	3.3822610	1.7816990
H	5.856182	3.6139590	1.3585490
H	4.17934	3.2430600	0.9522540
H	4.541689	4.2528540	2.3491540

30 Dimer

Eopt -3394.8124

ΔG -3394.1072

C	-5.36345	1.682568	-2.34361
C	-6.03615	1.362398	-1.15881
C	-5.51175	0.365793	-0.3402
C	-4.34924	-0.26392	-0.73951
C	-3.66014	0.037973	-1.91047
C	-4.19867	1.04073	-2.71957
H	-5.77282	2.454397	-2.98504
H	-6.01214	0.101351	0.583487
H	-3.70486	1.310609	-3.64452
C	-2.08096	-1.71837	-1.27741
C	-0.98356	-2.53785	-1.40586
C	-0.1673	-2.31976	-2.50681
C	-0.47581	-1.3377	-3.44234
C	-1.61024	-0.55872	-3.29365
C	-2.44703	-0.72906	-2.18897
H	-0.77134	-3.31325	-0.68599
H	0.181573	-1.18026	-4.2873
H	-1.84451	0.198247	-4.03046
I	-3.38836	-1.76189	0.353343
S	1.285941	-3.33955	-2.71244
O	2.248257	-2.51257	-3.4164
O	0.852236	-4.52067	-3.42402
O	1.716762	-3.63427	-1.33271
C	-7.2824	2.107751	-0.77199
H	-7.78607	1.582439	0.044451
H	-7.95801	2.108772	-1.62941
C	-7.01288	3.560457	-0.33918
H	-6.55012	4.080936	-1.18783
C	-8.3241	4.27767	-0.00572
H	-8.75286	3.822878	0.894766
H	-8.09443	5.313965	0.250105
C	-9.35579	4.272338	-1.12822
H	-9.77267	3.279179	-1.29939
H	-10.1868	4.938376	-0.89017
H	-8.91188	4.616035	-2.06639
C	-6.03534	3.604276	0.837961
H	-6.45806	3.018027	1.662097
H	-5.10542	3.10282	0.548866
C	-5.6883	5.011176	1.321077
H	-5.49562	5.647449	0.45074
H	-6.54197	5.449872	1.845634
C	-4.47342	5.046256	2.246123
H	-3.59795	4.67694	1.702842
H	-4.25958	6.084656	2.512368
C	-4.66181	4.229959	3.51956
H	-5.56223	4.546079	4.053019
H	-4.76071	3.164897	3.302139
H	-3.81318	4.351086	4.195011
C	5.36331	1.682598	2.343559
C	6.036078	1.36235	1.158821
C	5.51171	0.365709	0.340229
C	4.349167	-0.26396	0.739514
C	3.659994	0.038014	1.910411
C	4.198497	1.040807	2.719485
H	5.772668	2.454446	2.984982
H	6.01215	0.101206	-0.58341
H	3.704638	1.310747	3.644387
C	2.080881	-1.71842	1.277415
C	0.983535	-2.53795	1.405896
C	0.167196	-2.31976	2.506798
C	0.475592	-1.33758	3.442199
C	1.610001	-0.55855	3.293491
C	2.446877	-0.72899	2.188908
H	0.771408	-3.31343	0.686074
H	-0.18187	-1.18003	4.287076
H	1.844164	0.198519	4.030225
I	3.388348	-1.76201	-0.35329
S	-1.28598	-3.33962	2.71243
O	-2.24827	-2.51272	3.416509
O	-0.85217	-4.52076	3.423892
O	-1.71685	-3.63425	1.332682
C	7.28238	2.107642	0.772048
H	7.786076	1.582296	-0.04436
H	7.957942	2.108639	1.629507
C	7.012948	3.560356	0.339217
H	6.550146	4.080854	1.187827
C	8.324225	4.277522	0.00586
H	8.753043	3.822714	-0.89459
H	8.094617	5.313825	-0.24998
C	9.355825	4.272151	1.128446
H	9.772662	3.278979	1.299646
H	10.18689	4.93817	0.890477
H	8.911843	4.615848	2.066586
C	6.035514	3.604217	-0.83801
H	6.458299	3.017982	-1.66212
H	5.105556	3.102769	-0.54901

C	5.688549	5.011139	-1.32112
H	5.495824	5.647399	-0.45078
H	6.542274	5.44982	-1.8456
C	4.473738	5.046269	-2.24626
H	3.598225	4.676962	-1.70305
H	4.25994	6.08468	-2.51249
C	4.662212	4.229994	-3.5197
H	5.562682	4.546109	-4.05308
H	4.761074	3.164926	-3.30229
H	3.813634	4.351152	-4.19521

31 Monomer
Eopt -1928.4394
ΔG -1928.0255

C	-5.54081	0.651605	0.258245
C	-4.91409	1.790698	0.761693
C	-3.53657	1.8868	0.803512
C	-2.74314	0.833747	0.337699
C	-3.4259	-0.27552	-0.14978
C	-4.79487	-0.41694	-0.21276
C	-1.28352	0.802448	0.322755
C	-0.65413	-0.33849	-0.16589
I	-2.07565	-1.74473	-0.78632
C	-0.43326	1.819461	0.75817
C	0.938154	1.662144	0.695928
C	1.530206	0.494077	0.200485
C	0.703172	-0.53893	-0.24806
H	-5.51822	2.612406	1.125266
H	-0.85331	2.733809	1.155698
H	1.571426	2.460502	1.06035
H	1.122485	-1.44727	-0.65956
H	-5.28314	-1.29974	-0.6025
O	-7.71706	0.657902	1.632674
O	-7.64347	-0.70483	-0.38796
H	-3.06709	2.777901	1.199844
S	-7.33421	0.571876	0.232987
O	-7.73615	1.72643	-0.55288
C	3.00361	0.346538	0.152263
C	3.597572	-0.88776	0.405828
C	3.80871	1.445234	-0.1409
C	4.98135	-1.04028	0.373697
H	2.968435	-1.73395	0.655839
C	5.195434	1.328753	-0.18649
H	3.341306	2.39822	-0.35741
C	5.759859	0.079068	0.074745
H	6.833661	-0.02544	0.045353
C	6.039121	2.559153	-0.52424
C	5.591386	-2.41061	0.67327
C	5.189737	-2.84581	2.089095
H	4.106474	-2.92628	2.191677
H	5.623758	-3.82288	2.313975
H	5.552024	-2.13139	2.831868
C	5.058903	-3.43502	-0.33788
H	5.489932	-4.41772	-0.13273
H	3.972774	-3.52531	-0.28331
H	5.327969	-3.14961	-1.35748
C	7.117045	-2.39866	0.588303
H	7.46388	-2.12	-0.40909
H	7.556417	-1.7082	1.311391
H	7.49545	-3.39885	0.807497
C	7.537181	2.258333	-0.51106
H	7.871499	1.915025	0.470325
H	7.803642	1.5003	-1.25061
H	8.087214	3.169754	-0.75277
C	5.659516	3.064795	-1.92273
H	4.606841	3.34739	-1.97459
H	6.258144	3.943217	-2.17498
H	5.845198	2.296199	-2.67651
C	5.759712	3.664557	0.50317
H	4.709704	3.961257	0.498757
H	6.017338	3.331544	1.511206
H	6.360264	4.547082	0.270695

31 Dimer
Eopt -3856.9203
ΔG -3856.0619

C	0.21537	-2.60341	2.500444
C	0.539623	-1.61867	3.427605
C	1.668578	-0.83691	3.254151
C	2.482452	-1.00756	2.132745
C	2.101497	-1.99975	1.230443
C	1.009761	-2.82223	1.383539
C	3.686918	-0.23774	1.828516
C	4.355881	-0.54308	0.645453
I	3.374086	-2.04217	-0.42815
C	4.234211	0.771769	2.620589
C	5.388866	1.418642	2.222799
C	6.043692	1.092815	1.028178
C	5.50643	0.08728	0.221968
H	-0.10099	-1.46134	4.285332
H	3.75911	1.043154	3.554675
H	5.807775	2.18378	2.863612
H	5.977133	-0.17252	-0.7175
H	0.784874	-3.60021	0.670451
O	-0.782	-4.80808	3.433687
O	-1.69378	-3.91563	1.365202

H	1.916371	-0.0779	3.984323
S	-1.23214	-3.62506	2.736289
O	-2.17904	-2.80042	3.463147
C	7.282877	1.797217	0.621976
C	8.300169	1.112151	-0.03942
C	7.442706	3.15353	0.898476
C	9.471121	1.758847	-0.42674
H	8.181857	0.052578	-0.23329
C	8.598678	3.833696	0.523891
H	6.641229	3.687883	1.394457
C	9.598972	3.118091	-0.13615
H	10.5008	3.632414	-0.43107
C	8.726522	5.324495	0.841685
C	10.56374	0.965004	-1.14521
C	11.02822	-0.19037	-0.24804
H	10.21093	-0.87666	-0.02098
H	11.81435	-0.75756	-0.75193
H	11.43001	0.187685	0.694847
C	9.996131	0.390967	-2.4506
H	10.76842	-0.1786	-2.97295
H	9.154002	-0.27689	-2.26193
H	9.656408	1.19209	-3.11107
C	11.77881	1.826413	-1.48686
H	11.51509	2.651474	-2.15196
H	12.24433	2.241474	-0.59034
H	12.5224	1.210432	-1.99605
C	10.0542	5.909854	0.362456
H	10.90663	5.421388	0.839356
H	10.16742	5.821265	-0.72015
H	10.09028	6.970742	0.617211
C	7.586046	6.089826	0.156852
H	6.609289	5.750771	0.5055
H	7.6711	7.156773	0.375995
H	7.62706	5.958825	-0.92692
C	8.628435	5.528043	2.359721
H	7.673616	5.172673	2.750596
H	9.42952	4.993447	2.875358
H	8.716983	6.590442	2.598755
C	-0.21538	-2.60349	-2.50037
C	-0.53951	-1.61862	-3.42743
C	-1.66844	-0.83684	-3.25399
C	-2.4824	-1.00756	-2.13266
C	-2.10155	-1.99986	-1.23043
C	-1.00987	-2.8224	-1.38355
C	-3.68687	-0.23774	-1.82846
C	-4.35593	-0.54316	-0.64547
I	-3.37421	-2.04232	0.428107
C	-4.23409	0.771852	-2.62048
C	-5.38875	1.418724	-2.22272
C	-6.04366	1.092821	-1.02816
C	-5.50649	0.087202	-0.222
H	0.101193	-1.46124	-4.28508
H	-3.75892	1.043301	-3.55451
H	-5.8076	2.183924	-2.86349
H	-5.97725	-0.17266	0.717415
H	-0.78519	-3.6005	-0.67051
O	0.78199	-4.80821	-3.43355
O	1.693792	-3.91566	-1.36513
H	-1.91615	-0.07774	-3.9841
S	1.232125	-3.62514	-2.73621
O	2.179007	-2.80054	-3.46313
C	-7.28284	1.797246	-0.62198
C	-8.3002	1.112189	0.039327
C	-7.44261	3.153576	-0.89843
C	-9.47114	1.758913	0.426616
H	-8.18193	0.052605	0.233166
C	-8.59857	3.833773	-0.52387
H	-6.64109	3.687916	-1.39437
C	-9.59892	3.118178	0.136089
H	-10.5007	3.632524	0.430993
C	-8.72635	5.324583	-0.84163
C	-10.5638	0.965089	1.145029
C	-11.0285	-0.19011	0.247732
H	-10.2113	-0.8765	0.020569
H	-11.8147	-0.75725	0.751577
H	-11.4302	0.188115	-0.6951
C	-9.9962	0.39081	2.450309
H	-10.7685	-0.17877	2.972612
H	-9.15413	-0.27708	2.261497
H	-9.65637	1.191806	3.110878
C	-11.7788	1.826583	1.486867
H	-11.5149	2.651484	2.152119
H	-12.2443	2.241873	0.590435
H	-12.5224	1.210598	1.995952
C	-10.0541	5.909937	-0.36253
H	-10.9065	5.421525	-0.83958
H	-10.1674	5.821273	0.720047
H	-10.0901	6.970843	-0.61722
C	-7.58595	6.089892	-0.15665
H	-6.60916	5.750863	-0.50521
H	-7.67099	7.156847	-0.37576
H	-7.62708	5.958848	0.927111
C	-8.62809	5.528166	-2.35965
H	-7.67324	5.172778	-2.75042

H	-9.42913	4.993601	-2.87539
H	-8.71659	6.590573	-2.59867

Calculation in DCM:

1 Monomer
Eopt -1540.1520
ΔG -1539.9220

C	2.942729	-0.44147	-0.00039
C	1.579999	-0.27804	0.000944
C	0.901007	0.938083	0.006096
C	1.713477	2.068839	0.022395
C	3.094887	1.948313	0.020309
C	3.738833	0.70912	0.004223
H	3.390103	-1.42627	-0.00518
H	1.263396	3.053132	0.038537
H	3.680148	2.856398	0.03166
C	-0.55997	0.914192	-0.00336
C	-1.40161	2.030396	-0.02014
C	-1.19439	-0.32326	-0.00028
C	-2.77389	1.869394	-0.02289
H	-0.97341	3.023893	-0.03451
C	-2.55521	-0.53543	-0.0032
C	-3.34928	0.59993	-0.01134
H	-3.41627	2.740922	-0.03607
H	-3.00469	-1.51909	-0.00054
I	0.217653	-1.87158	0.00028
S	-5.13931	0.442533	0.00289
O	-5.57159	1.178492	-1.17206
O	-5.38564	-0.98724	-0.06475
O	-5.53662	1.056047	1.258212
C	5.257946	0.556809	-0.00346
C	5.966051	1.91036	-0.03038
H	5.703746	2.484791	-0.92115
H	5.729086	2.508011	0.851875
H	7.044671	1.746568	-0.0429
C	5.68043	-0.2409	-1.24488
H	5.255211	-1.24585	-1.24539
H	5.366877	0.266177	-2.15969
H	6.767785	-0.33864	-1.26281
C	5.693019	-0.20078	1.258314
H	5.398349	0.340817	2.15949
H	5.255398	-1.19966	1.29923
H	6.779491	-0.30994	1.263345

1 Dimer
Eopt -3080.3519
ΔG -3079.8624

C	6.776069	0.483602	-0.143168
C	5.496299	-0.023729	-0.162769
C	5.177347	-1.353984	0.10518
C	6.240857	-2.199444	0.405436
C	7.539369	-1.7136	0.427878
C	7.837648	-0.376175	0.156053
H	6.961277	1.52922	-0.355663
H	6.057204	-3.244002	0.622382
H	8.335546	-2.405027	0.664252
C	3.766515	-1.732663	0.029576
C	3.254159	-3.013803	0.239204
C	2.844861	-0.74171	-0.30584
C	1.898763	-3.258368	0.102679
H	3.923374	-3.822364	0.502227
C	1.497141	-0.946336	-0.482998
C	1.031428	-2.235408	-0.26353
H	1.507014	-4.254585	0.258687
H	0.841142	-0.149704	-0.804116
I	3.782463	1.109695	-0.543156
S	-0.715078	-2.559655	-0.45246
O	-0.831102	-3.980727	-0.688745
O	-1.312974	-2.143172	0.833151
O	-1.158106	-1.706449	-1.53942
C	9.25989	0.178887	0.173022
C	10.291407	-0.897321	0.507247
H	10.121689	-1.325955	1.49706
H	10.283321	-1.705645	-0.226791
H	11.28701	-0.450603	0.502525
C	9.354474	1.290296	1.227392
H	8.671579	2.112888	1.009337
H	9.118858	0.904228	2.221329
H	10.369762	1.692131	1.247383
C	9.590305	0.75947	-1.208841
H	9.523293	-0.010598	-1.980285
H	8.913934	1.572536	-1.477053
H	10.608065	1.155414	-1.207221
C	-6.776048	-0.483656	0.14324
C	-5.49629	0.023706	0.162815
C	-5.17737	1.353961	-0.105176
C	-6.240904	2.199389	-0.40544
C	-7.539405	1.713516	-0.427851
C	-7.837648	0.376088	-0.155999
H	-6.961237	-1.529265	0.355796
H	-6.057277	3.243946	-0.622416
H	-8.3356	2.40492	-0.66423
C	-3.766545	1.732671	-0.029611
C	-3.254212	3.013813	-0.239291
C	-2.844866	0.741743	0.305801

C	-1.898815	3.258401	-0.102815
H	-3.923447	3.822358	-0.502316
C	-1.497142	0.946385	0.482888
C	-1.031451	2.235459	0.263384
H	-1.507085	4.254619	-0.25886
H	-0.841147	0.149746	0.803984
I	-3.782428	-1.109658	0.543276
S	0.715057	2.559737	0.452252
O	0.831064	3.980819	0.68848
O	1.158136	1.706575	1.539224
O	1.312916	2.143219	-0.833366
C	-9.259876	-0.179008	-0.1729
C	-10.291461	0.897215	-0.506868
H	-10.283358	1.705415	0.227305
H	-10.121831	1.326023	-1.496621
H	-11.287046	0.450455	-0.50215
C	-9.590111	-0.759785	1.208922
H	-8.913641	-1.572828	1.476962
H	-9.523083	0.010192	1.980456
H	-10.607839	-1.155813	1.207355
C	-9.354548	-1.29028	-1.227406
H	-9.119038	-0.904076	-2.221317
H	-8.671622	-2.112893	-1.009534
H	-10.369832	-1.692126	-1.24735

2 Monomer
Eopt -1697.3854
ΔG -1697.0466

C	3.627971	0.676179	-0.00745
C	2.967533	1.904052	-0.01143
C	1.587844	1.971606	-0.01126
C	0.823728	0.799507	-0.00725
C	1.538309	-0.39015	-0.0055
C	2.911982	-0.50874	-0.00543
C	-0.63967	0.72917	-0.00479
C	-1.25999	-0.52448	-0.00231
I	0.231662	-2.01361	-0.00299
C	-1.46675	1.844755	-0.00337
C	-2.84955	1.710682	-2E-06
C	-3.3925	0.426756	0.00179
C	-2.6297	-0.74705	0.000934
H	3.549104	2.817285	-0.01555
H	-1.01606	2.828136	-0.0044
H	3.428053	-1.45917	-0.00441
O	5.806693	1.392423	-1.17498
O	5.763515	-0.77348	-0.05351
H	1.097916	2.936394	-0.01454
S	5.424066	0.637625	0.005487
O	5.783121	1.283616	1.256219
H	-4.46473	0.325336	0.004245
C	-3.30344	-2.12793	0.003406
C	-3.72097	2.965958	0.001868
C	-3.40076	3.795608	1.252996
H	-2.35504	4.105855	1.27407
H	-4.01904	4.695613	1.264479
H	-3.60788	3.226	2.161435
C	-3.40681	3.795198	-1.25104
H	-3.61854	3.225373	-2.15828
H	-4.02496	4.695327	-1.25973
H	-2.36114	4.105196	-1.27732
C	-5.21355	2.639274	0.005544
H	-5.50608	2.071664	-0.88049
H	-5.50181	2.071894	0.893118
H	-5.78121	3.571138	0.006798
C	-4.83006	-2.00247	0.007473
H	-5.19255	-1.48335	0.896114
H	-5.19745	-1.48446	-0.87979
H	-5.25683	-3.00575	0.009253
C	-2.92644	-2.9125	-1.26097
H	-3.19191	-2.34985	-2.15744
H	-1.86877	-3.16916	-1.33245
H	-3.47434	-3.85621	-1.27065
C	-2.91987	-2.91151	1.266431
H	-1.86216	-3.16948	1.332131
H	-3.17958	-2.34771	2.163859
H	-3.46873	-3.85462	1.28022

2 Dimer
Eopt -3394.8170
ΔG -3394.1121

C	-0.77015	2.438477	-1.47375
C	-1.35077	3.308372	-0.55498
C	-2.4819	2.934537	0.147558
C	-3.04508	1.668198	-0.03486
C	-2.41828	0.839089	-0.95818
C	-1.31123	1.183483	-1.70245
C	-4.22592	1.148298	0.661734
C	-4.63483	-0.159	0.374647
I	-3.31513	-1.03668	-1.00389
C	-4.96354	1.885306	1.579498
C	-6.07918	1.336202	2.197875
C	-6.44189	0.030073	1.868745
C	-5.74747	-0.76575	0.9518
H	-0.90626	4.280785	-0.38674
H	-4.65962	2.898616	1.80778
H	-0.88987	0.505699	-2.42825

O	0.201433	3.670294	-3.53543
O	1.342389	1.671756	-2.74349
H	-2.92361	3.624747	0.853315
S	0.683443	2.940555	-2.38582
O	1.47842	3.718443	-1.45178
H	-7.30787	-0.39303	2.349511
C	-6.23384	-2.18374	0.613864
C	-6.86722	2.175483	3.203611
C	-5.93886	2.586003	4.355021
H	-5.09814	3.184849	4.001639
H	-6.49474	3.182635	5.08152
H	-5.54172	1.706633	4.866673
C	-7.39301	3.43551	2.502149
H	-8.05371	3.171741	1.673534
H	-7.95911	4.04326	3.211343
H	-6.57989	4.047354	2.108721
C	-8.05712	1.417238	3.790273
H	-8.77295	1.127313	3.018243
H	-7.74104	0.519249	4.325537
H	-8.57609	2.06348	4.500126
C	-7.38247	-2.6147	1.531845
H	-7.07858	-2.62646	2.580612
H	-8.25586	-1.97025	1.425479
H	-7.68486	-3.62553	1.256666
C	-6.765	-2.21329	-0.82671
H	-7.61604	-1.53867	-0.93505
H	-6.01689	-1.92533	-1.56709
H	-7.09223	-3.22562	-1.07224
C	-5.10999	-3.22062	0.785757
H	-4.40792	-3.26056	-0.0493
H	-4.54613	-3.05431	1.705402
H	-5.54999	-4.21787	0.831964
C	0.77011	-2.43806	-1.47425
C	1.350649	-3.30815	-0.55561
C	2.481759	-2.93449	0.147052
C	3.044993	-1.66814	-0.0351
C	2.418267	-0.83881	-0.95828
C	1.311242	-1.18303	-1.70267
C	4.225853	-1.14844	0.661613
C	4.634812	0.158899	0.374776
I	3.315095	1.036983	-1.00346
C	4.963504	-1.88565	1.579197
C	6.079189	-1.33671	2.19763
C	6.441944	-0.03053	1.868738
C	5.747517	0.765477	0.951967
H	0.906097	-4.28058	-0.38758
H	4.65956	-2.89899	1.807309
H	0.889957	-0.50509	-2.42837
O	-0.20137	-3.66943	-3.53625
O	-1.34239	-1.67108	-2.74392
H	2.923426	-3.62486	0.852677
S	-0.68343	-2.93995	-2.3865
O	-1.47845	-3.71805	-1.45267
H	7.307959	0.392471	2.349542
C	6.233992	2.183426	0.614104
C	6.867197	-2.17622	3.203199
C	5.938757	-2.58705	4.354433
H	5.098042	-3.18577	4.000815
H	6.494573	-3.18389	5.080803
H	5.541602	-1.70781	4.866302
C	7.393039	-3.43607	2.501457
H	8.053788	-3.17209	1.672949
H	7.959103	-4.04399	3.210538
H	6.579958	-4.04783	2.107828
C	8.057056	-1.41813	3.790138
H	8.772908	-1.12796	3.018223
H	7.740938	-0.52031	4.32566
H	8.576006	-2.06457	4.49982
C	7.382223	2.614476	1.532552
H	7.077872	2.626253	2.581188
H	8.255692	1.970075	1.4266
H	7.684699	3.625304	1.257459
C	6.765725	2.212684	-0.82627
H	7.616986	1.538256	-0.93406
H	6.017917	1.924248	-1.56678
H	7.092765	3.225032	-1.07199
C	5.11002	3.220336	0.785226
H	4.409329	3.261068	-0.05097
H	4.544703	3.053428	1.703869
H	5.55005	4.217498	0.832905

3 Monomer
Eopt -834.2557
ΔG -834.1408

C	0.732855	3.395021	0
C	2.02546	2.895765	0
C	2.326638	1.530338	0
C	1.264201	0.627614	0
C	0	1.18495	0
C	-0.41139	2.533069	0
C	1.317818	-0.83729	0
C	0.104377	-1.53136	0
I	-1.52894	-0.2117	0
C	2.479983	-1.60908	0
C	2.401606	-2.99201	0

C	1.169824	-3.63962	0
C	-0.00944	-2.90619	0
H	0.558868	4.464582	0
H	2.847173	3.602827	0
H	3.350621	1.184137	0
H	3.443882	-1.11618	0
H	3.312302	-3.57652	0
H	1.121204	-4.72052	0
H	-0.97123	-3.40071	0
O	-1.63006	2.859388	0

3 Dimer
Eopt -1668.5327
ΔG -1668.2790

C	-2.17911	-3.51298	0.10985
C	-3.53876	-3.76931	0.038348
C	-4.49991	-2.75985	-0.02878
C	-4.0645	-1.43572	-0.02227
C	-2.69613	-1.2306	0.049138
C	-1.66231	-2.18352	0.118677
C	-4.88965	-0.22728	-0.08276
C	-4.22236	1.000235	-0.06582
I	-2.14131	0.772107	0.047853
C	-6.28244	-0.1941	-0.1547
C	-6.94968	1.018399	-0.20759
C	-6.24873	2.221026	-0.18986
C	-4.86223	2.223546	-0.11731
H	-1.46775	-4.3287	0.161013
H	-3.86939	-4.80147	0.034256
H	-5.55374	-2.99568	-0.08541
H	-6.83758	-1.12335	-0.16731
H	-8.03044	1.029811	-0.26323
H	-6.78058	3.162398	-0.23177
H	-4.31057	3.154177	-0.10181
C	2.179274	3.513071	0.109702
C	3.538932	3.769318	0.03837
C	4.500004	2.759772	-0.02864
C	4.064488	1.435686	-0.02219
C	2.696091	1.230619	0.049104
C	1.662347	2.18364	0.11849
C	4.889635	0.227239	-0.08267
C	4.222328	-1.00026	-0.06586
I	2.141276	-0.77214	0.047657
C	6.282426	0.194038	-0.15448
C	6.949653	-1.01846	-0.2074
C	6.248675	-2.22108	-0.18983
C	4.862179	-2.22358	-0.11739
H	1.467928	4.328813	0.16073
H	3.869633	4.801456	0.034314
H	5.553868	2.995513	-0.08518
H	6.837589	1.123279	-0.16695
H	8.030418	-1.02989	-0.26294
H	6.78052	-3.16246	-0.23179
H	4.310493	-3.1542	-0.10202
O	0.436797	1.851709	0.179829
O	-0.43681	-1.85138	0.180327

4 Monomer
Eopt -834.2445
ΔG -834.1307

C	3.434456	0.57708	-0.00053
C	2.858319	1.902319	-0.00014
C	1.509418	2.13419	-5.3E-05
C	0.568241	1.083898	-9.9E-05
C	1.152035	-0.18385	-0.00012
C	2.47157	-0.50806	-0.00023
C	-0.87352	1.204025	0.000005
C	-1.64127	0.036911	0.000049
I	-0.39403	-1.64551	0.000039
C	-1.60725	2.398406	0.000066
C	-2.99087	2.378943	0.000141
C	-3.70109	1.180388	0.000169
C	-3.01726	-0.02815	0.000127
H	3.549683	2.73627	0.000022
H	1.144286	3.155052	0.000108
H	-1.07847	3.342994	0.000048
H	-3.53079	3.317086	0.000181
H	-4.78268	1.180834	0.000235
H	-3.54636	-0.97137	0.000164
H	2.835658	-1.52575	-0.00018
O	4.664415	0.365018	0.000127

4 Dimer
Eopt -1668.5101
ΔG -1668.2564

C	-0.41821	-2.84605	-1.05954
C	-1.23827	-3.57358	-0.12706
C	-2.30574	-3.00624	0.518364
C	-2.6676	-1.65681	0.3254
C	-1.87292	-0.97509	-0.59216
C	-0.8309	-1.4905	-1.31072
C	-3.76399	-0.9481	0.958673
C	-3.93889	0.40115	0.641339
I	-2.44857	1.063058	-0.6708
C	-4.68959	-1.48699	1.859837
C	-5.70051	-0.70078	2.385779
C	-5.82808	0.641803	2.038196

C	-4.92997	1.215184	1.147739
H	-0.97641	-4.60731	0.063148
H	-2.88503	-3.60348	1.213435
H	-4.61213	-2.52917	2.142134
H	-6.40479	-1.14091	3.080015
H	-6.62298	1.244481	2.456437
H	-5.0098	2.257156	0.867702
C	0.418173	2.845777	-1.06024
C	1.237744	3.573315	-0.12734
C	2.305067	3.006072	0.518412
C	2.667242	1.656742	0.325365
C	1.873078	0.975024	-0.59265
C	0.831212	1.490345	-1.31153
C	3.763499	0.948142	0.958999
C	3.938778	-0.40104	0.641586
I	2.449194	-1.063	-0.67135
C	4.688577	1.487081	1.860667
C	5.69938	0.700982	2.386995
C	5.827347	-0.64154	2.039308
C	4.929749	-1.21497	1.148365
H	0.9756	4.606964	0.06293
H	2.883951	3.603301	1.213831
H	4.610799	2.529209	2.143071
H	6.403249	1.141138	3.081624
H	6.622158	-1.24413	2.45784
H	5.0099	-2.25689	0.868223
H	-0.3219	-0.94576	-2.09225
H	0.322684	0.945718	-2.09345
O	-0.60823	3.324736	-1.61133
O	0.608303	-3.32509	-1.61036

5 Monomer
Eopt -947.6362
ΔG -947.5112

C	0.242332	3.251372	0
C	1.622651	3.063444	0
C	2.184544	1.792456	0
C	1.360593	0.664746	0
C	0	0.919029	0
C	-0.60391	2.149495	0
C	1.760218	-0.74923	0
C	0.727578	-1.69105	0
I	-1.17235	-0.77303	0
C	3.073693	-1.21816	0
C	3.321886	-2.58112	0
C	2.273439	-3.49665	0
C	0.957023	-3.05446	0
H	-0.18776	4.244513	0
H	2.27539	3.926749	0
H	3.260882	1.678632	0
H	3.899322	-0.51771	0
H	4.344017	-2.93667	0
H	2.48035	-4.55893	0
H	0.138678	-3.76341	0
C	-2.12161	2.223913	0
O	-2.66036	3.322848	0
O	-2.69798	1.084019	0

5 Dimer
Eopt -1895.2864
ΔG -1895.0144

C	4.83706	-3.50999	0.000287
C	5.458297	-2.26785	0.000387
C	4.690072	-1.10208	0.000224
C	3.314518	-1.27746	-3.7E-05
C	5.169295	0.285412	0.000305
C	4.188524	1.281118	0.000112
I	2.235524	0.482281	-0.00027
C	6.507263	0.679285	0.000562
C	6.831304	2.026259	0.000621
C	5.834735	2.998201	0.000424
C	4.49584	2.629902	0.000168
H	5.446454	-4.4044	0.000411
H	6.538841	-2.20418	0.000591
H	7.292147	-0.06681	0.000721
H	7.871564	2.324778	0.000823
H	6.099601	4.047571	0.000469
H	3.718817	3.384211	0.000013
C	3.449464	-3.62669	0.000033
C	2.657858	-2.48498	-0.00013
H	2.969442	-4.59665	-3.9E-05
C	-2.65793	2.485005	-6.9E-05
C	-3.31455	1.27746	0.000017
C	-3.44958	3.626683	0.000072
C	-4.6901	1.102028	0.000241
I	-2.23549	-0.48224	-0.00017
C	-4.83717	3.509934	0.00029
H	-2.96959	4.596663	0.00001
C	-5.45837	2.267771	0.000377
C	-5.16927	-0.28548	0.000306
C	-4.18847	-1.28115	0.000133
H	-5.4466	4.404328	0.000396
H	-6.53891	2.204067	0.00055
C	-6.50723	-0.6794	0.000524
C	-4.49573	-2.62994	0.000169
C	-6.83122	-2.02639	0.000563

H	-7.29214	0.066658	0.000664
C	-5.83461	-2.99829	0.000386
H	-3.71868	-3.38422	0.000031
H	-7.87147	-2.32494	0.000734
H	-6.09944	-4.04767	0.000417
C	1.140278	-2.5029	-0.00041
C	-1.14035	2.502983	-0.0003
O	0.609582	-1.34302	-0.00049
O	0.551365	-3.57807	-0.00044
O	-0.55149	3.578189	-0.00033
O	-0.6096	1.34313	-0.00047

6 Monomer
Eopt -947.6048
ΔG -947.4818

C	-2.85844	0.504809	0.000023
C	-2.38504	1.815249	0.000023
C	-1.03003	2.101636	0.000015
C	-0.09327	1.06699	0.00001
C	-0.6197	-0.22263	0.000014
C	-1.95266	-0.55114	0.00002
C	1.360756	1.219827	0
C	2.141878	0.066717	-4E-06
I	0.931387	-1.64042	0.000008
C	2.055678	2.430593	-0.00001
C	3.440258	2.442225	-2.2E-05
C	4.170062	1.25731	-2.4E-05
C	3.518808	0.030907	-1.5E-05
H	-3.11209	2.616257	0.000025
H	1.50378	3.361445	-9E-06
H	3.962015	3.389967	-0.00003
H	5.251626	1.281064	-3.3E-05
H	4.072314	-0.89798	-1.6E-05
H	-2.323	-1.56718	0.00002
C	-4.37038	0.198494	0.000016
O	-5.1171	1.192342	0.000014
O	-4.67	-1.00936	-9.9E-05
H	-0.6889	3.128835	0.000012

6 Dimer
Eopt -1895.2669
ΔG -1894.9919

C	-0.86904	-2.31522	-0.39706
C	-1.67166	-3.19718	0.325267
C	-2.95674	-2.84803	0.707499
C	-3.46418	-1.57715	0.419626
C	-2.62145	-0.72484	-0.28765
C	-1.371	-1.06638	-0.73946
C	-4.78498	-1.06497	0.792363
C	-5.06826	0.252017	0.429683
I	-3.44632	1.184918	-0.52664
C	-5.7715	-1.78324	1.468906
C	-6.98603	-1.18458	1.75915
C	-7.23878	0.132841	1.386292
C	-6.27209	0.869595	0.715361
H	-1.26995	-4.16652	0.589635
H	-5.58711	-2.80783	1.765353
H	-7.74701	-1.74866	2.28253
H	-8.19174	0.589972	1.618851
H	-6.46354	1.895559	0.427802
C	0.869123	2.315344	-0.39697
C	1.671826	3.197294	0.325283
C	2.956902	2.848069	0.70748
C	3.464261	1.577146	0.419618
C	2.621452	0.724882	-0.28763
C	1.370997	1.066471	-0.73936
C	4.785009	1.064871	0.792381
C	5.06819	-0.25215	0.429734
I	3.446253	-1.18488	-0.52678
C	5.771566	1.783053	1.468963
C	6.986029	1.184284	1.759272
C	7.238675	-0.13316	1.386439
C	6.271947	-0.86983	0.715472
H	1.27018	4.166661	0.589656
H	5.587249	2.80766	1.7654
H	7.747026	1.7483	2.282685
H	8.191587	-0.59038	1.619043
H	6.463323	-1.89581	0.427917
H	-0.80418	-0.37461	-1.33245
H	0.804099	0.374714	-1.33228
C	-0.57724	2.65528	-0.73513
C	0.577326	-2.65511	-0.73526
O	-1.05433	3.694832	-0.2838
O	1.054583	-3.69444	-0.28361
O	1.194155	-1.78554	-1.4366
O	-1.19424	1.785554	-1.43613
H	3.568754	3.55483	1.253249
H	-3.56854	-3.55482	1.253291

14 Monomer
Eopt -1461.5426
ΔG -1461.3689

C	2.714148	0.526987	-0.00834
C	2.185615	1.816891	-0.0112
C	0.820116	2.026553	-0.01067
C	-0.06048	0.941283	-0.00698
C	0.526644	-0.31935	-0.00599

C	1.879651	-0.57896	-0.00674
C	-1.52077	1.022055	-0.00372
C	-2.23512	-0.17268	-0.00068
I	-0.93898	-1.81634	-0.00222
C	-2.27747	2.190008	-0.00191
C	-3.66834	2.136696	0.002533
C	-4.30985	0.897687	0.005261
C	-3.60761	-0.30635	0.003959
H	2.859249	2.664587	-0.01462
H	-1.77871	3.152135	-0.00334
H	2.294564	-1.57766	-0.00652
O	4.955137	1.016953	-1.1745
O	4.690454	-1.13435	-0.05551
H	0.425169	3.033673	-0.01334
S	4.497265	0.303908	0.005002
O	4.918856	0.908917	1.256495
H	-5.39304	0.859631	0.008888
C	-4.27954	-1.64414	0.008114
H	-3.99767	-2.22181	-0.87701
H	-3.99593	-2.21688	0.895873
H	-5.36192	-1.53577	0.008886
C	-4.4632	3.410582	0.004802
H	-4.22138	4.010463	0.883875
H	-4.22618	4.010696	-0.87541
H	-5.53292	3.208558	0.007729

14 Dimer
Eopt -2923.1315
ΔG -2922.7548

C	0.872043	2.447889	-0.844
C	1.531256	3.338289	-0.00067
C	2.700208	2.96591	0.637455
C	3.225326	1.681992	0.465102
C	2.516977	0.830885	-0.38003
C	1.36936	1.172819	-1.05974
C	4.441704	1.160636	1.091144
C	4.777388	-0.15835	0.80663
I	3.383192	-1.06916	-0.45086
C	5.292489	1.873795	1.936933
C	6.426364	1.272944	2.463909
C	6.710458	-0.05851	2.141114
C	5.895092	-0.81115	1.30613
H	1.120226	4.327039	0.158003
H	5.071408	2.905007	2.182559
C	-0.87207	-2.44787	-0.84416
C	-1.53111	-3.33821	-0.00063
C	-2.70002	-2.96583	0.637583
C	-3.22522	-1.68196	0.465154
C	-2.51703	-0.8309	-0.38016
C	-1.36951	-1.17287	-1.06002
C	-4.44157	-1.16061	1.091265
C	-4.77739	0.158311	0.8066
I	-3.38333	1.06909	-0.45106
C	-5.29221	-1.87371	1.937247
C	-6.42608	-1.27287	2.464247
C	-6.71032	0.058513	2.14129
C	-5.89509	0.811095	1.306127
H	-1.11998	-4.3269	0.158129
H	-5.07102	-2.90486	2.183021
H	0.886577	0.480004	-1.7304
H	-0.88696	-0.48015	-1.73095
O	1.382663	-3.70778	-0.69183
O	-1.38278	3.707615	-0.69149
O	-1.29212	1.696666	-2.04203
O	1.292143	-1.69661	-2.04205
H	-3.20487	-3.6698	1.285727
H	3.205175	3.669928	1.285467
S	-0.62475	2.959306	-1.67799
S	0.624685	-2.95927	-1.67823
O	-0.19524	3.722275	-2.827
O	0.195157	-3.72203	-2.82736
H	-7.59855	0.523785	2.554957
H	7.598689	-0.5238	2.554763
C	6.19486	-2.23756	0.957611
H	5.385688	-2.89785	1.282774
H	6.315854	-2.35742	-0.12281
H	7.11264	-2.57107	1.437702
C	7.351269	2.030674	3.372352
H	8.358198	2.065096	2.952406
H	7.005977	3.051911	3.524906
H	7.420983	1.539944	4.344903
C	-6.19499	2.23744	0.957443
H	-5.38581	2.897824	1.282394
H	-6.31614	2.357132	-0.12298
H	-7.11272	2.570973	1.437607
C	-7.35083	-2.03054	3.37291
H	-8.35783	-2.06499	2.953145
H	-7.00551	-3.05176	3.525487
H	-7.42037	-1.53973	4.345436

30 Monomer
Eopt -1697.3826
ΔG -1697.0437

C	2.106884	-2.16231	0.130061
C	2.698296	-1.20169	-0.69716
C	1.902709	-0.17861	-1.20779

C	0.569716	-0.18048	-0.86144
C	-0.05798	-1.11323	-0.04324
C	0.76429	-2.12694	0.455435
H	2.722972	-2.96199	0.524063
H	2.330148	0.580995	-1.84946
H	0.342286	-2.89003	1.096575
C	-2.14558	0.116516	-0.37735
C	-3.48371	0.415855	-0.2487
C	-4.22654	-0.44141	0.546875
C	-3.62595	-1.5371	1.164681
C	-2.27856	-1.79701	1.003117
C	-1.48807	-0.95782	0.212021
H	-3.95388	1.26231	-0.73031
H	-4.22925	-2.19364	1.778892
H	-1.82936	-2.65284	1.48968
I	-0.80689	1.255357	-1.51825
S	-5.97829	-0.13144	0.801749
O	-6.08373	0.147306	2.22352
O	-6.62392	-1.36802	0.397572
O	-6.27879	1.00526	-0.05111
C	4.168616	-1.2604	-1.00157
H	4.399479	-0.5835	-1.82888
H	4.405302	-2.27359	-1.33149
C	5.056688	-0.89947	0.203503
H	4.851024	-1.62738	0.998981
C	6.538651	-1.0118	-0.16653
H	6.777994	-0.22729	-0.89366
H	7.132541	-0.79849	0.724236
C	6.958475	-2.37049	-0.71605
H	6.550083	-2.55791	-1.70985
H	8.044923	-2.43418	-0.7938
H	6.624746	-3.17732	-0.05814
C	4.716048	0.495125	0.735345
H	4.847395	1.219088	-0.07728
H	3.655505	0.525175	1.007482
C	5.533331	0.923799	1.952581
H	5.584288	0.08743	2.657615
H	6.562376	1.14405	1.654875
C	4.956246	2.145392	2.665257
H	3.952942	1.906822	3.031799
H	5.565603	2.362552	3.546179
C	4.892513	3.385588	1.781383
H	5.875574	3.613203	1.360836
H	4.198015	3.250905	0.950105
H	4.561919	4.257352	2.348446

30 Dimer
Eopt -3394.8125
ΔG -3394.1070

C	-5.55664	1.585105	-2.33997
C	-6.19283	1.273065	-1.13319
C	-5.62183	0.308796	-0.3074
C	-4.45117	-0.29856	-0.71858
C	-3.79802	-0.00162	-1.91136
C	-4.38332	0.967304	-2.72897
H	-6.00193	2.330728	-2.98837
H	-6.09345	0.051115	0.633165
H	-3.91928	1.231025	-3.67099
C	-2.15048	-1.69215	-1.27396
C	-1.02687	-2.47568	-1.40546
C	-0.23825	-2.25279	-2.52482
C	-0.59922	-1.30447	-3.4767
C	-1.75769	-0.56345	-3.32394
C	-2.56717	-0.73666	-2.1992
H	-0.77453	-3.22693	-0.67329
H	0.038036	-1.14357	-4.33645
H	-2.03257	0.167421	-4.07281
I	-3.42093	-1.7413	0.38605
S	1.254369	-3.21388	-2.73783
O	2.190298	-2.33162	-3.40975
O	0.872685	-4.39145	-3.48091
O	1.683381	-3.52167	-1.35977
C	-7.44995	1.993143	-0.73259
H	-7.9259	1.466892	0.099842
H	-8.14198	1.964494	-1.57624
C	-7.2106	3.4583	-0.32374
H	-6.77191	3.977629	-1.18588
C	-8.53436	4.148836	0.01765
H	-8.93788	3.69988	0.932545
H	-8.32641	5.194562	0.252484
C	-9.58361	4.099593	-1.08738
H	-9.98082	3.09463	-1.23438
H	-10.4259	4.750308	-0.84707
H	-9.16409	4.437278	-2.03886
C	-6.2188	3.542876	0.839302
H	-6.61934	2.963271	1.679022
H	-5.28277	3.055053	0.546963
C	-5.89419	4.96421	1.295017
H	-5.71872	5.588546	0.412537
H	-6.75228	5.396498	1.817518
C	-4.67431	5.03791	2.211575
H	-3.79549	4.674567	1.669743
H	-4.47769	6.084815	2.456626
C	-4.83974	4.242949	3.501559

H	-5.74324	4.551195	4.034265
H	-4.91778	3.17198	3.306265
H	-3.99028	4.394189	4.169713
C	5.556705	1.585285	2.340011
C	6.19287	1.273378	1.133188
C	5.621945	0.309074	0.307382
C	4.451366	-0.29843	0.718586
C	3.798218	-0.0016	1.911395
C	4.383458	0.967347	2.729034
H	6.00194	2.330924	2.988436
H	6.093563	0.051468	-0.6332
H	3.919432	1.230974	3.671081
C	2.150759	-1.69219	1.273919
C	1.027198	-2.4758	1.405378
C	0.238572	-2.25302	2.524754
C	0.599504	-1.30472	3.476684
C	1.757924	-0.56363	3.323967
C	2.567417	-0.73672	2.199214
H	0.774869	-3.227	0.673147
H	-0.03777	-1.14391	4.336438
H	2.032749	0.167209	4.072886
I	3.421216	-1.74118	-0.3861
S	-1.25399	-3.2142	2.737705
O	-2.18993	-2.33205	3.409755
O	-0.87222	-4.39184	3.480626
O	-1.68304	-3.52184	1.359618
C	7.449847	1.993659	0.732503
H	7.925771	1.467544	-0.10003
H	8.141986	1.965063	1.57607
C	7.210207	3.458813	0.323802
H	6.771782	3.97806	1.186121
C	8.533773	4.149498	-0.01807
H	8.936878	3.700743	-0.93324
H	8.325646	5.195254	-0.25261
C	9.58355	4.100099	1.086445
H	9.980891	3.095128	1.233044
H	10.42567	4.750922	0.845862
H	9.164475	4.437557	2.038204
C	6.217984	3.54335	-0.83889
H	6.618258	2.963735	-1.67874
H	5.282063	3.055527	-0.54622
C	5.893198	4.964668	-1.29453
H	5.717913	5.588995	-0.412
H	6.75114	5.396998	-1.81724
C	4.673082	5.03831	-2.21078
H	3.794418	4.6749	-1.66874
H	4.476341	6.085211	-2.45575
C	4.838244	4.243402	-3.50083
H	5.741568	4.55177	-4.03377
H	4.916462	3.172437	-3.30559
H	3.988582	4.394561	-4.16874

31 Monomer
Eopt -1928.4365
ΔG -1928.0219

C	-5.54178	0.64663	0.277555
C	-4.91464	1.774302	0.804901
C	-3.537	1.87226	0.842092
C	-2.74312	0.833456	0.345641
C	-3.42779	-0.26668	-0.15929
C	-4.79599	-0.41034	-0.21888
C	-1.28335	0.805339	0.318813
C	-0.65484	-0.32988	-0.18451
I	-2.07783	-1.72581	-0.82419
C	-0.43091	1.82033	0.75515
C	0.94023	1.663178	0.689138
C	1.53149	0.497589	0.187241
C	0.702626	-0.52955	-0.27091
H	-5.52069	2.584292	1.190886
H	-0.84903	2.734162	1.155747
H	1.575171	2.458451	1.05733
H	1.122037	-1.43278	-0.6937
H	-5.2874	-1.28462	-0.62339
O	-7.71312	0.626645	1.65502
O	-7.63851	-0.7051	-0.38689
H	-3.06787	2.753188	1.260965
S	-7.33692	0.56307	0.253507
O	-7.73678	1.728879	-0.51456
C	3.004422	0.346572	0.144113
C	3.593709	-0.88689	0.410823
C	3.813711	1.440729	-0.15295
C	4.977074	-1.04324	0.389017
H	2.960214	-1.72845	0.665664
C	5.200152	1.320763	-0.18968
H	3.349551	2.392648	-0.38055
C	5.759332	0.071931	0.08546
H	6.832936	-0.03525	0.064162
C	6.049168	2.546281	-0.53255
C	5.582917	-2.41184	0.705703
C	5.168953	-2.83466	2.121866
H	4.085079	-2.91683	2.215706
H	5.602436	-3.80854	2.360771
H	5.521845	-2.11253	2.861422
C	5.058455	-3.44443	-0.30139
H	5.485965	-4.42616	-0.08489

H	3.971799	-3.53347	-0.25543
H	5.335687	-3.16802	-1.32112
C	7.10939	-2.40214	0.63409
H	7.465741	-2.13361	-0.36265
H	7.543009	-1.70585	1.354982
H	7.485674	-3.40022	0.865868
C	7.546508	2.241978	-0.50678
H	7.87389	1.90683	0.479683
H	7.8164	1.477503	-1.23831
H	8.101075	3.149471	-0.7525
C	5.680552	3.040512	-1.93816
H	4.628997	3.324964	-2.00093
H	6.282436	3.915457	-2.19449
H	5.869301	2.265076	-2.68391
C	5.766427	3.661266	0.483694
H	4.718239	3.963817	0.468489
H	6.013356	3.335556	1.496612
H	6.372631	4.539439	0.249669

31 Dimer
Eopt -3856.921
ΔG -3856.0620

C	0.273517	-2.49519	2.519164
C	0.645716	-1.54472	3.464423
C	1.800349	-0.80164	3.29391
C	2.593291	-0.97489	2.15757
C	2.165616	-1.93226	1.239173
C	1.046412	-2.71848	1.388767
C	3.818	-0.238	1.851454
C	4.455791	-0.53709	0.649594
I	3.409617	-1.97948	-0.44123
C	4.410299	0.735788	2.655911
C	5.576013	1.357916	2.251206
C	6.197999	1.042382	1.036562
C	5.616015	0.071403	0.219603
H	0.020497	-1.38392	4.332964
H	3.961615	0.999598	3.60512
H	6.030823	2.094416	2.900987
H	6.061005	-0.17772	-0.73517
H	0.785153	-3.47206	0.662182
O	-0.8195	-4.6391	3.484547
O	-1.66681	-3.76049	1.381639
H	2.084958	-0.06936	4.037738
S	-1.21458	-3.45842	2.754016
O	-2.13979	-2.57963	3.444527
C	7.447595	1.721921	0.620556
C	8.436502	1.022507	-0.06789
C	7.645555	3.069409	0.913451
C	9.616158	1.645626	-0.46631
H	8.28886	-0.03125	-0.27298
C	8.811097	3.726848	0.528902
H	6.865067	3.615926	1.429423
C	9.781852	2.996717	-0.15824
H	10.69081	3.493181	-0.46137
C	8.98007	5.209916	0.864119
C	10.67694	0.836562	-1.21544
C	11.12838	-0.34363	-0.34413
H	10.2991	-1.01359	-0.11217
H	11.89144	-0.92248	-0.86968
H	11.55505	0.009668	0.597246
C	10.07478	0.297874	-2.52061
H	10.82484	-0.27954	-3.06613
H	9.221397	-0.35499	-2.33031
H	9.74135	1.117197	-3.16144
C	11.90723	1.673454	-1.5642
H	11.65365	2.514647	-2.21278
H	12.3975	2.062723	-0.6694
H	12.62767	1.048184	-2.09477
C	10.31435	5.769748	0.372382
H	11.16185	5.255056	0.829967
H	10.40922	5.693827	-0.71287
H	10.38071	6.825929	0.640175
C	7.848602	6.011371	0.205943
H	6.868894	5.69154	0.564131
H	7.961864	7.073227	0.436588
H	7.869971	5.892904	-0.87969
C	8.910041	5.396193	2.386017
H	7.9533	5.05879	2.787822
H	9.705126	4.835647	2.882727
H	9.02767	6.452763	2.637833
C	-0.27325	-2.4936	-2.52022
C	-0.64693	-1.54455	-3.46636
C	-1.80183	-0.80194	-3.29567
C	-2.59384	-0.9745	-2.15854
C	-2.16493	-1.93072	-1.23955
C	-1.04506	-2.71603	-1.38896
C	-3.81865	-0.23791	-1.85213
C	-4.4554	-0.5363	-0.64954
I	-3.40827	-1.97796	0.441364
C	-4.41192	0.73504	-2.65689
C	-5.57757	1.357033	-2.25179
C	-6.19855	1.042146	-1.03647
C	-5.61559	0.072015	-0.2192
H	-0.02263	-1.38437	-4.33568
H	-3.96404	0.998314	-3.60663

H	-6.03314	2.092885	-2.90177
H	-6.05983	-0.17659	0.736054
H	-0.78225	-3.46804	-0.66139
O	0.820584	-4.63604	-3.4877
O	1.666816	-3.76001	-1.38325
H	-2.08746	-0.07062	-4.04004
S	1.215149	-3.45627	-2.75544
O	2.140536	-2.57647	-3.44446
C	-7.44815	1.721439	-0.6201
C	-8.43636	1.022021	0.069354
C	-7.64685	3.068667	-0.91367
C	-9.61604	1.644882	0.468093
H	-8.28815	-0.03155	0.274988
C	-8.81243	3.725854	-0.52882
H	-6.86689	3.615188	-1.43044
C	-9.78249	2.995722	0.159312
H	-10.6915	3.491983	0.462674
C	-8.98218	5.208671	-0.86476
C	-10.6761	0.8358	1.218295
C	-11.1277	-0.34488	0.347772
H	-10.2984	-1.01464	0.115465
H	-11.8902	-0.9238	0.874088
H	-11.5552	0.007912	-0.59343
C	-10.0728	0.297845	2.523283
H	-10.8224	-0.27958	3.06954
H	-9.21937	-0.35483	2.332689
H	-9.73924	1.117543	3.163546
C	-11.9064	1.672448	1.567562
H	-11.6526	2.513991	2.215614
H	-12.3974	2.061192	0.672942
H	-12.6262	1.04717	2.098889
C	-10.3165	5.768193	-0.3728
H	-11.164	5.252886	-0.82976
H	-10.4109	5.692829	0.71254
H	-10.3834	6.824199	-0.64114
C	-7.85079	6.010939	-0.20743
H	-6.87109	5.691317	-0.56581
H	-7.96458	7.072628	-0.43858
H	-7.87171	5.893031	0.878271
C	-8.91281	5.394191	-2.38678
H	-7.95608	5.056989	-2.78878
H	-9.70785	4.833049	-2.88289
H	-9.03099	6.45058	-2.63909

References

[1] M. Meyer, W . Paciorek, A. Kowalski, A. Muszynski, A. W isniewski, M. Pol, M. Przewozniczek, P. Stec, D. Bujnik, H. Kulza, *CrysAlisPro* **2018**.
[2] L. J. Farrugia, *J. Appl. Crystallogr.* **1999**, *32*, 837.
[3] G. M. Sheldrick, *Acta Crystallogr. Sect. A, Found Crystallogr.* **2008**, *64*, 112.
[4] C. B. Hübschle, G. M. Sheldrick, B. Dittrich, *J. Appl. Crystallogr.* **2011**, *44*, 1281.
[5] K. B. H. Putz, can be found under http://www.crystalimpact.com/diamond.
[6] K. Masahumi, N. Kazuhiro, Y. Hideki, O. Naohito, K. Atsuyuki, N. Tsuyoshi, M. Akira, *US-B1 6169107* **2001**.
[7] T. Enta, G. Yoshino, Y. Kuninobu, *ChemRxiv* **2025**.
[8] E. R. Barth, D. Längle, F. Wesseler, C. Golz, A. Krupp, D. Schade, C. Strohmann, *Eur. J. Inorg. Chem.* **2020**, *2020*, 176.
[9] H. A. Wegner, H. Reisch, K. Rauch, A. Demeter, K. A. Zachariasse, A. de Meijere, L. T. Scott, *J. Org. Chem.* **2006**, *71*, 9080.
[10] H. Tomori, J. M. Fox, S. L. Buchwald, *J. Org. Chem.* **2000**, *65*, 5334.
[11] D. Chen, G. Shi, H. Jiang, Y. Zhang, Y. Zhang, *Org. Lett.* **2016**, *18*, 2130.
[12] D. Cao, M. Hong, A. K. Blackburn, Z. Liu, J. M. Holcroft, J. F. Stoddart, *Chem. Sci.* **2014**, *5*, 4242.
[13] M. Bielawski, M. Zhu, B. Olofsson, *Adv. Synth. Catal.* **2007**, *349*, 2610.
[14] No measurement of 13C NMR was possible due to low solubility.
[15] T. Ami, K. Oka, K. Tsuchiya, N. Tohnai, *Angew. Chem. Int. Ed.* **2022**, *61*, e202202597.
[16] Y. Zou, S. Li, R. Wang, L. Xu, X. Xu, H. Xu, J. Xu, *Tetrahedron Lett.* **2024**, *143*, 155116.
[17] S. Maity, D. Das, S. Sarkar, R. Samanta, *Org. Lett.* **2018**, *20*, 5167.
[18] M. Tashiro, T. Yamato, *J. Org. Chem.* **1979**, *44*, 3037.
[19] Elemental analysis could not be measured due to problems with sample preperation.
[20] No measurement of 13C NMR or mass spectroscopy was possible due to bad solubility.
[21] F. Perrin, *J. Phys. Radium* **1936**, *7*, 1.
[22] H. C. Chen, S. H. Chen, *J. Phys. Chem.* **1984**, *88*, 5118.
[23] Gaussian 16, Revision B.01, Frisch, M.J., Trucks, G.W., Schlegel, H.B., Scuseria, G.E., Robb, M.A., Cheeseman, J.R., G. Scalmani, V. Barone, G. A. Petersson, H. Nakatsuji, X. Li, M. Caricato, A. V. Marenich, Bloino, J., Janesko, B.G., Gomperts, R., Mennucci, B., Hratchian, H.P., Ortiz, J.V., Izmaylov, A.F., Sonnenberg, J.L., Williams-Young, D., Ding, F., Lipparini, F., Egidi, F., Goings, J., Peng, B., Petrone, A., Henderson, T., Ranasinghe, D., Zakrzewski, V.G., Gao, J., Rega, N., Zheng, G., Liang, W., Hada, M., Ehara, M., Toyota, K., Fukuda, R., Hasegawa, J., Ishida, M., Nakajima, T., Honda, Y., Kitao, O., Nakai, H., Vreven, T., Throssell, K., Montgomery Jr., J.A., Peralta, J.E., Ogliaro, F., Bearpark, M.J., Heyd, J.J., Brothers, E.N., Kudin, K.N., Staroverov, V.N., Keith, T.A., Kobayashi, R., Normand, J., Raghavachari, K., Rendell, A.P., Burant, J.C., Iyengar, S.S., Tomasi, J., Cossi, M., Millam, J.M., Klene, M., Adamo, C., Cammi, R., Ochterski, J.W., Martin, R.L., Morokuma, K., Farkas, O., Foresman, J.B., Fox, D.J., *Gaussian Inc.* **2016**.
[24] Y. Zhao, D. G. Truhlar, *Theor. Chem. Acc.* **2008**, *120*, 215.
[25] S. Grimme, *J. Comput. Chem.* **2006**, *27*, 1787.
[26] S. Grimme, J. Antony, S. Ehrlich, H. Krieg, *J. Chem. Phys.* **2010**, *132*, 154104.
[27] F. Weigend, R. Ahlrichs, *PCCP* **2005**, *7*, 3297.
[28] D. Rappoport, F. Furche, *J. Chem. Phys.* **2010**, *133*, 134105.
[29] E. Engelage, D. Reinhard, S. M. Huber, *Chem. Eur. J.* **2020**, *26*, 3843.
[30] K. A. Peterson, *J. Chem. Phys.* **2003**, *119*, 11099.
[31] E. Engelage, N. Schulz, F. Heinen, S. M. Huber, D. G. Truhlar, C. J. Cramer, *Chem. Eur. J.* **2018**, *24*, 15983.
[32] Ignacio Funes-Ardoiz, Robert S. Paton, *GoodVibes: version 2.0.3*, Zenodo, **2018**.
[33] S. Grimme, *Chem. Eur. J.* **2012**, *18*, 9955.
